# Synthesis of isoluminol derivatives with a terminal carboxyl group for protein labelling†

**DOI:** 10.1039/d5ra00677e

**Published:** 2025-05-28

**Authors:** Qiao Ma, Lei Liu, Chunfeng Luo, Yue Wu, Fengshu Qin, Kai Du

**Affiliations:** a Shenzhen New Industries Biomedical Engineering Co., Ltd, Reagent Key Raw Materials R&D and Production Center Pingshan District Shenzhen Guangdong 518122 P. R. China kai.du@snibe.cn

## Abstract

*N*-(4-Aminobutyl)-*N*-ethylisoluminol (ABEI) as an efficient label is widely used in automated immunoassays, yet the labelling process of ABEI onto biomacromolecules like proteins is not straightforward as the transformation of an amino group into a carboxyl group is needed. We report the synthesis of isoluminol derivatives with a terminal carboxyl group for protein labelling. And also, an *N*-hydroxysuccinimide active ester of carboxylic acid as a direct protein labelling reagent was isolated with high purity. This labelling reagent was proved to have good chemiluminescence efficiency, be stable and possess high labelling efficacy with BSA. The introduction of the propyl sulfonic group as a hydrophilic functional group increased not only water solubility but also the chemiluminescence quantum yields. In addition, non-specific binding of magnetic microparticles was also decreased notably when water soluble labelling reagents were used.

## Abstract 2

We synthesized isoluminol derivatives with a terminal carboxyl group. The resulting NHS ester is a stable, efficient chemiluminescent reagent with high protein labelling efficacy.

## Graphical abstract

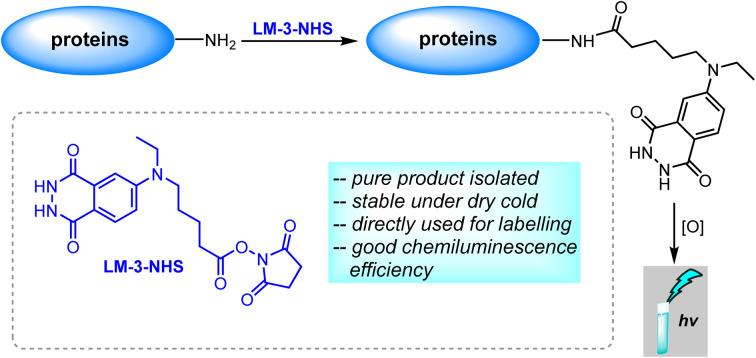

## Introduction

Biological research often requires the use of small molecule labels,^1^ such as fluorophores,^2^ radionuclides,^3^ and chemiluminescent molecules,^4^ that can covalently bind to the proteins of interest, thereby assisting in detection or tracing. The labelled proteins not only retain the activity of original proteins but also possess the characteristics of the labels. NHS-activated esters of terminal carboxyl groups and isothiocyanates are common forms of utilization for small-molecule labels, examples include the fluorescent label FITC^5^ and the chemiluminescent label tris(bipyridine)ruthenium.^6^ For an ideal small molecule label, the following criteria are essential: (1) it must be readily synthesized to yield a chemically pure substance with reasonable water solubility. (2) Post-labelling, it should neither impinge on the activity of the target protein nor alter the inherent properties of the labelling molecule. Moreover, it needs to maintain sufficient stability under physiological conditions. (3) Cost-effectiveness is also a key requirement, with the label being inexpensive to produce.

Since luminol, isoluminol and their derivatives produce light with high quantum efficiencies *via* simple oxidation reactions, they can be used as universal labelling reagents.^7^ Although the quantum efficiency of isoluminol is merely around 10% in comparison to luminol, it should be noted that substitution of the aryl amino group enhances the efficiency by a factor of 10. In contrast, similar substitution on luminol results in a conspicuous decrease,^8^ likely attributable to steric hindrance,^9^ intramolecular quenching,^8*d*^ or label polymerization.^8*e*^ Therefore, isoluminol derivatives are superior as labelling reagents, and the labelled objects are typically attached to the amino residue of isoluminol through an alkyl-bridging group.

**Scheme 1:**
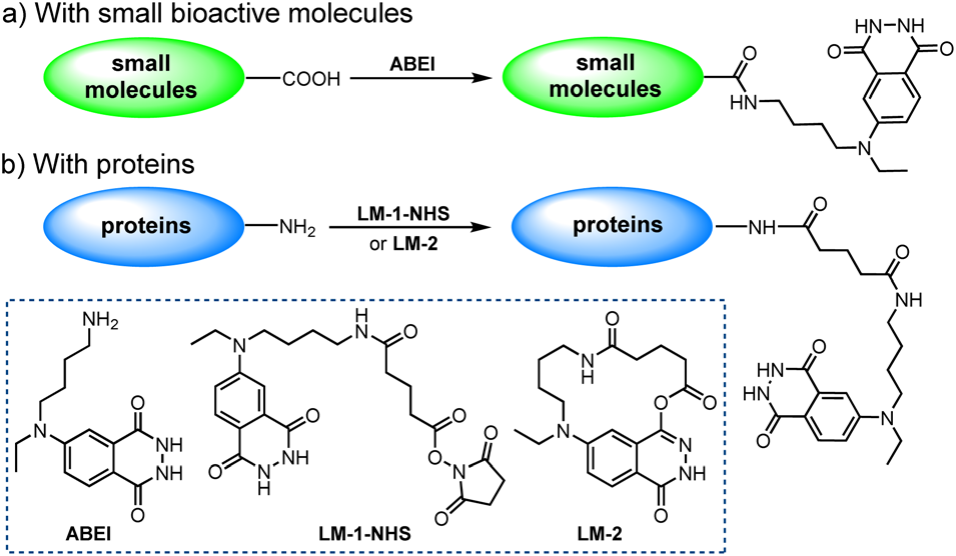
Bioconjugation of isoluminol derivatives.

Among the numerous isoluminol derivatives, *N*-(4-aminobutyl)-*N*-ethylisoluminol (ABEI) is prominent owing to its relatively higher chemiluminescence (CL) efficiency as well as the appropriate length of alkyl-bridging group (Scheme 1).^10^ With its terminal amino group, ABEI can be easily labelled to small bioactive molecules such as thyroxine,^9^ cortisol^11^ and multiple steroid derivatives.^12^ These molecules or their corresponding derivatives generally contain a carboxylic group that can be conveniently transformed into *N*-hydroxysuccinimide (NHS) active esters. The NHS active esters will react with the terminal amino group of ABEI quickly (Scheme 1a).

When conjugating ABEI to proteins, given the abundance of amino groups on proteins, using variants of ABEI represents a more flexible approach. To date, a dichotomy of variants has been identified^13^ (Scheme 1b): (1) LM-1-NHS; (2) LM-2. The former approach hinges on the high reactivity of the NHS active esters of ABEI-glutarate (or -succinate). Nevertheless, no pure NHS products have been isolated thus far, likely owing to their vulnerability to hydrolysis.^14^ Additionally, an overly long structure proves detrimental to high-efficiency conjugation. The latter macrocyclic lactone^15^ exhibits good reactivity towards the amino group and greater stability against hydrolysis. However, its specific cyclic structure impedes further modification of this compound.

Apparently, although ABEI is commercially available, the process of labelling ABEI onto biomacromolecules such as proteins is not as straightforward as it seems. This is because the conversion to ABEI-glutarate (or -succinate) is an inevitable step. Moreover, long-chain NHS esters pose the risk of self-cyclization, especially in solution. Therefore, we envisioned that a labelling compound internally equipped with a terminal carboxyl group might be more straightforward for the purpose. Herein, we report the novel synthesis of isoluminol derivatives bearing a terminal carboxyl group for protein labelling.

## Results and discussion

**Scheme 2:**
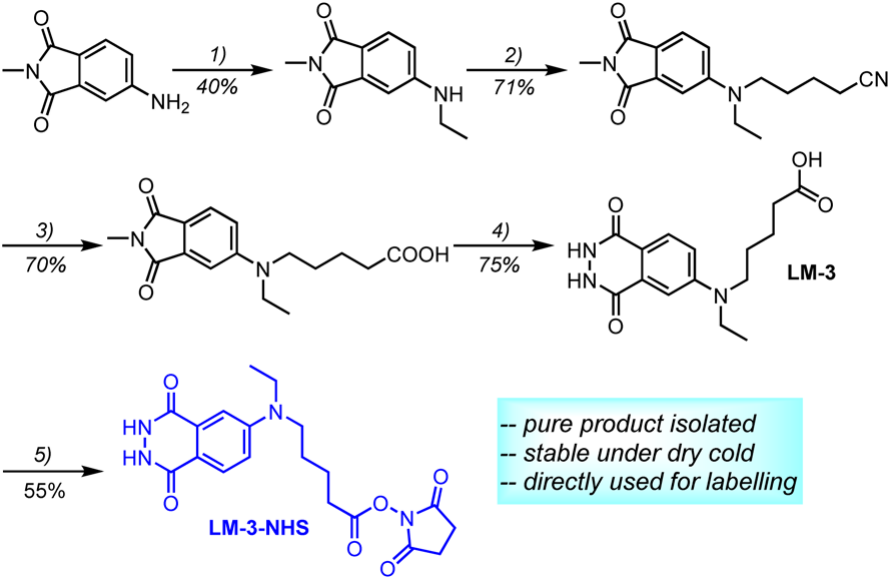
Synthesis of LM-3-NHS. (1) Diethyl sulfate, 110 °C, 24 h; (2) 5-bromovaleronitrile, DMF, 120 °C, 24 h; (3) H_2_SO_4_, H_2_O, AcOH, 70 °C, 16 h; (4) N_2_H_4_·H_2_O, EtOH, 50 °C, 24 h; (5) *N*-hydroxysuccinimide, EDC, DMSO, 40 °C, 12 h.

We successfully synthesized the first isoluminol derivative, LM-3, through the introduction of a carboxyl group. The synthesis process utilized 5-bromovaleronitrile as the precursor (Scheme 2). Since phthalhydrazide compounds barely tolerate the drastic acidic conditions, acidolysis of the cyano-group is carried out prior to hydrazinolysis. The synthetic route is brief and suitable for large-scale production, with an overall acceptable yield. The successful synthesis of the carboxyl-functionalized luminol LM-3 inspired us to synthesize its corresponding NHS active ester immediately. Carboxyl group activation was nearly quantitative. What was more gratifying was that a 97.5% (by HPLC, details see ESI†) pure active ester LM-3-NHS could be isolated under weakly acidic conditions with a moderate yield.

**Fig. 1:**
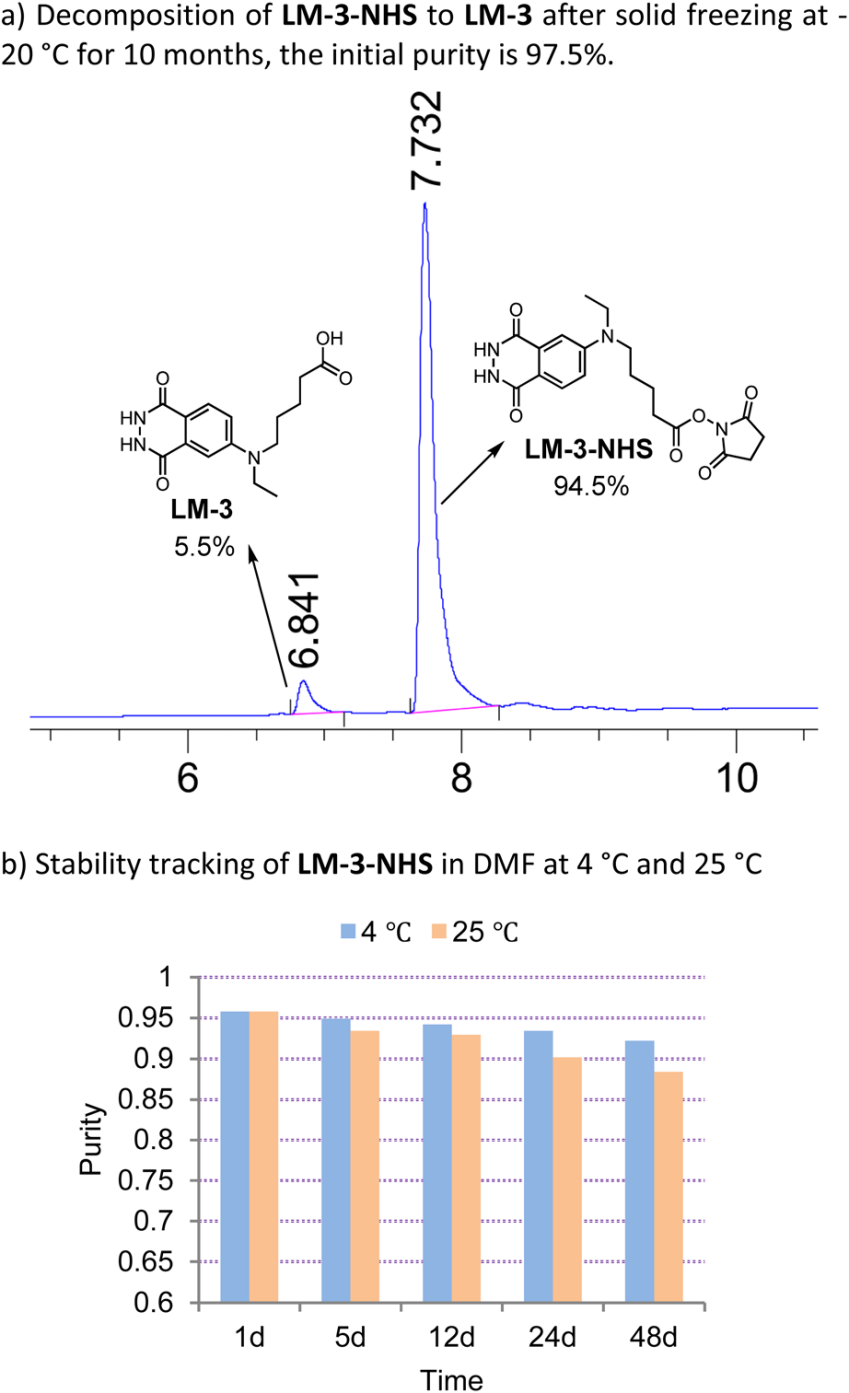
Stability of LM-3-NHS. (a) Decomposition of LM-3-NHS to LM-3 after solid freezing at −20 °C for 10 months, the initial purity is 97.5%. (b) Stability tracking of LM-3-NHS in DMF at 4 °C and 25 °C.

The storage stability was then strictly assessed. We found that the purity of LM-3-NHS decreased by around 3% after freezing at −20 °C for 10 months (Fig. 1a, full spectrograms see ESI†). Before conjugation with biomacromolecules, labelling compounds are normally dissolved in anhydrous dimethyl formamide to a particular concentration. The stability of LM-3-NHS in dimethyl formamide is also crucial to the labelling efficacy. We monitored the decrease in purity of LM-3-NHS in dimethyl formamide at both 4 °C and 25 °C for as long as 48 days (Fig. 1b). At 4 °C, the decrease in purity was only 3.6% after 48 days. Even at 25 °C for the same time, the residual purity was still close to 90%. Therefore, we believed that LM-3-NHS was stable enough for protein labelling.

**Fig. 2:**
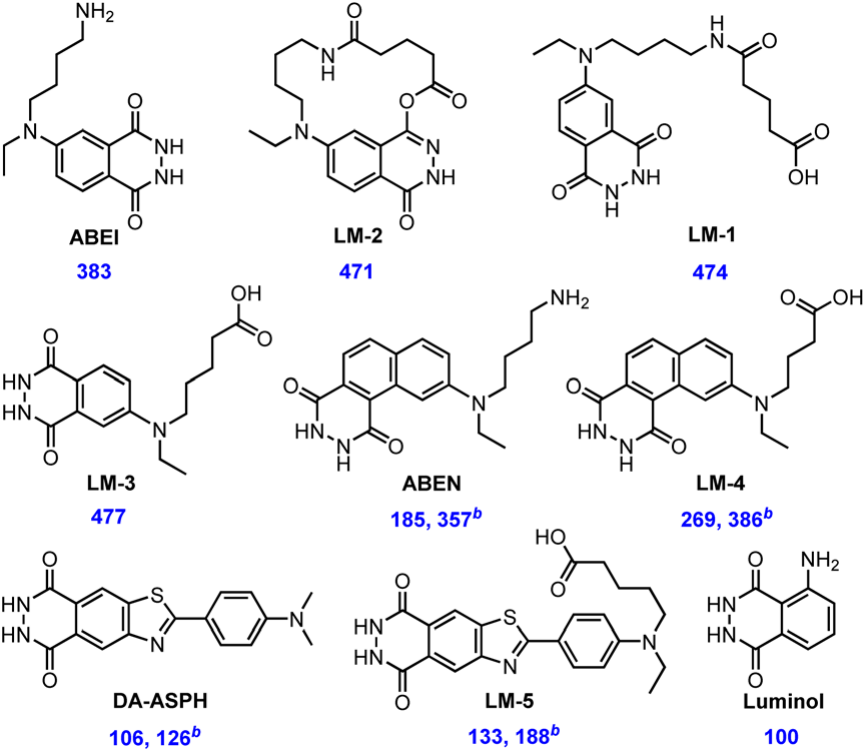
Relative CL intensity of isoluminol (or luminol) derivatives.^*a a*^Follow general procedure with the standard oxidation condition. ^*b*^Follow general procedure with the diluted oxidation condition.

Apart from aminophthalhydrazides, 7-*N*-(4-aminobutyl-*N*-ethyl)naphthalene-1,2-dicarboxylic acid hydrazide (ABEN) with a terminal amino group was also proved to have high chemiluminescence efficiency.^16^ Following the same tactic as LM-3, the aminonaphthalhydrazide LM-4 equipped with a terminal carboxyl group was designed and synthesized (Fig. 2, synthesis see ESI†). Novel luminol-related compounds containing a 2-arylbenzothiazole moiety were found to produce intense chemiluminescence in 2000.^17^ And DA-ASPH was screened as the best among them but with no conjugation anchor. Following the reported method, by reacting 4-(methylamino)benzaldehyde with 5-bromovaleronitrile, we ultimately synthesized compound LM-5 with an internal terminal carboxyl group (Fig. 2, synthesis see ESI†).

The mechanism of luminol oxidation, especially in aqueous solution, has not yet been fully elucidated;^18^ therefore, a prediction of chemiluminescence efficiency after structure modification is insignificant. The chemiluminescence efficiency of several isoluminol (or luminol) derivatives, including three novel carboxylic compounds, was evaluated and compared using the standard oxidation system of MAGLUMI® X3 from Snibe Diagnostic (details see ESI†). As shown in Fig. 2, the chemiluminescence efficiency of ABEI was found to have an increase of about 4-fold compared to the well-known reference compound luminol. Variation of ABEI to LM-1 and LM-2 slightly increased chemiluminescence efficiency. The encouraging result for us was that LM-3 showed similar excellent chemiluminescence performance to LM-2, which basically confirmed the potential for protein labelling. The low performance of ABEN and DA-ASPH with the standard oxidation system was improved mildly by diluting the existing system. For LM-4 and LM-5, their introduced carboxyl groups have little influence on the chemiluminescence efficiency.

The comparison of the labelling efficacy between LM-3-NHS and LM-2 was then verified by conjugating them to bovine serum albumin (BSA). BSA conjugates were analyzed by MALDI-TOF mass spectrometry to measure the incorporation (details see ESI†). As can be noted from Table 1, the labelling efficacy of LM-3-NHS is superior to that of LM-2, and we speculated that the high reactivity of the NHS active ester accelerated the conjugation process.

**Table 1:** The measurement of (i) labeling efficacy by mass spectroscopy and (ii) BSA conjugates relative CL intensity

Conjugate	Equivalents of labels^a^	(i) No. of labels	(ii) Relative CL intensity
LM-2-BSA	12	5–6^b^	171, 125^c^
LM-3-BSA	12	8–9^b^	334, 260^c^

a Molar ratio to BSA.

b Analyzed by MALDI-TOF mass spectrometry.

c Relative CL intensity after samples kept at 4 °C for 10 days.

The chemiluminescence efficiency of BSA conjugates was also measured. The relative CL intensity of LM-3-BSA was obviously higher than that of LM-2-BSA, which was consistent with LM-3-BSA's better labelling efficacy. We further traced the chemiluminescent stability of these two BSA conjugates. The relative CL intensity of LM-2-BSA dropped 27% after 10 days of storage at 4 °C. For LM-3-BSA, the decline was 22%, which proved LM-3-BSA's better stability.

Acridinium dimethylphenyl esters represent another category of highly efficient labels employed in automated immunoassays. For these labels, the *N*-sulfopropyl group typically serves as the hydrophilic functional group. It enhances the water solubility of the labels and mitigates the hydrophobic nature of the acridinium ring.^19^ This strategy proves to be highly effective. Enhanced water solubility can significantly boost the performance in more biocompatible environments. Specifically, it plays a crucial role in reducing the non-specific binding to magnetic microparticles. This, in turn, can enhance the detection sensitivity of clinical analysis.^20^ Similarly, increasing the water solubility of isoluminol (or luminol) derivatives holds the potential for achieving higher chemiluminescence quantum yields or providing a better user experience in aqueous buffer systems. However, such instances are relatively scarce.^21^

**Fig. 3:**
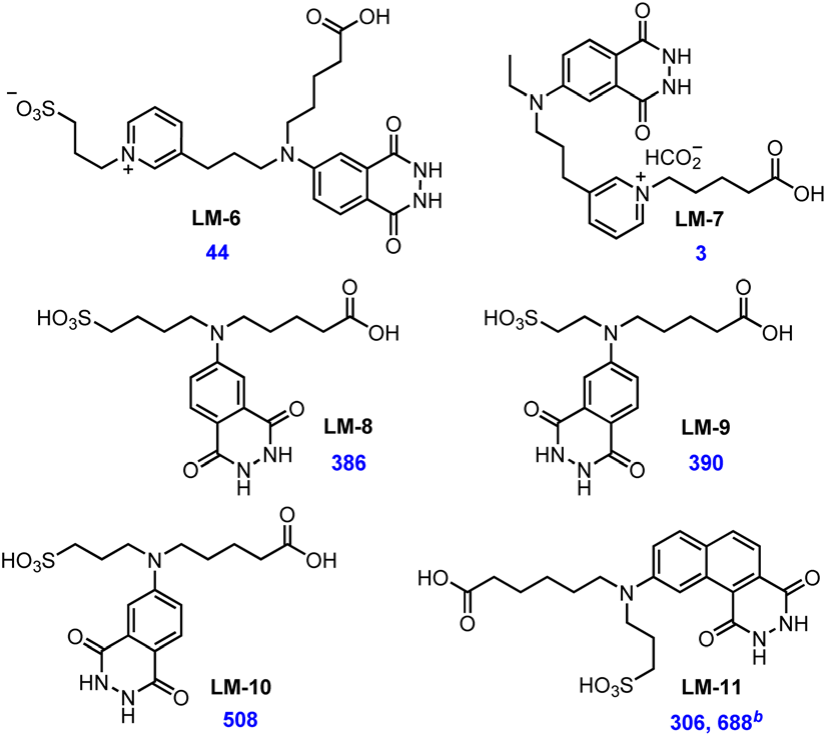
Relative CL intensity of isoluminol derivatives with hydrophilic groups.^*a a*^Follow general procedure with the standard oxidation condition. ^*b*^Follow general procedure with the diluted oxidation condition.

Apart from the better labelling efficacy, there is more convenience and flexibility of LM-3-NHS on further modification compared to the cyclic LM-2. Based on the carboxyl group, we decided to make a further attempt to improve the aqueous solubility of labelling compounds by introducing a hydrophilic group (Fig. 3). The introduction of pyridinium could remarkably improve the solubility, while it had a significant negative impact on the chemiluminescence efficiency.

When a pyridinium propyl sulphobetaine was introduced, the relative CL intensity of LM-6 (synthesis see ESI†) decreased to only 44. Even worse, after the insertion of pyridine into the carboxyl chain, labelling compound LM-7 (synthesis see ESI†) barely released photons at the same test concentration. Though concrete details were still vague, we roughly realized that pyridinium-based hydrophilic groups did not match the chemiluminescence system of isoluminol.

After quick adjustment, linear alkyl sulfonic acids were selected as the hydrophilic group. Following the same synthetic strategy, three novel isoluminol labelling compounds LM-8, LM-9 and LM-10 were synthesized (synthesis see ESI†), all of which showed remarkable water solubility. The length of the alkyl chain was found crucial to chemiluminescence efficiency. The isoluminol labelling compounds containing ethyl sulfonic acid and butyl sulfonic acid both showed a restraining effect (compared to LM-3). On the contrary, LM-10 with the propyl sulfonic group slightly increased the relative CL intensity to 508, which was undoubtedly encouraging. We also applied the propyl sulfonic group to the aminonaphthalhydrazide system. LM-11 (synthesis see ESI†) was synthesized after fine-tuning the carboxyl chain length. The relative CL intensity of this sulfonic aminonaphthalhydrazide similarly increased in the standard oxidation system compared to LM-4, and an even more distinct rising trend was observed under the diluted oxidation system.

As discussed above, lowering the non-specific binding of magnetic microparticles would improve the detection sensitivity of clinical analysis, and isoluminol derivatives as sensitive labels have significant relevance to this binding. Non-specific binding of five isoluminol derivatives to two particles with different surface compositions and charge was measured and compared. Typically, lower fNSB reflects greater resistance to non-specific binding as well as better efficacy of the wash step.

**Fig. 4:**
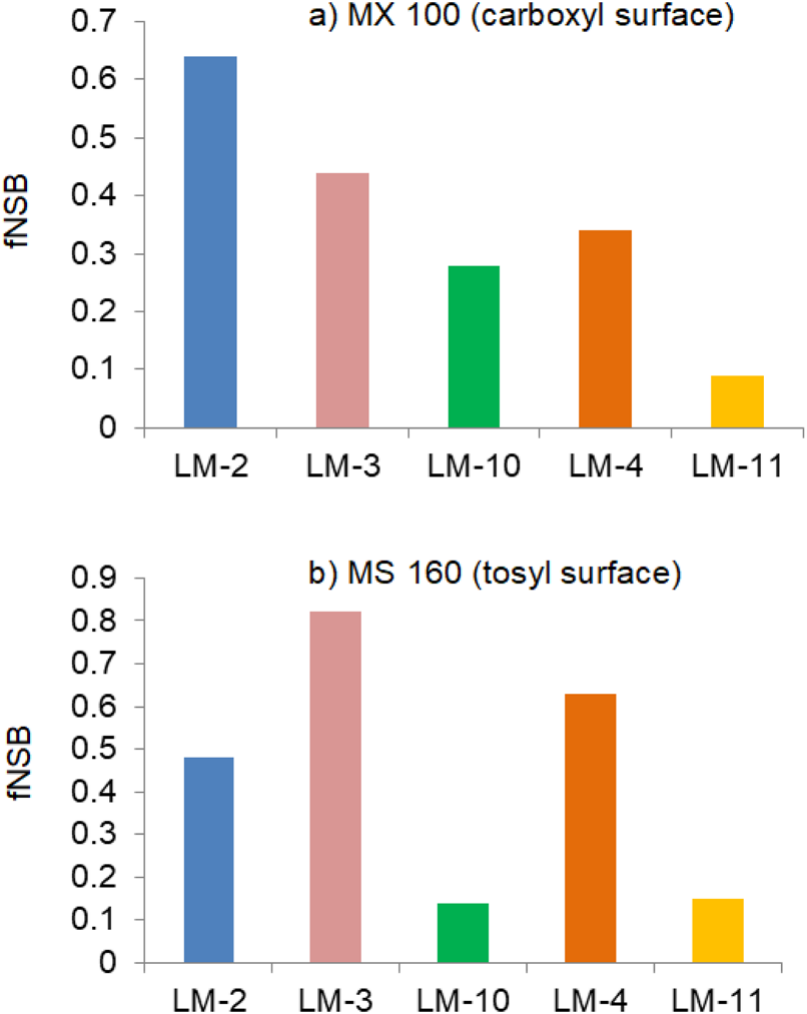
The measurement of non-specific binding. ^*a a*^The measurement of non-specific binding entails measuring residual chemiluminescence associated with the magnetic microparticles after a wash step (details see ESI†). Residual chemiluminescence, when divided by the total chemiluminescence input, gives the fractional non-specific binding (fNSB).

Two commercially-available magnetic microparticles from JSR Life Sciences were selected for this assessment. The MX 100 (carboxyl surface) is hydrophobic while MS 160 (tosyl surface) is hydrophilic. As shown in Fig. 4, the comparison results of LM-2 and LM-3 varied with particle surfaces. LM-3 showed a mild advantage with MX 100 (carboxyl surface). When LM-10 was compared to LM-2 and LM-3, a lower fNSB was observed on both hydrophilic and hydrophobic surfaces, and it was particularly prominent with MS 160 (tosyl surface). Likewise, LM-11 was compared to LM-4, and we found obviously lower fNSB on the two selected particles. Therefore, as a whole, the introduction of the propyl sulfonic group reduced the non-specific binding of labelling compounds to magnetic microparticles with different surfaces. We speculated that the lower fractional non-specific binding of LM-10 and LM-11 was attributed to the hydrophilic propyl sulfonic group, which improved the washing efficiency.

## Conclusions

In conclusion, we have synthesized a series of isoluminol (or luminol) derivatives with a terminal carboxyl group. The propyl sulfonic group as a hydrophilic functional group could increase both water solubility and chemiluminescence quantum yields. Non-specific binding of magnetic microparticles was studied, and for labelling reagents, better water solubility could decrease the non-specific binding notably. An *N*-hydroxysuccinimide active ester of carboxylic acid (LM-3-NHS) as a direct protein labelling reagent was isolated successfully with high purity, which was found to be stable, possess good chemiluminescence efficiency and high labelling efficacy with BSA. It is certain that LM-3-NHS is a convenient and practical direct protein labelling reagent, and further study on chemiluminescence enhancement is currently being explored by our team.

## Data availability

All relevant data are within the manuscript and its additional files.

## Conflicts of interest

There are no conflicts to declare.

## Supplementary Material

RA-015-D5RA00677E-s001
