# Supplementary material for: Synthesis of isoluminol derivatives with a terminal carboxyl group for protein labelling
Source: RSC Adv. 2025 May 28;15(22):17776–80. doi: 10.1039/d5ra00677e (PMC12117363; doi:10.1039/d5ra00677e)
Supplement: RA-015-D5RA00677E-s001 [file RA-015-D5RA00677E-s001.pdf]

Supplementary Information (SI) for RSC Advances.
This journal is © The Royal Society of Chemistry 2025

Synthesis of Isoluminol Derivatives with a Terminal Carboxyl Group for Protein Labelling

Qiao Ma,[a‡] Lei Liu,[a‡] Chunfeng Luo,[a] Yue Wu,[a] Fengshu Qin,[a] Kai Du[a]*

‡ Equal contribution

[a] Shenzhen New Industries Biomedical Engineering Co., Ltd., Reagent Key Raw Materials R&D and Production Center, Pingshan District, Shenzhen, Guangdong 518122, P. R. China; Email: kai.du@snibe.cn

Table of Contents.

General Experimental……………………………………………………………………………………S1
Materials…………………………………………………………………………………………………S1
Instrumentation…………………………………………………………………………………………S1
Synthesis and Characterization…………………………………………………………………………S2
Chemiluminescence Measurements……………………………………………………………………S17
Labelling Efficacy Measurements………………………………………………………………………S20
Fractional Non-Specific Binding Measurements………………………………………………………S22
References………………………………………………………………………………………………S22
Spectra……………………………………………………………………………………………………S23

General Experimental. Unless otherwise noted, all reactions were performed in oven-dried glassware. Reactions that require heating were carried out in the oil bath. Analytical thin layer chromatography (TLC) was performed with EM Science silica gel 60 F254 aluminum plates. Visualization was done under a UV lamp (254 nm) and by immersion in ethanolic phosphomolybdic acid (PMA) or potassium permanganate ($KMnO_4$), followed by heating using a heat gun. Purification of reaction products were generally done through reversed-phase C18 column (CH_3CN/H_2O, 0.1% TFA) or flash column chromatography with Grace Materials Technologies 230-400 mesh silica gel. Organic solutions were concentrated by rotary evaporation at 23–55 °C. Samples were dried though vacuum freeze-drying or rotary evaporation.

Materials. Reagents and solvents used for organic synthesis, HPLC solvents and BSA (bovine serum albumin) were purchased from Sigma-Aldrich, Acros, J&K Scientific and Aladdin, which were used without any further purification unless specified. Magnetic microparticles MX 100 and MS 160 were purchased from JSR Life Sciences. The oxidation reagents (A and B) were homegrown from Snibe (Shenzhen New Industries Biomedical Engineering Co., Ltd.) Diagnostic.

Instrumentation. Proton nuclear magnetic resonance spectra (^{1}H NMR) and carbon nuclear magnetic resonance spectra (^{13}C NMR) were recorded at 23 °C on Bruker 400 MHz spectrometer in $CDCl_3$, DMSO-d_6 and Methanol-d_4. Chemical shifts of ^{1}H NMR spectra were reported as parts per million in δ scale using residual solvent signal ($CDCl_3$: 7.26 ppm, DMSO-d_6: 2.50 ppm, Methanol-d_4: 3.31ppm) or tetramethylsilane (0.00 ppm) as internal standard. Chemical shifts of ^{13}C NMR spectra were reported using residual solvent signal of $CDCl_3$ (77.16 ppm), DMSO-d_6 (39.52 ppm) or Methanol-d_4 (49.00 ppm) on the δ scale. Data are represented as follows: chemical shift (δ ppm), multiplicity (s = singlet, d = doublet, t = triplet, q = quartet, m = multiplet, br = broad), coupling constant (J, Hz) and integration. LC-MS analysis results were obtained on Agilent 1260 (HPLC) and Agilent 6120 (MS). High resolution mass spectra (HRMS) were obtained on a Waters Xevo G2-XS QTof. Preparative reverse phase liquid chromatography was Buchi C-815 which was equipped with C18 columns from Santai Technologies. Chemiluminescence measurements were collected on the MAGLUMI® X3 chemiluminescence immunoassay (CLIA) system, which was a homegrown instrument from Snibe (Shenzhen New Industries Biomedical Engineering Co., Ltd.) Diagnostic.

Synthesis and Characterization

1. Synthesis of LM-1.

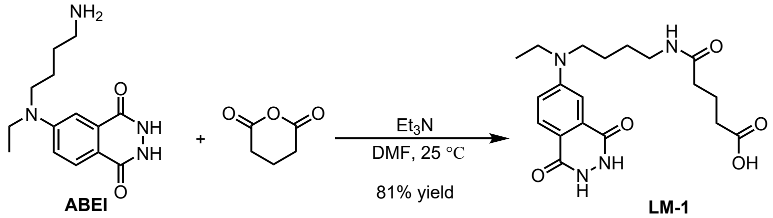

To a flask was added **ABEI** (276 mg, 1 mmol), glutaric anhydride (228 mg, 2 mmol), triethylamine (404 mg, 4 mmol) and DMF (3 mL), resulting mixture was kept stirring at 25 °C for 6 h. After that, crude mixture was used for purification with a reversed-phase C18 column (CH_3CN/H_2O, 0.1% TFA) to offer pure **LM-1** (316 mg, 81% yield), which was dried by vacuum freeze-drying. **^{1}H NMR** (400 MHz, DMSO-d_6) δ 11.44 (s, 3H), 7.83 (d, J = 8.6 Hz, 2H), 7.27 – 6.93 (m, 2H), 3.52 – 3.42 (m, 2H), 3.17 – 2.99 (m, 2H), 2.19 (t, J = 6.6 Hz, 2H), 2.08 (t, J = 6.4 Hz, 2H), 1.78 – 1.62 (m, 2H), 1.49 (d, J = 40.5 Hz, 4H), 1.12 (s, 3H). **^{13}C NMR** (101 MHz, DMSO-d_6) δ 174.2, 171.5, 155.5, 154.3, 150.3, 128.8, 127.1, 116.6, 115.2, 103.3, 49.3, 44.5, 38.1, 34.5, 33.1, 26.7, 24.2, 20.8, 11.9. **HRMS** m/z (ESI): calcd. for $C_{19}H_{27}N_4O_5$ $[M+H]^+$: 391.1981; found: 391.1988.

2. LM-3-NHS.

1) Synthesis of LM-3-NHS.

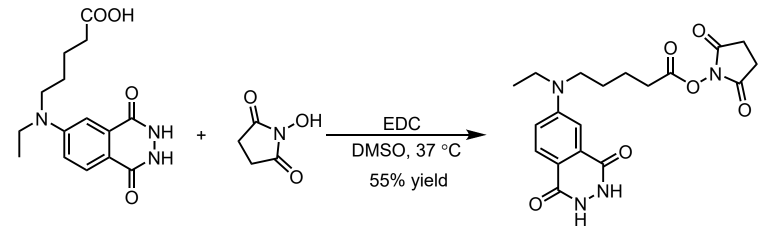

LM-3 (100 mg, 0.328 mmol) was dissolved with DMSO (5 mL), followed by adding 1-(3-dimethylaminopropyl)-3-ethylcarbodiimide hydrochloride (EDC) (125.6 mg, 0.655 mmol), resulting mixture was kept stirring at room temperature for 20 min under argon protection. To this mixture was then added *N*-hydroxysuccinimide (75.4 mg, 0.655 mmol), the reaction was stirred at 40 °C for 12 h. After that, crude mixture was used for purification with a reversed-phase C18 column (CH_3CN/H_2O, 0.1% TFA) to offer pure **LM-3-NHS** (73 mg, 55% yield) which was dried by vacuum freeze-drying. **^{1}H NMR** (400 MHz, DMSO-d_6) δ 11.12 (s, 2H), 7.83 (d, J = 9.0 Hz, 1H), 7.19 (dd, J = 9.1, 2.6 Hz, 1H), 7.03 (s, 1H), 3.46 (dt, J = 14.5, 7.0 Hz, 4H), 2.81 (s, 4H), 2.76 (t, J = 6.6 Hz, 2H), 1.77 – 1.60 (m, 4H), 1.13 (t, J = 7.0 Hz, 3H). **^{13}C NMR** (101 MHz, DMSO-d_6) δ 172.8, 170.2, 169.0, 155.4, 154.3, 150.3, 128.8, 127.1, 116.7, 115.3, 103.4, 49.1, 44.5, 30.0, 25.9, 25.5, 25.3, 21.8, 11.8. **HRMS** m/z (ESI): calcd. for $C_{19}H_{23}N_4O_6$ $[M+H]^+$: 403.1618; found: 403.1668.

2) Stability of LM-3-NHS.

The solid sample of **LM-3-NHS** was stored frozen at -20 °C for ten months, and then its purity was detected by means of HPLC (High Performance Liquid Chromatography) and ^{1}H-NMR (Nuclear Magnetic Resonance Spectroscopy). The information is as follows:

(1) HPLC Detection：C18 column (150 mm × 4.6 mm, 5 μm); acetonitrile-water; 25 °C; 2mL/min.

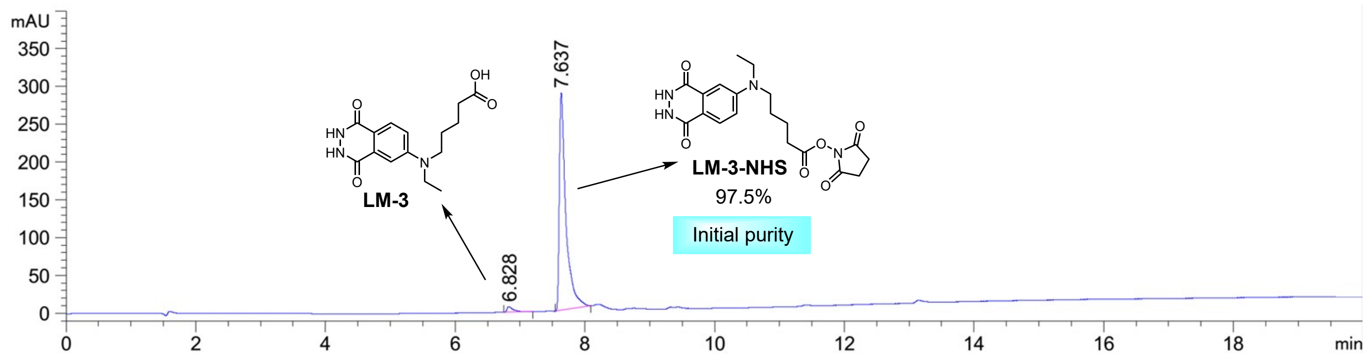

Peak #	RetTime [min]	Type	Width [min]	Area [mAU*s]	Height [mAU]	Area %
1	6.828	BB	0.0984	51.66996	7.31730	2.4789
2	7.637	BB	0.1026	2032.72644	287.06778	97.5211

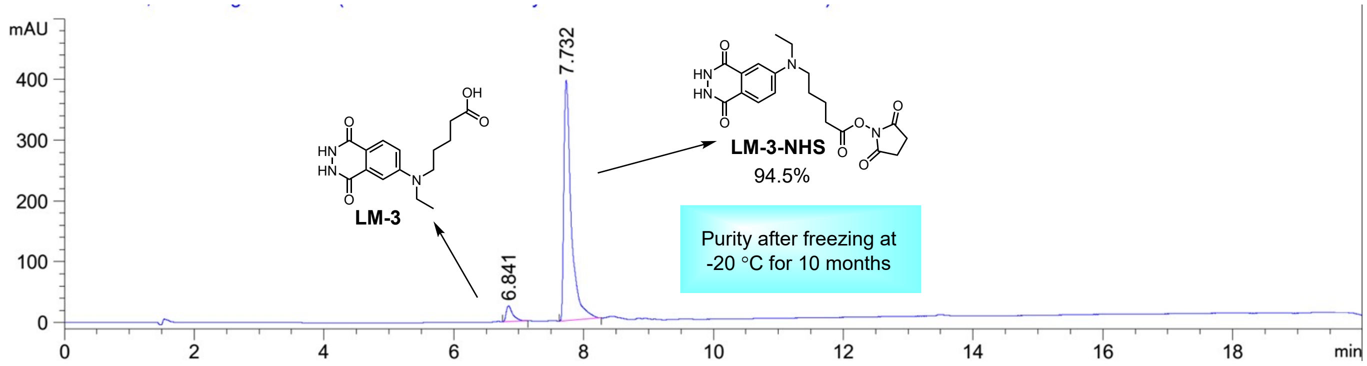

Peak #	RetTime [min]	Type	Width [min]	Area [mAU*s]	Height [mAU]	Area %
1	6.841	BB	0.1054	181.69264	25.41640	5.5160
2	7.732	BB	0.1164	3112.22876	395.27722	94.4840

(2) ^{1}H-NMR Detection.

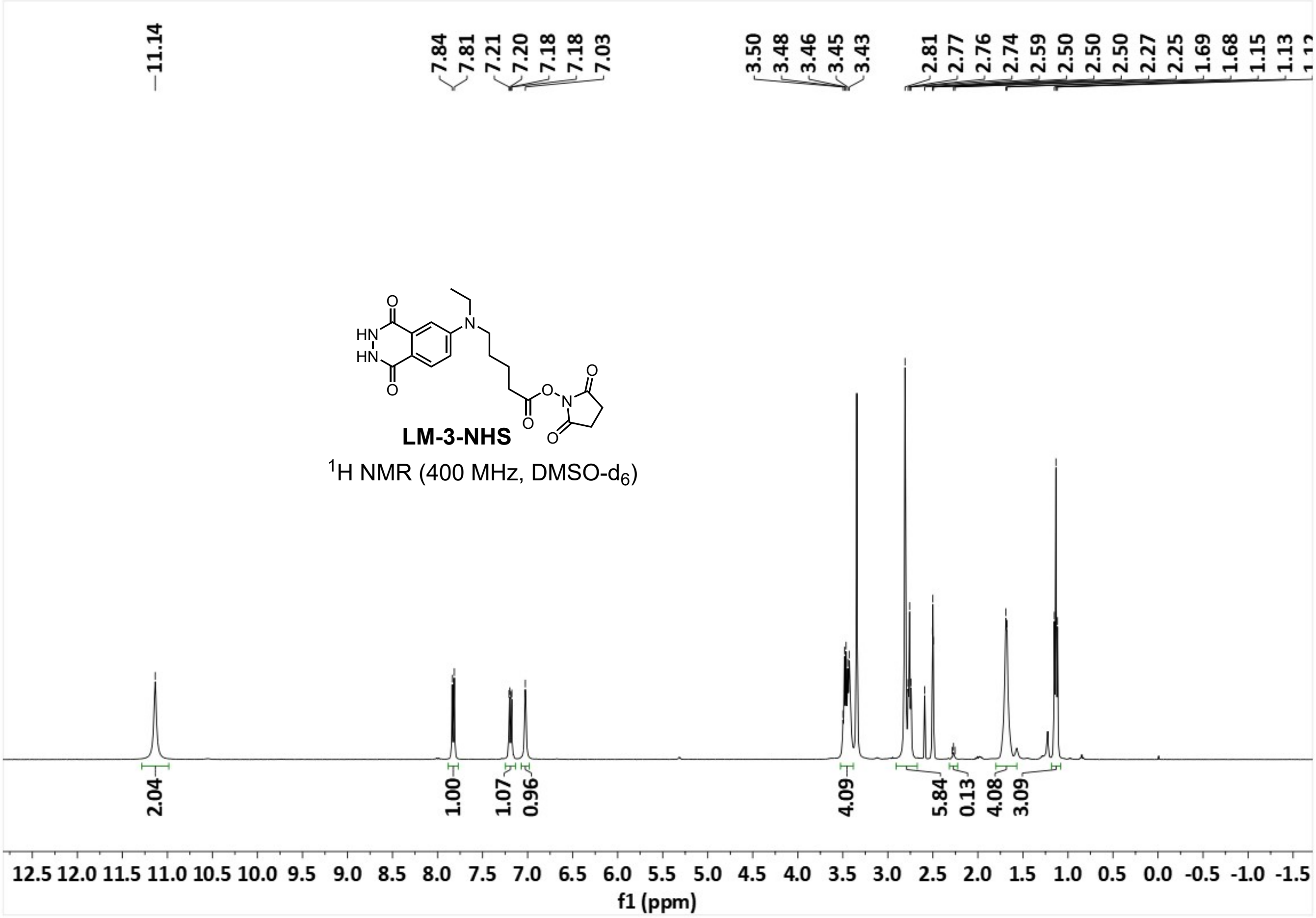

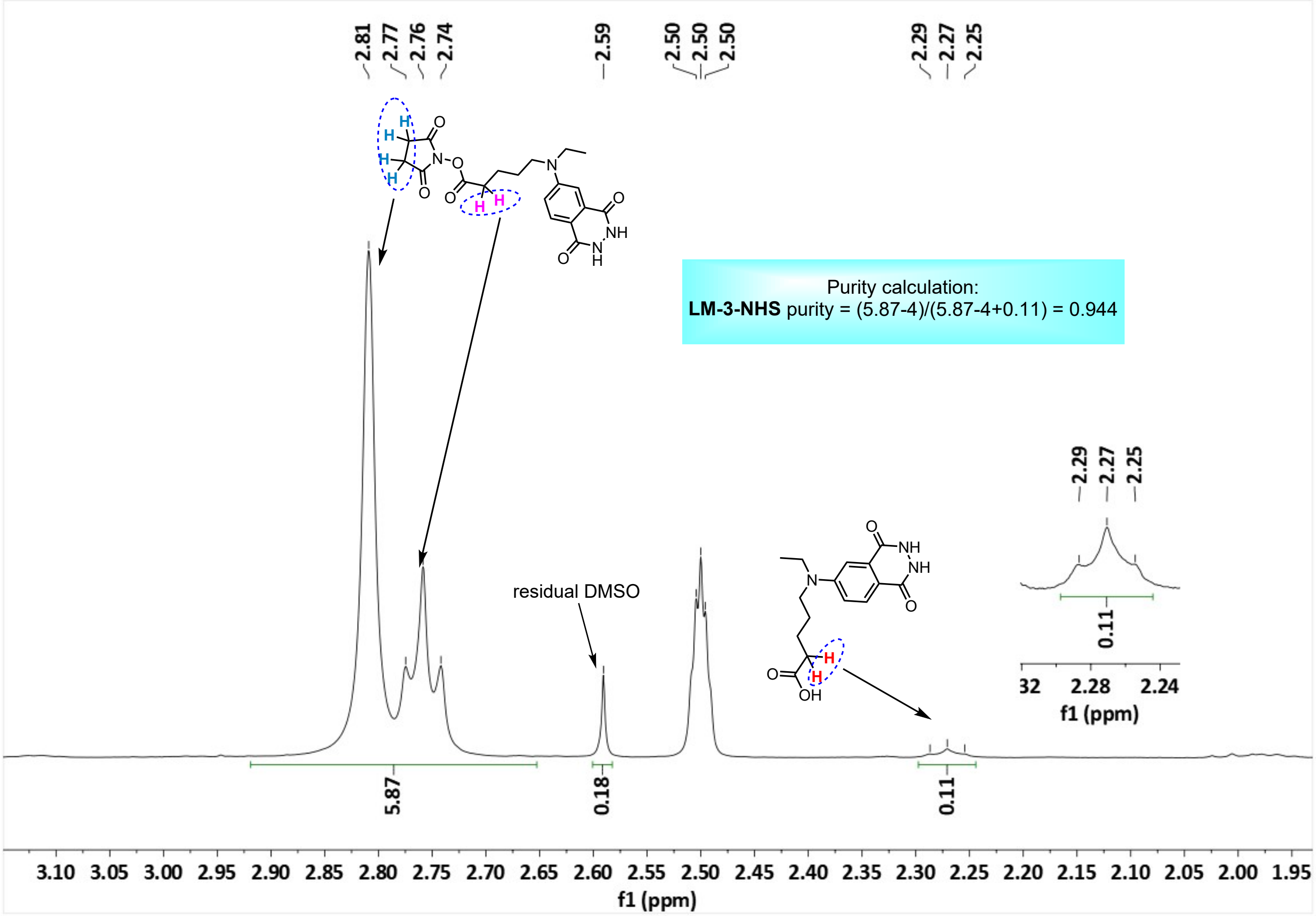

3. Synthesis of LM-3.

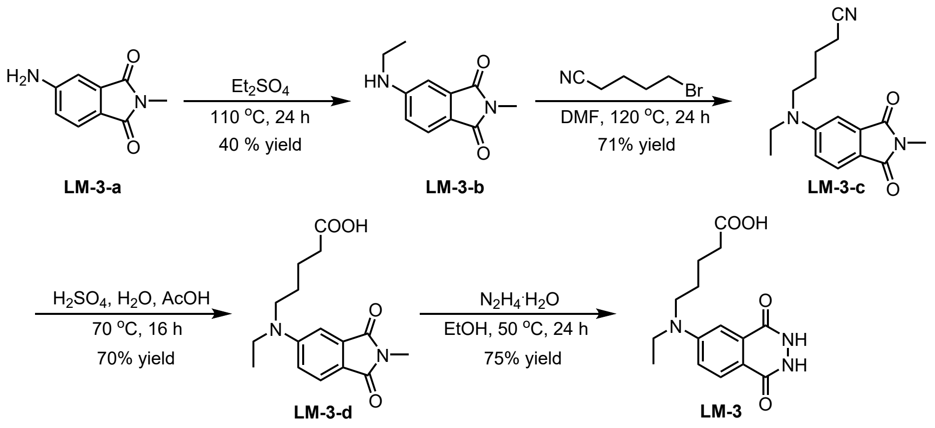

1) Preparation of LM-3-b.

To a three-necked flask was added **LM-3-a** (5.0 g, 28.4 mmol) and diethyl sulfate (50 mL, 379 mmol), resulting mixture was heated at 110 °C under argon protection for 24h. The reaction was monitored by TLC and terminated when starting material fully converted. After that, the mixture was cooled to room temperature before adding H_2O (50 mL), kept stirring for 5 min then extracted with DMC (200 mL). The collected organic phase was dried, solvents were removed to form a crude sample of **LM-3-b**, which was further purified by silica-gel column chromatography to offer the pure **LM-3-b** (2.32 g, 40 % yield). **^{1}H NMR** (400 MHz, DMSO-d_6) δ 7.50 (d, J = 8.3 Hz, 1H), 6.95 (t, J = 5.2 Hz, 1H), 6.89 (d, J = 2.1 Hz, 1H), 6.76 (dd, J = 8.3, 2.1 Hz, 1H), 3.16 (qd, J = 7.1, 5.1 Hz, 2H), 2.95 (s, 3H), 1.18 (t, J = 7.2 Hz, 3H). **^{13}C NMR** (101 MHz, DMSO-d_6) δ 168.5, 168.1, 154.0, 134.6, 124.7, 116.6, 114.6, 105.2, 37.2, 23.4, 14.0. MS m/z (ESI by Agilent 6120): calcd. for $C_{11}H_{13}N_2O_2$ $[M+H]^+$: 205.1; found: 205.5.

2) Preparation of LM-3-c.

LM-3-b (2.32 g, 11.4 mmol) was dissolved with DMF (50 mL), followed by adding 5-bromovaleronitrile (3.7 g, 22.8 mmol), the reaction was heated at 120 °C under argon protection for 24h. After that, water (200 mL) was added to this system and extracted with ethyl acetate (400 mL), a crude sample of **LM-3-b** was got after removing solvents, this crude sample was further purified by silica-gel column chromatography to offer the pure **LM-3-c** (2.3g, 71% yield). **^{1}H NMR** (400 MHz, DMSO-d_6) δ 7.55 (d, J = 8.5 Hz, 1H), 6.98 (d, J = 2.3 Hz, 1H), 6.89 (dd, J = 8.6, 2.4 Hz, 1H), 3.53 – 3.39 (m, 4H), 2.96 (s, 3H), 2.59 – 2.53 (m, 2H), 1.63 (p, J = 3.6 Hz, 4H), 1.12 (t, J = 7.0 Hz, 3H). **^{13}C NMR** (101 MHz, DMSO-d_6) δ 168.5, 168.0, 152.0, 134.8, 124.7, 120.6, 116.2, 114.3, 104.8, 49.0, 44.8, 26.0, 23.5, 22.2, 16.0, 11.9. MS m/z (ESI by Agilent 6120): calcd. for $C_{16}H_{20}N_3O_2$ $[M+H]^+$: 286.2; found: 286.4.

3) Preparation of LM-3-d.

To a flask was added concentrated sulfuric acid (2 mL), purified water (2 mL), acetic acid (2 mL) and well mixed, followed by the addition of **LM-5-c** (2.2 g, 7.71 mmol), the resulting mixture was heated at 70 °C for 16 h. After that, the reaction was cooled to room temperature before adding H_2O（10 mL）, followed by the addition of solid sodium bicarbonate until no gas was released. DMF (10 mL) was added to get a clean solution, which was directly used for purification with a reversed-phase C18 column (CH_3CN/H_2O, 0.1% TFA) to offer the pure **LM-3-d** (1.64 g, 70% yield). **^{1}H NMR** (400 MHz, DMSO-d_6) δ 12.04 (s, 1H), 7.55 (d, J = 8.6 Hz, 1H), 6.96 (d, J = 2.4 Hz, 1H), 6.87 (dd, J = 8.6, 2.4 Hz, 1H), 3.47 (q, J = 7.0 Hz, 2H), 3.43 – 3.38 (m, 2H), 2.96 (s, 3H), 2.31 – 2.21 (m, 2H), 1.63 – 1.48 (m, 4H), 1.11 (t, J = 7.0 Hz, 3H). **^{13}C NMR** (101 MHz, DMSO-d_6) δ 174.4, 168.5, 168.0, 152.1, 134.8, 124.7, 116.0, 114.3, 104.8, 49.6, 44.8, 33.4, 26.3, 23.5, 21.9, 11.9. **HRMS** m/z (ESI): calcd. for $C_{16}H_{20}N_2O_4Na$ $[M+Na]^+$: 327.1321; found: 327.1341.

4) Preparation of LM-3.

LM-3-d (5.5 g, 18.1 mmol) and hydrazine hydrate (18.12g, 362 mmol) were dissolved with ethanol (55 mL), resulting mixture was refluxed for 24 h. The reaction was monitored by LCMS and terminated when starting material fully converted. After that, ethanol and residual hydrazine hydrate were removed with rotary evaporator, a crude sample of **LM-3** was obtained after adjusting the pH to 2-3 with 1 N HCl. DMF (100 mL) was used to dissolved the crude sample to get a clean solution which was directly used for purification with a reversed-phase C18 column (CH_3CN/H_2O, 0.1% TFA) to offer the pure **LM-**

3 (4.1 g, 75% yield). **^{1}H NMR** (400 MHz, Methanol-d_4) δ 8.00 (d, J = 8.8 Hz, 1H), 7.23 (d, J = 9.1 Hz, 2H), 3.55 (q, J = 7.0 Hz, 2H), 3.49 (d, J = 6.6 Hz, 2H), 2.43 – 2.31 (m, 2H), 1.76 – 1.63 (m, 4H), 1.22 (t, J = 7.0 Hz, 3H). **^{13}C NMR** (101 MHz, DMSO-d_6) δ 174.7, 156.0, 154.7, 150.6, 129.0, 127.3, 116.8, 115.4, 103.6, 49.6, 44.7, 33.7, 26.6, 22.2, 12.0. **HRMS** m/z (ESI): calcd. for $C_{15}H_{20}N_3O_4$ [M+H]$^+$: 306.1454; found: 306.1472.

4. Synthesis of LM-4.

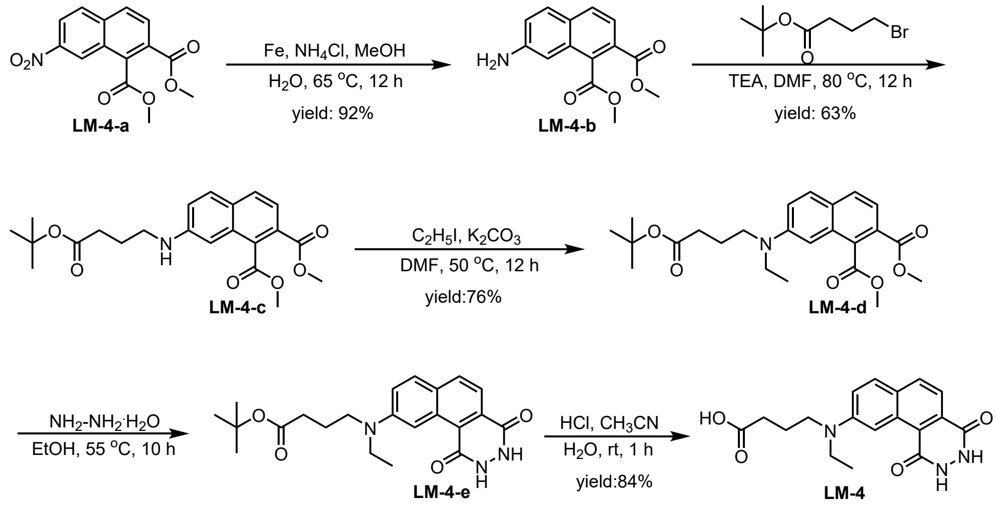

1) Preparation of LM-4-b.

To a flask was added **LM-4-a** (3 g，10.4 mmol), NH_4Cl (2.8 g，52.3 mmol), methanol (90 mL), water (30 mL) and well mixed, followed by the addition of iron powder (5.8 g, 103.6 mmol）under intensely stirring, resulting mixture was then kept heating at 65 °C for 12h under protection of nitrogen. The reaction was monitored by TLC and terminated when starting material fully converted. After that, cooled down the mixture to room temperature before filtration with celite, the solution was then collected which offered a residual after removing methanol. To this residual was added water (20 mL), extracted with DCM and collected the organic phase, a crude sample of **LM-4-b** was got after solvents were removed. This crude sample was further purified with a reversed-phase C18 column (CH_3CN/H_2O, 0.1% TFA) to offer pure **LM-4-b** (2.5 g, 9.6 mmol, 92% yield). **^{1}H NMR** (400 MHz, Chloroform-d) δ 7.78 – 7.70 (m, 2H), 7.67 (d, J = 8.7 Hz, 1H), 7.03 (dd, J = 8.7, 2.2 Hz, 1H), 6.94 (d, J = 2.2 Hz, 1H), 4.04 (s, 3H), 3.93 (s, 3H). **^{13}C NMR** (101 MHz, Chloroform-d) δ 170.2, 166.8, 146.1, 132.3, 131.1, 129.7, 129.5, 129.5, 125.5, 121.4, 120.8, 105.9, 52.9, 52.7. MS m/z (ESI by Agilent 6120): calcd. for $C_{14}H_{14}NO_4$ [M+H]$^+$: 260.1; found: 228.4.

2) Preparation of LM-4-c.

To a pressure bottle was added **LM-4-b** (1 g, 3.9 mmol), DMF (15 mL), 4-bromobutanoic acid *tert*-butyl ester (3.6 mL, 20.3 mmol) and triethylamine (2.8 mL, 20.3 mmol), the bottle was then well sealed. The reaction was kept stirring at 80 °C for 12 h. After that, cooled down the mixture to room temperature, the crude mixture was directly purified with a reversed-phase C18 column (CH_3CN/H_2O, 0.1% TFA) to offer pure **LM-4-c** (1.0 g, 2.5 mmol, 63% yield). **^{1}H NMR** (400 MHz, Chloroform-d) δ 7.69 (q, J = 8.5 Hz, 2H), 7.61 (d, J = 8.9 Hz, 1H), 6.95 (dd, J = 8.8, 2.2 Hz, 1H), 6.68 (d, J = 1.8 Hz, 1H), 4.23 (s, 1H), 4.04 (s, 3H), 3.91 (s, 3H), 3.21 (t, J = 6.8 Hz, 2H), 2.35 (t, J = 7.1 Hz, 2H), 1.94 (p, J = 6.9 Hz, 2H), 1.44 (s, 9H). **^{13}C NMR** (101 MHz, Chloroform-d) δ 172.9, 170.3, 166.9, 147.4, 132.1, 131.4, 129.3, 129.3, 129.1, 125.5, 120.8, 120.3, 101.4, 80.7, 52.8, 52.6, 43.2, 33.3, 28.2, 28.2, 28.2, 24.4. **HRMS** m/z (ESI): calcd. for $C_{22}H_{27}NO_6Na$ [M+Na]$^+$: 424.1736; found: 424.1757.

3) Preparation of LM-4-d.

To a pressure bottle was added **LM-4-c** (1.0 g, 2.5 mmol), DMF (15 mL), potassium carbonate (1 g, 7.2 mmol) and ethyl iodide (4 mL, 50 mmol), the bottle was then well sealed. The reaction was kept stirring at 50 °C for 12 h. After that, cooled down the mixture to room temperature, the crude mixture was directly purified with a reversed-phase C18 column (CH_3CN/H_2O, 0.1% TFA) to offer pure **LM-4-d** (816.1 mg, 1.9 mmol, 76% yield). **^{1}H NMR** (400 MHz, Chloroform-d) δ 7.75 – 7.60 (m, 3H), 7.19 (dd, J = 9.2, 2.4 Hz, 1H), 6.78 (d, J = 2.1 Hz, 1H), 4.04 (s, 3H), 3.91 (s, 3H), 3.49 – 3.34 (m, 4H), 2.28 (t, J = 7.1 Hz, 2H), 1.89 (p, J = 7.2 Hz, 2H), 1.44 (s, 9H), 1.18 (t, J = 7.1 Hz, 3H). **^{13}C NMR** (101 MHz, Chloroform-d) δ

172.4, 170.2, 166.9, 146.9, 132.2, 131.4, 129.1, 129.0, 128.1, 125.3, 120.4, 118.2, 102.7, 80.5, 52.5, 52.5, 49.7, 45.2, 32.7, 28.1, 28.1, 28.1, 22.9, 12.1. **HRMS** m/z (ESI): calcd. for $C_{24}H_{32}NO_6$ $[M+H]^+$: 430.2230; found:430.2253.

4) Preparation of LM-4-e.

LM-4-d (816.1 mg, 1.9 mmol) and hydrazine hydrate (2.9 mL, 57 mmol) were dissolved with ethanol (8 mL), resulting mixture was kept stirring at 50 °C for 10 h. The reaction was monitored by LCMS and terminated when starting material mostly converted. After that, ethanol and residual hydrazine hydrate were removed with rotary evaporator to get a crude sample of **LM-4-e**. To this was added water (20 mL) and acetonitrile (20 mL), adjusting the pH to 2-3 with 1 N HCl to offer a crude solution of **LM-4-e** which was directly used for next step. MS m/z (ESI by Agilent 6120): calcd. for $C_{22}H_{28}N_3O_4$ $[M+H]^+$: 398.2; found: 398.0.

5) Preparation of LM-4.

To the above solution of **LM-4-e** was added hydrochloric acid (4M in dioxane, 48 mL, 192.0 mmol) slowly, resulting mixture was kept stirring at room temperature for 1 h after addition. The reaction was monitored by LCMS and terminated when starting material mostly converted. After that, most of the organic solvents were removed with rotary evaporator to get a residual. To this residual was added a small amount of DMF to form a clean solution, which was directly used for purification with a reversed-phase C18 column (CH_3CN/H_2O, 0.1% TFA) to offer pure **LM-4** (530 mg, 1.6 mmol, 84% yield). **^{1}H NMR** (400 MHz, DMSO-d_6) δ 9.32 – 9.24 (m, 1H), 8.09 (d, J = 8.6 Hz, 1H), 7.87 (d, J = 9.2 Hz, 1H), 7.59 (d, J = 8.5 Hz, 1H), 7.35 (dd, J = 9.1, 2.5 Hz, 1H), 3.52 – 3.47 (m, 2H), 3.45 – 3.41 (m, 2H), 2.33 (t, J = 7.3 Hz, 2H), 1.84 (p, J = 7.3 Hz, 2H), 1.16 (t, J = 7.0 Hz, 3H). **^{13}C NMR** (101 MHz, DMSO-d_6) δ 174.8, 160.0, 152.2, 147.9, 134.2, 132.3, 129.9, 126.6, 126.5, 121.6, 116.8, 115.2, 105.4, 49.5, 45.1, 31.3, 22.7, 12.3. **HRMS** m/z (ESI): calcd. for $C_{18}H_{20}N_3O_4$ $[M+H]^+$: 342.1454; found:342.1478.

5. Synthesis of LM-5.

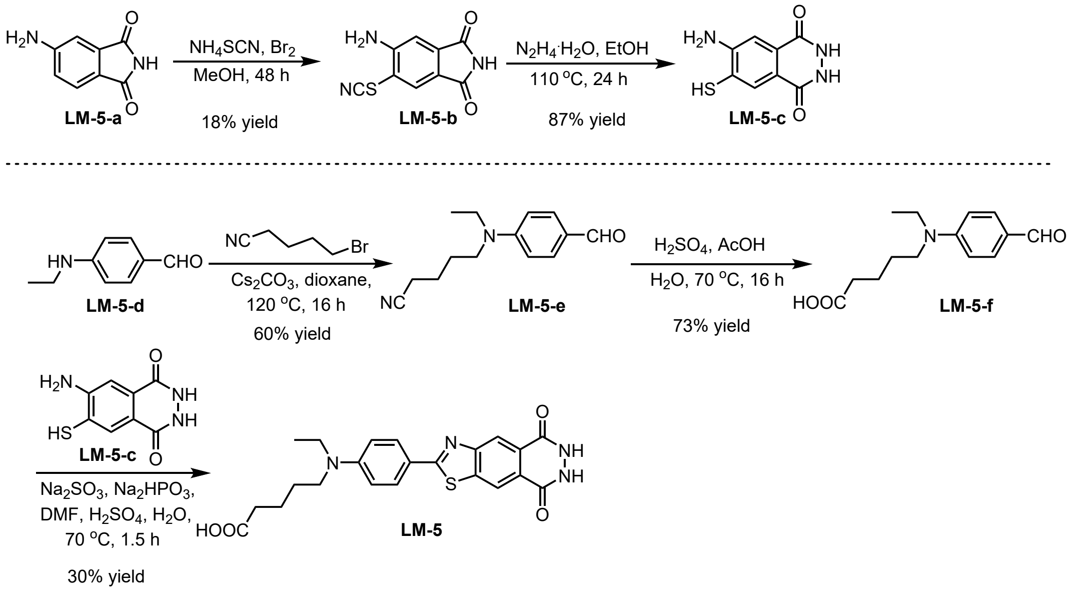

LM-5-b and **LM-5-c** are known compounds followed reported procedures.[1]

1) Preparation of LM-5-b.

Compound **LM-5-a** (3.2 g, 19.7 mmol) and NH_4SCN (3 g, 39.5 mmol) was added to methanol (150 mL), the mixture was cooled to 0 °C with ice bath. Bromine (1.7 mL, 33.2 mmol) was diluted with methanol (150 mL) before adding drop wise to the reaction with a pressure-equalizing dropping funnel. After addition, this resulting mixture was kept at 0 °C for one more hour followed by reacting at 25 °C for 48 h. The reaction was monitored by LCMS and terminated when starting material mostly converted. Pure **LM-5-b** (777mg, 18% yield) was obtained after purification with a reversed-phase C18 column (CH_3CN/H_2O, 0.1% TFA). MS m/z (ESI by Agilent 6120): calcd. for $C_9H_6N_3O_2S$ $[M+H]^+$: 220.0; found: 220.4.

2) Preparation of LM-5-c.

LM-5-b (1.5 g, 6.85 mmol) and hydrazine hydrate (1.5 g, 6.85 mmol) was dissolved with ethanol (20 mL), resulting mixture was refluxed for 24 h. The reaction was monitored by LCMS and terminated when starting material fully converted. After that, ethanol and residual hydrazine hydrate were removed with rotary evaporator, a crude sample of **LM-5-c** was obtained after adjusting the pH to 2-3 with 1 N HCl. This crude sample was further purified with a reversed-phase C18 column (CH_3CN/H_2O, 0.1% TFA) to offer pure **LM-5-c** (1.24 g, 87% yield). MS m/z (ESI by Agilent 6120): calcd. for $C_8H_8N_3O_2S$ $[M+H]^+$: 210.0; found:210.1.

3) Preparation of LM-5-e.

To a flask was added **LM-5-d** (1.0 g, 6.71 mmol), cesium carbonate (4.3 g, 13.2mmol), 5-bromovaleronitrile (22 g, 135.8 mmol) and 1,4-dioxane (2 mL), the mixture was refluxed under argon protection for 16 h. The reaction was monitored by TLC and terminated when starting material fully converted. After that, a viscous sample was obtained after removing solvents. This viscous sample was further purified with silica-gel column chromatography (hexane/EA=10:1) to offer a crude **LM-5-e** (927mg, 60% yield) which was directly used for next step. MS m/z (ESI by Agilent 6120): calcd. for $C_{14}H_{19}N_2O$ $[M+H]^+$: 231.1; found: 231.5.

4) Preparation of LM-5-f.

To a flask was added concentrated sulfuric acid (1 mL), purified water (1 mL), acetic acid (1 mL) and well mixed, followed by the addition of **LM-5-e** (1.0 g, 4.34 mmol), the resulting mixture was heated at 70 °C for 16 h. The reaction was monitored by LCMS and terminated when starting material fully converted. After that, the reaction was diluted with water, adjusted the pH to 2 by adding sodium bicarbonate portion wise. This crude mixture was then directly used for purification with a reversed-phase C18 column (CH_3CN/H_2O, 0.1% TFA) to offer pure **LM-5-f** (789 mg, 73% yield). **^{1}H NMR** (400 MHz, Chloroform-*d*) δ 9.62 – 9.53 (m, 2H), 7.68 (d, *J* = 8.9 Hz, 2H), 6.63 (d, *J* = 9.0 Hz, 2H), 3.41 (q, *J* = 7.1 Hz, 2H), 3.34 (t, *J* = 6.9 Hz, 2H), 2.39 (t, *J* = 6.6 Hz, 2H), 1.74 – 1.59 (m, 4H), 1.16 (t, *J* = 7.1 Hz, 3H). **^{13}C NMR** (101 MHz, Chloroform-*d*) δ 190.5, 178.4, 152.5, 132.6, 124.3, 110.7, 110.7, 110.7, 50.1, 45.2, 33.7, 26.8, 22.1, 12.2. MS m/z (ESI by Agilent 6120): calcd. for $C_{14}H_{20}NO_3$ $[M+H]^+$: 250.1; found: 250.5.

5) Preparation of LM-5.

To a tube was added **LM-5-f** (500 mg, 2.0 mmol), **LM-5-c** (836 mg, 4.0 mmol), sodium sulfite (160 mg, 1.27 mmol) and Na_2HPO_3 (493mg, 3.47 mmol), followed by the addition of DMF (40 mL) and 1.5 M sulfuric acid (40 mL). The resulting mixture was kept stirring at 80 °C for 1.5 h. The reaction was monitored by LCMS and terminated when starting material fully converted. After that, to the reaction was added ice (20 g), precipitation of solids was observed after cooling the mixture with ice bath. The solids were collected by filtration as a crude sample of **LM-5**, which then recrystallized with acetonitrile to offer pure **LM-5** (263mg, 30% yield). **^{1}H NMR** (400 MHz, DMSO-d_6) δ 11.69 (s, 3H), 8.76 (s, 1H), 8.40 (s, 1H), 7.94 (d, *J* = 8.9 Hz, 2H), 6.82 (d, *J* = 9.0 Hz, 2H), 3.49 – 3.43 (m, 2H), 3.39 (s, 2H), 2.28 (s, 2H), 1.58 (s, 4H), 1.14 (t, *J* = 6.9 Hz, 3H). **^{13}C NMR** (101 MHz, DMSO-d_6) δ 189.5, 174.4, 174.4, 172.3, 156.6, 152.1, 150.7, 139.0, 129.6, 129.6, 124.0, 119.8, 118.5, 111.4, 111.4, 110.7, 49.3, 44.4, 33.5, 26.6, 22.0, 12.2. **HRMS** m/z (ESI): calcd. for $C_{22}H_{23}N_4O_4S$ $[M+H]^+$: 439.1440; found:439.1441.

6. Synthesis of LM-6.

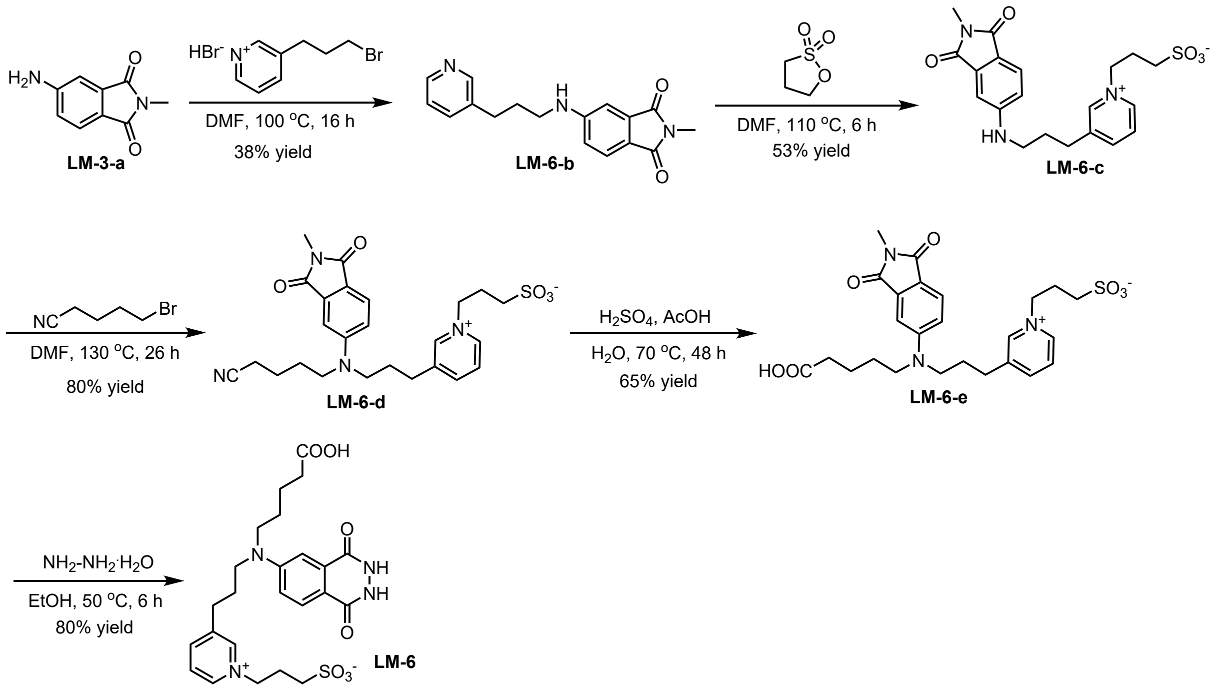

1) Preparation of LM-6-b.

To a flask was added **LM-3-a** (500 mg, 2.8 mmol) and 4-(3-bromopropyl)pyridine hydrobromide (1.5 g, 5.3 mmol), followed by the addition of DMF (30 mL), the resulting mixture was heated at 100 °C for 16 h under the protection of argon. The reaction was monitored by LCMS and terminated when starting material mostly converted. After that, the mixture was cooled to room temperature, crude mixture was directly used for purification with a reversed-phase C18 column (CH_3CN/H_2O, 0.1% TFA) to offer pure **LM-6-b** (300 mg, 38% yield). **^{1}H NMR** (400 MHz, Methanol-*d*$_4$) δ 8.43 (d, *J* = 1.9 Hz, 1H), 8.37 (dd, *J* = 4.9, 1.5 Hz, 1H), 7.75 (dt, *J* = 7.8, 1.8 Hz, 1H), 7.49 (d, *J* = 8.3 Hz, 1H), 7.38 (dd, *J* = 7.8, 4.9 Hz, 1H), 6.92 (d, *J* = 2.1 Hz, 1H), 6.76 (dd, *J* = 8.3, 2.2 Hz, 1H), 3.23 (t, *J* = 6.9 Hz, 2H), 3.03 (s, 3H), 2.86 – 2.74 (m, 2H), 1.98 (dt, *J* = 14.5, 7.0 Hz, 2H). **^{13}C NMR** (101 MHz, Methanol-*d*$_4$) δ 170.6, 170.5, 155.7, 149.9, 147.6, 139.5, 138.5, 136.3, 125.8, 125.3, 118.7, 116.0, 106.7, 43.4, 31.2, 31.1, 23.7. MS m/z (ESI by Agilent 6120): calcd. for $C_{17}H_{18}N_3O_2$ $[M+H]^+$: 296.1; found: 296.4.

2) Preparation of LM-6-c.

To a flask was added **LM-6-b** (1.0 g, 3.6 mmol) and 1,3-propanesultone (0.82 g, 6.0 mmol), followed by adding DMF (40 mL), resulting mixture was kept stirring at 110 °C for 6 h under protection of argon. The reaction was monitored by LCMS and terminated when starting material mostly converted. After that, the mixture was cooled to room temperature, crude mixture was directly used for purification with a reversed-phase C18 column (CH_3CN/H_2O, 0.1% TFA) to offer pure **LM-6-c** (797 mg, 53% yield). **^{1}H NMR** (400 MHz, DMSO-*d*$_6$) δ 9.04 (s, 1H), 8.94 (d, *J* = 6.0 Hz, 1H), 8.48 (d, *J* = 8.0 Hz, 1H), 8.06 (dd, *J* = 7.8, 6.2 Hz, 1H), 7.51 (d, *J* = 8.3 Hz, 1H), 7.09 (t, *J* = 5.3 Hz, 1H), 6.91 (d, *J* = 1.8 Hz, 1H), 6.79 (dd, *J* = 8.3, 1.9 Hz, 1H), 4.68 (t, *J* = 7.0 Hz, 2H), 3.20 (q, *J* = 6.5 Hz, 2H), 2.95 (s, 3H), 2.93 – 2.88 (m, 2H), 2.44 (t, *J* = 7.1 Hz, 2H), 2.24 (p, *J* = 7.0 Hz, 2H), 1.96 (p, *J* = 7.1 Hz, 2H). **^{13}C NMR** (101 MHz, DMSO-*d*$_6$) δ 168.5, 168.1, 163.6, 153.9, 145.2, 144.3, 142.6, 142.4, 134.6, 127.6, 124.7, 116.8, 114.8, 59.7, 47.1, 41.7, 29.4, 28.7, 27.3, 23.5. **HRMS** m/z (ESI): calcd. for $C_{20}H_{24}N_3O_5S$ $[M+H]^+$: 418.1437; found:418.1438.

3) Preparation of LM-6-d.

To a pressure bottle was added **LM-6-c** (500 mg, 1.20 mmol), 5-bromovaleronitrile (1.94 g, 12.0 mmol) and DMF (5 mL), the bottle was then well sealed and resulting mixture was heated at 130 °C for 26 h. The reaction was monitored by LCMS and terminated when starting material mostly converted. After that, reaction mixture was cooled down to room temperature, DMF (5 mL) was used to dilute the reaction. The crude reaction mixture was then directly used for purification with a reversed-phase C18 column (CH_3CN/H_2O, 0.1% TFA) to offer pure **LM-6-d** (479 mg, 80% yield). **^{1}H NMR** (400 MHz, DMSO-*d*$_6$) δ 9.04 (s, 1H), 8.94 (d, *J* = 6.0 Hz, 1H), 8.49 (d, *J* = 8.1 Hz, 1H), 8.06 (dd, *J* = 7.9, 6.1 Hz, 1H), 7.57 (d, *J* = 8.5 Hz, 1H), 7.00 (d, *J* = 2.1 Hz, 1H), 6.93 (dd, *J* = 8.6, 2.2 Hz, 1H), 4.68 (t, *J* = 7.0 Hz, 2H), 3.49 (dd, *J* = 15.8,

7.8 Hz, 4H), 2.96 (s, 3H), 2.93 – 2.84 (m, 2H), 2.60 – 2.52 (m, 2H), 2.44 (t, *J* = 7.1 Hz, 2H), 2.23 (p, *J* = 7.0 Hz, 2H), 1.94 (p, *J* = 8.3 Hz, 2H), 1.62 (s, 4H). **^{13}C NMR** (101 MHz, DMSO-*d*$_6$) δ 168.5, 168.0, 152.2, 145.0, 144.2, 142.6, 142.3, 134.7, 127.6, 124.7, 120.7, 116.5, 114.6, 105.1, 59.7, 49.6, 49.4, 47.1, 29.1, 27.4, 26.9, 25.7, 23.5, 22.2, 16.1. **HRMS** m/z (ESI): calcd. for $C_{25}H_{31}N_4O_5S$ [M+H]$^+$: 499.2015; found:499.2047.

4) Preparation of LM-6-e.

To a flask was added concentrated sulfuric acid (1.8 mL), purified water (0.9 mL), acetic acid (0.9 mL) and well mixed, followed by the addition of **LM-6-d** (900 mg, 1.8 mmol), the resulting mixture was heated at 70 °C for 16 h. The reaction was monitored by LCMS and terminated when starting material fully converted. After that, the reaction was diluted with H_2O（15 mL）, adjusted the pH to 2 by adding sodium bicarbonate portion wise. This crude mixture was then directly used for purification with a reversed-phase C18 column (CH_3CN/H_2O, 0.1% TFA) to offer pure **LM-6-e** (606 mg, 65% yield). **^{1}H NMR** (400 MHz, DMSO-*d*$_6$) δ 9.04 (s, 1H), 8.94 (d, *J* = 6.0 Hz, 1H), 8.50 (d, *J* = 8.1 Hz, 1H), 8.06 (dd, *J* = 8.0, 6.1 Hz, 1H), 7.56 (d, *J* = 8.5 Hz, 1H), 6.97 (d, *J* = 2.1 Hz, 1H), 6.90 (dd, *J* = 8.6, 2.2 Hz, 1H), 4.68 (t, *J* = 7.0 Hz, 2H), 3.56 – 3.46 (m, 2H), 3.43 (s, 2H), 3.35 (s, 2H), 2.96 (s, 3H), 2.91 – 2.85 (m, 2H), 2.44 (t, *J* = 7.1 Hz, 2H), 2.24 (dd, *J* = 12.7, 5.5 Hz, 4H), 1.94 (p, *J* = 8.0 Hz, 2H), 1.55 (s, 4H). **^{13}C NMR** (101 MHz, DMSO-*d*$_6$) δ 174.4, 168.5, 168.0, 152.2, 145.0, 144.2, 142.6, 142.3, 134.7, 127.5, 124.7, 116.3, 114.5, 105.0, 59.7, 50.0, 49.7, 47.1, 33.4, 29.1, 27.4, 27.0, 26.0, 23.5, 21.9. **HRMS** m/z (ESI): calcd. for $C_{25}H_{32}N_3O_7S$ [M+H]$^+$: 518.1961; found:518.1960.

5) Preparation of LM-6.

To a flask was added **LM-6-e** (500 mg, 0.97 mmol), hydrazine hydrate (966.0 mg, 19.3 mmol) and ethanol (5 mL), resulting mixture was heated at 50 °C for 6 h. After that, ethanol and residual hydrazine hydrate were removed with rotary evaporator, a crude sample of **LM-6** was obtained after adjusting the pH to 2-3 with 1 N HCl. This crude sample was dissolved again with H_2O（10 mL）and acetonitrile（10 mL）to get a clean solution which was then used for purification with a reversed-phase C18 column (CH_3CN/H_2O, 0.1% TFA) to offer pure **LM-6** (415 mg, 80 % yield). **^{1}H NMR** (400 MHz, DMSO-*d*$_6$) δ 9.06 (s, 1H), 8.94 (d, *J* = 6.0 Hz, 1H), 8.50 (d, *J* = 8.1 Hz, 1H), 8.05 (dd, *J* = 7.9, 6.1 Hz, 1H), 7.82 (d, *J* = 9.0 Hz, 1H), 7.19 (dd, *J* = 9.1, 2.4 Hz, 1H), 6.95 (d, *J* = 1.9 Hz, 1H), 4.69 (t, *J* = 7.0 Hz, 2H), 3.46 (dd, *J* = 18.1, 10.7 Hz, 4H), 2.88 (t, *J* = 7.6 Hz, 2H), 2.47 (t, *J* = 7.1 Hz, 2H), 2.31 – 2.18 (m, 4H), 2.02 – 1.88 (m, 2H), 1.55 (s, 4H). **^{13}C NMR** (101 MHz, DMSO-*d*$_6$) δ 174.5, 155.6, 154.3, 150.4, 145.1, 144.2, 142.6, 142.4, 128.8, 127.6, 127.1, 116.8, 115.5, 103.5, 59.7, 49.8, 49.4, 47.2, 33.6, 29.2, 27.4, 27.0, 26.1, 22.0. **HRMS** m/z (ESI): calcd. for $C_{24}H_{31}N_4O_7S$ [M+H]$^+$:519.1913; found: 519.1936.

7. Synthesis of LM-7.

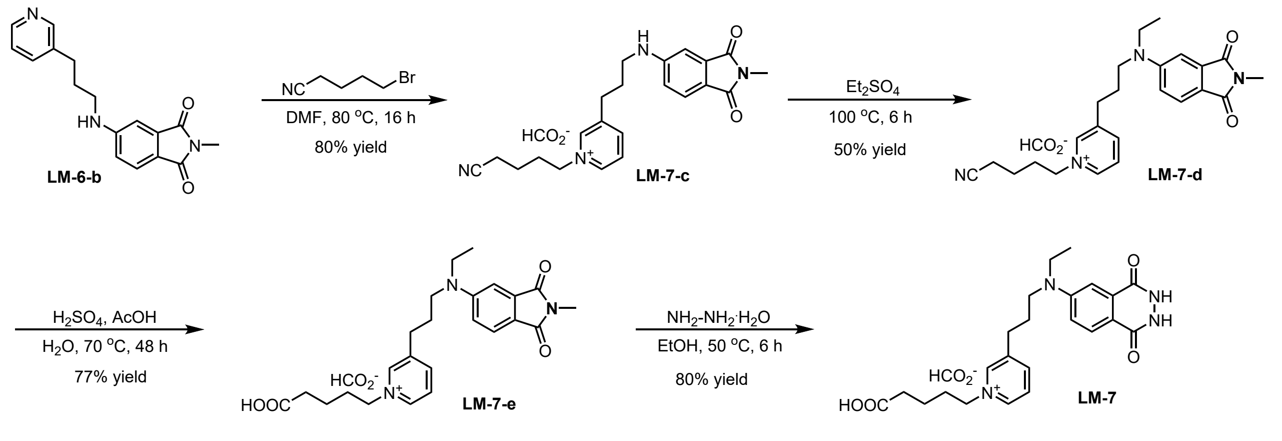

1) Preparation of LM-7-c.

To a flask was added **LM-6-b** (500 mg, 1.69 mmol), 5-bromovaleronitrile (317 mg, 1.96 mmol) and DMF (10 mL), resulting mixture was kept stirring at 80 °C for 16 h. The reaction was monitored by LCMS and terminated when starting material mostly converted. After that, reaction was cooled down to room temperature, the crude mixture was directly used for purification with a reversed-phase C18 column (CH_3CN/H_2O, 0.1% TFA) to offer pure **LM-7-c** (571 mg, 80% yield). **^{1}H NMR** (400 MHz, Methanol-*d*$_4$) δ 8.96 (s, 1H), 8.85 (d, *J* = 6.0 Hz, 1H), 8.52 (d, *J* = 6.4 Hz, 2H), 8.11 – 7.97 (m, 1H), 7.50 (d, *J* = 8.3 Hz, 1H), 6.90 (d, *J* = 2.0 Hz, 1H), 6.79 (dd, *J* = 8.3, 2.1 Hz, 1H), 4.85 (s, 2H), 4.64 (t, *J* = 7.5 Hz, 2H), 3.03 (s, 5H), 2.54 (t, *J* = 7.1 Hz, 2H), 2.10 (tt, *J* = 14.0, 7.3 Hz, 4H), 1.73 (dt, *J* = 14.8, 7.1 Hz, 2H). **^{13}C NMR** (101 MHz, Methanol-*d*$_4$) δ

170.5, 170.4, 155.5, 146.9, 145.4, 145.1, 143.6, 136.3, 129.1, 125.8, 120.5, 119.0, 116.4, 106.5, 62.0, 43.1, 31.41, 31.1, 30.3, 23.7, 23.3, 16.9. **HRMS** m/z (ESI): calcd. for $C_{22}H_{25}N_4O_2$ $[M+H]^{2+}$: 378.2056.; found:378.2041.

2) Preparation of LM-7-d.

To a flask was added **LM-7-c** (300 mg, 0.67 mmol) and diethyl sulfate (3 g, 19.46 mmol), resulting mixture was heated at 100 °C for 16 h. The reaction was monitored by LCMS and terminated when starting material mostly converted. After that, reaction was cooled down to room temperature, the crude mixture was directly used for purification with a reversed-phase C18 column (CH_3CN/H_2O, 0.1% TFA) to offer crude **LM-7-d** (151 mg, 50% yield). MS m/z (ESI by Agilent 6120): calcd. for $C_{24}H_{29}N_4O_2$ $[M]^+$: 405.2; found: 405.4.

3) Preparation of LM-7-e.

To a flask was added concentrated sulfuric acid (1.0 mL), purified water (0.5 mL), acetic acid (0.5 mL) and well mixed, followed by the addition of **LM-7-d** (500 mg, 1.11 mmol), the resulting mixture was heated at 70 °C for 48 h. The reaction was monitored by LCMS and terminated when starting material fully converted. After that, the reaction was diluted with H_2O (15 mL), adjusted the pH to 2 by adding sodium bicarbonate portion wise. This crude mixture was then directly used for purification with a reversed-phase C18 column (CH_3CN/H_2O, 0.1% TFA) to offer pure **LM-7-e** (401 mg, 77% yield). **^{1}H NMR** (400 MHz, Deuterium Oxide) δ 8.75 (s, 1H), 8.70 (d, *J* = 6.0 Hz, 1H), 8.42 (d, *J* = 8.1 Hz, 1H), 7.98 (dd, *J* = 7.8, 6.3 Hz, 1H), 7.00 (d, *J* = 8.6 Hz, 1H), 6.47 (dd, *J* = 8.7, 1.9 Hz, 1H), 6.34 (d, *J* = 1.8 Hz, 1H), 4.56 (t, *J* = 7.2 Hz, 2H), 3.28 (dt, *J* = 24.7, 7.1 Hz, 4H), 2.89 (t, *J* = 7.6 Hz, 2H), 2.70 (s, 3H), 2.34 (t, *J* = 7.3 Hz, 2H), 1.96 (tq, *J* = 15.7, 8.1 Hz, 4H), 1.55 (p, *J* = 7.4 Hz, 2H), 1.04 (t, *J* = 7.0 Hz, 3H). **^{13}C NMR** (101 MHz, Deuterium Oxide) δ 176.0, 168.2, 167.9, 150.4, 143.5, 141.4, 141.4, 140.2, 131.8, 126.1, 122.8, 112.7, 112.2, 103.0, 59.5, 47.4, 43.4, 31.2, 28.1, 27.5, 25.3, 21.3, 19.0, 9.5. **HRMS** m/z (ESI): calcd. for $C_{24}H_{30}N_3O_4$ $[M]^+$: 424.2236; found: 424.2263.

4) Preparation of LM-7.

To a flask was added **LM-7-e** (200 mg，0.43 mmol), hydrazine hydrate (440 mg，7.47 mmol) and ethanol (2 mL), resulting mixture was heated at 50 °C for 6 h. After that, ethanol and residual hydrazine hydrate were removed with rotary evaporator, a crude sample of **LM-7** was obtained after adjusting the pH to 2-3 with 1 N HCl. This crude sample was dissolved again with H_2O（10 mL）and acetonitrile（10 mL）to get a clean solution which was then used for purification with a reversed-phase C18 column (CH_3CN/H_2O, 0.1% TFA) to offer pure **LM-7** (162 mg, 80% yield). **^{1}H NMR** (400 MHz, DMSO-d_6) δ 9.11 (s, 1H), 8.98 (d, *J* = 6.0 Hz, 1H), 8.52 (d, *J* = 8.1 Hz, 1H), 8.06 (dd, *J* = 8.0, 6.1 Hz, 1H), 7.86 (d, *J* = 9.0 Hz, 1H), 7.26 (d, *J* = 7.9 Hz, 1H), 7.06 (s, 1H), 4.58 (t, *J* = 7.3 Hz, 2H), 3.55 – 3.39 (m, 4H), 2.88 (t, *J* = 7.6 Hz, 2H), 2.26 (t, *J* = 7.3 Hz, 2H), 1.93 (tt, *J* = 15.1, 7.8 Hz, 4H), 1.47 (p, *J* = 7.4 Hz, 2H), 1.11 (t, *J* = 7.0 Hz, 3H). **^{13}C NMR** (101 MHz, DMSO-d_6) δ 174.1, 159.8, 155.6, 154.4, 150.1, 145.2, 144.1, 142.5, 128.8, 127.7, 127.2, 117.1, 115.7, 103.9, 60.4, 49.1, 44.7, 33.0, 30.2, 29.2, 27.2, 21.0, 11.9. **HRMS** m/z (ESI): calcd. for $C_{23}H_{30}N_4O_4$ $[M+H]^{2+}$: 426.2267; found: 426.2269.

8. Synthesis of LM-8.

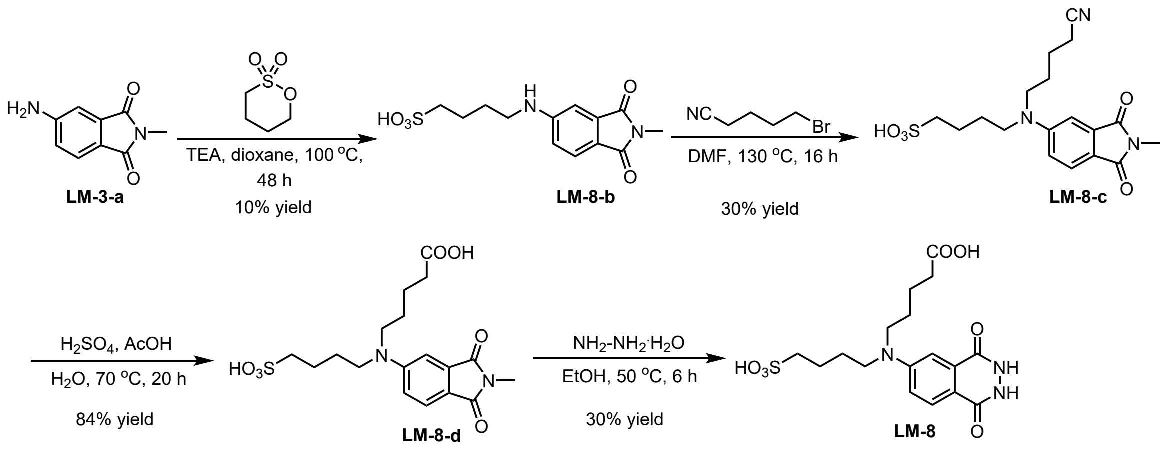

1) Preparation of LM-8-b.

To a flask was added **LM-3-a** (3 g, 17.03 mmol), 1,4-butylenesulfone (23.2 g, 170.3 mmol) and triethylamine (5.16 g, 51.09 mmol), followed by the addition of 1,4-dioxane (60 mL), the reaction was heated at 100 °C for 48 h under argon protection. The conversion was around 15% by LCMS. After that, reaction was cooled down to room temperature, the crude mixture was directly used for purification with a reversed-phase C18 column (CH_3CN/H_2O, 0.1% TFA) to offer pure **LM-8-b** (531 mg, 10% yield). **^{1}H NMR** (400 MHz, DMSO-d_6) δ 7.49 (d, J = 8.3 Hz, 1H), 6.91 (d, J = 2.0 Hz, 1H), 6.78 (dd, J = 8.4, 2.1 Hz, 1H), 3.15 – 3.05 (m, 4H), 2.94 (s, 3H), 2.56 – 2.51 (m, 2H), 1.76 – 1.55 (m, 4H), 1.17 (t, J = 7.3 Hz, 4H). **^{13}C NMR** (101 MHz, DMSO-d_6) δ 168.5, 168.2, 154.2, 134.6, 124.7, 116.4, 114.6, 105.3, 51.1, 45.8, 27.5, 23.4, 22.7. **HRMS** m/z (ESI): calcd. for $C_{13}H_{17}N_2O_5S$ $[M+H]^+$:313.0858; found: 313.0873.

2) Preparation of LM-8-c.

To a pressure bottle was added **LM-8-b** (460 mg, 1.47 mmol), 5-bromovaleronitrile (2.38 g, 14.7 mmol) and DMF (4 mL), the bottle was well sealed, then resulting mixture was heated at 130 °C for 16 h. The reaction was monitored by LCMS and terminated when starting material fully converted. After that, reaction was cooled down to room temperature, the crude mixture was directly used for purification with a reversed-phase C18 column (CH_3CN/H_2O, 0.1% TFA) to offer pure **LM-8-c** (173 mg, 30% yield).**^{1}H NMR** (400 MHz, DMSO-d_6) δ 7.55 (d, J = 8.5 Hz, 1H), 7.00 (d, J = 2.2 Hz, 1H), 6.91 (dd, J = 8.6, 2.2 Hz, 1H), 3.46 – 3.38 (m, 3H), 2.96 (s, 2H), 2.67 – 2.59 (m, 2H), 2.53 – 2.48 (m, 1H), 1.62 (s, 7H). **^{13}C NMR** (101 MHz, DMSO-d_6) δ 168.5, 168.0, 152.2, 134.8, 124.7, 120.7, 116.2, 114.5, 105.0, 51.1, 50.1, 49.5, 25.8, 25.6, 23.5, 22.2, 22.2, 16.1. **HRMS** m/z (ESI): calcd. for $C_{18}H_{24}N_3O_5S$ $[M+H]^+$: 394.1437; found: 394.1475.

3) Preparation of LM-8-d.

To a flask was added concentrated sulfuric acid (3 mL), purified water (1.5 mL), acetic acid (1.5 mL) and well mixed, followed by the addition of **LM-8-c** (160 mg, 0.4 mmol), the resulting mixture was heated at 70 °C for 20 h. The reaction was monitored by LCMS and terminated when starting material fully converted. After that, the reaction was diluted with H_2O (15 mL), adjusted the pH to 2 by adding sodium bicarbonate portion wise. This crude mixture was then directly used for purification with a reversed-phase C18 column (CH_3CN/H_2O, 0.1% TFA) to offer pure **LM-8-d** (138 mg, 84% yield). **^{1}H NMR** (400 MHz, DMSO-d_6) δ 10.10 (s, 2H), 7.53 (d, J = 8.5 Hz, 1H), 6.96 (d, J = 2.0 Hz, 1H), 6.88 (dd, J = 8.6, 2.1 Hz, 1H), 3.41 (d, J = 6.4 Hz, 4H), 2.95 (s, 3H), 2.68 – 2.58 (m, 2H), 2.25 (s, 2H), 1.70 – 1.58 (m, 4H), 1.54 (s, 4H). **^{13}C NMR** (101 MHz, DMSO-d_6) δ 174.4, 168.5, 168.0, 152.2, 134.7, 124.7, 116.1, 114.5, 105.0, 51.1, 50.2, 50.1, 33.5, 26.1, 25.6, 23.5, 22.2, 21.9. **HRMS** m/z (ESI): calcd. for $C_{18}H_{24}N_2O_7S$ $[M+H]^+$: 413.1382; found: 413.1364.

4) Preparation of LM-8.

To a flask was added **LM-8-d** (140 mg, 0.34 mmol), hydrazine hydrate (340 mg, 6.8 mmol) and ethanol (7 mL), resulting mixture was heated at 50 °C for 6 h. After that, ethanol and residual hydrazine hydrate were removed with rotary evaporator, a crude sample of **LM-8** was obtained after adjusting the pH to 2-3 with 1 N HCl. This crude sample was dissolved again with H_2O（10 mL）and acetonitrile（10 mL）to get a clean solution which was then used for purification with a reversed-phase C18 column (CH_3CN/H_2O, 0.1% TFA) to offer pure **LM-8** (42 mg, 30% yield). **^{1}H NMR** (400 MHz, DMSO-d_6) δ 7.84 (d, J = 9.0 Hz, 1H), 7.20 (dd, J = 9.1, 2.3 Hz, 1H), 7.06 – 7.00 (m, 1H), 3.40 (s, 4H), 2.66 – 2.59 (m, 2H), 2.25 (t, J = 6.6 Hz, 2H), 1.65 (d, J = 10.1 Hz, 4H), 1.55 (s, 4H). **^{13}C NMR** (101 MHz, DMSO-d_6) δ 174.4, 155.6, 154.4, 150.5, 128.8, 127.1, 117.1, 115.4, 103.7, 51.1, 50.1, 50.1, 33.6, 26.1, 25.6, 22.3, 22.0. **HRMS** m/z (ESI): calcd. for $C_{17}H_{24}N_3O_7S$ $[M+H]^+$: 414.1335; found: 414.1396.

9. Synthesis of LM-9.

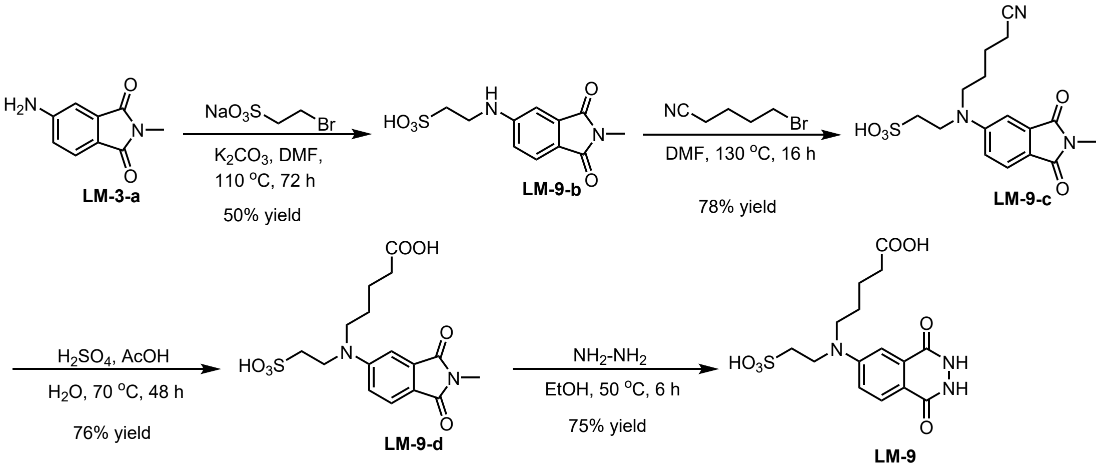

1) Preparation of LM-9-b.

To a flask was added **LM-3-a** (2 g, 11.35 mmol), sodium 2-bromoethanesulphonate (24 g, 113.7 mmol), potassium carbonate (7.9 g, 57.2 mmol) and DMF (40mL), the resulting mixture was heated at 110 °C for 72 h. The reaction was monitored by LCMS and terminated when starting material mostly converted. After that, the reaction was cooled down to room temperature, water was added to make the solution clear. This crude mixture was then directly used for purification with a reversed-phase C18 column (CH_3CN/H_2O, 0.1% TFA) to offer pure **LM-9-b** (1.6 g, 50% yield). **^{1}H NMR** (400 MHz, DMSO-d_6) δ 7.49 (d, J = 8.2 Hz, 1H), 6.85 (s, 1H), 6.75 (d, J = 7.9 Hz, 1H), 3.41 (t, J = 6.9 Hz, 2H), 2.92 (s, 3H), 2.76 (t, J = 6.9 Hz, 2H). **^{13}C NMR** (101 MHz, DMSO-d_6) δ 168.7, 168.5, 153.8, 134.8, 125.0, 117.3, 115.1, 105.7, 49.8, 49.8, 23.7. **HRMS** m/z (ESI): calcd. for $C_{11}H_{13}N_2O_5S$ $[M+H]^+$: 285.0545; found: 285.0536.

2) Preparation of LM-9-c.

To a pressure bottle was added **LM-9-b** (1.0 g, 3.52 mmol), 5-bromovaleronitrile (5.7 g, 35.2 mmol) and DMF (10 mL), the bottle was well sealed, then resulting mixture was heated at 130 °C for 16 h. The reaction was monitored by LCMS and terminated when starting material fully converted. After that, reaction was cooled down to room temperature, the crude mixture was directly used for purification with a reversed-phase C18 column (CH_3CN/H_2O, 0.1% TFA) to offer pure **LM-9-c** (1.0 g, 78% yield). **^{1}H NMR** (400 MHz, DMSO-d_6) δ 8.22 (s, 1H), 7.57 (d, J = 8.5 Hz, 1H), 7.00 (s, 1H), 6.91 (dd, J = 8.6, 1.8 Hz, 1H), 3.72 – 3.63 (m, 2H), 3.47 (s, 2H), 2.96 (s, 3H), 2.79 – 2.71 (m, 2H), 2.56 (t, J = 5.5 Hz, 4H), 1.62 (s, 2H). **^{13}C NMR** (101 MHz, DMSO-d_6) δ 168.4, 168.0, 152.0, 134.8, 124.7, 120.7, 116.6, 114.4, 105.0, 49.7, 47.6, 34.4, 26.0, 23.5, 22.3, 16.1. **HRMS** m/z (ESI): calcd. for $C_{16}H_{20}N_3O_5S$ $[M+H]^+$: 366.1124; found:366.1178.

3) Preparation of LM-9-d.

To a flask was added concentrated sulfuric acid (2 mL), purified water (1 mL), acetic acid (1 mL) and well mixed, followed by the addition of **LM-9-c** (500 mg, 1.37 mmol), the resulting mixture was heated at 70 °C for 48 h. The reaction was monitored by LCMS and terminated when starting material fully converted. After that, the reaction was diluted with H_2O (15 mL), adjusted the pH to 2 by adding sodium bicarbonate portion wise. This crude mixture was then directly used for purification with a reversed-phase C18 column (CH_3CN/H_2O, 0.1% TFA) to offer pure **LM-9-d** (400 mg, 76% yield) **^{1}H NMR** (400 MHz, DMSO-d_6) δ 7.57 (d, J = 8.5 Hz, 1H), 6.98 (s, 1H), 6.89 (d, J = 8.5 Hz, 1H), 3.73 – 3.60 (m, 2H), 3.44 (s, 2H), 2.96 (s, 3H), 2.80 – 2.65 (m, 2H), 2.26 (s, 2H), 1.54 (s, 4H). **^{13}C NMR** (101 MHz, DMSO-d_6) δ 172.8, 168.4, 168.0, 152.0, 134.8, 124.7, 116.4, 114.3, 104.9, 59.8, 50.2, 47.6, 33.3, 26.1, 21.9, 14.1. **HRMS** m/z (ESI): calcd. for $C_{16}H_{21}N_2O_7S$ $[M+H]^+$: 385.1069; found:385.1097.

4) Preparation of LM-9.

To a flask was added **LM-9-d** (200 mg, 0.52 mmol), hydrazine hydrate (520 mg, 8.83 mmol) and ethanol (2 mL), resulting mixture was heated at 50 °C for 6 h. After that, ethanol and residual hydrazine hydrate were removed with rotary evaporator, a crude sample of **LM-9** was obtained after adjusting the pH to 2-3 with 1 N HCl. This crude sample was dissolved again with H_2O（10 mL）and acetonitrile（10 mL）to get a clean solution which was then used for purification with a reversed-phase C18 column (CH_3CN/H_2O, 0.1% TFA) to offer pure **LM-9** (150 mg, 75% yield). **^{1}H NMR** (400 MHz,

DMSO-d_6) δ 7.85 (d, J = 9.0 Hz, 1H), 7.18 (dd, J = 9.0, 2.2 Hz, 1H), 7.08 – 7.05 (m, 1H), 3.76 – 3.61 (m, 2H), 3.42 (s, 2H), 2.85 – 2.69 (m, 2H), 2.27 (d, J = 6.3 Hz, 2H), 1.55 (s, 4H). **^{13}C NMR** (101 MHz, DMSO-d_6) δ 174.4, 155.7, 154.3, 150.4, 128.8, 127.2, 116.8, 115.6, 103.7, 50.1, 47.7, 47.1, 33.5, 26.3, 22.1. **HRMS** m/z (ESI): calcd. for $C_{15}H_{20}N_3O_7S$ [M+H]$^+$: 386.1022; found: 386.1025.

10. Synthesis of LM-10.

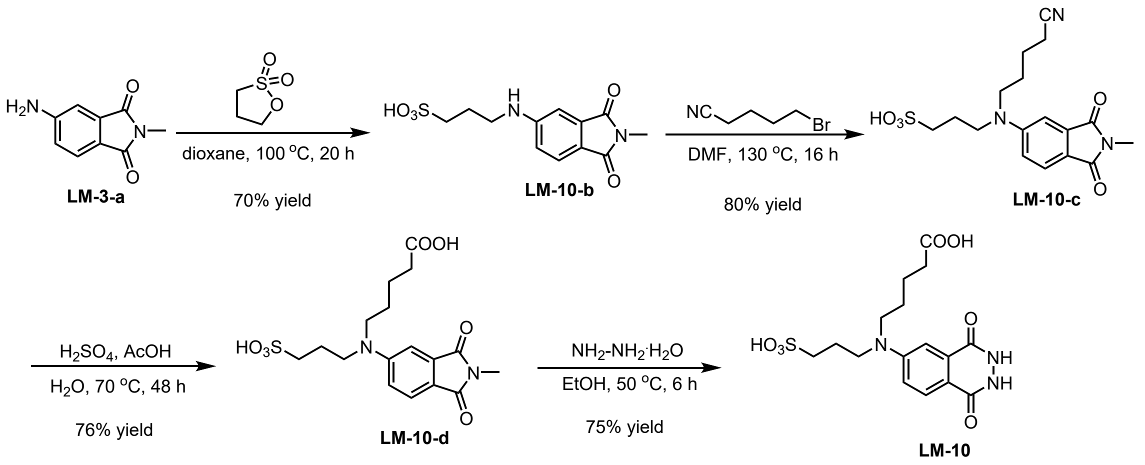

1) Preparation of LM-10-b.

To a flask was added **LM-3-a** (3 g, 17.03 mmol), 1,3-propanesultone (20.8 g, 170.3 mmol) and 1,4-dioxane (55mL), resulting mixture was heated at 100 °C for 20 h. The reaction was monitored by LCMS and terminated when starting material mostly converted. After that, reaction mixture was filtered at a high temperature, the solids were collected and washed with hot 1,4-dioxane for three times. Then solvents were removed to offer the pure **LM-10-b** (3.2 g, 70% yield). **^{1}H NMR** (400 MHz, DMSO-d_6) δ 7.50 (d, J = 8.3 Hz, 1H), 6.90 (d, J = 2.0 Hz, 1H), 6.78 (dd, J = 8.3, 2.1 Hz, 1H), 3.23 (t, J = 7.0 Hz, 2H), 2.95 (s, 3H), 2.60 – 2.52 (m, 2H), 1.86 (p, J = 7.1 Hz, 2H). **^{13}C NMR** (101 MHz, DMSO-d_6) δ 168.7, 168.5, 154.2, 134.8, 124.9, 116.8, 114.90, 105.7, 49.2, 41.9, 24.6, 23.6. MS m/z (ESI by Agilent 6120): calcd. for $C_{12}H_{17}N_2O_3S$ [M+H]$^+$: 269.1; found: 269.0.

2) Preparation of LM-10-c.

To a pressure bottle was added **LM-10-b** (800 mg, 2.98 mmol), 5-bromovaleronitrile (4.35 g, 26.85 mmol) and DMF (8 mL), the bottle was well sealed, then resulting mixture was heated at 130 °C for 16 h. The reaction was monitored by LCMS and terminated when starting material fully converted. After that, reaction was cooled down to room temperature, the crude mixture was directly used for purification with a reversed-phase C18 column (CH_3CN/H_2O, 0.1% TFA) to offer pure **LM-10-c** (904 mg, 80% yield). **^{1}H NMR** (400 MHz, DMSO-d_6) δ 7.54 (d, J = 8.5 Hz, 1H), 7.02 (d, J = 2.2 Hz, 1H), 6.94 (dd, J = 8.6, 2.2 Hz, 1H), 3.57 – 3.49 (m, 2H), 3.45 (s, 2H), 2.95 (s, 3H), 2.62 (t, J = 7.3 Hz, 2H), 2.55 (q, J = 4.2, 3.8 Hz, 2H), 1.85 (p, J = 7.8 Hz, 2H), 1.61 (s, 4H). **^{13}C NMR** (101 MHz, DMSO-d_6) δ 168.5, 168.1, 152.2, 134.8, 124.7, 120.7, 116.4, 114.6, 105.2, 49.5, 49.4, 48.5, 25.8, 23.5, 22.6, 22.3, 16.1. **HRMS** m/z (ESI): calcd. for $C_{17}H_{22}N_3O_5S$ [M+H]$^+$: 380.1280; found:380.1292.

3) Preparation of LM-10-d.

To a flask was added concentrated sulfuric acid (8 mL), purified water (4 mL), acetic acid (4 mL) and well mixed, followed by the addition of **LM-10-c** (800 mg, 2.1 mmol), the resulting mixture was heated at 70°C for 48 h. The reaction was monitored by LCMS and terminated when starting material fully converted. After that, the reaction was diluted with H_2O (15 mL), adjusted the pH to 2 by adding sodium bicarbonate portion wise. This crude mixture was then directly used for purification with a reversed-phase C18 column (CH_3CN/H_2O, 0.1% TFA) to offer pure **LM-10-d** (635 mg, 76% yield). **^{1}H NMR** (400 MHz, DMSO-d_6) δ 7.54 (d, J = 8.5 Hz, 1H), 7.01 (s, 1H), 6.93 (d, J = 8.6 Hz, 1H), 3.58 – 3.47 (m, 2H), 3.42 (s, 2H), 2.96 (s, 3H), 2.58 (t, J = 7.3 Hz, 2H), 2.25 (s, 1H), 2.08 (d, J = 11.3 Hz, 1H), 1.93 – 1.76 (m, 2H), 1.54 (s, 4H). **^{13}C NMR** (101 MHz, DMSO-d_6) δ 174.7, 168.8, 168.4, 152.5, 134.9, 124.9, 116.2, 114.7, 105.2, 50.2, 49.7, 48.7, 33.7, 26.3, 23.7, 22.9, 22.1. **HRMS** m/z (ESI): calcd. for $C_{17}H_{23}N_2O_7S$ [M+H]$^+$: 399.1226; found:399.1247.

4) Preparation of LM-10.

To a flask was added **LM-10-d** (700 mg, 1.76 mmol), hydrazine hydrate (1.76 g, 35.2 mmol) and ethanol (7 mL), resulting mixture was heated at 50 °C for 6 h. After that, ethanol and residual hydrazine hydrate were removed with rotary evaporator, a crude sample of **LM-10** was obtained after adjusting the pH to 2-3 with 1 N HCl. This crude sample was dissolved again with H_2O（10 mL）and acetonitrile（10 mL）to get a clear solution which was then used for purification with a reversed-phase C18 column (CH_3CN/H_2O, 0.1% TFA) to offer pure **LM-10** (527 mg, 75% yield). **^{1}H NMR** (400 MHz, DMSO-*d*$_6$) δ 7.84 (d, *J* = 9.0 Hz, 1H), 7.24 (dd, *J* = 9.1, 2.5 Hz, 1H), 7.04 (d, *J* = 2.4 Hz, 1H), 3.56 – 3.46 (m, 2H), 3.41 (s, 2H), 2.58 (t, *J* = 7.4 Hz, 2H), 2.25 (t, *J* = 6.5 Hz, 2H), 1.86 (p, *J* = 7.9 Hz, 2H), 1.54 (s, 4H). **^{13}C NMR** (101 MHz, DMSO-*d*$_6$) δ 174.8, 156.1, 154.8, 150.9, 128.9, 127.4, 117.3, 115.5, 103.9, 50.2, 49.5, 48.9, 34.7, 33.8, 26.3, 22.2. **HRMS** m/z (ESI): calcd. for $C_{16}H_{21}N_3O_7SNa$ $[M+Na]^+$: 422.0998; found: 422.0998.

11. Synthesis of LM-11.

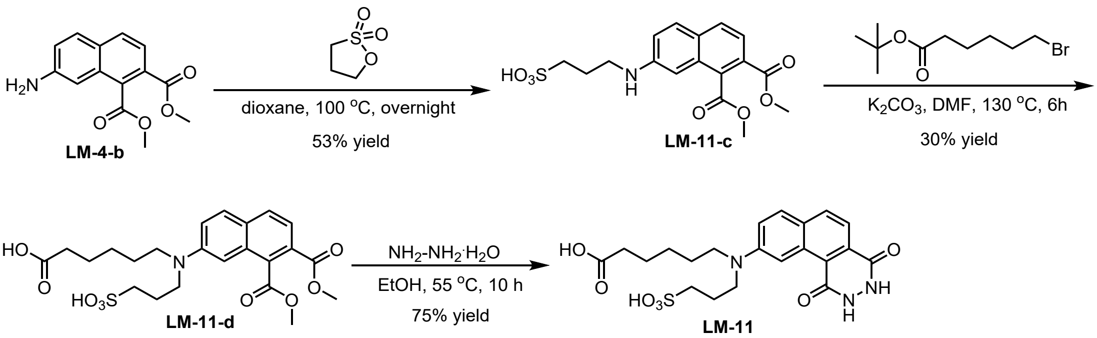

1) Preparation of LM-11-c.

To a flask was added **LM-4-b** (1 g, 3.9 mmol) and 1,3-propanesultone (2.4 g, 19.3 mmol), followed by the addition of dioxane (20 mL), resulting mixture was heated at 100°C for 12 h. The reaction was monitored by LCMS and terminated when starting material fully converted. After that, reaction mixture was cooled down to room temperature, DCM (40 mL) was added to precipitate solids which was then collected through filtration. Water (20 mL) was used to dissolved solids, resulting solution was directly used for purification with a reversed-phase C18 column (CH_3CN/H_2O, 0.1% TFA) to offer pure **LM-11-c** (780 mg, 53% yield). **^{1}H NMR** (400 MHz, DMSO-*d*$_6$) δ 7.97 (dd, *J* = 15.7, 8.8 Hz, 2H), 7.72 (d, *J* = 8.5 Hz, 1H), 7.43 (dd, *J* = 8.9, 1.7 Hz, 1H), 7.10 (s, 1H), 3.95 (s, 3H), 3.87 (s, 3H), 3.32 (t, *J* = 6.9 Hz, 2H), 2.73 (t, *J* = 7.3 Hz, 2H), 1.97 (p, *J* = 7.0 Hz, 2H). **^{13}C NMR** (101 MHz, DMSO-*d*$_6$) δ 168.6, 166.1, 143.8, 132.0, 130.2, 130.1, 129.8, 129.7, 125.5, 121.8, 121.7, 106.7, 52.8, 52.8, 49.1, 45.2, 23.4. **HRMS** m/z (ESI): calcd. for $C_{17}H_{20}NO_7S$ $[M+H]^+$: 382.0960; found:382.0991.

2) Preparation of LM-11-d.

To a flask was added **LM-11-c** (500 mg, 1.3 mmol)，DMF (15 mL)，tert-butyl 6-bromohexanoate (5.5 mL, 26.2 mmol) and potassium carbonate (542 mg, 3.9 mmol)，resulting mixture was kept stirring at 130°C for 6 h. The reaction was monitored by LCMS and terminated when starting material mostly converted. After that, reaction mixture was cooled down to room temperature, a clear solution was obtained through filtration. This solution was further used for purification with a reversed-phase C18 column (CH_3CN/H_2O, 0.1% TFA) to offer a crude **LM-11-d** (200 mg，0.4 mmol，30% yield), which was directly used for next step. MS m/z (ESI by Agilent 6120): calcd. for $C_{23}H_{28}NO_9S$ $[M-H]^+$: 494.1; found: 494.0.

3) Preparation of LM-11.

To a flask was added **LM-11-d** (200 mg，0.4 mmol), hydrazine hydrate (405 uL，8 mmol) and ethanol (8 mL), resulting mixture was heated at 55 °C for 10 h. After that, ethanol and residual hydrazine hydrate were removed with rotary evaporator, a crude sample of **LM-11** was obtained after adjusting the pH to 2-3 with 1 N HCl. This crude sample was dissolved again with H_2O (10 mL) and acetonitrile (10 mL) to get a clear solution which was then used for purification with a reversed-phase C18 column (CH_3CN/H_2O, 0.1% TFA) to offer pure **LM-11** (140 mg，0.3 mmol，75% yield). **^{1}H NMR** (400 MHz, DMSO-*d*$_6$) δ 9.71 (s, 1H), 8.27 (d, *J* = 8.7 Hz, 1H), 8.12 (d, *J* = 9.0 Hz, 1H), 7.82 (d, *J* = 8.6 Hz, 1H), 7.70 (d, *J* = 8.6 Hz, 1H), 3.68 – 3.64 (m, 2H), 3.55 – 3.50 (m, 2H), 2.57 (t, *J* = 7.2 Hz, 2H), 2.16 (t, *J* = 7.2 Hz, 2H), 1.83 (p, *J* = 7.2 Hz, 2H), 1.47 (dt, *J* = 15.2, 7.2 Hz, 4H), 1.30 (p, *J* = 7.4 Hz, 2H). **^{13}C NMR** (101 MHz, DMSO-*d*$_6$) δ 174.8, 159.5, 152.1, 143.7, 134.4, 131.1, 130.8, 130.0, 126.7, 122.5, 122.0, 118.7, 118.4, 53.6, 53.1, 48.8, 33.8, 26.0, 25.7, 24.4, 22.5. **HRMS** m/z (ESI):

calcd. for $C_{21}H_{26}N_3O_7S$ $[M+H]^+$: 464.1491; found:464.1492.

12. Preparation of BSA conjugates

To a penicillin bottle was added bovine serum albumin (1 mg, 1.5×10^{-5} mmol), dissolved with 0.1M $NaHCO_3$ (0.1mL), followed by slowly adding **LM-02** (1.8×10^{-4} mmol) or **LM-03-NHS** (1.8×10^{-4} mmol). The reactions were incubated on a tilt shaker at 25 °C for one hour. After the indicated reaction time, the solutions were transferred onto 0.5 mL Zeba desalting column (7000 molecular weight cutoff) and de-salted with 0.01M PBS using a bench top centrifuge (5804R, Eppendorf). The final concentrated solutions were transferred into amber glass vials with 0.01M PBS (0.1mL), **LM-2-BSA** or **LM-3-BSA** was obtained with a concentration around 1.5×10^{-4} M.

Chemiluminescence Measurements.

The chemiluminescence in this study was measured on MAGLUMI® X3 (Fig. S1), oxidation system of X3 contains two reagents, A and B. We had the standard oxidation condition for most examples while as supplementary the diluted oxidation condition was prepared for cases react too fast (Fig. S2). A simplified chemiluminescence reaction pathway of isoluminol derivatives was shown in Fig. S2, an initial treatment with reagent A converts the dianion intermediate, which will be further oxidized to an excited state compound, light emits when excited state goes back to ground state. In this system, hydrogen peroxide itself cannot oxidize the dianion intermediate, it's a precursor of real oxidant which will be released with catalyst.

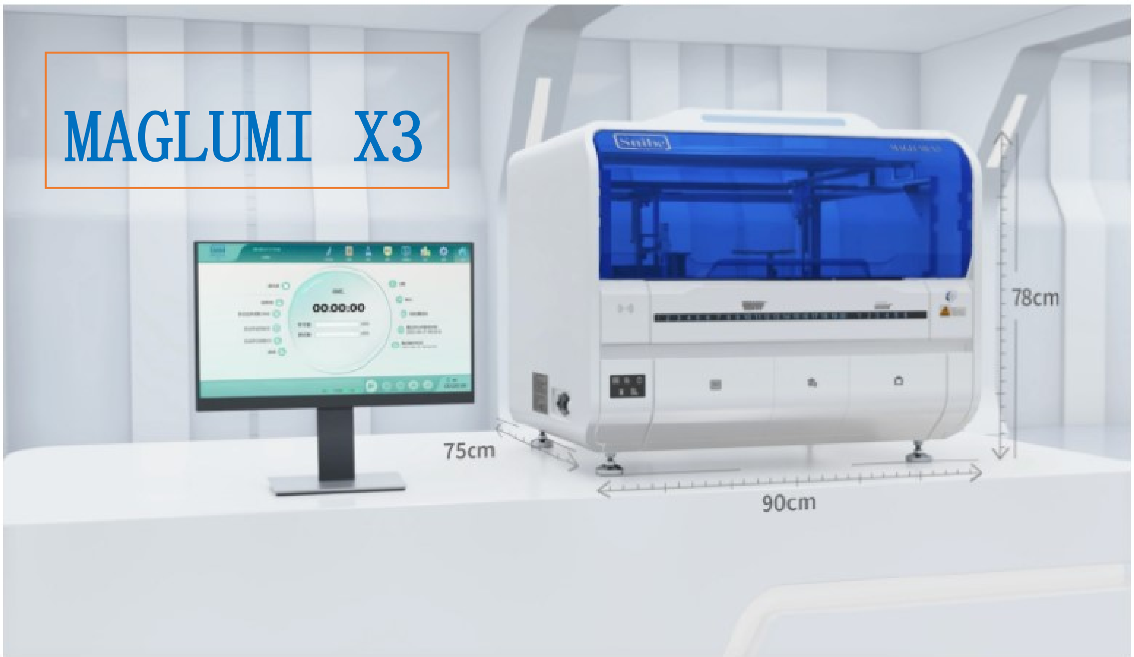

Fig. S1 The MAGLUMI® X3 chemiluminescence immunoassay (CLIA) system, developed by Snibe (Shenzhen New Industries Biomedical Engineering Co., Ltd.) Diagnostic.

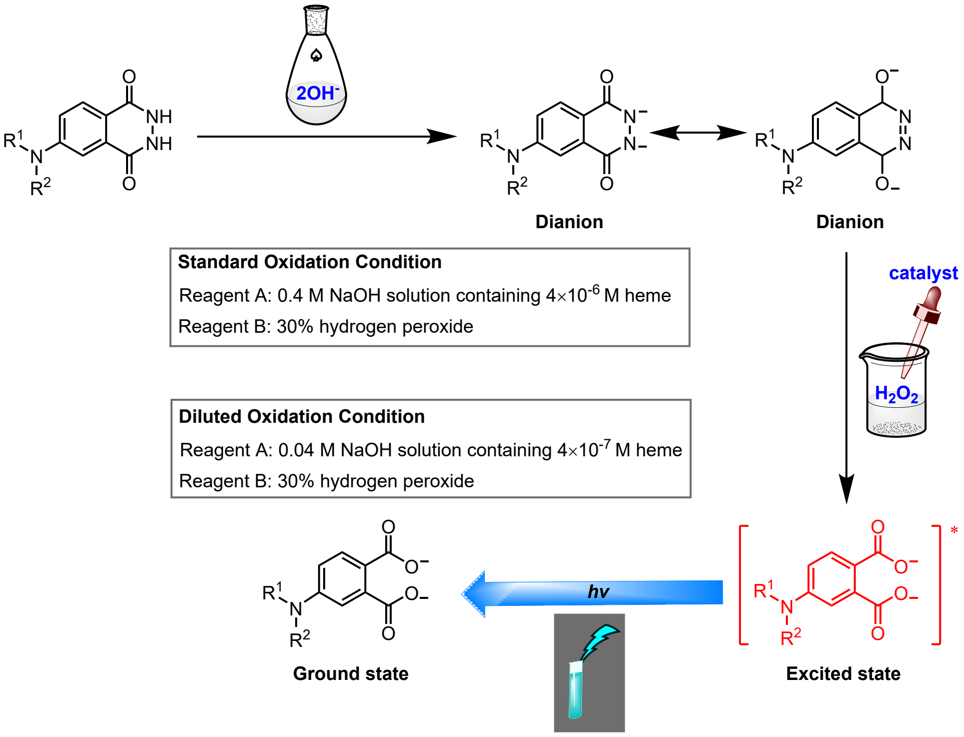

Fig. S2 Simplified chemiluminescence reaction pathway of isoluminol derivatives.

General procedure for the measurement of chemiluminescence efficiency (using ABEI as an example):

To ABEI (2.76 mg, 0.01mmol) was added DMF (10 mL) to get a 1.0 mM solution, which was then serially diluted to 1×10^{-8} M with purified water for measurements. Emission from 10μL samples were measured on MAGLUMI® X3 Chemiluminescence Immunoassay (CLIA) System through the addition of 200 μL 0.4 M NaOH solution containing 4×10^{-6} M heme followed by injecting 200 μL 30% hydrogen peroxide. Light was collected for a total of 3 seconds integrated at 0.5 second intervals. The output of the luminometer was in RLUs (Relative Light Units).

Table S1 Chemiluminescence of **Luminol**, **ABEI**, **LM-1**, **LM-2** and **LM-3**.[a]

Point in time (s)	Compounds and relative light units				
	**Luminol**	**ABEI**	**LM-1**	**LM-2**	**LM-3**
0.5	380864	938276	993976	958662	1025214
1.0	35168	380502	499359	515453	501271
1.5	5579	156157	240835	251376	234206
2.0	1497	78590	136926	137136	130353
2.5	625	44052	84801	81864	79177
3.0	311	26929	55445	51559	51381
Total of 3 secs	424044	1624506	2011342	1996050	2021602
Relative CL intensity	100	383	474	471	477

[a] Follow general procedure with the standard oxidation condition.

Table S2 Chemiluminescence of **ABEN**, **LM-4**, **DA-ASPH** and **LM-5**.[a]

Point in time (s)	Compounds and relative light units							
	**ABEN**		**LM-4**		**DA-ASPH**		**LM-5**	
0.5	713372	938985	1118447	826271	333007	294077	485246	481562
1.0	38629	353039	19977	427090	55179	117126	41690	169583
1.5	14710	117343	740	193801	26719	55132	17161	69210
2.0	8346	53611	478	99237	16605	32298	9953	38153
2.5	5338	30326	360	56084	11282	20711	6528	24051
3.0	3639	19442	305	33982	8070	14173	4493	16506
Total of 3 secs	784034	1512746	1140307	1636465	450862	533517[b]	565071	799065
Relative CL intensity	185	357	269	386	106	126[b]	133	188

[a] Follow general procedure with the standard oxidation condition. [b]Follow general procedure with the diluted oxidation condition.

Table S3 Chemiluminescence of **LM-6**, **LM-7**, **LM-8, LM-9**, **LM-10** and **LM-11**.[a]

Point in time (s)	Compounds and relative light units						
	**LM-6**	**LM-7**	**LM-8**	**LM-9**	**LM-10**	**LM-11**	
0.5	103271	9131	757370	789109	955749	1204807	1083456
1.0	38437	1976	398821	392245	525750	81591	763298
1.5	18770	888	208326	204457	286091	6186	457416
2.0	11400	515	128064	125320	179086	2753	290443
2.5	7418	340	85067	83049	120638	1568	191961
3.0	5250	240	59511	57711	84560	1041	131907
Total of 3 secs	184546	13090	1637159	1651891	2151874	1297946	2918481[b]
Relative CL intensity	44	3	386	390	508	306	688 [b]

[a] Follow general procedure with the standard oxidation condition. [b]Follow general procedure with the diluted oxidation condition.

Table S4 Chemiluminescence of **LM-2-BSA** and **LM-3-BSA**.[a]

Point in time (s)	Compounds and relative light units			
	**LM-2-BSA**		**LM-3-BSA**	
0.5	265150	193054	436803	314657
1.0	159884	129472	318274	258169
1.5	106013	80973	223411	195147
2.0	79453	52722	174478	154481
2.5	63180	41181	142794	99574
3.0	51839	33321	120270	82228
Total of 3 secs	725519	530723 [b]	1416030	1104256 [b]
Relative CL intensity	171	125 [b]	334	260 [b]

[a] **LM-2-BSA** or **LM-3-BSA** was serially diluted to 1.5×10^{-9} M, 10μL samples were measured on MAGLUMI® X3, others follow general procedure with the standard oxidation condition. [b]Samples kept at 4 °C for 10 days.

Labelling Efficacy Measurements

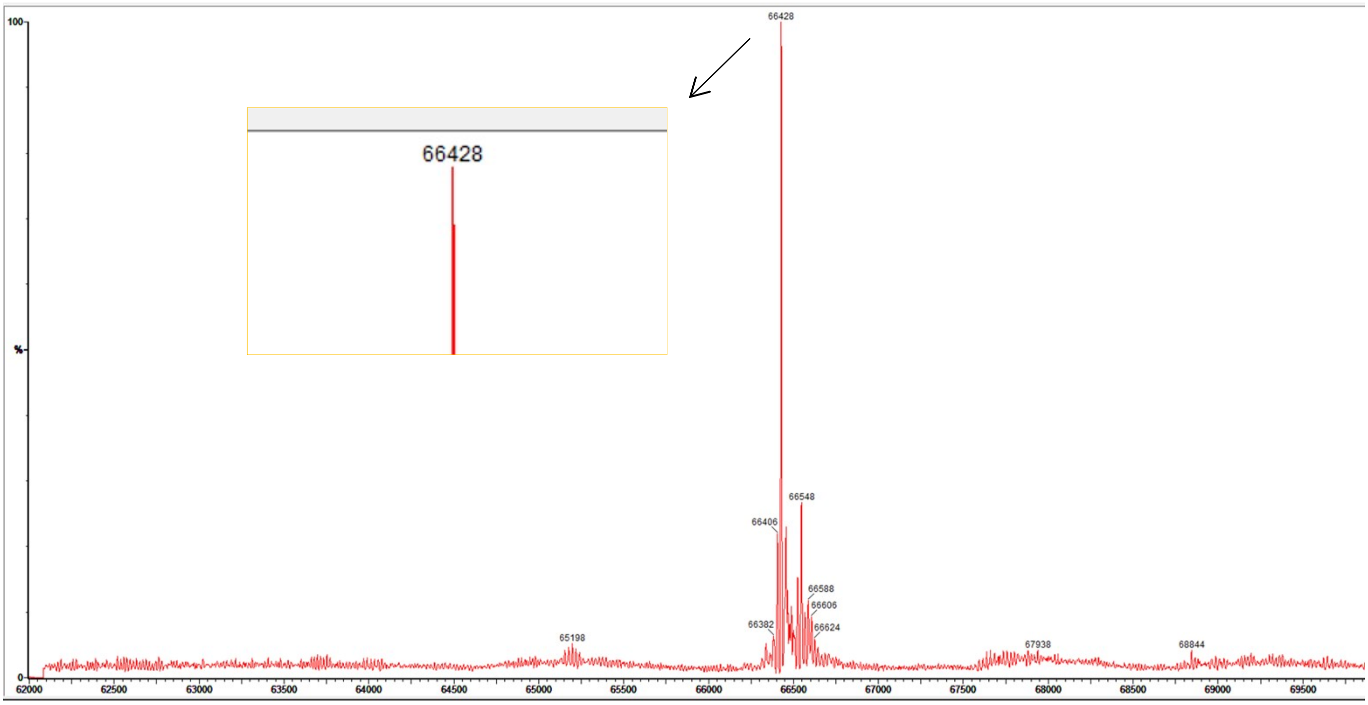

Fig. S3 Mass spectrum of **BSA**.

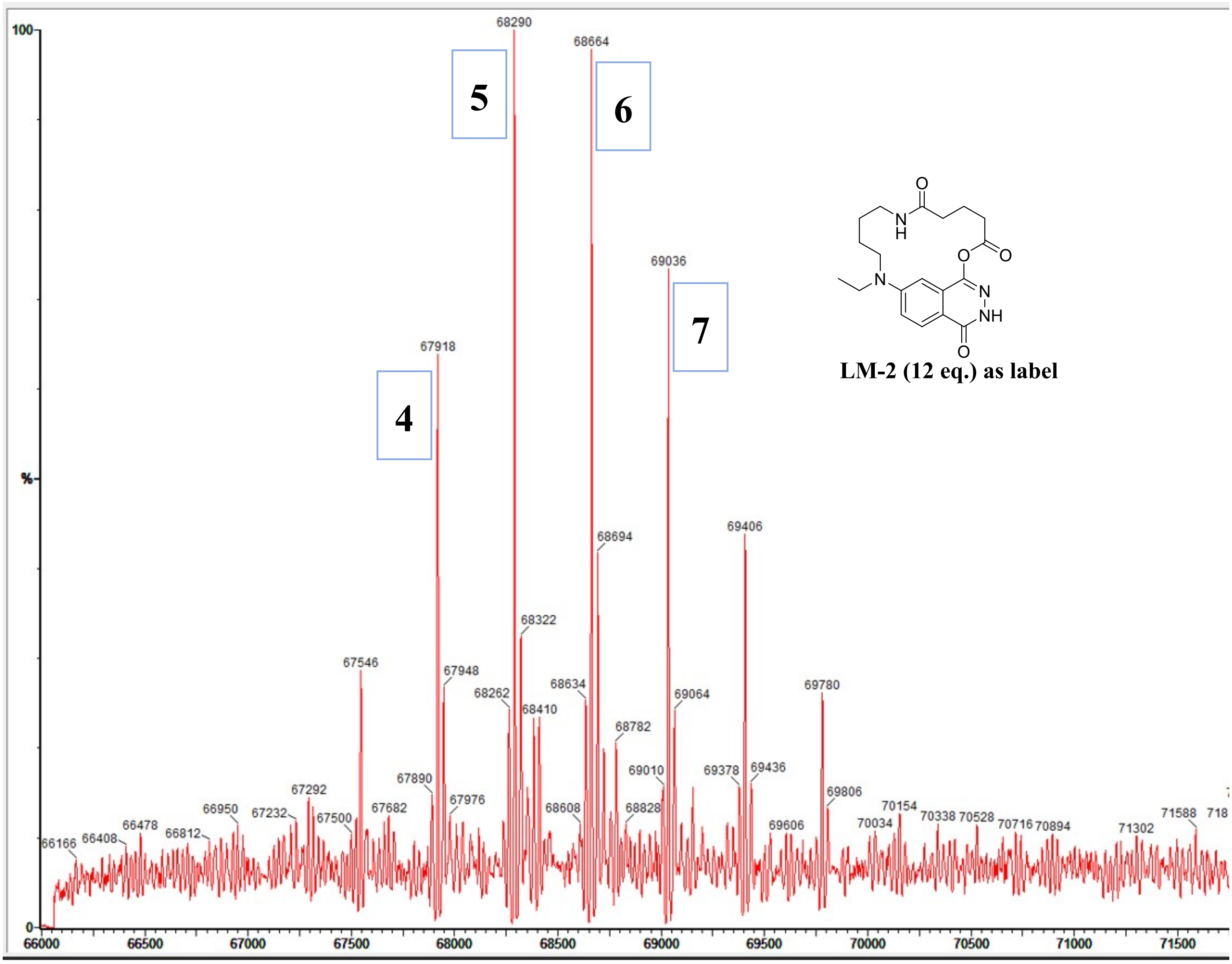

Fig. S4 Mass spectrum of **LM-2-BSA**.

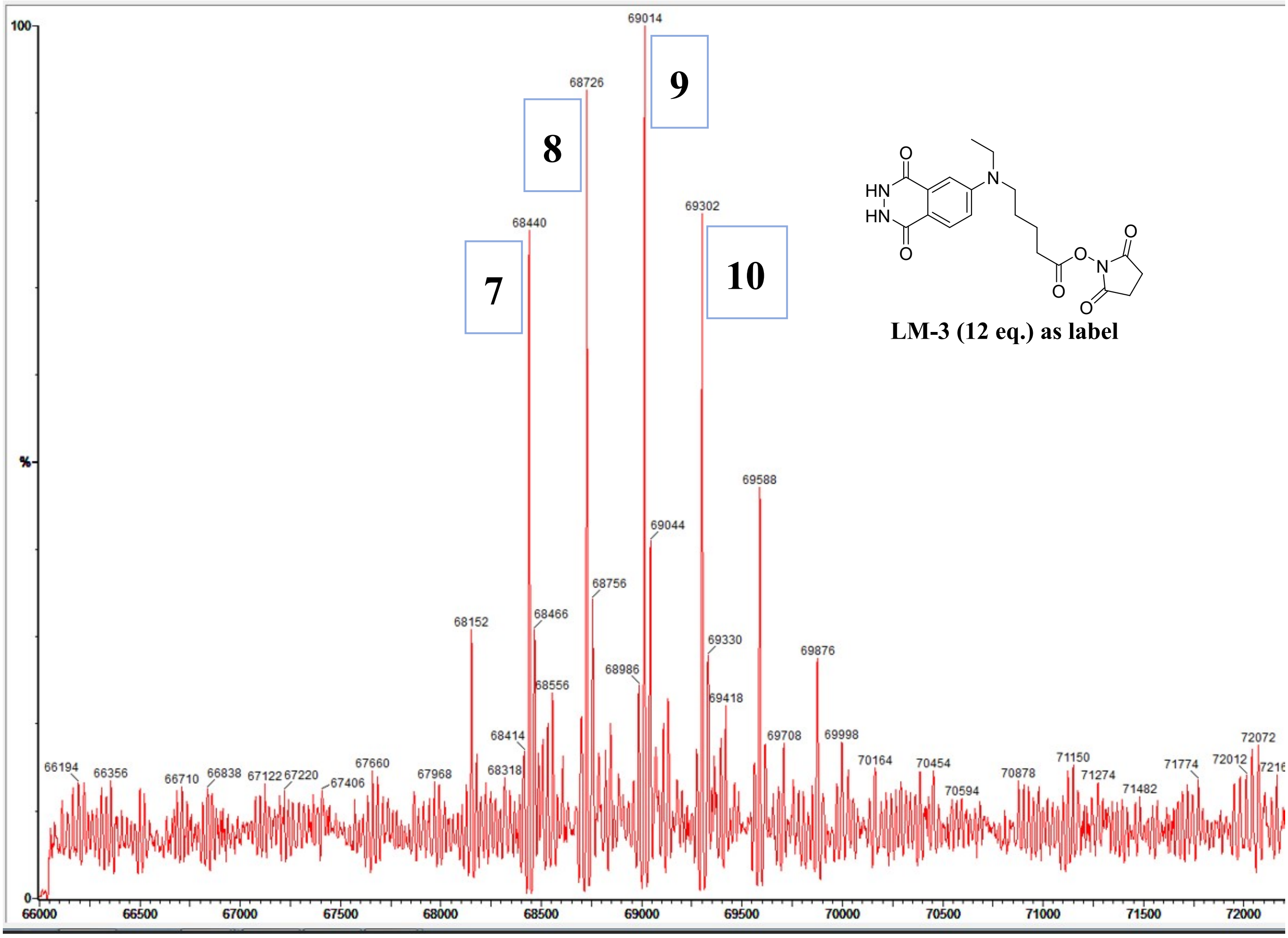

Fig. S5 Mass spectrum of **LM-3-BSA**.

Fractional Non-Specific Binding Measurements

Procedure for the measurement of fNSB:

To a 2.0 mL EP tube was added 1×10^{-8} M **LM-n (n=2,3,10,4,11)** 400 μL, followed by the addition of 100 μL magnetic microparticles MX 100 (solid content in slurry 2%) or MS 160 (solid content in slurry 1%), the mixture was incubated on a tilt shaker at 25 degree for 10 min. 10 μL samples were sucked up with pipette for measurements on MAGLUMI® X3 followed the general procedure which gained results of group A. The tube was then kept stationary for 10 min, a magnet was used to gather and fix magnetic microparticles before carefully sucking away the dispersion liquid. To the residual sample was added 500 μL purified water and well mixed, 10 μL samples were sucked up for chemiluminescence measurements which gained results of group B. For both group, the total relative light units of 3 secs were calculated, the ratio of B to A gave fNSB.

Table S5 Chemiluminescence and fNSB of **LM-2, LM-3, LM-10, LM-4**, **LM-11** with MX 100.

Point in time (s)	Compounds and relative light units / MX 100 (Carboxyl surface)									
	**LM-2**		**LM-3**		**LM-10**		**LM-4**		**LM-11**	
	A	B	A	B	A	B	A	B	A	B
0.5	577956	457313	753767	532585	843344	256663	671996	221948	960362	107412
1.0	233264	97027	330162	54482	585838	159119	236379	82859	634278	51805
1.5	112627	49491	163087	20548	395419	104678	82210	30693	366039	25472
2.0	65678	32331	96299	12414	300018	78819	36368	14574	228216	15642
2.5	42127	22943	62470	8270	239994	62958	19082	8189	150846	10623
3.0	28426	16915	42629	5840	197390	51682	11441	5080	104054	7642
Total of 3 secs	1060078	676020	1448414	634139	2562003	713919	1057476	363343	2443795	218596
fNSB	0.64		0.44		0.28		0.34		0.09	

Table S6 Chemiluminescence and fNSB of **LM-2, LM-3, LM-10, LM-4**, **LM-11** with MS 160.

Point in time (s)	Compounds and relative light units / MS 160 (tosyl surface)									
	**LM-2**		**LM-3**		**LM-10**		**LM-4**		**LM-11**	
	A	B	A	B	A	B	A	B	A	B
0.5	760029	532235	861164	809189	1023480	292008	487207	277469	917196	142728
1.0	305135	68946	363098	258351	696260	55694	174918	119613	605216	86287
1.5	149478	30420	181199	132236	481770	31162	65513	50504	350043	46457
2.0	88395	17913	108228	82423	370321	21608	31937	26430	216000	29144
2.5	57224	11876	70662	55919	298560	16203	18824	15969	140422	19855
3.0	39405	8371	48971	40225	247316	12842	12615	10651	95086	14036
Total of 3 secs	1399666	669761	1633322	1378343	3117707	429517	791014	500636	2323963	338507
fNSB	0.48		0.84		0.14		0.63		0.15	

References

1 H. Yoshida, R. Nakao, H. Nohta and M. Yamaguchi, *Dyes Pigm.*, 2000, **47**, 239–245.

Spectra

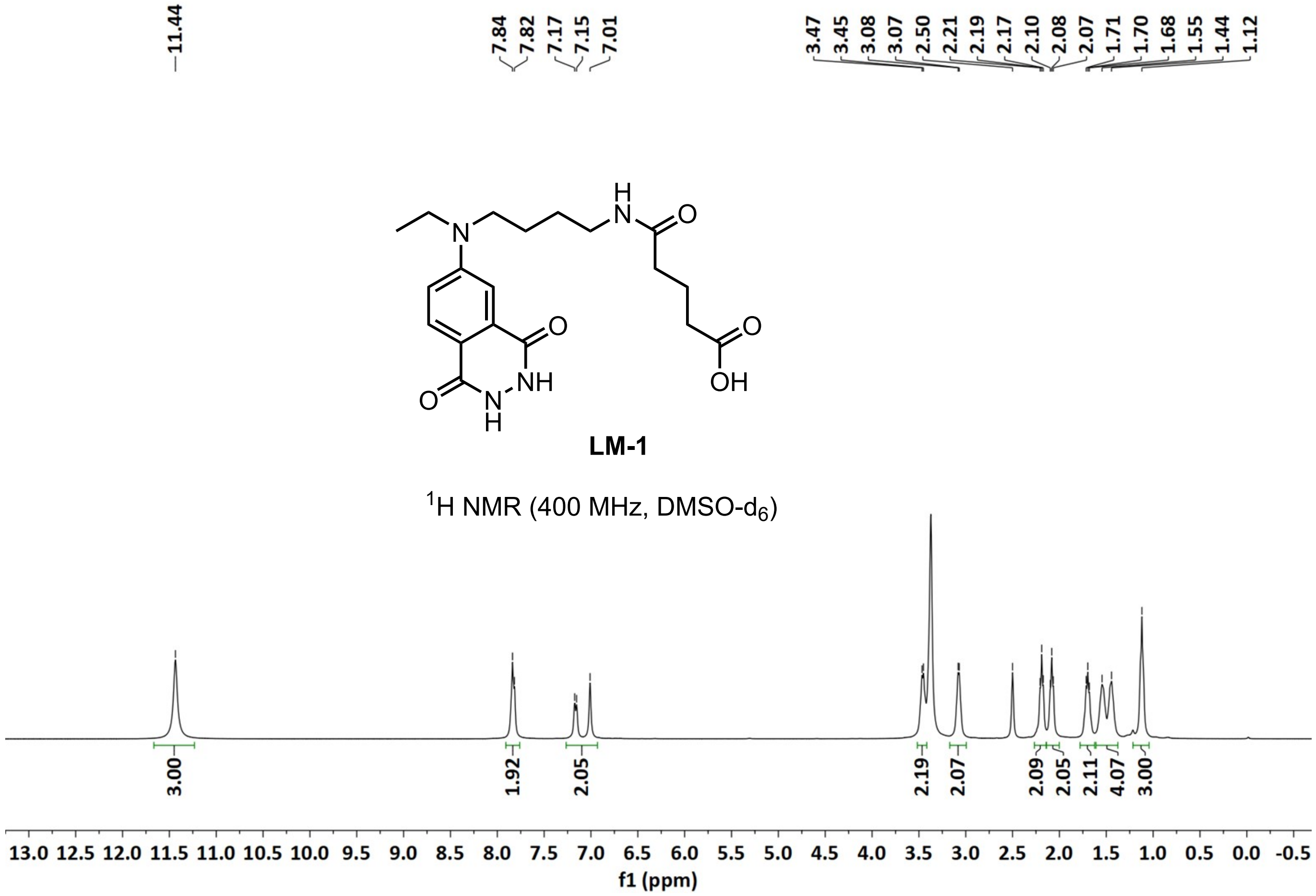
11.44
7.84
7.82
7.17
7.15
7.01
3.47
3.45
3.08
3.07
2.50
2.21
2.19
2.17
2.10
2.08
2.07
1.71
1.70
1.68
1.55
1.44
1.12
LM-1
^{1}H NMR (400 MHz, DMSO-d$_6$)
3.00
1.92
2.05
2.19
2.07
2.09
2.05
2.11
4.07
3.00
13.0 12.5 12.0 11.5 11.0 10.5 10.0 9.5 9.0 8.5 8.0 7.5 7.0 6.5 6.0 5.5 5.0 4.5 4.0 3.5 3.0 2.5 2.0 1.5 1.0 0.5 0.0 -0.5
f1 (ppm)

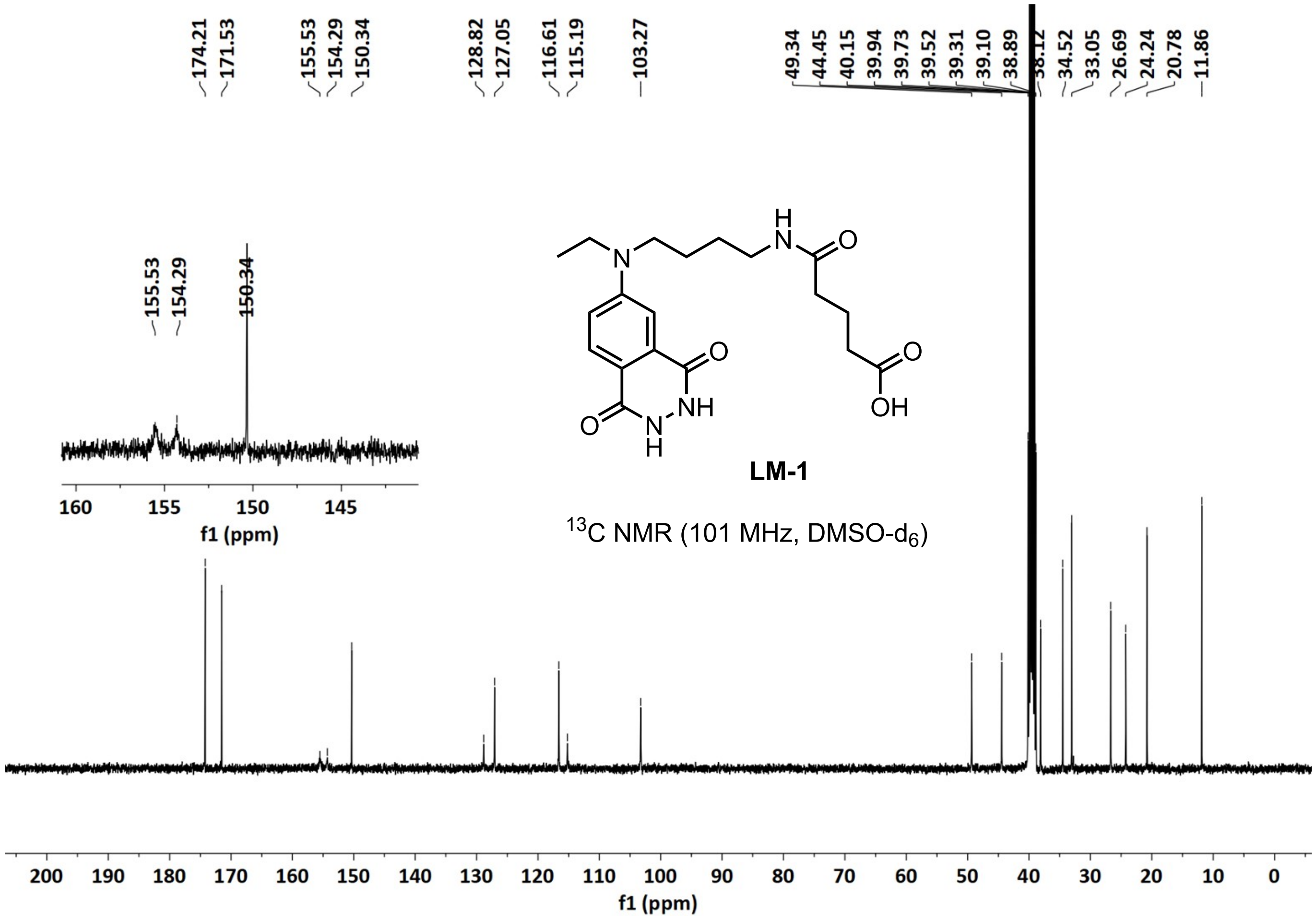
174.21
171.53
155.53
154.29
150.34
128.82
127.05
116.61
115.19
103.27
49.34
44.45
40.15
39.94
39.73
39.52
39.31
39.10
38.89
38.12
34.52
33.05
26.69
24.24
20.78
11.86
160 155 150 145
f1 (ppm)
LM-1
^{13}C NMR (101 MHz, DMSO-d$_6$)
200 190 180 170 160 150 140 130 120 110 100 90 80 70 60 50 40 30 20 10 0
f1 (ppm)

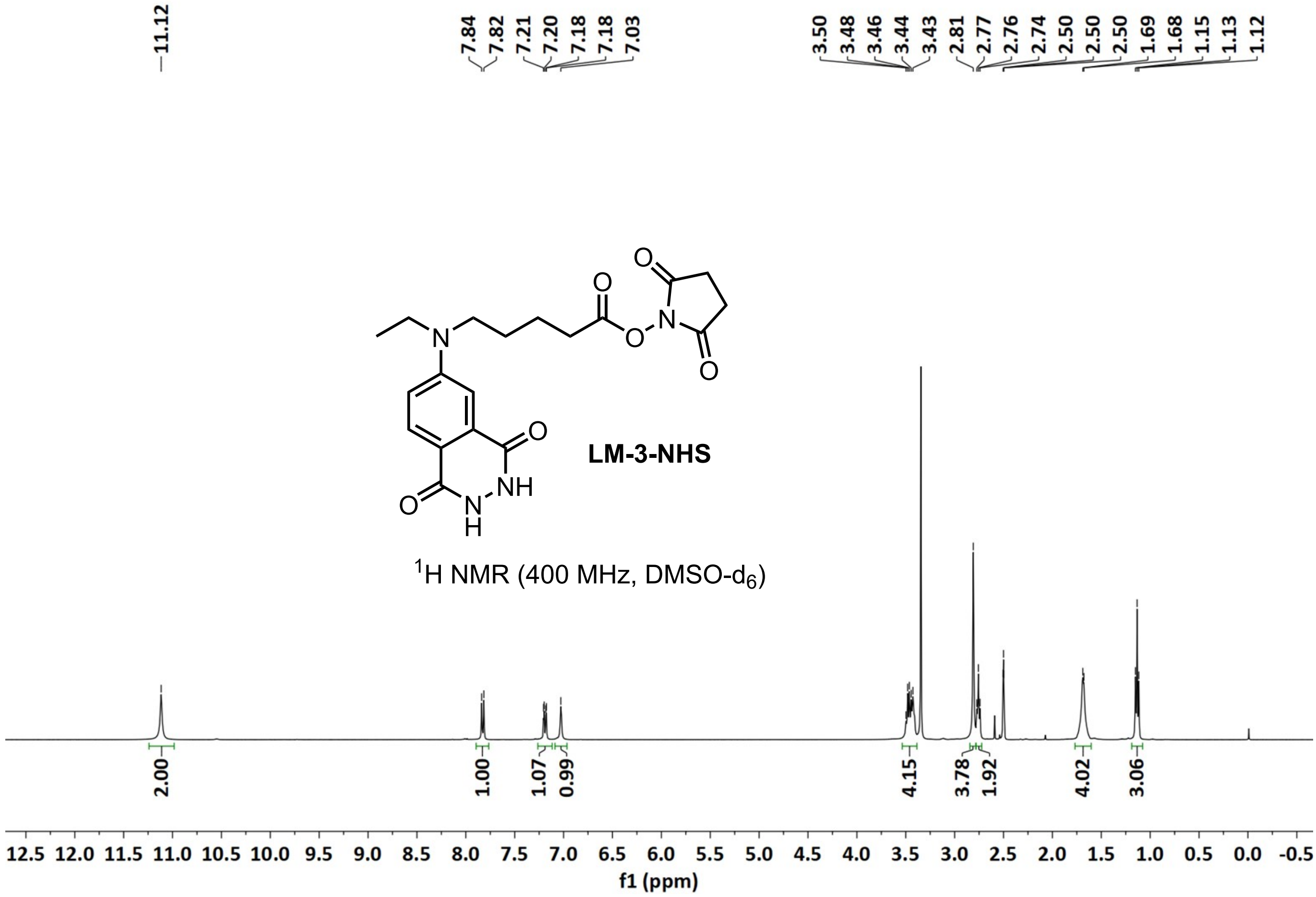

11.12
7.84 7.82 7.21 7.20 7.18 7.18 7.03
3.50 3.48 3.46 3.44 3.43 2.81 2.77 2.76 2.74 2.50 2.50 2.50 1.69 1.68 1.15 1.13 1.12
LM-3-NHS
^{1}H NMR (400 MHz, DMSO-d_6)
2.00 1.00 1.07 0.99 4.15 3.78 1.92 4.02 3.06
12.5 12.0 11.5 11.0 10.5 10.0 9.5 9.0 8.5 8.0 7.5 7.0 6.5 6.0 5.5 5.0 4.5 4.0 3.5 3.0 2.5 2.0 1.5 1.0 0.5 0.0 -0.5
f1 (ppm)

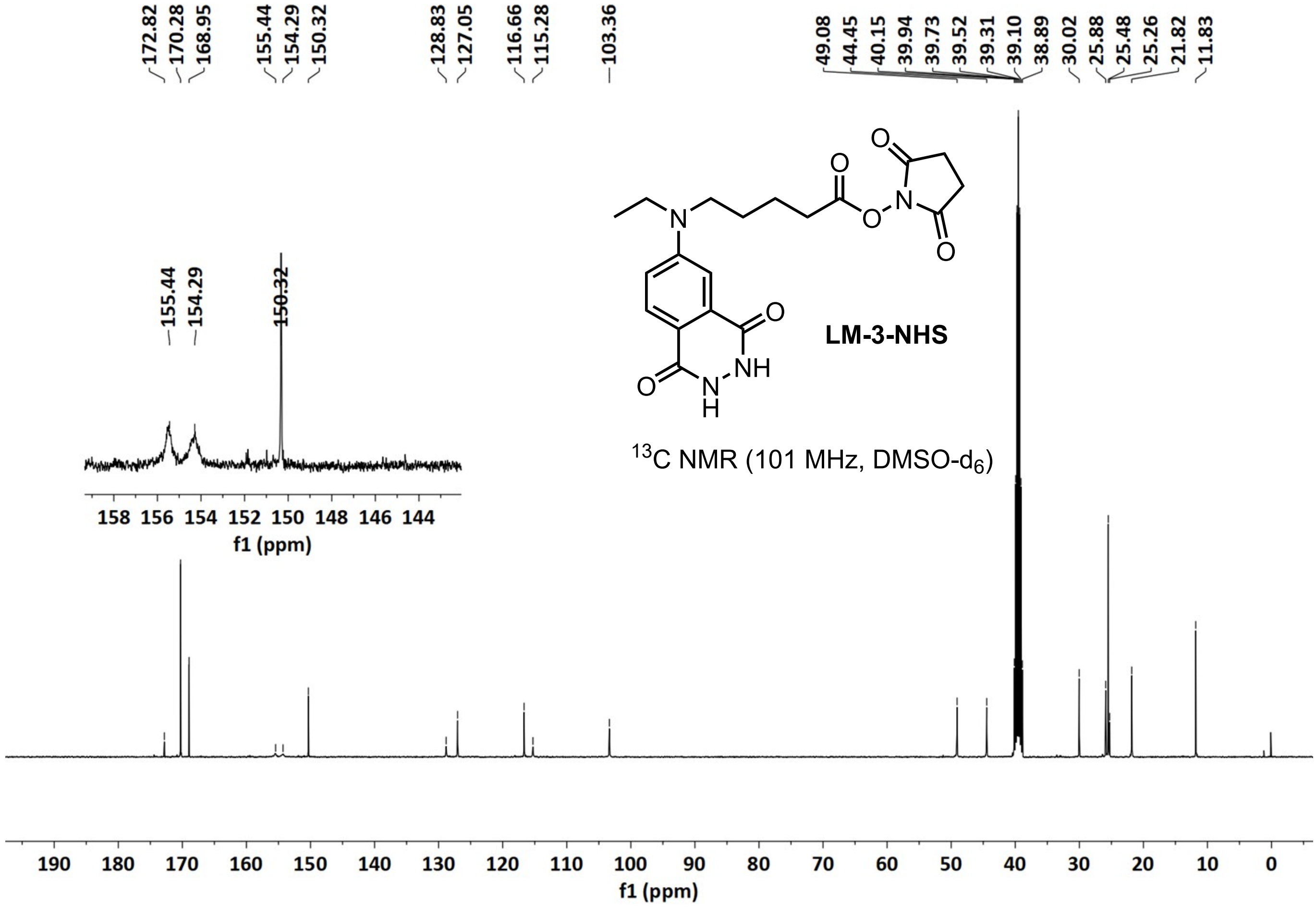

172.82 170.28 168.95 155.44 154.29 150.32 128.83 127.05 116.66 115.28 103.36
49.08 44.45 40.15 39.94 39.73 39.52 39.31 39.10 38.89 30.02 25.88 25.48 25.26 21.82 11.83
155.44 154.29 150.32
158 156 154 152 150 148 146 144
f1 (ppm)
LM-3-NHS
^{13}C NMR (101 MHz, DMSO-d_6)
190 180 170 160 150 140 130 120 110 100 90 80 70 60 50 40 30 20 10 0
f1 (ppm)

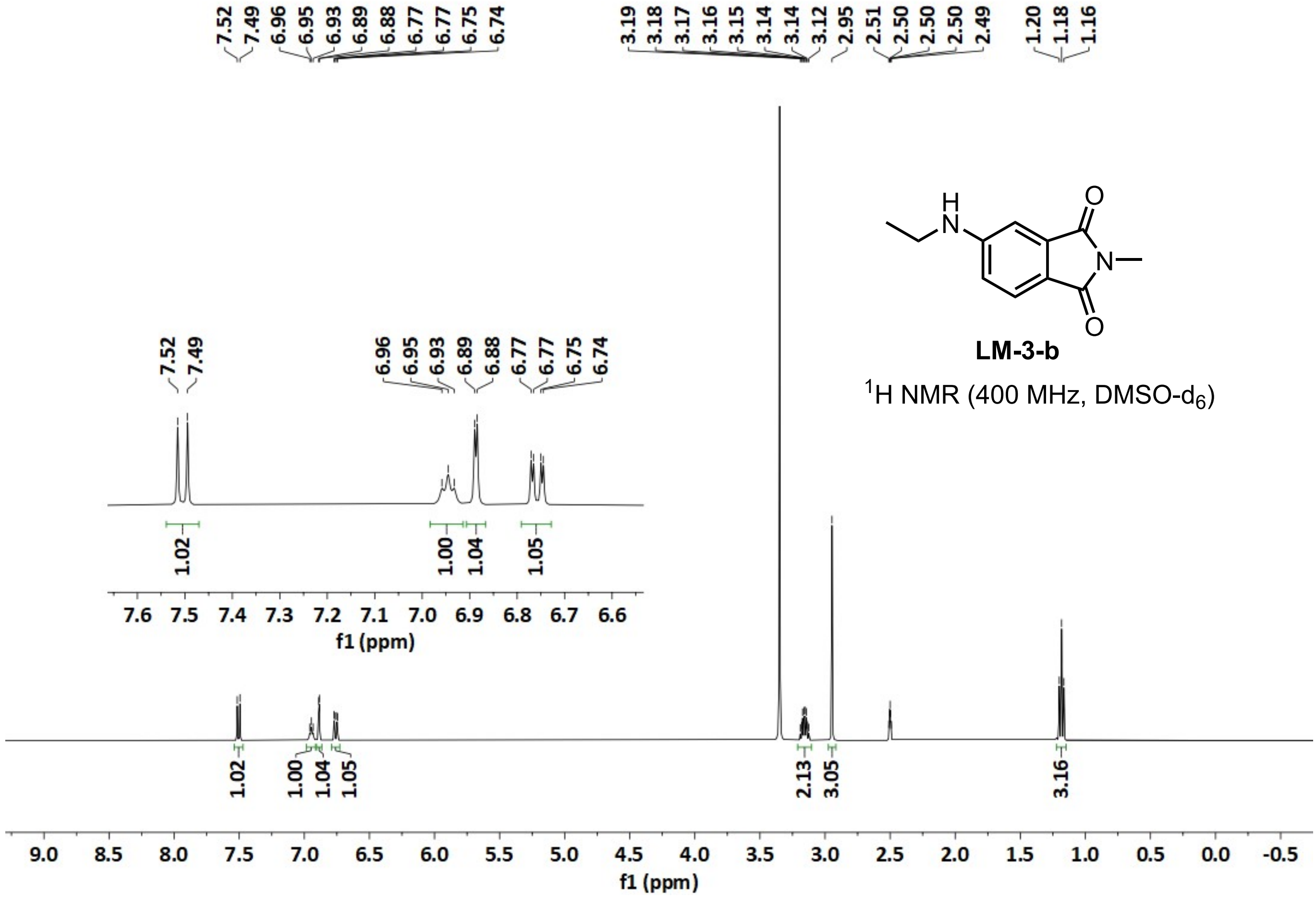
7.52
7.49
6.96
6.95
6.93
6.89
6.88
6.77
6.77
6.75
6.74
3.19
3.18
3.17
3.16
3.15
3.14
3.14
3.12
2.95
2.51
2.50
2.50
2.50
2.49
1.20
1.18
1.16
LM-3-b
^{1}H NMR (400 MHz, DMSO-d$_6$)
1.02
1.00
1.04
1.05
2.13
3.05
3.16
f1 (ppm)

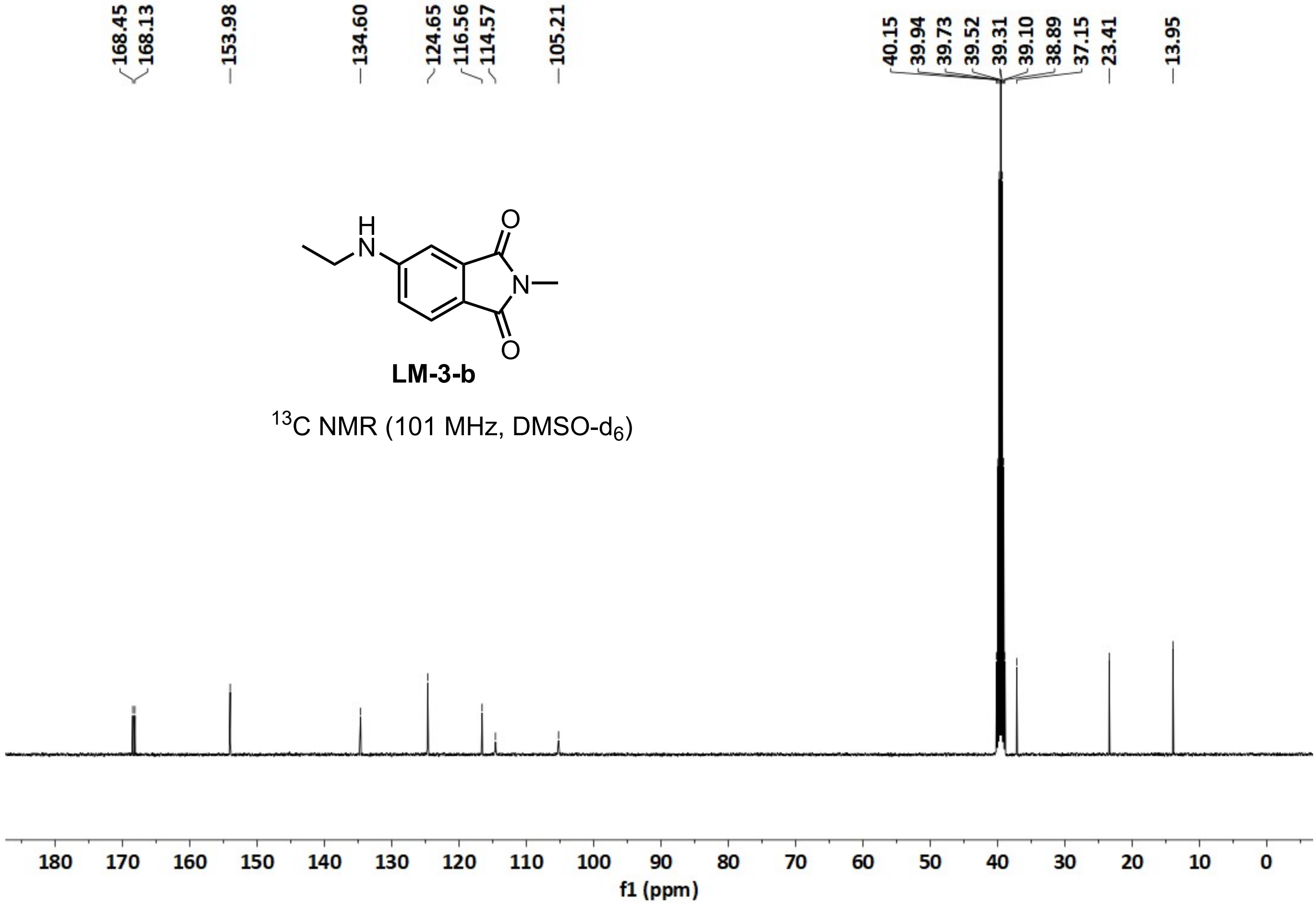
168.45
168.13
153.98
134.60
124.65
116.56
114.57
105.21
40.15
39.94
39.73
39.52
39.31
39.10
38.89
37.15
23.41
13.95
LM-3-b
^{13}C NMR (101 MHz, DMSO-d$_6$)
f1 (ppm)

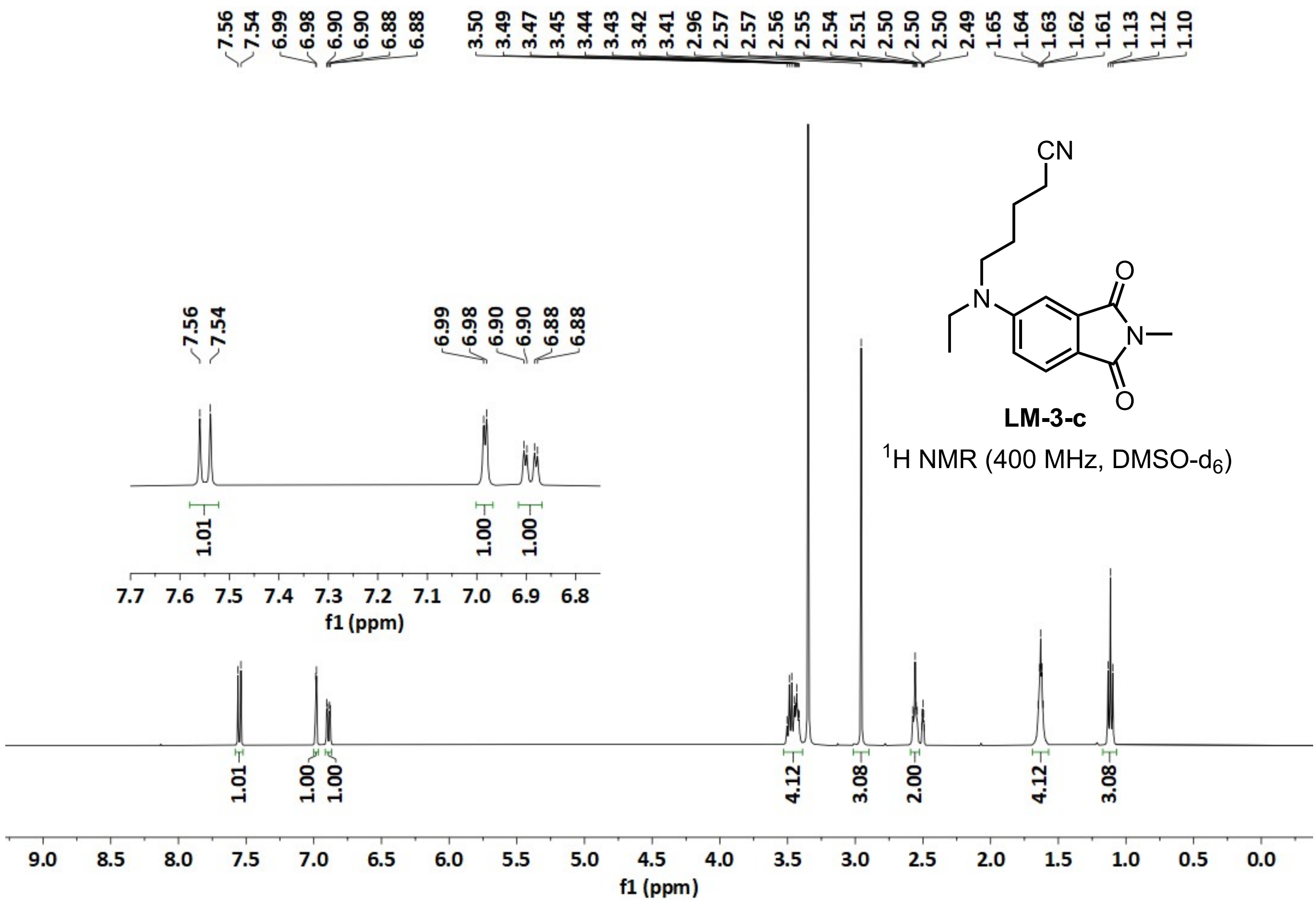
7.56 7.54 6.99 6.98 6.90 6.90 6.88 6.88 3.50 3.49 3.47 3.45 3.44 3.43 3.42 3.41 2.96 2.57 2.57 2.56 2.55 2.54 2.51 2.50 2.50 2.50 2.49 1.65 1.64 1.63 1.62 1.61 1.13 1.12 1.10
CN
N
O
N
O
LM-3-c
^{1}H NMR (400 MHz, DMSO-d$_6$)
1.01
1.00
1.00
4.12
3.08
2.00
4.12
3.08
f1 (ppm)

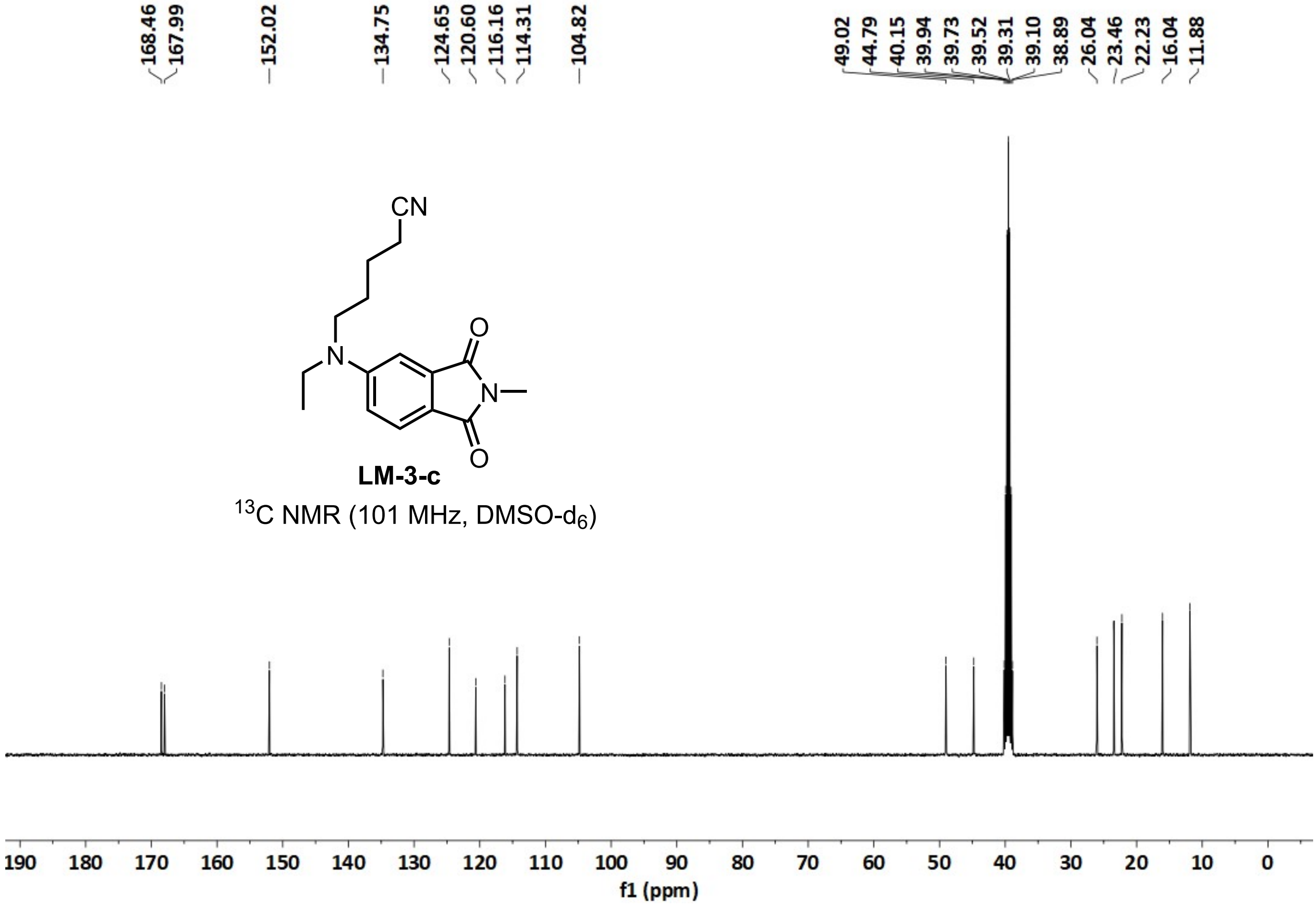
168.46 167.99 152.02 134.75 124.65 120.60 116.16 114.31 104.82
49.02 44.79 40.15 39.94 39.73 39.52 39.31 39.10 38.89 26.04 23.46 22.23 16.04 11.88
CN
N
O
N
O
LM-3-c
^{13}C NMR (101 MHz, DMSO-d$_6$)
f1 (ppm)

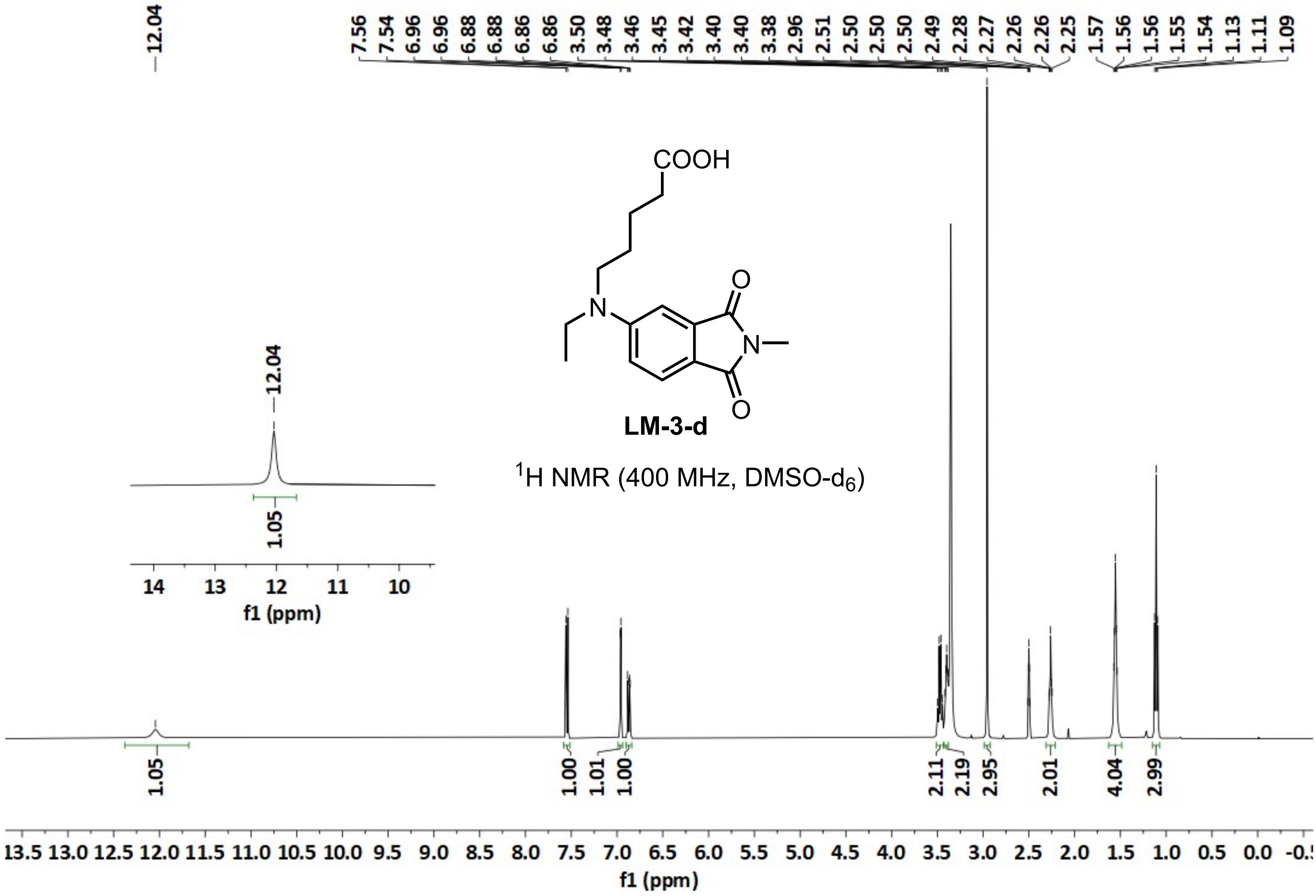
12.04
7.56 7.54 6.96 6.96 6.88 6.88 6.86 6.86 3.50 3.48 3.46 3.45 3.42 3.40 3.40 3.38 2.96 2.51 2.50 2.50 2.50 2.49 2.28 2.27 2.26 2.26 2.25 1.57 1.56 1.56 1.55 1.54 1.13 1.11 1.09
COOH
N
O
N
O
LM-3-d
¹H NMR (400 MHz, DMSO-d6)
1.05
1.00 1.01 1.00
2.11 2.19 2.95 2.01 4.04 2.99
f1 (ppm)

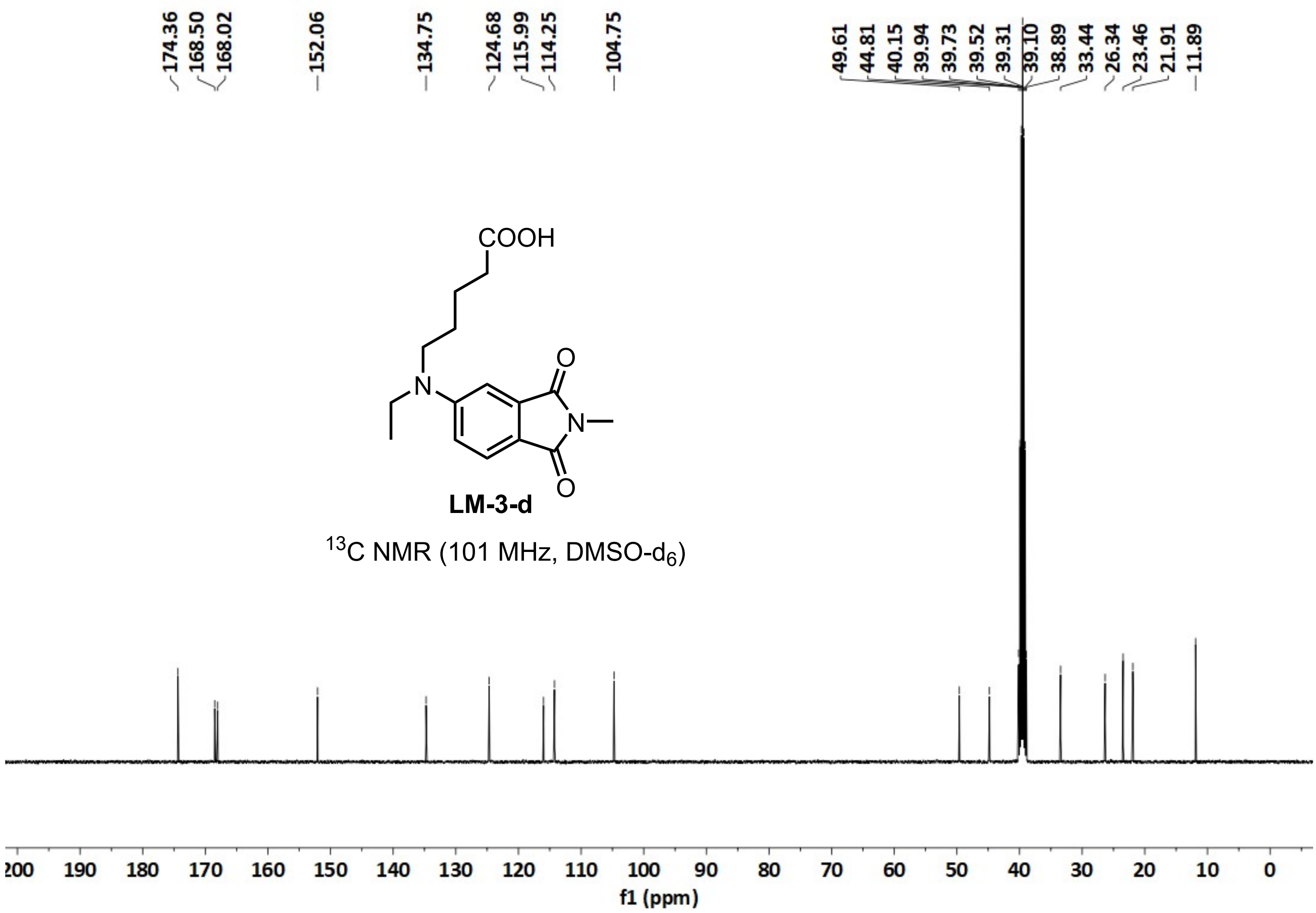
174.36 168.50 168.02 152.06 134.75 124.68 115.99 114.25 104.75
49.61 44.81 40.15 39.94 39.73 39.52 39.31 39.10 38.89 33.44 26.34 23.46 21.91 11.89
COOH
N
O
N
O
LM-3-d
¹³C NMR (101 MHz, DMSO-d6)
f1 (ppm)

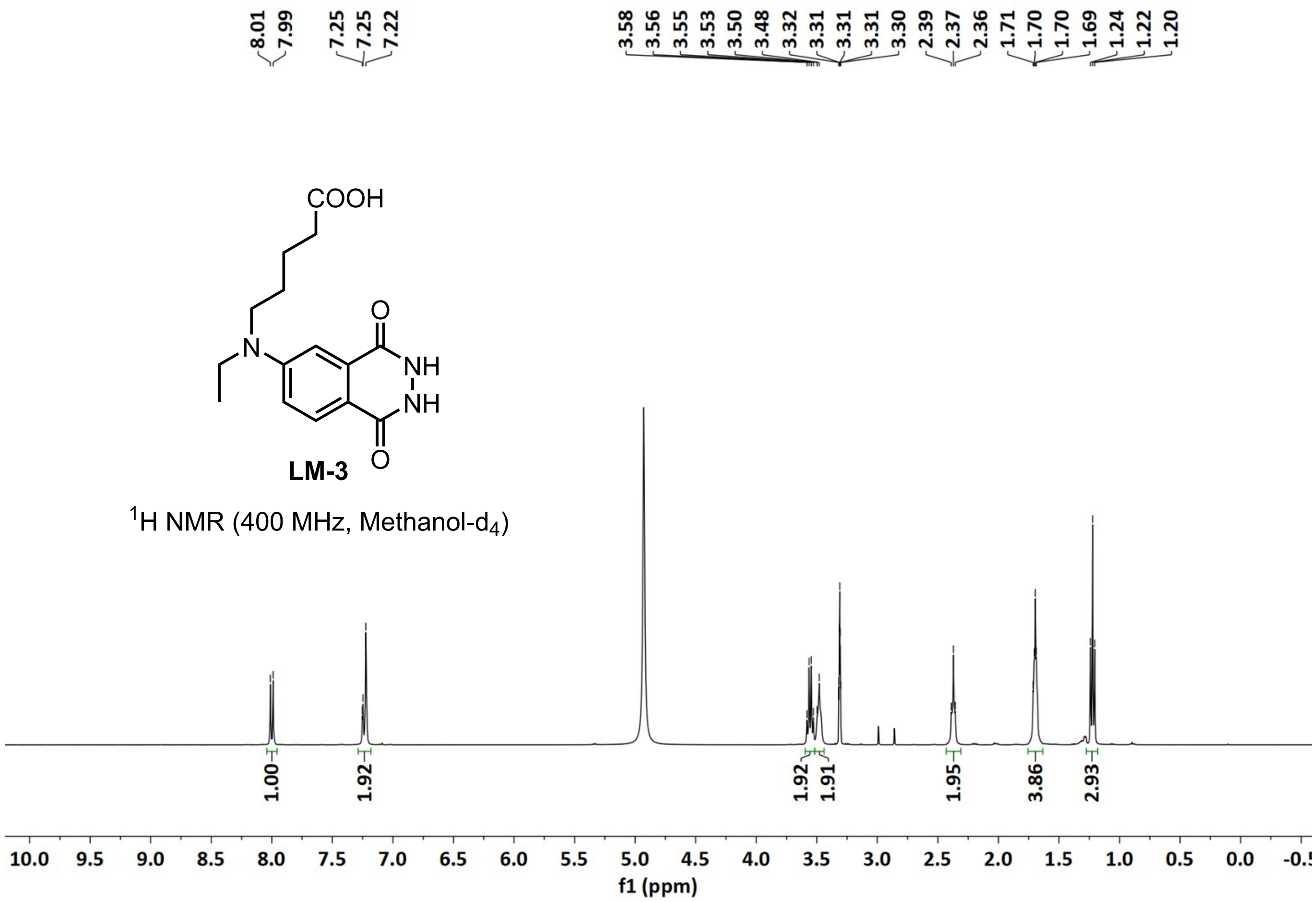
8.01
7.99
7.25
7.25
7.22
3.58
3.56
3.55
3.53
3.50
3.48
3.32
3.31
3.31
3.31
3.30
2.39
2.37
2.36
1.71
1.70
1.70
1.69
1.24
1.22
1.20
COOH
O
N
NH
NH
O
LM-3
^{1}H NMR (400 MHz, Methanol-d$_4$)
1.00
1.92
1.92
1.91
1.95
3.86
2.93
f1 (ppm)

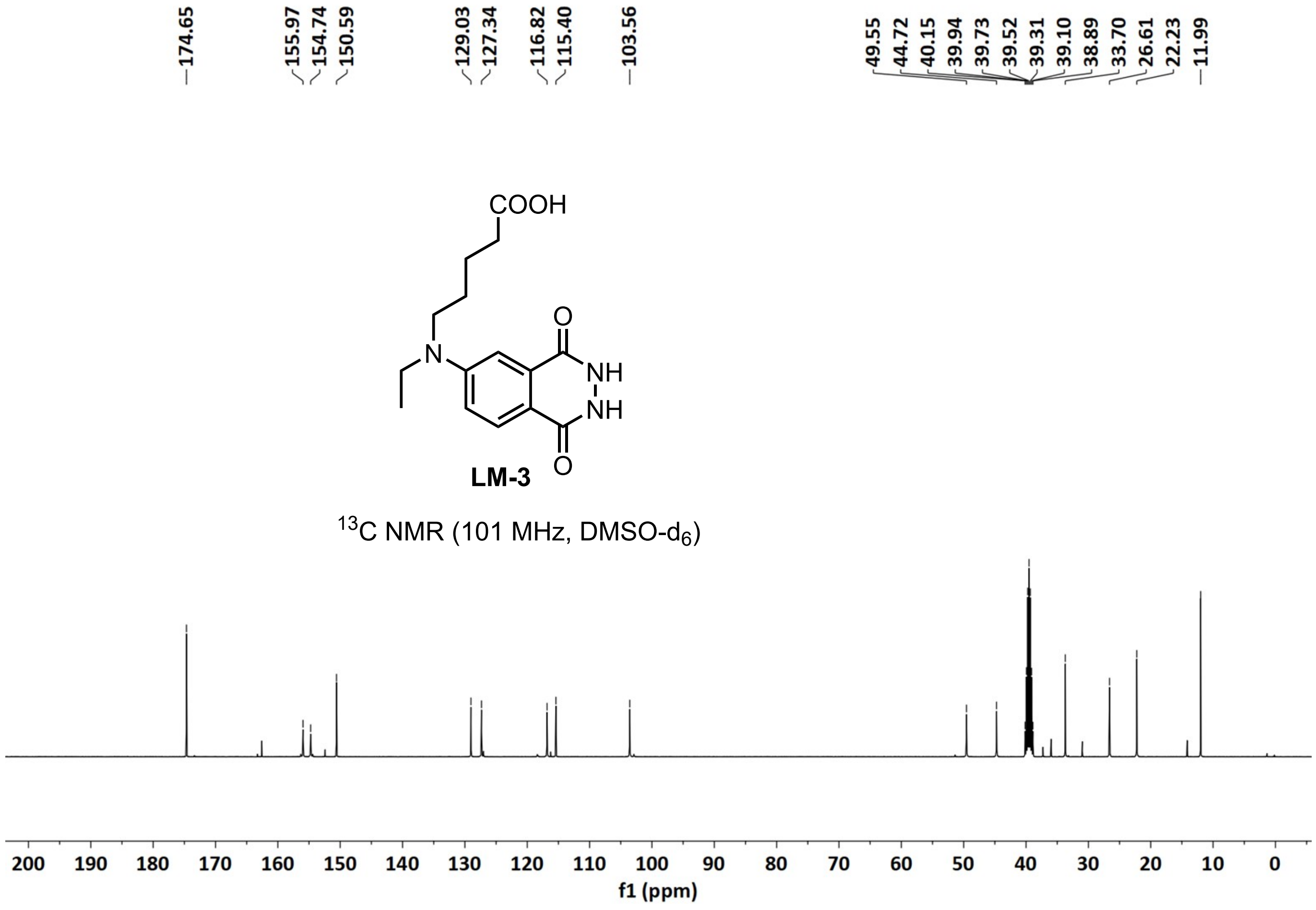
174.65
155.97
154.74
150.59
129.03
127.34
116.82
115.40
103.56
49.55
44.72
40.15
39.94
39.73
39.52
39.31
39.10
38.89
33.70
26.61
22.23
11.99
COOH
O
N
NH
NH
O
LM-3
^{13}C NMR (101 MHz, DMSO-d$_6$)
f1 (ppm)

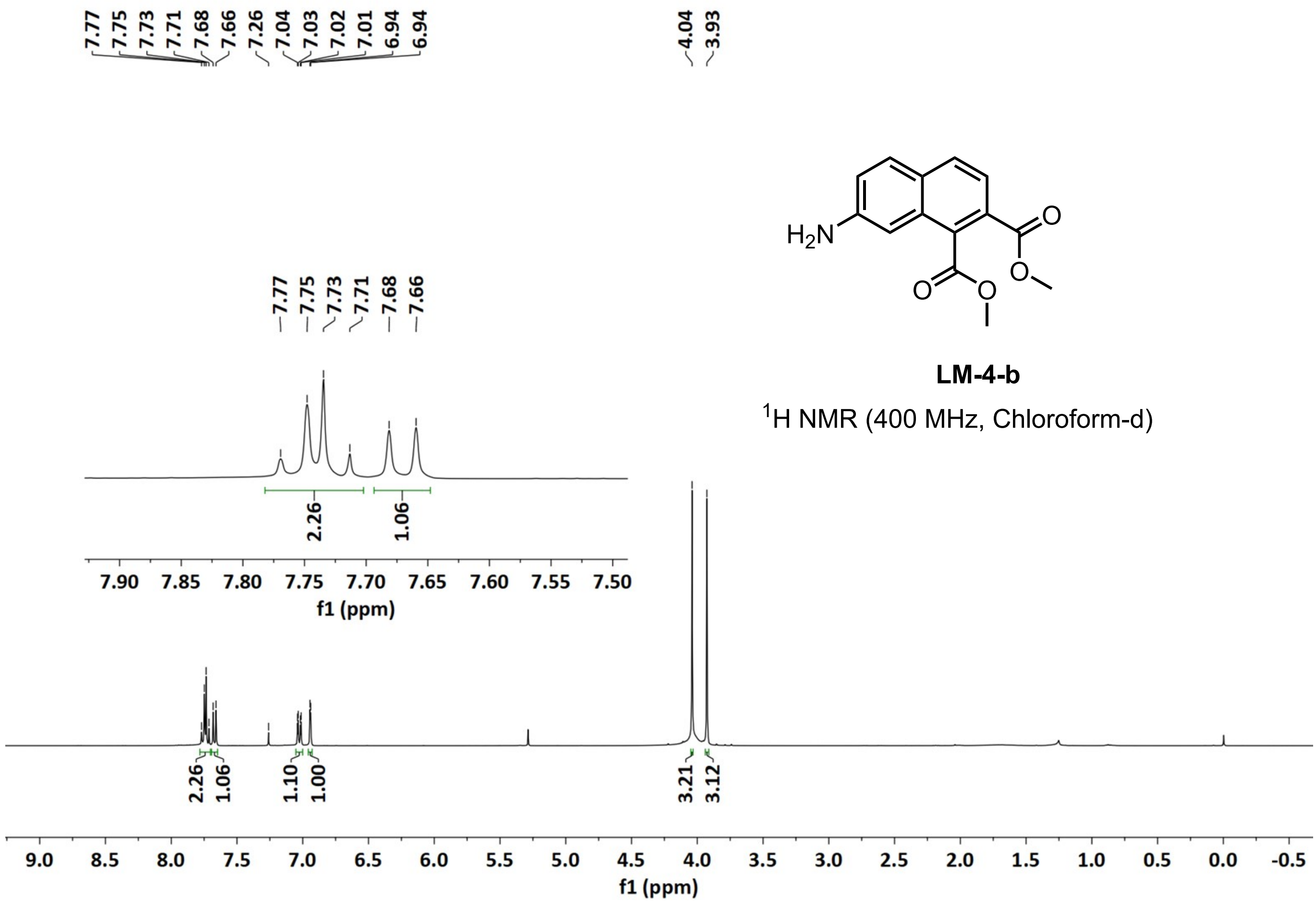

LM-4-b

^{1}H NMR (400 MHz, Chloroform-d)

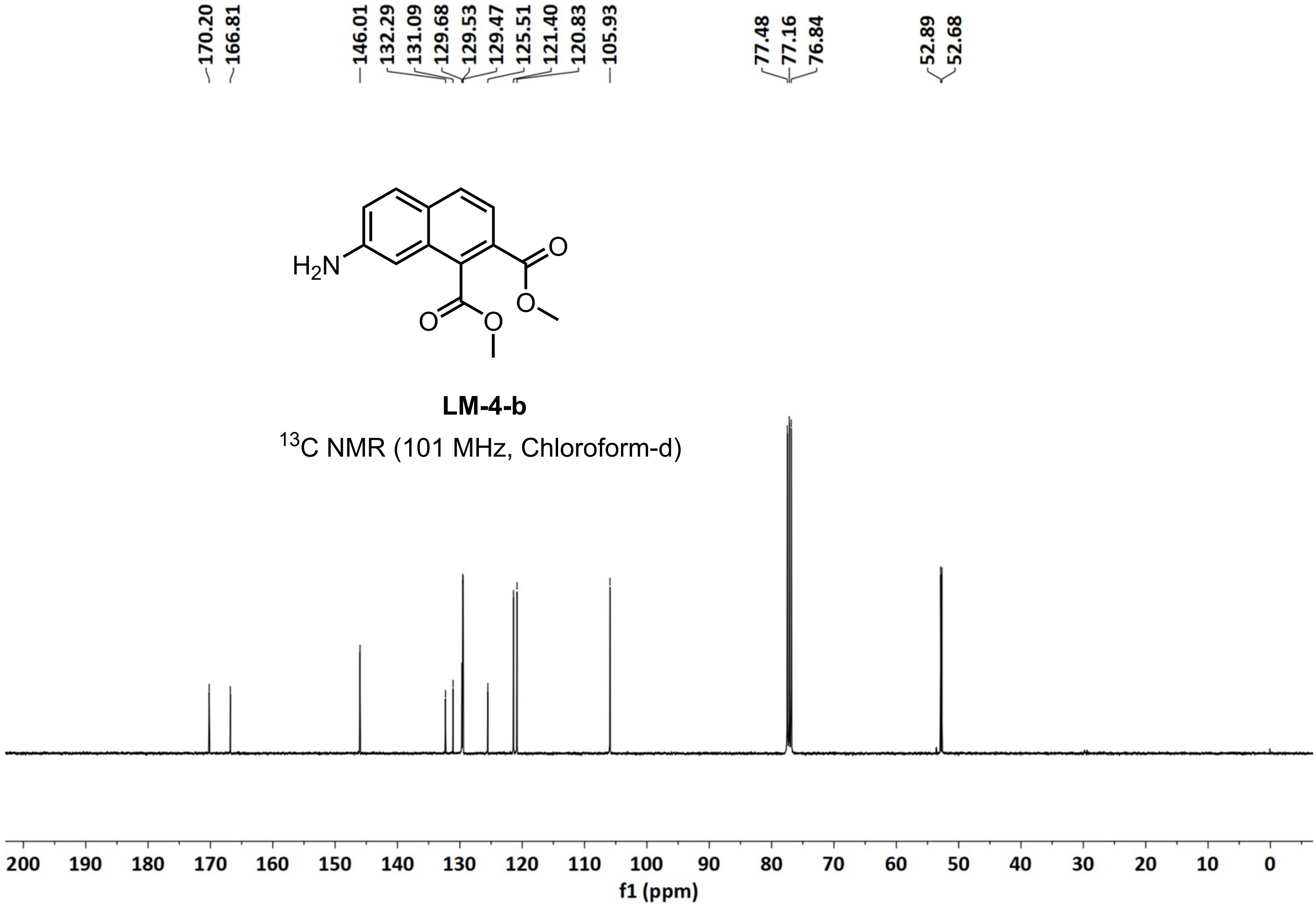

LM-4-b

^{13}C NMR (101 MHz, Chloroform-d)

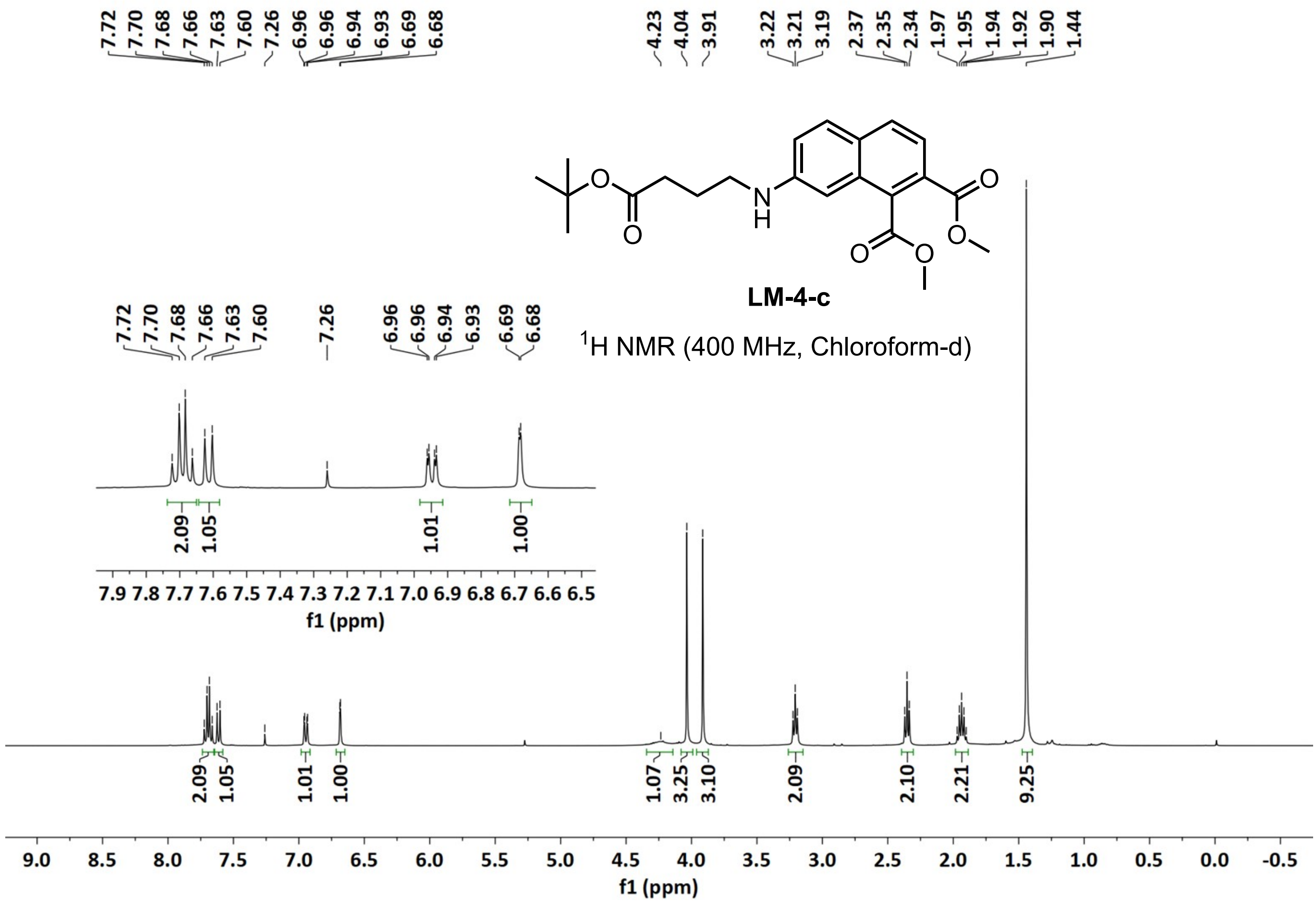
7.72
7.70
7.68
7.66
7.63
7.60
7.26
6.96
6.96
6.94
6.93
6.69
6.68
4.23
4.04
3.91
3.22
3.21
3.19
2.37
2.35
2.34
1.97
1.95
1.94
1.92
1.90
1.44
LM-4-c
^{1}H NMR (400 MHz, Chloroform-d)
2.09
1.05
1.01
1.00
1.07
3.25
3.10
2.09
2.10
2.21
9.25
f1 (ppm)

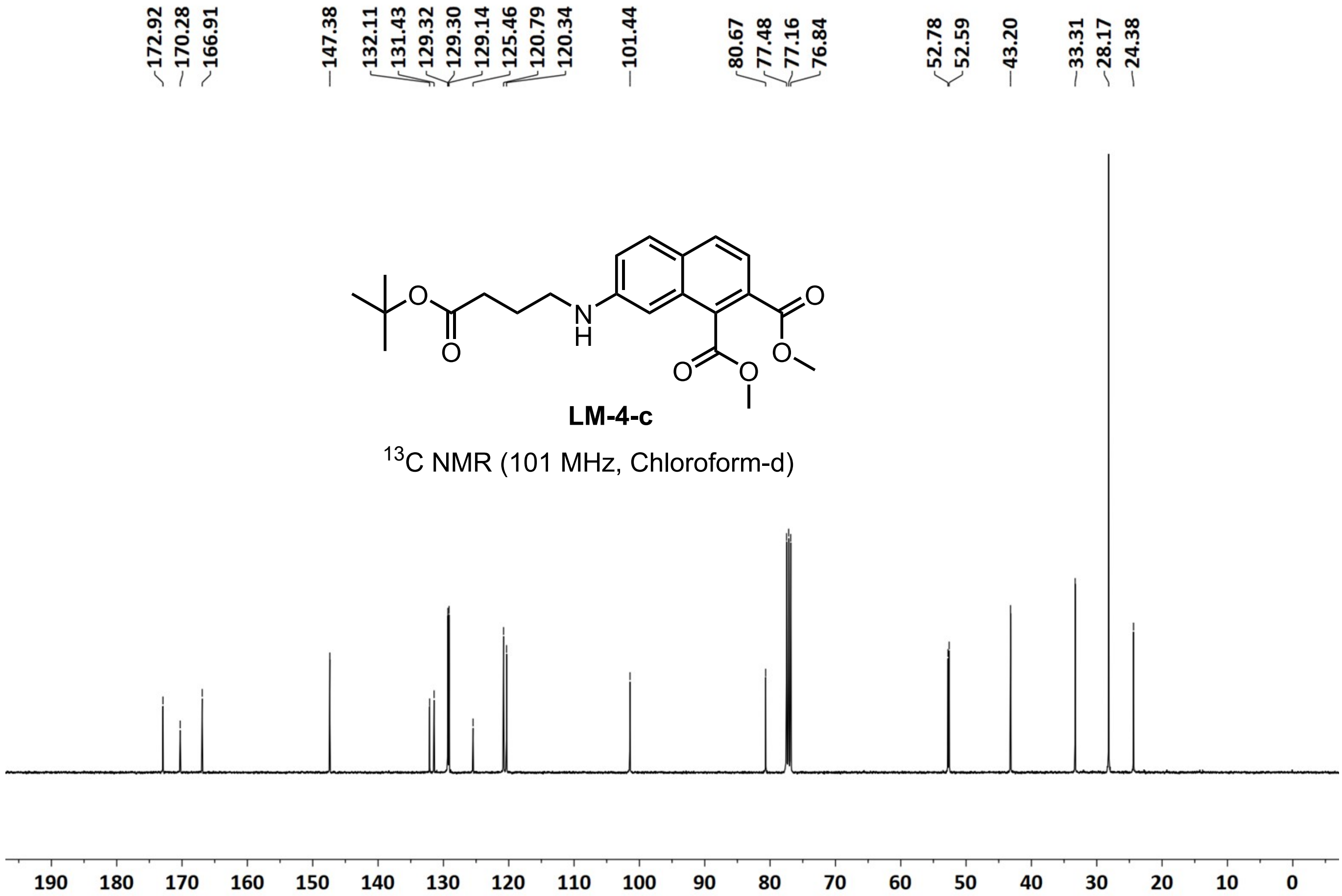
172.92
170.28
166.91
147.38
132.11
131.43
129.32
129.30
129.14
125.46
120.79
120.34
101.44
80.67
77.48
77.16
76.84
52.78
52.59
43.20
33.31
28.17
24.38
LM-4-c
^{13}C NMR (101 MHz, Chloroform-d)
f1 (ppm)

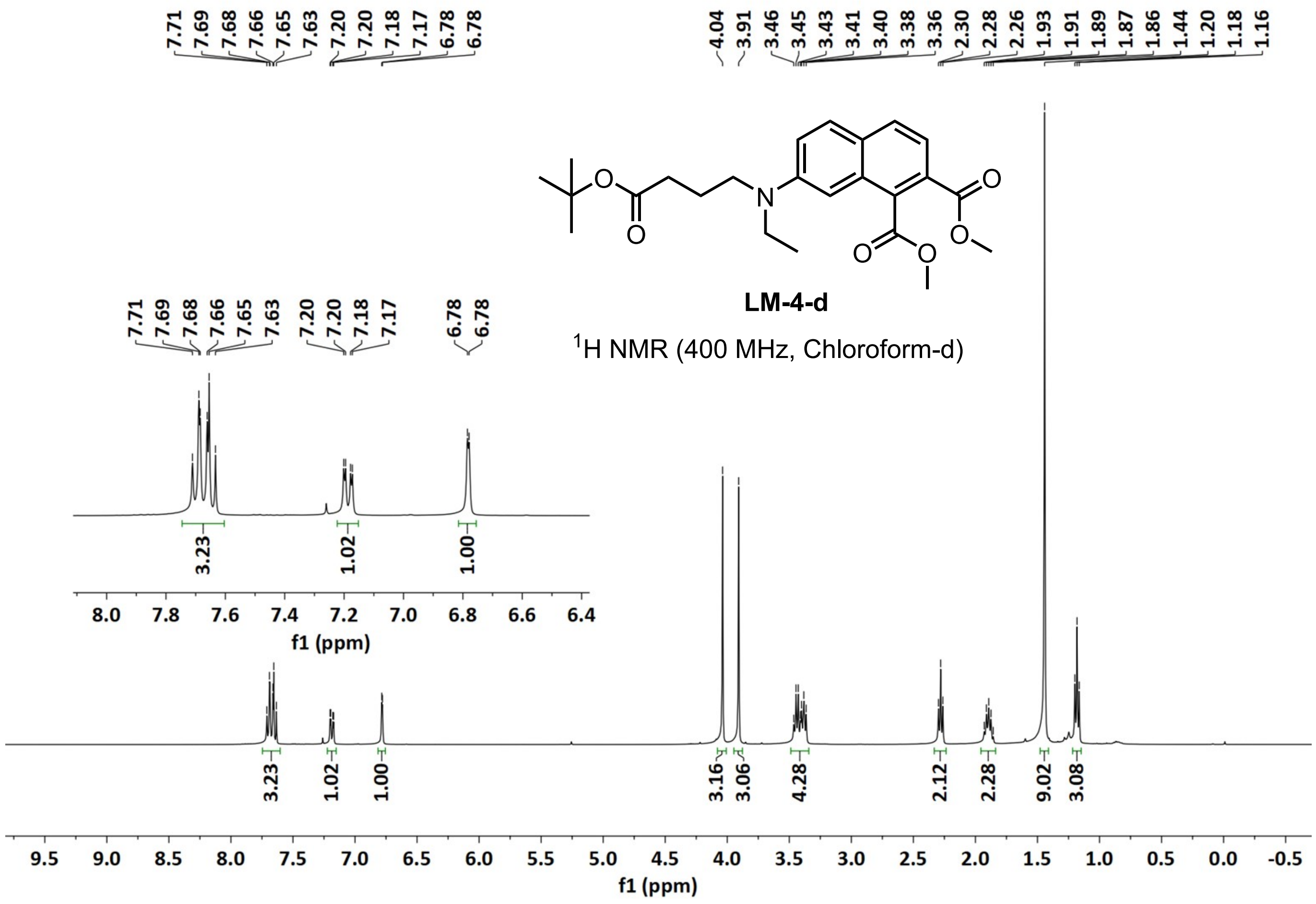
7.71 7.69 7.68 7.66 7.65 7.63 7.20 7.20 7.18 7.17 6.78 6.78
4.04 3.91 3.46 3.45 3.43 3.41 3.40 3.38 3.36 2.30 2.28 2.26 1.93 1.91 1.89 1.87 1.86 1.44 1.20 1.18 1.16
LM-4-d
^{1}H NMR (400 MHz, Chloroform-d)
3.23 1.02 1.00
3.16 3.06 4.28 2.12 2.28 9.02 3.08
f1 (ppm)

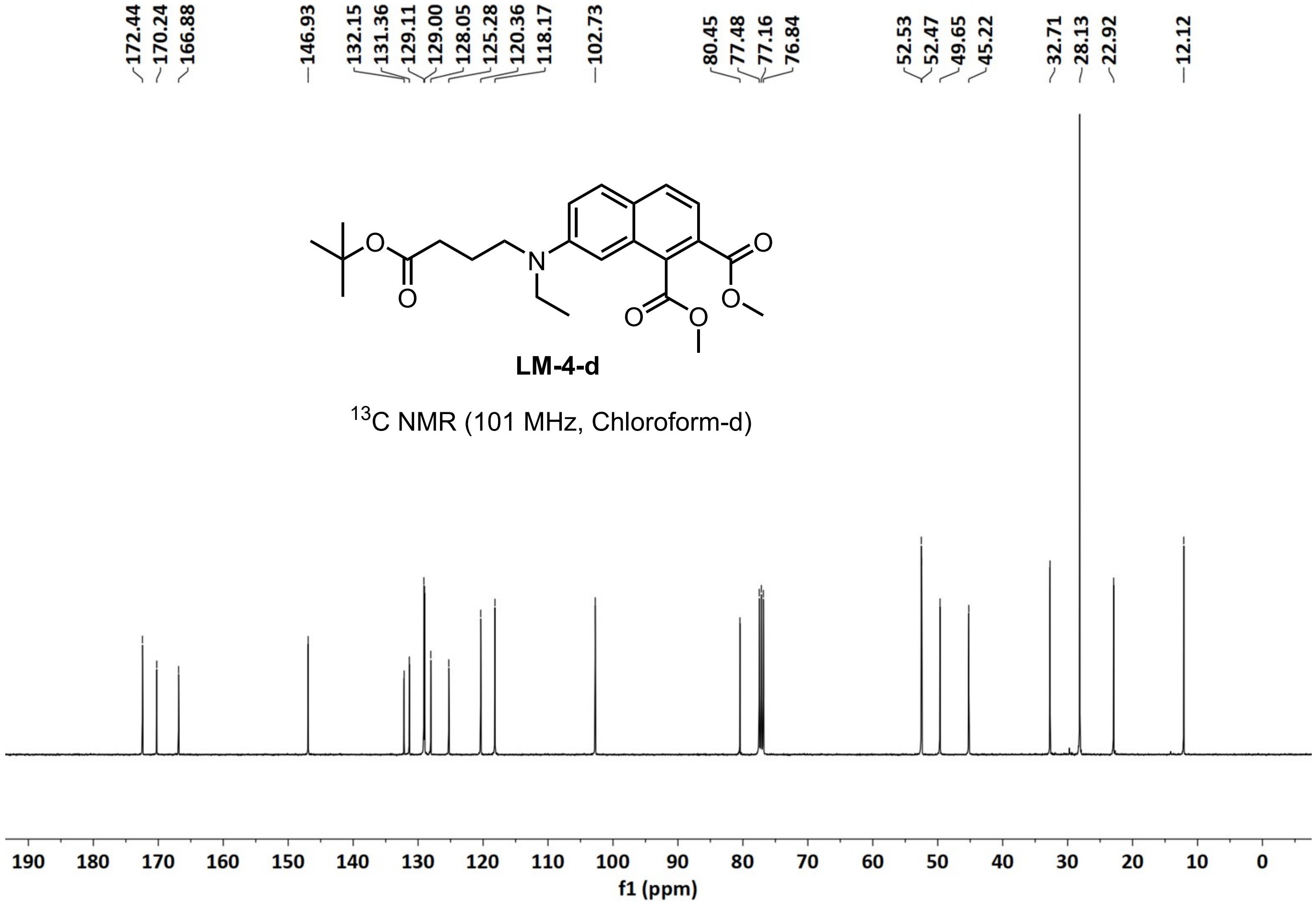
172.44 170.24 166.88 146.93 132.15 131.36 129.11 129.00 128.05 125.28 120.36 118.17 102.73 80.45 77.48 77.16 76.84 52.53 52.47 49.65 45.22 32.71 28.13 22.92 12.12
LM-4-d
^{13}C NMR (101 MHz, Chloroform-d)
f1 (ppm)

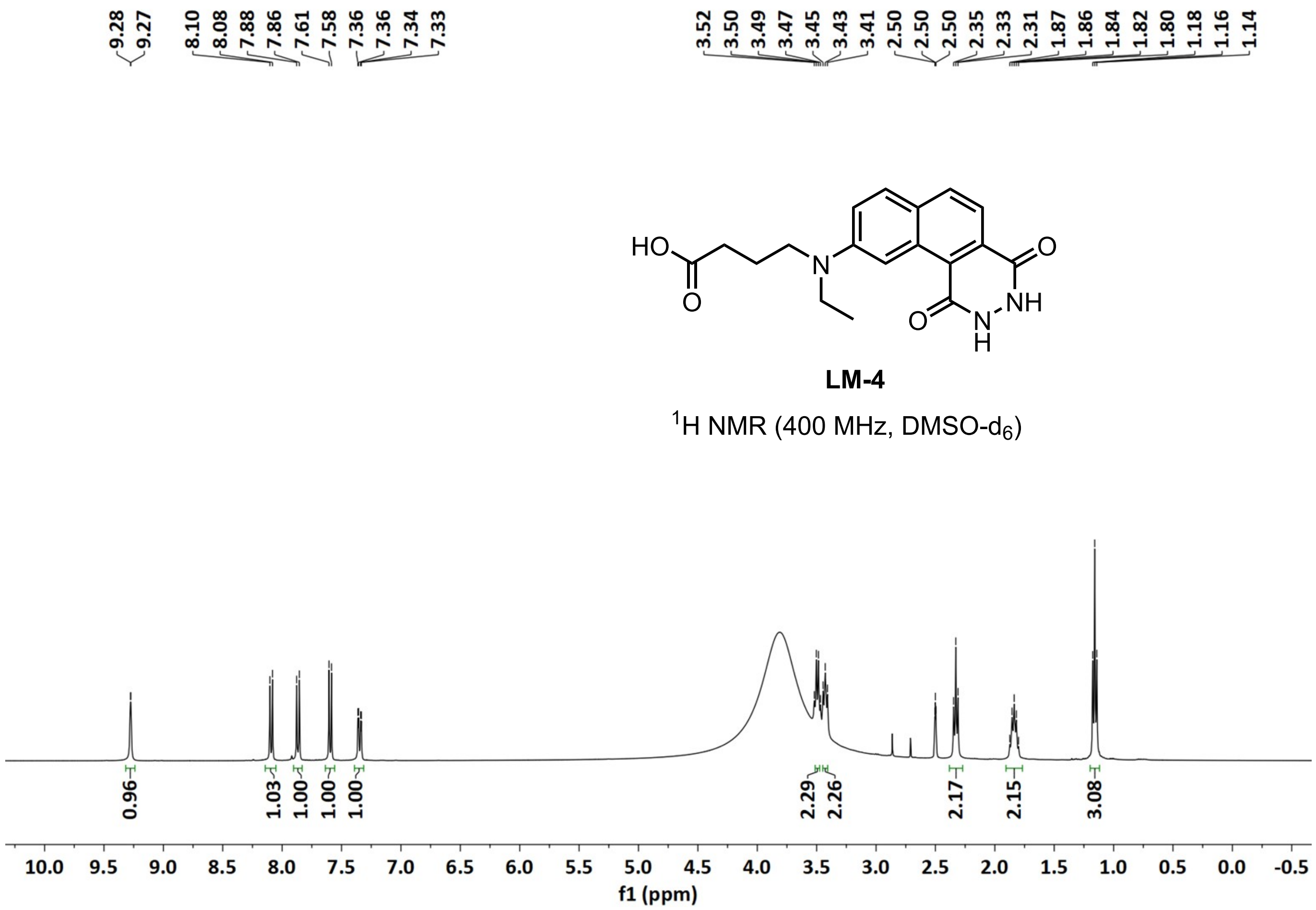
9.28
9.27
8.10
8.08
7.88
7.86
7.61
7.58
7.36
7.36
7.34
7.33
3.52
3.50
3.49
3.47
3.45
3.43
3.41
2.50
2.50
2.50
2.35
2.33
2.31
1.87
1.86
1.84
1.82
1.80
1.18
1.16
1.14
HO
O
N
O
NH
O
N
H
LM-4
1H NMR (400 MHz, DMSO-d6)
0.96
1.03
1.00
1.00
1.00
2.29
2.26
2.17
2.15
3.08
f1 (ppm)

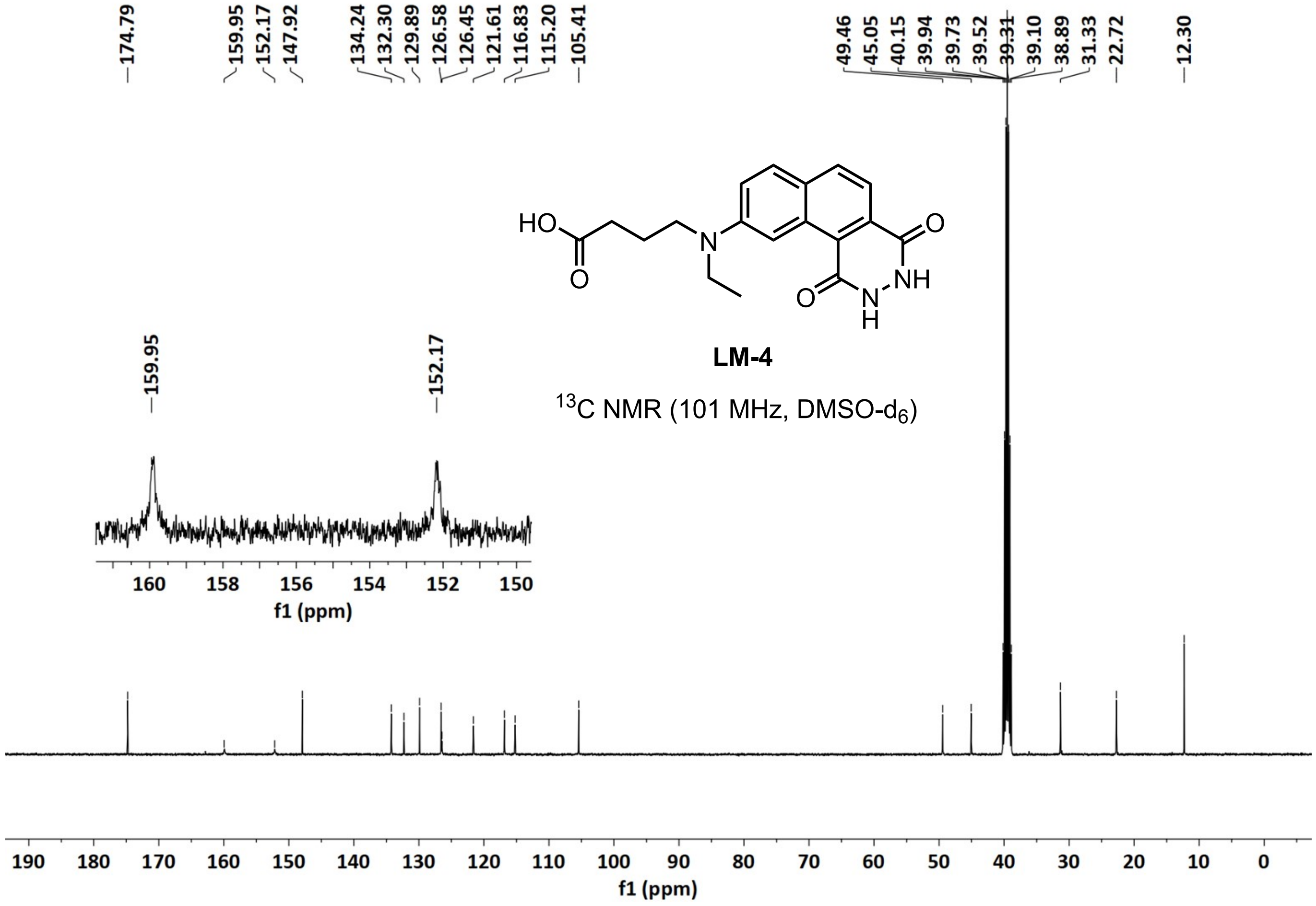
174.79
159.95
152.17
147.92
134.24
132.30
129.89
126.58
126.45
121.61
116.83
115.20
105.41
49.46
45.05
40.15
39.94
39.73
39.52
39.31
39.10
38.89
31.33
22.72
12.30
HO
O
N
O
NH
O
N
H
LM-4
13C NMR (101 MHz, DMSO-d6)
159.95
152.17
f1 (ppm)
f1 (ppm)

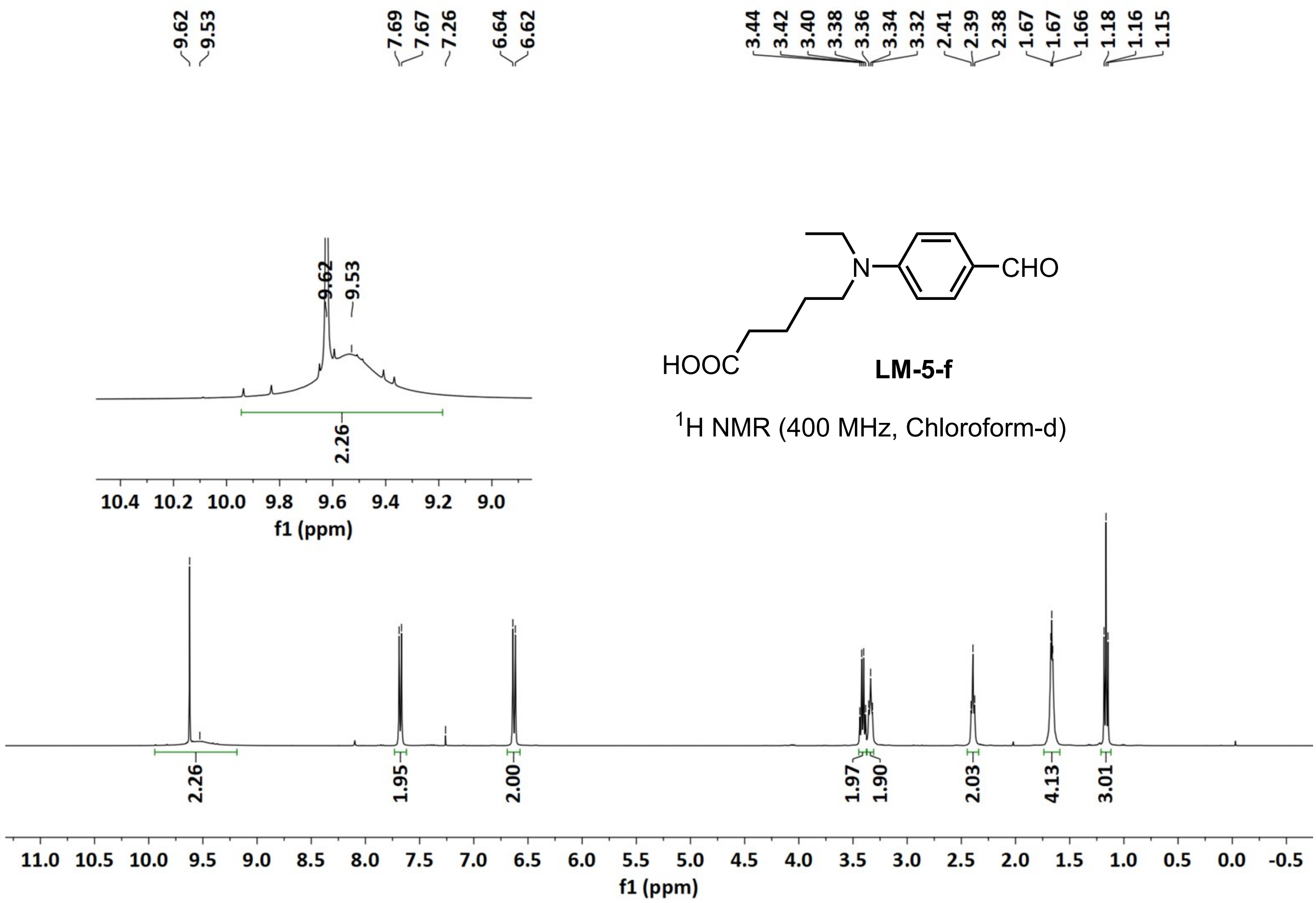
9.62
9.53
7.69
7.67
7.26
6.64
6.62
3.44
3.42
3.40
3.38
3.36
3.34
3.32
2.41
2.39
2.38
1.67
1.67
1.66
1.18
1.16
1.15
CHO
N
HOOC
LM-5-f
^{1}H NMR (400 MHz, Chloroform-d)
2.26
10.4 10.2 10.0 9.8 9.6 9.4 9.2 9.0
f1 (ppm)
2.26
1.95
2.00
1.97
1.90
2.03
4.13
3.01
11.0 10.5 10.0 9.5 9.0 8.5 8.0 7.5 7.0 6.5 6.0 5.5 5.0 4.5 4.0 3.5 3.0 2.5 2.0 1.5 1.0 0.5 0.0 -0.5
f1 (ppm)

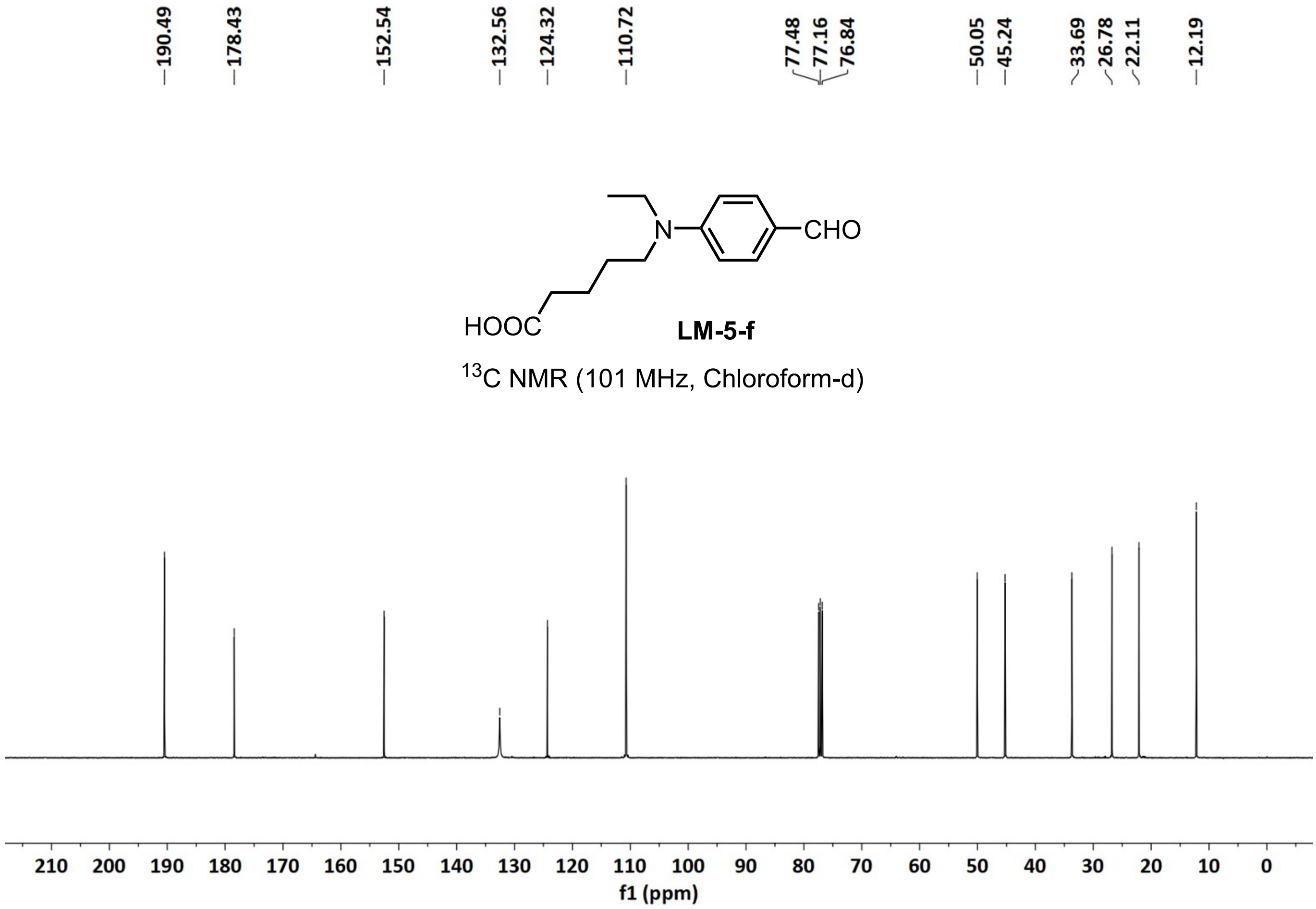
190.49
178.43
152.54
132.56
124.32
110.72
77.48
77.16
76.84
50.05
45.24
33.69
26.78
22.11
12.19
CHO
N
HOOC
LM-5-f
^{13}C NMR (101 MHz, Chloroform-d)
210 200 190 180 170 160 150 140 130 120 110 100 90 80 70 60 50 40 30 20 10 0
f1 (ppm)

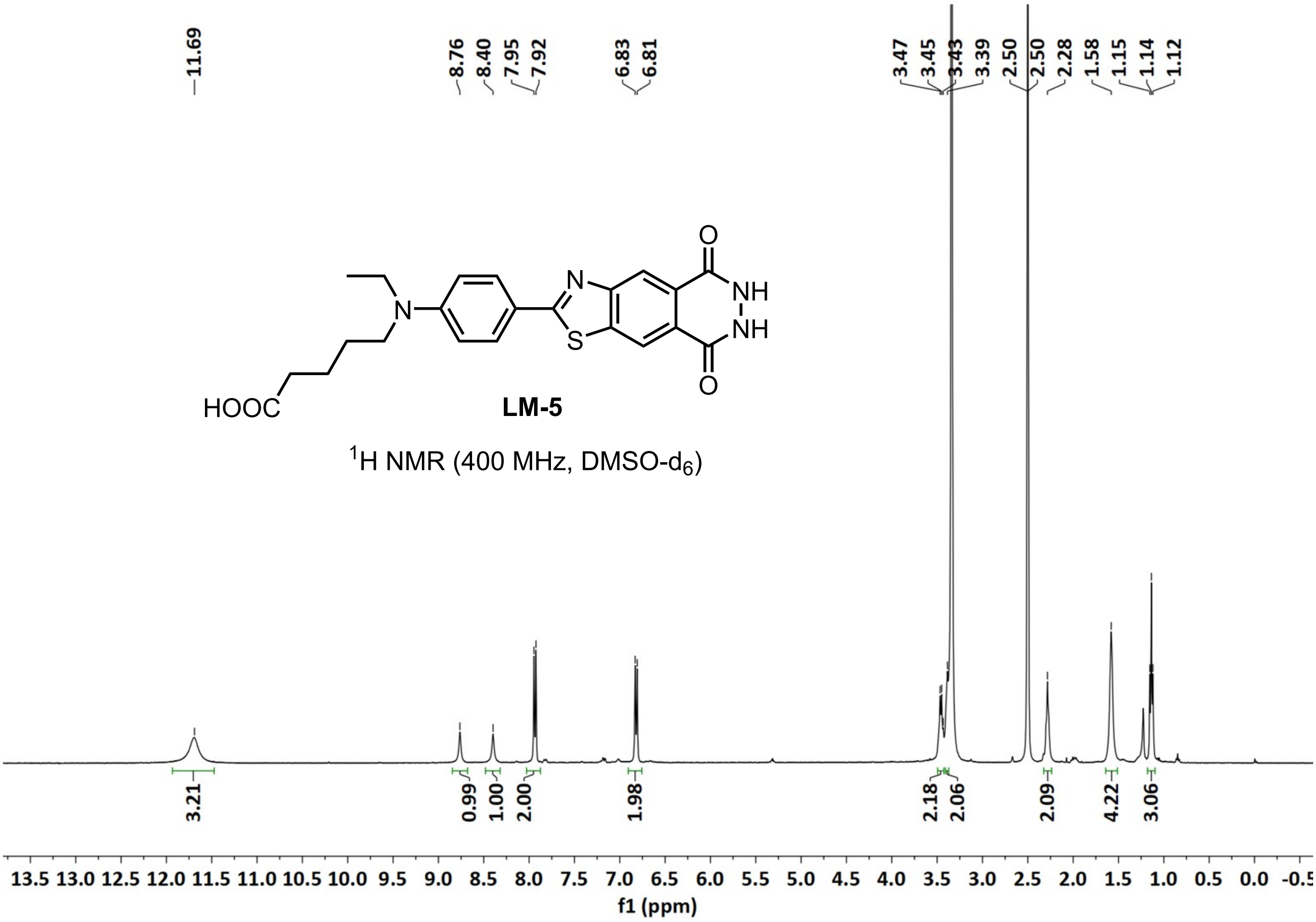
11.69
8.76
8.40
7.95
7.92
6.83
6.81
3.47
3.45
3.43
3.39
2.50
2.50
2.28
1.58
1.15
1.14
1.12
O
N
NH
N
NH
S
O
HOOC
LM-5
^{1}H NMR (400 MHz, DMSO-d_6)
3.21
0.99
1.00
2.00
1.98
2.18
2.06
2.09
4.22
3.06
13.5 13.0 12.5 12.0 11.5 11.0 10.5 10.0 9.5 9.0 8.5 8.0 7.5 7.0 6.5 6.0 5.5 5.0 4.5 4.0 3.5 3.0 2.5 2.0 1.5 1.0 0.5 0.0 -0.5
f1 (ppm)

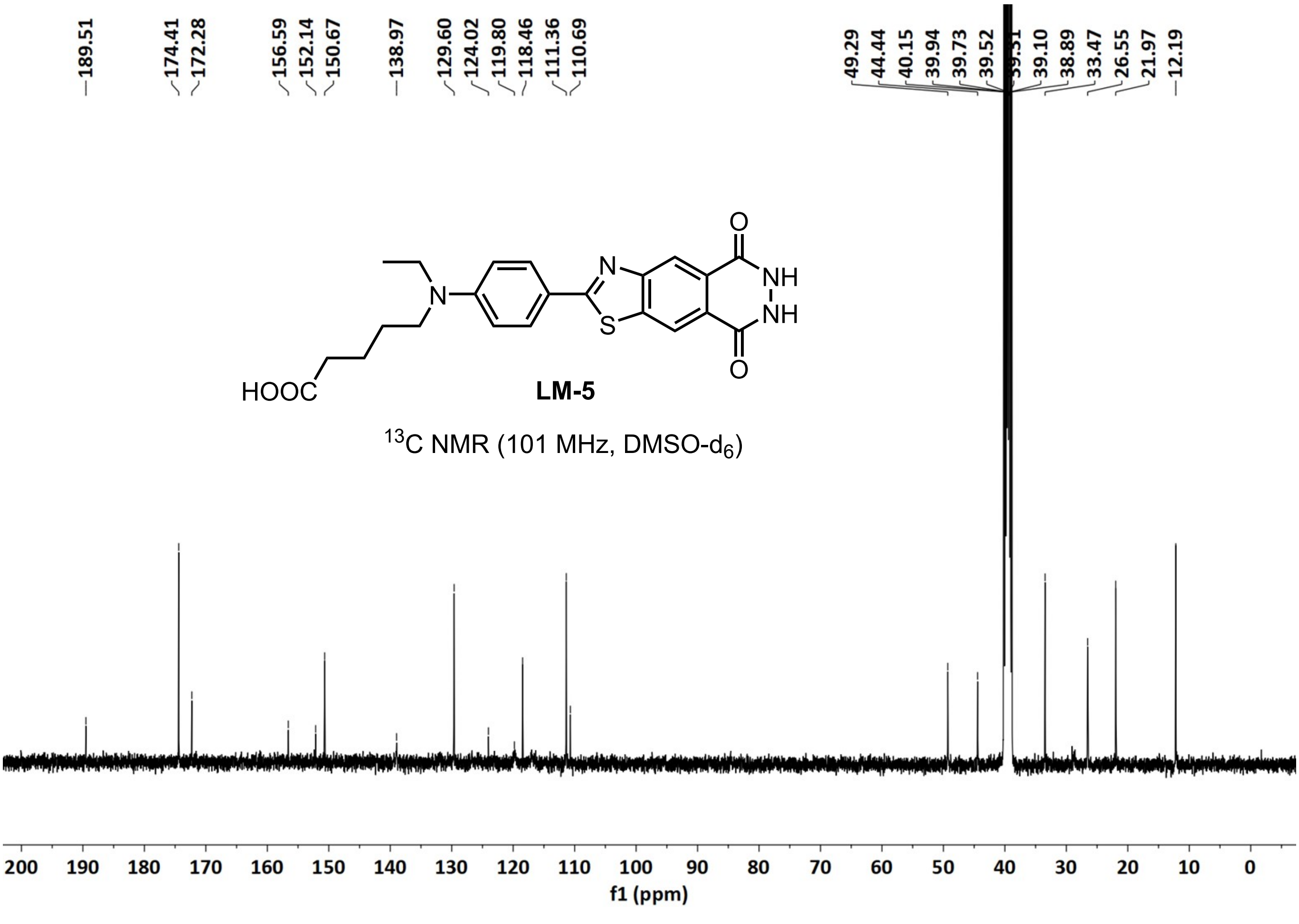
189.51
174.41
172.28
156.59
152.14
150.67
138.97
129.60
124.02
119.80
118.46
111.36
110.69
49.29
44.44
40.15
39.94
39.73
39.52
39.31
39.10
38.89
33.47
26.55
21.97
12.19
O
N
NH
N
NH
S
O
HOOC
LM-5
^{13}C NMR (101 MHz, DMSO-d_6)
200 190 180 170 160 150 140 130 120 110 100 90 80 70 60 50 40 30 20 10 0
f1 (ppm)

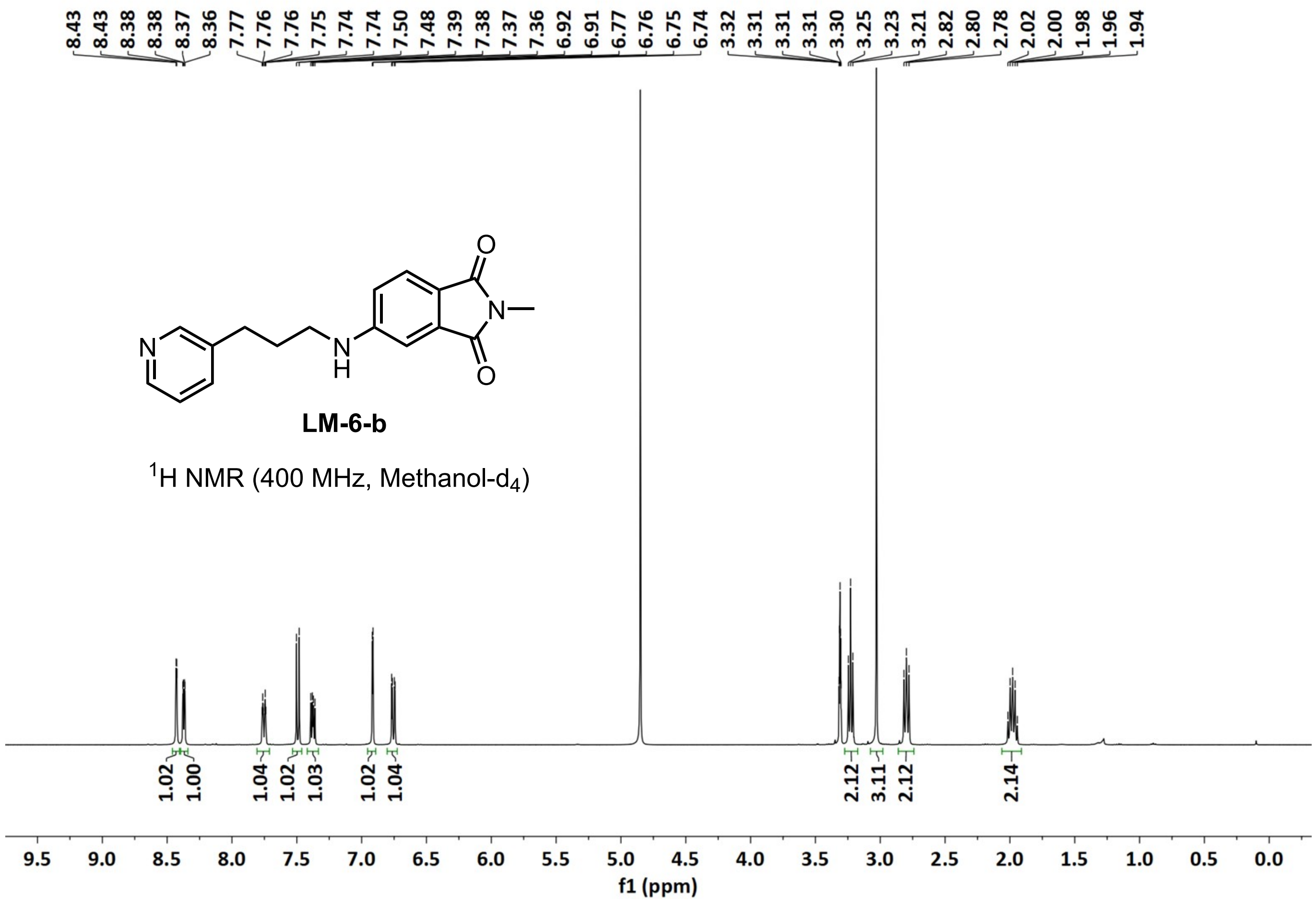
8.43 8.43 8.38 8.38 8.37 8.36 7.77 7.76 7.76 7.75 7.74 7.74 7.50 7.48 7.39 7.38 7.37 7.36 6.92 6.91 6.77 6.76 6.75 6.74 3.32 3.31 3.31 3.31 3.30 3.25 3.23 3.21 2.82 2.80 2.78 2.02 2.00 1.98 1.96 1.94
N
N
H
O
O
LM-6-b
^{1}H NMR (400 MHz, Methanol-d$_4$)
1.02 1.00 1.04 1.02 1.03 1.02 1.04 2.12 3.11 2.12 2.14
9.5 9.0 8.5 8.0 7.5 7.0 6.5 6.0 5.5 5.0 4.5 4.0 3.5 3.0 2.5 2.0 1.5 1.0 0.5 0.0
f1 (ppm)

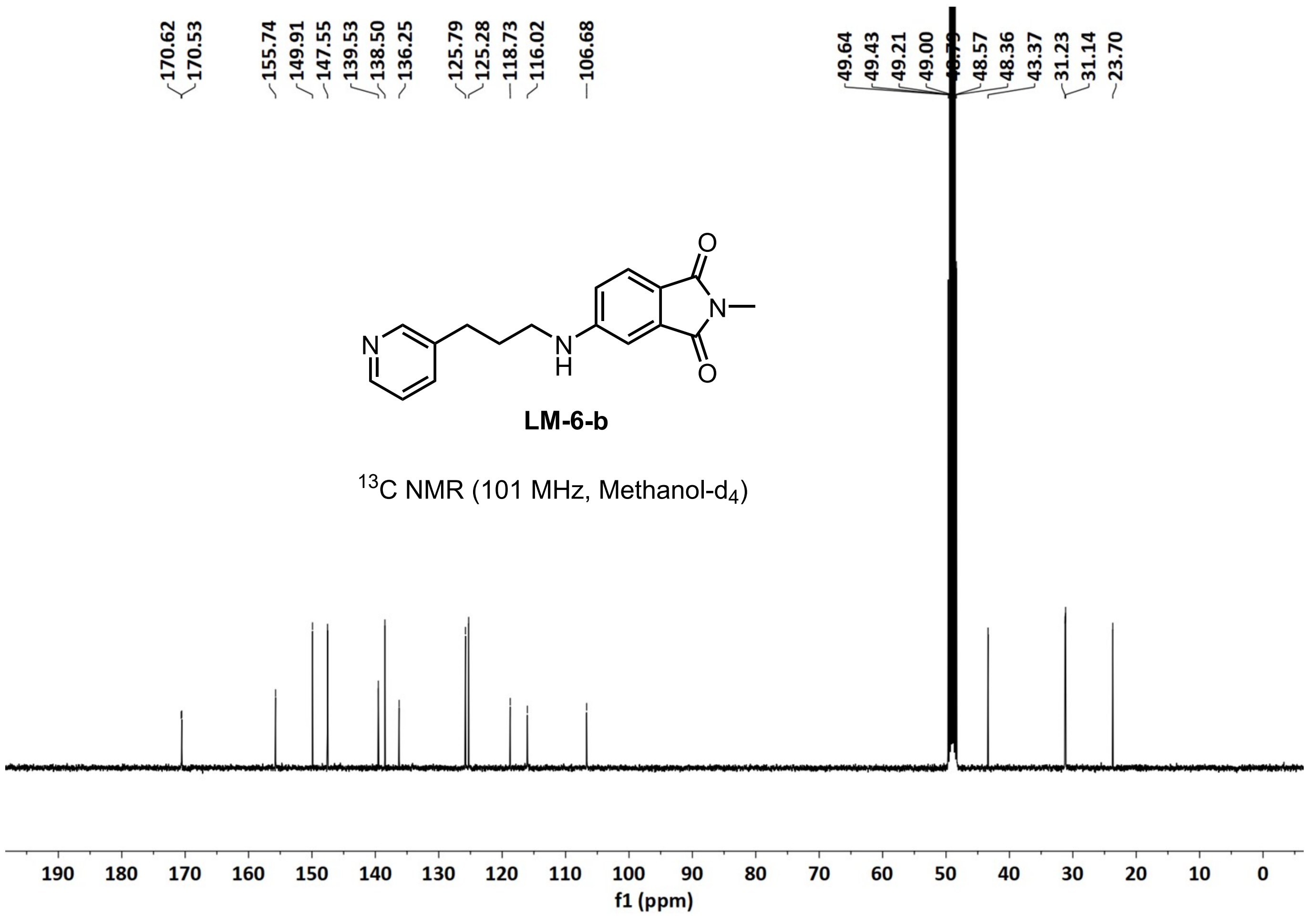
170.62 170.53 155.74 149.91 147.55 139.53 138.50 136.25 125.79 125.28 118.73 116.02 106.68
49.64 49.43 49.21 49.00 48.57 48.36 43.37 31.23 31.14 23.70
N
N
H
O
O
LM-6-b
^{13}C NMR (101 MHz, Methanol-d$_4$)
190 180 170 160 150 140 130 120 110 100 90 80 70 60 50 40 30 20 10 0
f1 (ppm)

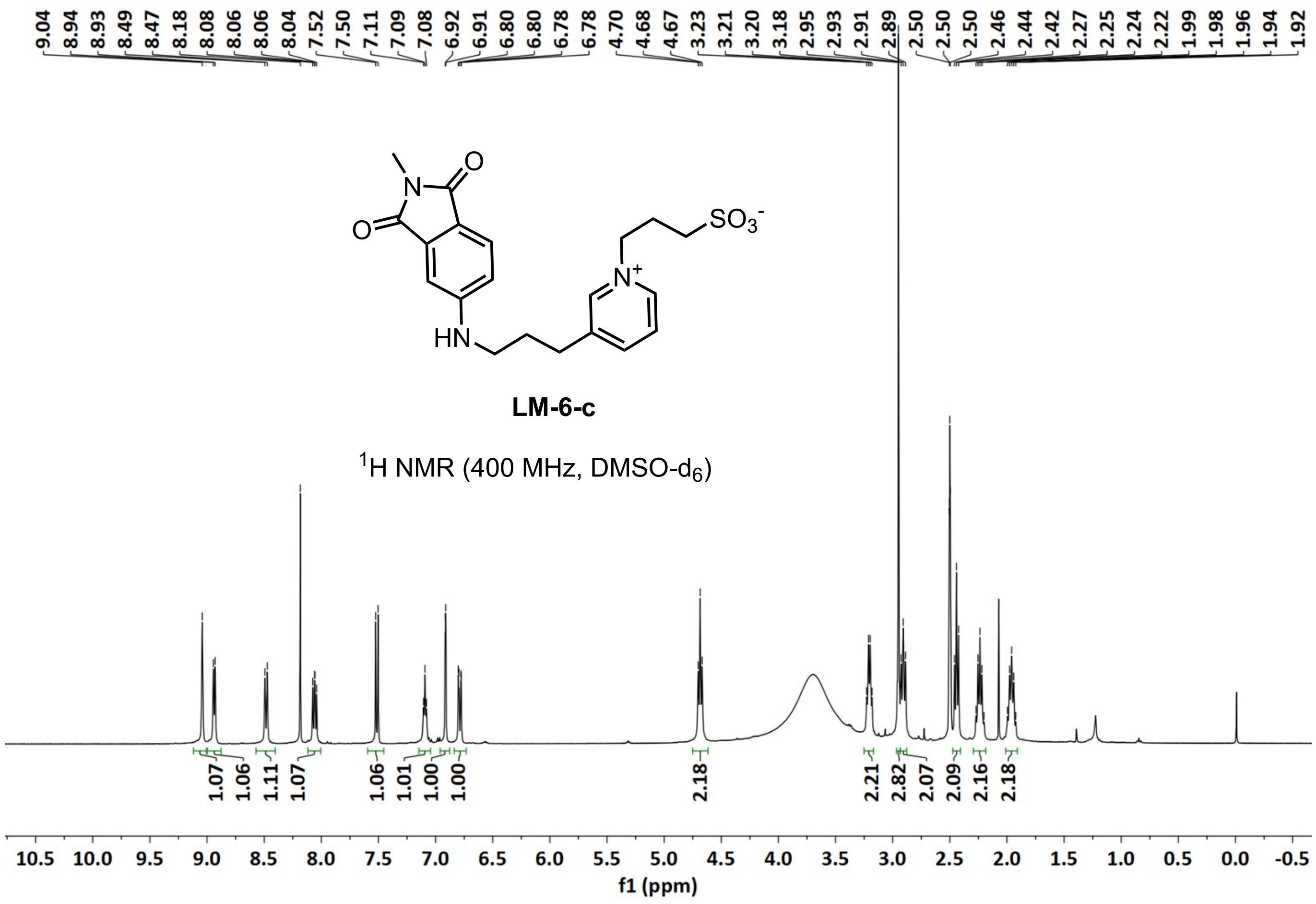
9.04 8.94 8.93 8.49 8.47 8.18 8.08 8.06 8.06 8.04 7.52 7.50 7.11 7.09 7.08 6.92 6.91 6.80 6.80 6.78 6.78 4.70 4.68 4.67 3.23 3.21 3.20 3.18 2.95 2.93 2.91 2.89 2.50 2.50 2.50 2.46 2.44 2.42 2.27 2.25 2.24 2.22 1.99 1.98 1.96 1.94 1.92
O
N
O
SO3-
N+
HN
LM-6-c
1H NMR (400 MHz, DMSO-d6)
1.07 1.06 1.11 1.07 1.06 1.01 1.00 1.00 2.18 2.21 2.82 2.07 2.09 2.16 2.18
10.5 10.0 9.5 9.0 8.5 8.0 7.5 7.0 6.5 6.0 5.5 5.0 4.5 4.0 3.5 3.0 2.5 2.0 1.5 1.0 0.5 0.0 -0.5
f1 (ppm)

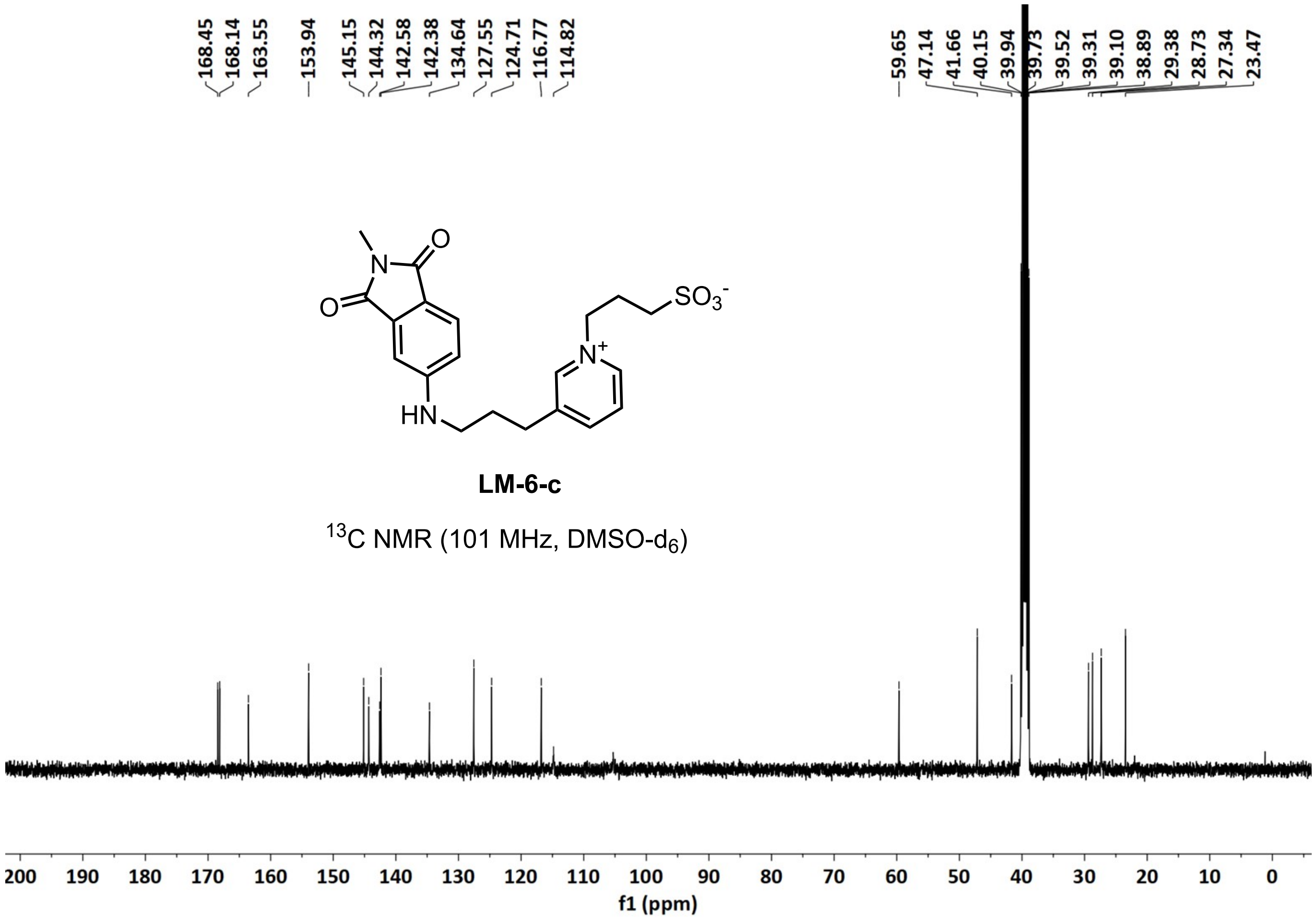
168.45 168.14 163.55 153.94 145.15 144.32 142.58 142.38 134.64 127.55 124.71 116.77 114.82
59.65 47.14 41.66 40.15 39.94 39.73 39.52 39.31 39.10 38.89 29.38 28.73 27.34 23.47
O
N
O
SO3-
N+
HN
LM-6-c
13C NMR (101 MHz, DMSO-d6)
200 190 180 170 160 150 140 130 120 110 100 90 80 70 60 50 40 30 20 10 0
f1 (ppm)

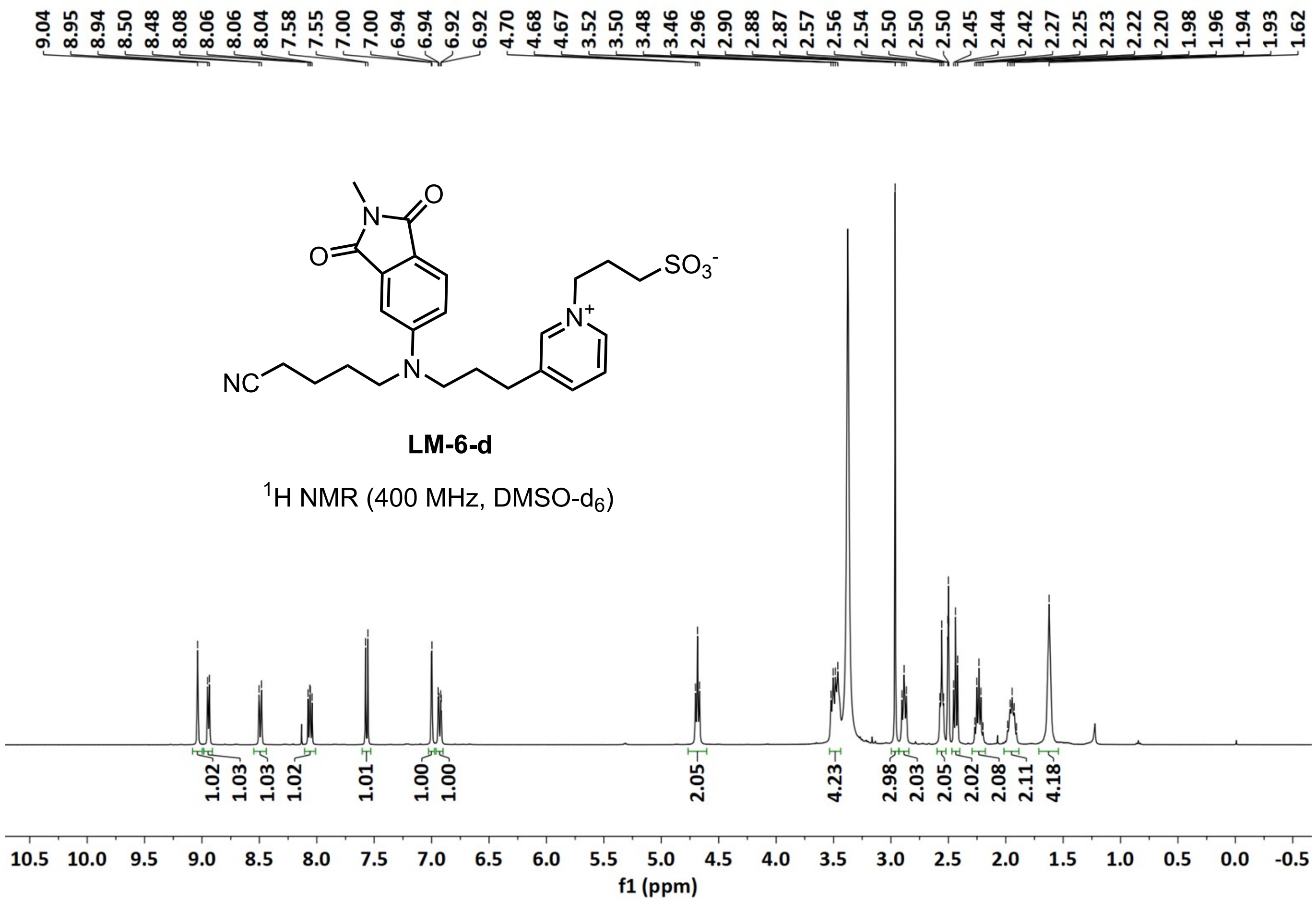
9.04 8.95 8.94 8.50 8.48 8.08 8.06 8.06 8.04 7.58 7.55 7.00 7.00 6.94 6.94 6.92 6.92 4.70 4.68 4.67 3.52 3.50 3.48 3.46 2.96 2.90 2.88 2.87 2.57 2.56 2.54 2.50 2.50 2.50 2.45 2.44 2.42 2.27 2.25 2.23 2.22 2.20 1.98 1.96 1.94 1.93 1.62
NC
N
O
SO3-
N+
LM-6-d
1H NMR (400 MHz, DMSO-d6)
1.02 1.03 1.03 1.02 1.01 1.00 1.00 2.05 4.23 2.98 2.03 2.05 2.02 2.08 2.11 4.18
10.5 10.0 9.5 9.0 8.5 8.0 7.5 7.0 6.5 6.0 5.5 5.0 4.5 4.0 3.5 3.0 2.5 2.0 1.5 1.0 0.5 0.0 -0.5
f1 (ppm)

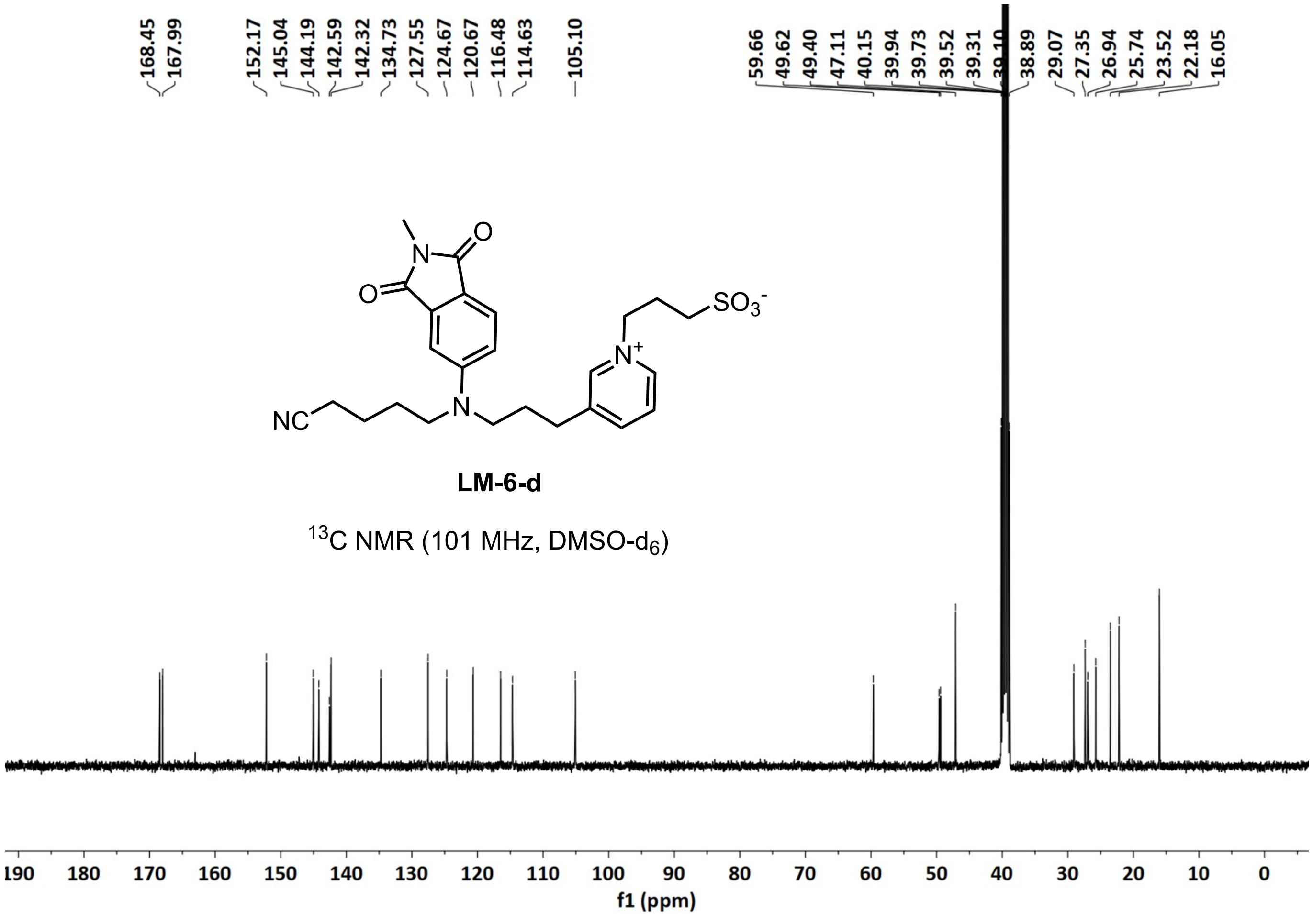
168.45 167.99 152.17 145.04 144.19 142.59 142.32 134.73 127.55 124.67 120.67 116.48 114.63 105.10
59.66 49.62 49.40 47.11 40.15 39.94 39.73 39.52 39.31 38.89 29.07 27.35 26.94 25.74 23.52 22.18 16.05
NC
N
O
SO3-
N+
LM-6-d
13C NMR (101 MHz, DMSO-d6)
180 170 160 150 140 130 120 110 100 90 80 70 60 50 40 30 20 10 0
f1 (ppm)

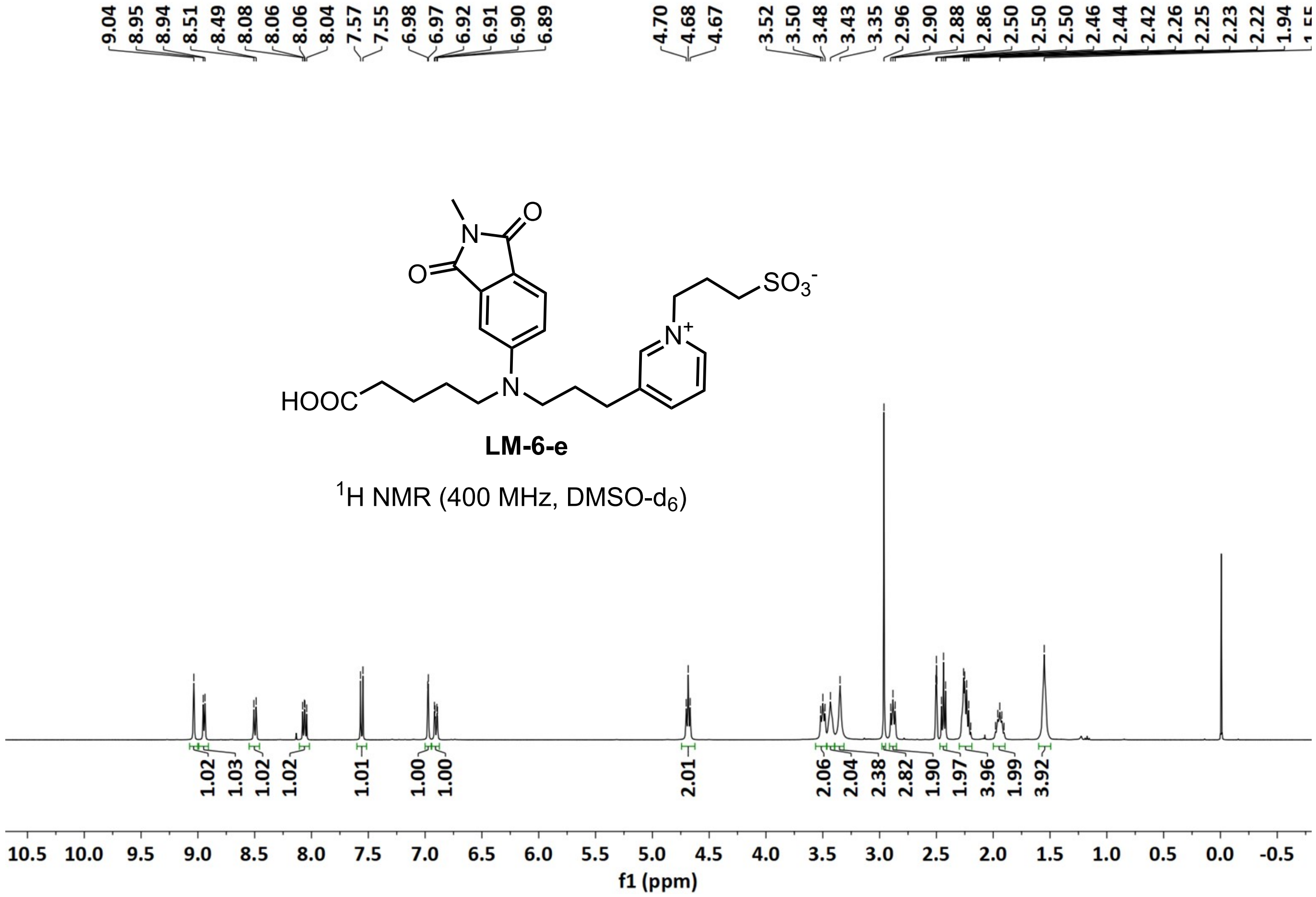
9.04 8.95 8.94 8.51 8.49 8.08 8.06 8.06 8.04 7.57 7.55 6.98 6.97 6.92 6.91 6.90 6.89
4.70 4.68 4.67
3.52 3.50 3.48 3.43 3.35 2.96 2.90 2.88 2.86 2.50 2.50 2.50 2.46 2.44 2.42 2.26 2.25 2.23 2.22 1.94
HOOC
N
O
SO3-
N+
LM-6-e
1H NMR (400 MHz, DMSO-d6)
1.02 1.03 1.02 1.02 1.01 1.00 1.00 2.01 2.06 2.04 2.38 2.82 1.90 1.97 3.96 1.99 3.92
10.5 10.0 9.5 9.0 8.5 8.0 7.5 7.0 6.5 6.0 5.5 5.0 4.5 4.0 3.5 3.0 2.5 2.0 1.5 1.0 0.5 0.0 -0.5
f1 (ppm)

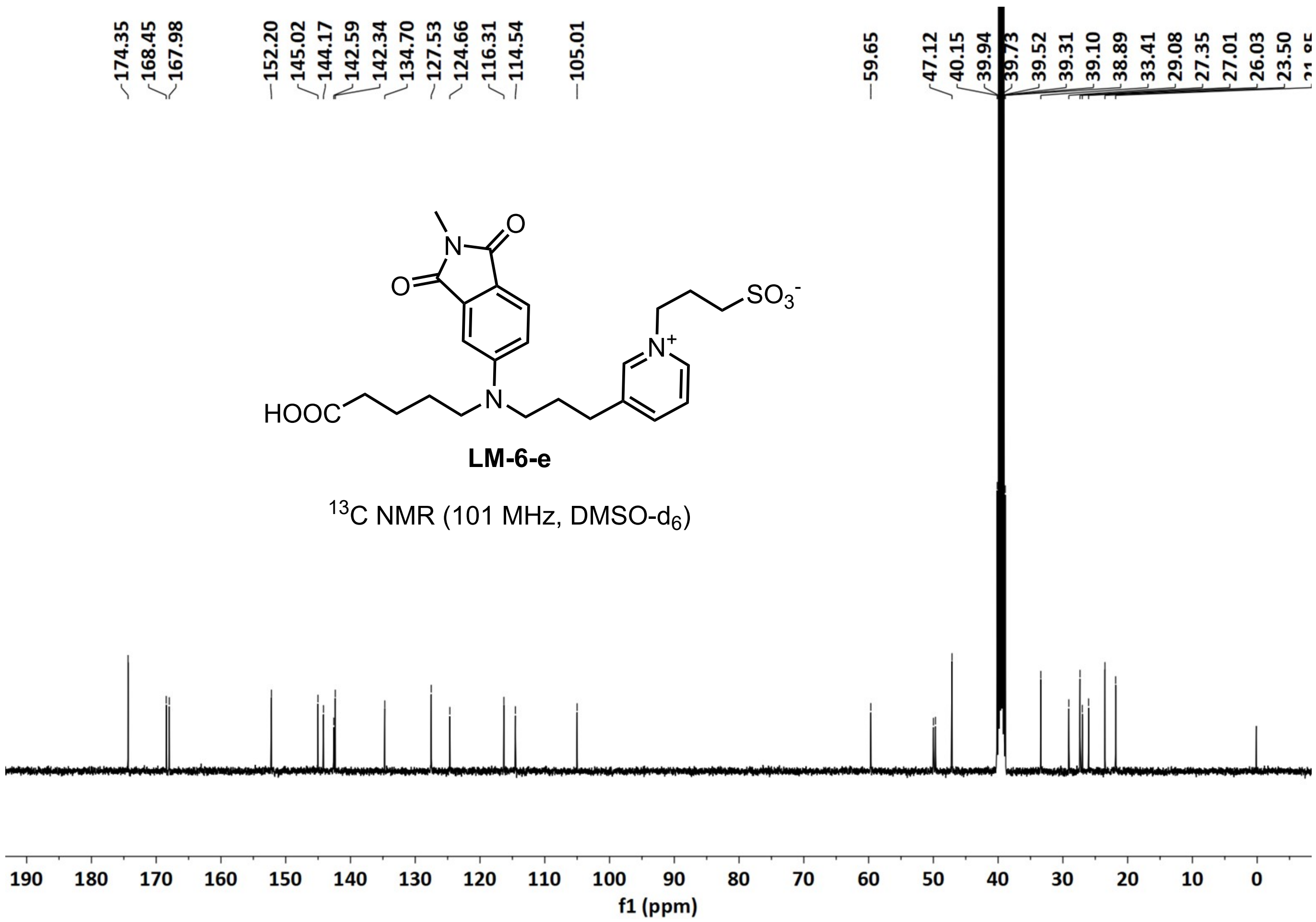
174.35 168.45 167.98 152.20 145.02 144.17 142.59 142.34 134.70 127.53 124.66 116.31 114.54 105.01
59.65 47.12 40.15 39.94 39.73 39.52 39.31 39.10 38.89 33.41 29.08 27.35 27.01 26.03 23.50
HOOC
N
O
SO3-
N+
LM-6-e
13C NMR (101 MHz, DMSO-d6)
190 180 170 160 150 140 130 120 110 100 90 80 70 60 50 40 30 20 10 0
f1 (ppm)

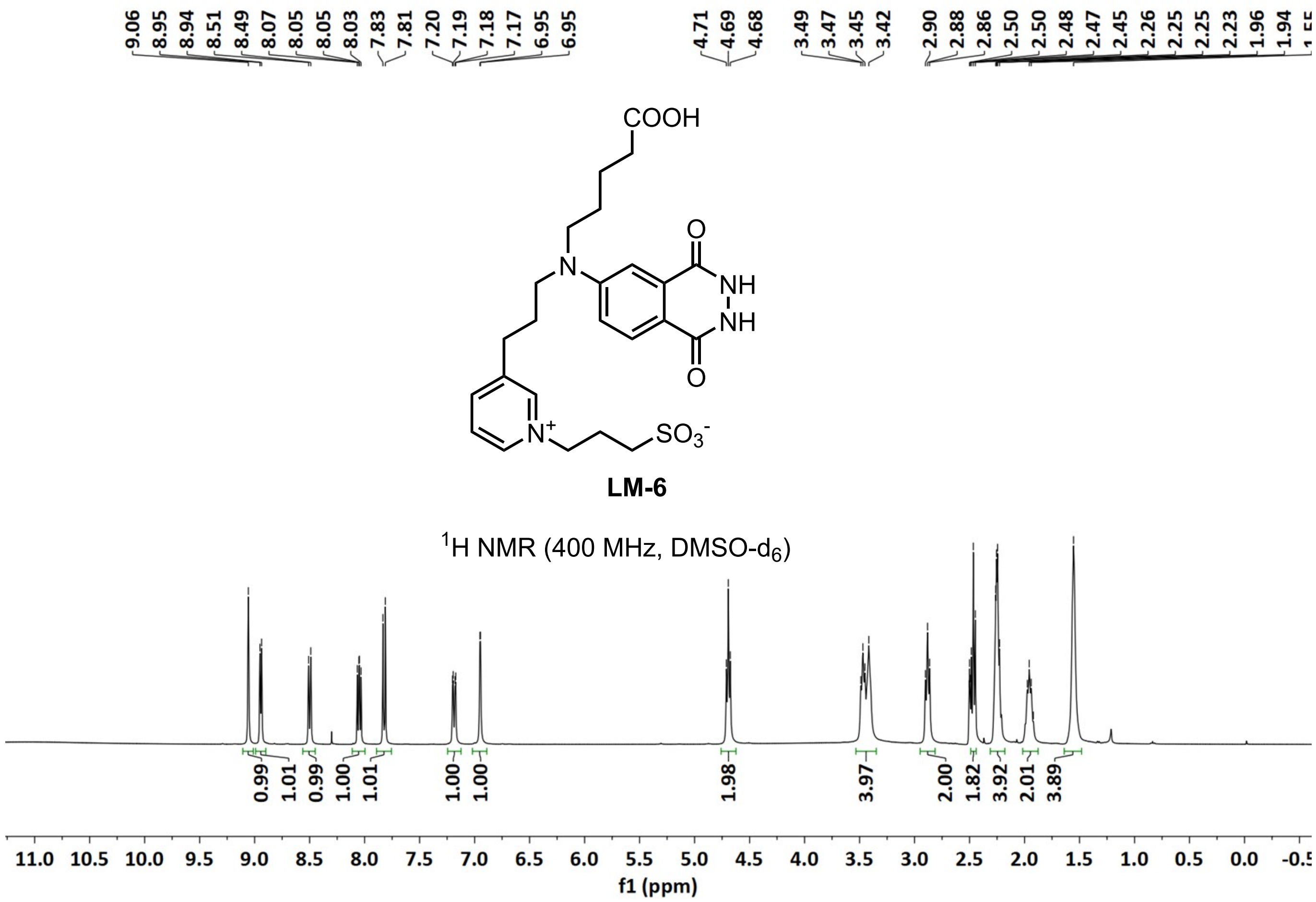
9.06 8.95 8.94 8.51 8.49 8.07 8.05 8.05 8.03 7.83 7.81 7.20 7.19 7.18 7.17 6.95 6.95 4.71 4.69 4.68 3.49 3.47 3.45 3.42 2.90 2.88 2.86 2.50 2.50 2.48 2.47 2.45 2.26 2.25 2.25 2.23 1.96 1.94
COOH
O
NH
NH
O
N
N+
SO3-
LM-6
1H NMR (400 MHz, DMSO-d6)
0.99 1.01 0.99 1.00 1.01 1.00 1.00 1.98 3.97 2.00 1.82 3.92 2.01 3.89
11.0 10.5 10.0 9.5 9.0 8.5 8.0 7.5 7.0 6.5 6.0 5.5 5.0 4.5 4.0 3.5 3.0 2.5 2.0 1.5 1.0 0.5 0.0
f1 (ppm)

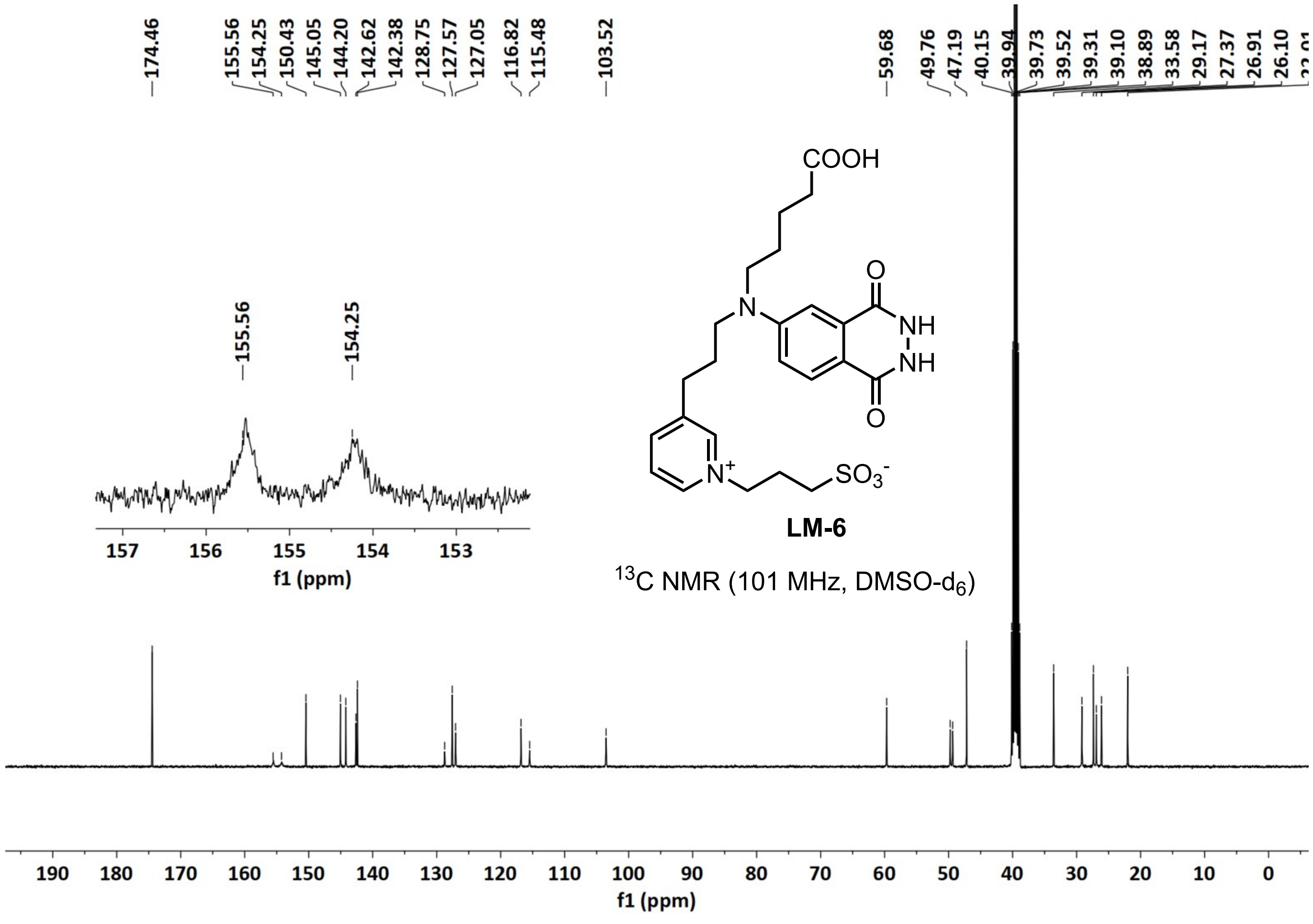
174.46 155.56 154.25 150.43 145.05 144.20 142.62 142.38 128.75 127.57 127.05 116.82 115.48 103.52 59.68 49.76 47.19 40.15 39.94 39.73 39.52 39.31 39.10 38.89 33.58 29.17 27.37 26.91 26.10
155.56 154.25
157 156 155 154 153
f1 (ppm)
COOH
O
NH
NH
O
N
N+
SO3-
LM-6
13C NMR (101 MHz, DMSO-d6)
190 180 170 160 150 140 130 120 110 100 90 80 70 60 50 40 30 20 10 0
f1 (ppm)

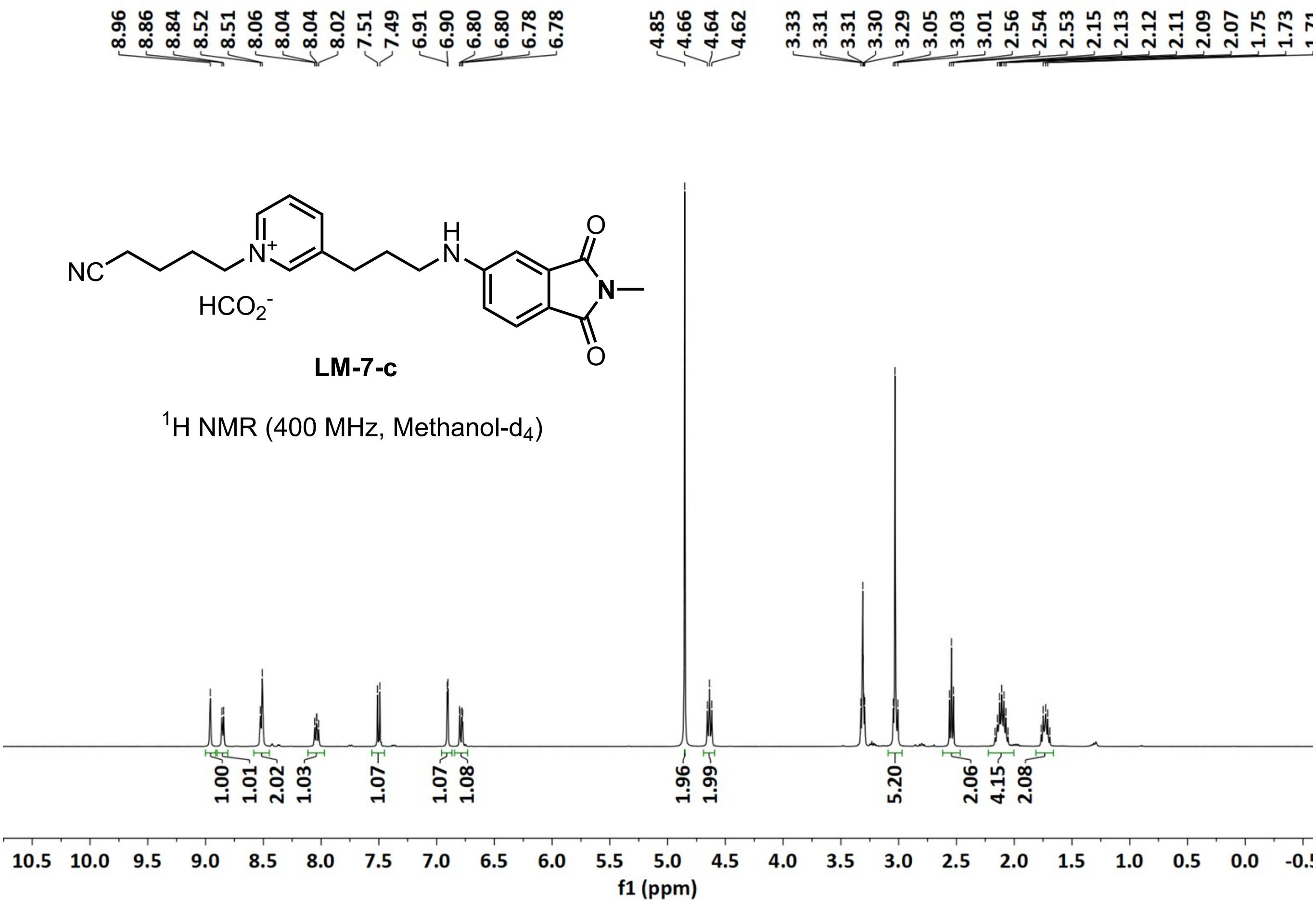
8.96 8.86 8.84 8.52 8.51 8.06 8.04 8.04 8.02 7.51 7.49 6.91 6.90 6.80 6.80 6.78 6.78
4.85 4.66 4.64 4.62
3.33 3.31 3.31 3.30 3.29 3.05 3.03 3.01 2.56 2.54 2.53 2.15 2.13 2.12 2.11 2.09 2.07 1.75 1.73 [illegible]
NC
HCO2-
H
N
N+
O
O
N
LM-7-c
1H NMR (400 MHz, Methanol-d4)
1.00 1.01 2.02 1.03 1.07 1.07 1.08 1.96 1.99 5.20 2.06 4.15 2.08
f1 (ppm)

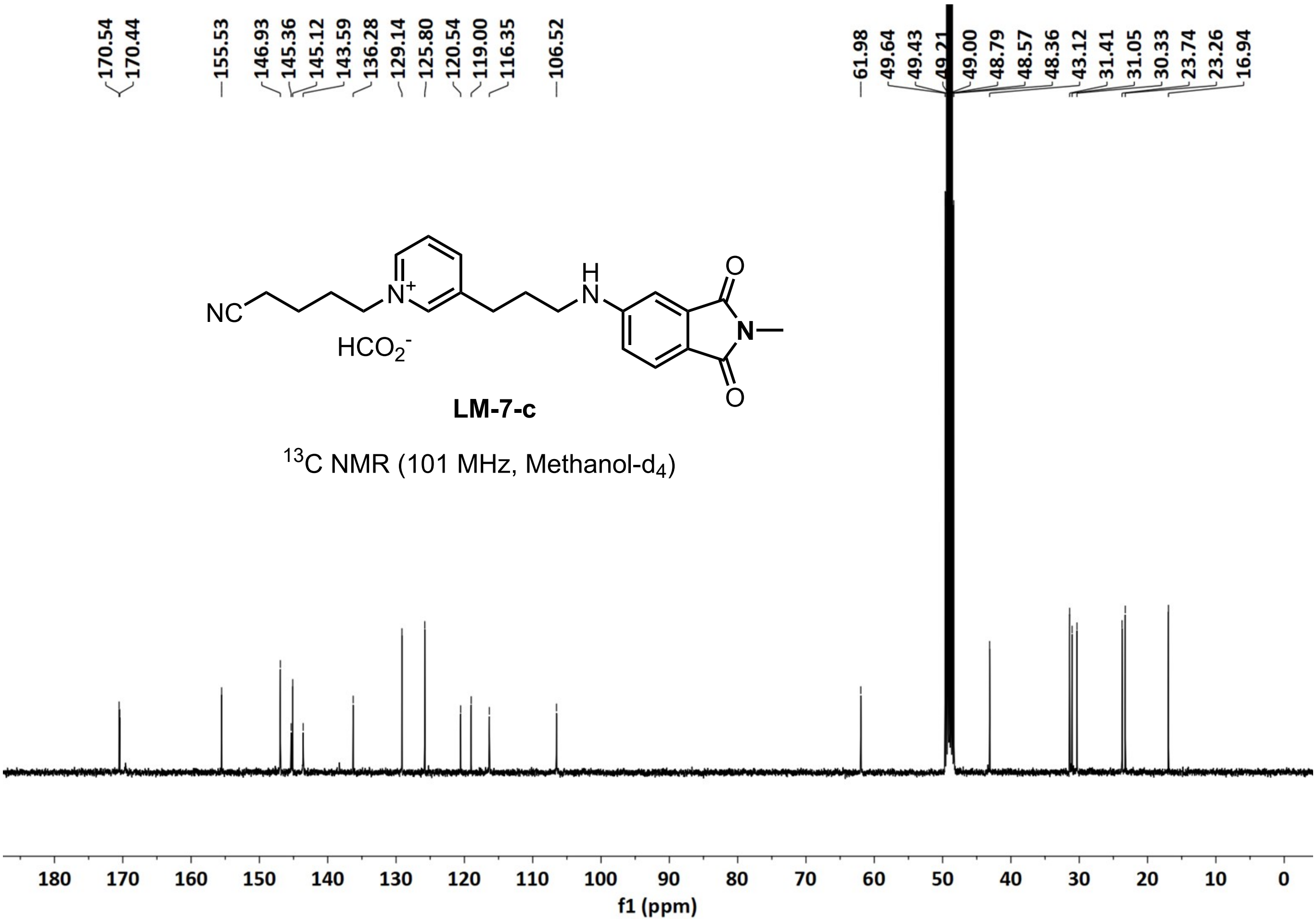
170.54 170.44 155.53 146.93 145.36 145.12 143.59 136.28 129.14 125.80 120.54 119.00 116.35 106.52
61.98 49.64 49.43 49.21 49.00 48.79 48.57 48.36 43.12 31.41 31.05 30.33 23.74 23.26 16.94
NC
HCO2-
H
N
N+
O
O
N
LM-7-c
13C NMR (101 MHz, Methanol-d4)
f1 (ppm)

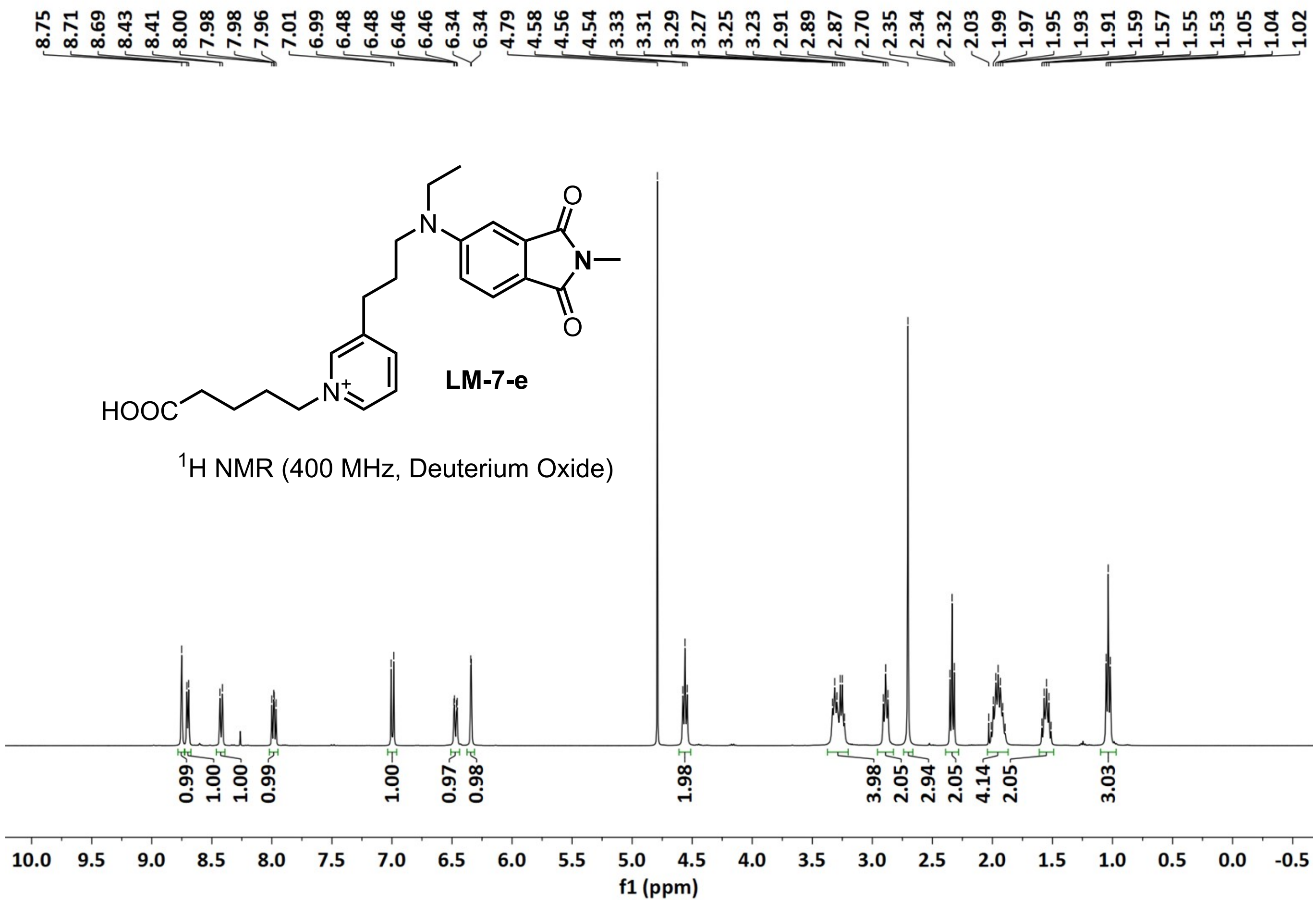
8.75 8.71 8.69 8.43 8.41 8.00 7.98 7.98 7.96 7.01 6.99 6.48 6.48 6.46 6.46 6.34 6.34 4.79 4.58 4.56 4.54 3.33 3.31 3.29 3.27 3.25 3.23 2.91 2.89 2.87 2.70 2.35 2.34 2.32 2.03 1.99 1.97 1.95 1.93 1.91 1.59 1.57 1.55 1.53 1.05 1.04 1.02
LM-7-e
HOOC
^{1}H NMR (400 MHz, Deuterium Oxide)
0.99 1.00 1.00 0.99 1.00 0.97 0.98 1.98 3.98 2.05 2.94 2.05 4.14 2.05 3.03
10.0 9.5 9.0 8.5 8.0 7.5 7.0 6.5 6.0 5.5 5.0 4.5 4.0 3.5 3.0 2.5 2.0 1.5 1.0 0.5 0.0 -0.5
f1 (ppm)

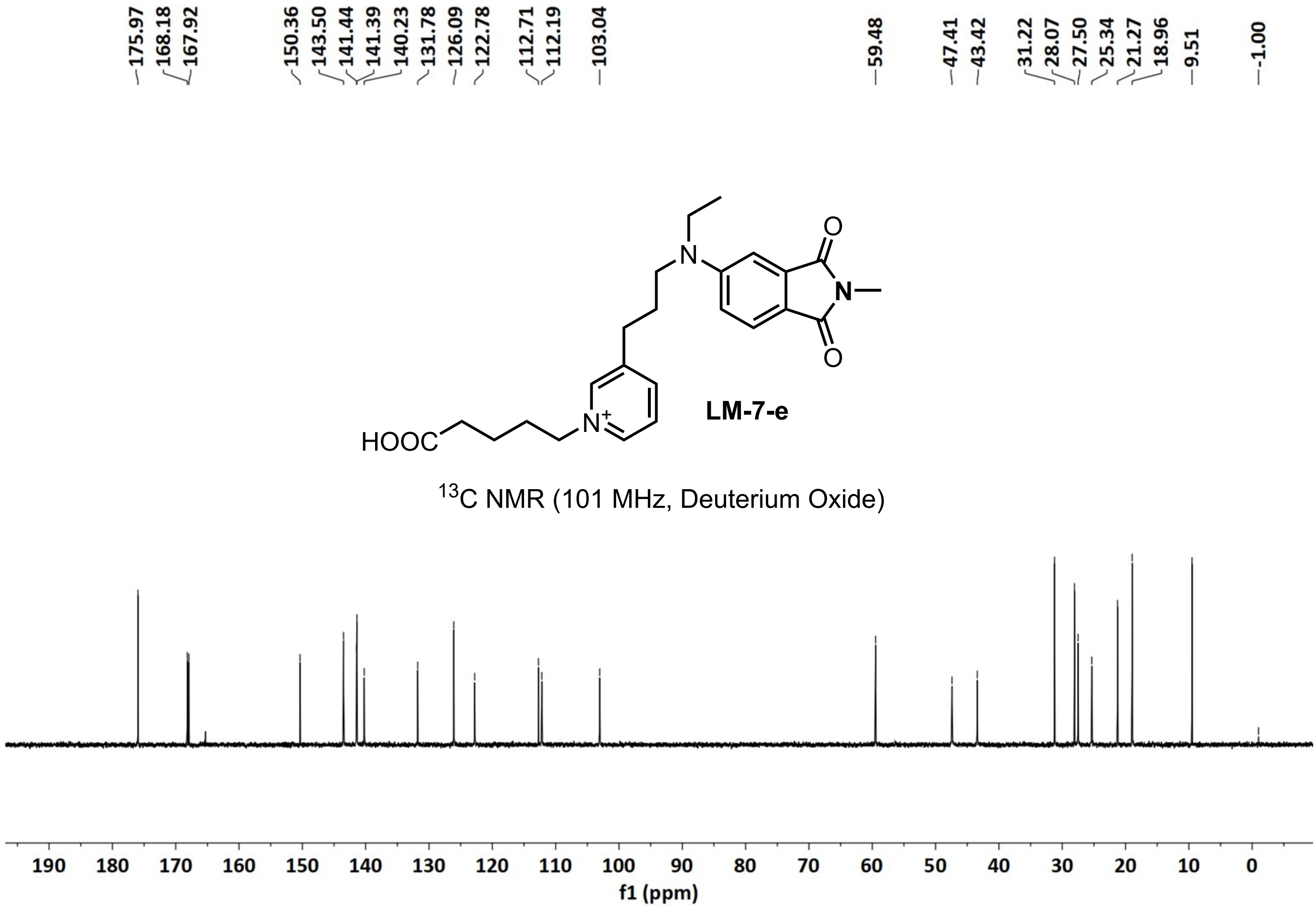
175.97 168.18 167.92 150.36 143.50 141.44 141.39 140.23 131.78 126.09 122.78 112.71 112.19 103.04 59.48 47.41 43.42 31.22 28.07 27.50 25.34 21.27 18.96 9.51 -1.00
LM-7-e
HOOC
^{13}C NMR (101 MHz, Deuterium Oxide)
190 180 170 160 150 140 130 120 110 100 90 80 70 60 50 40 30 20 10 0
f1 (ppm)

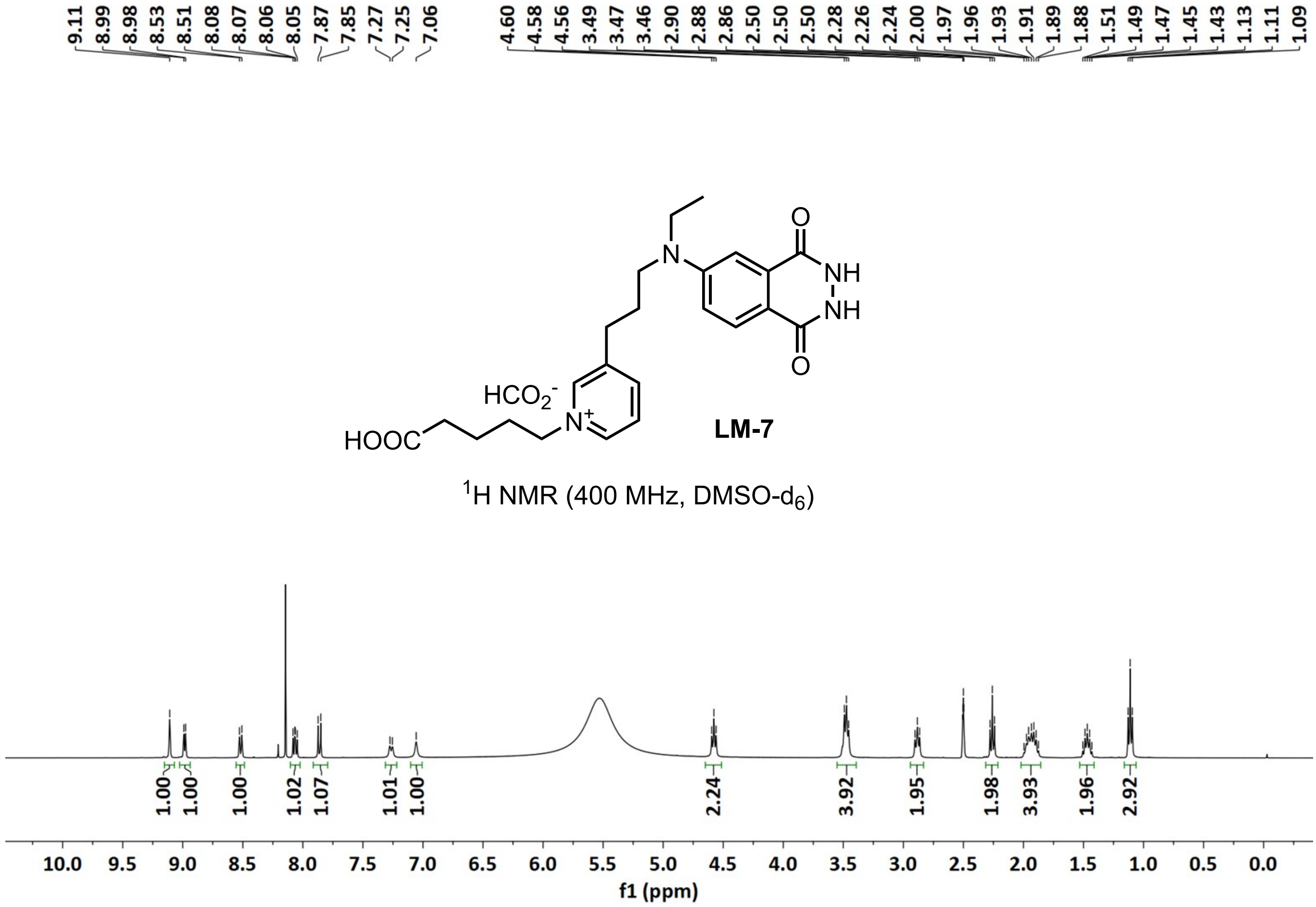
9.11 8.99 8.98 8.53 8.51 8.08 8.07 8.06 8.05 7.87 7.85 7.27 7.25 7.06
4.60 4.58 4.56 3.49 3.47 3.46 2.90 2.88 2.86 2.50 2.50 2.50 2.28 2.26 2.24 2.00 1.97 1.96 1.93 1.91 1.89 1.88 1.51 1.49 1.47 1.45 1.43 1.13 1.11 1.09
O
NH
NH
O
N
HCO2-
N+
HOOC
LM-7
1H NMR (400 MHz, DMSO-d6)
1.00 1.00 1.00 1.02 1.07 1.01 1.00 2.24 3.92 1.95 1.98 3.93 1.96 2.92
10.0 9.5 9.0 8.5 8.0 7.5 7.0 6.5 6.0 5.5 5.0 4.5 4.0 3.5 3.0 2.5 2.0 1.5 1.0 0.5 0.0
f1 (ppm)

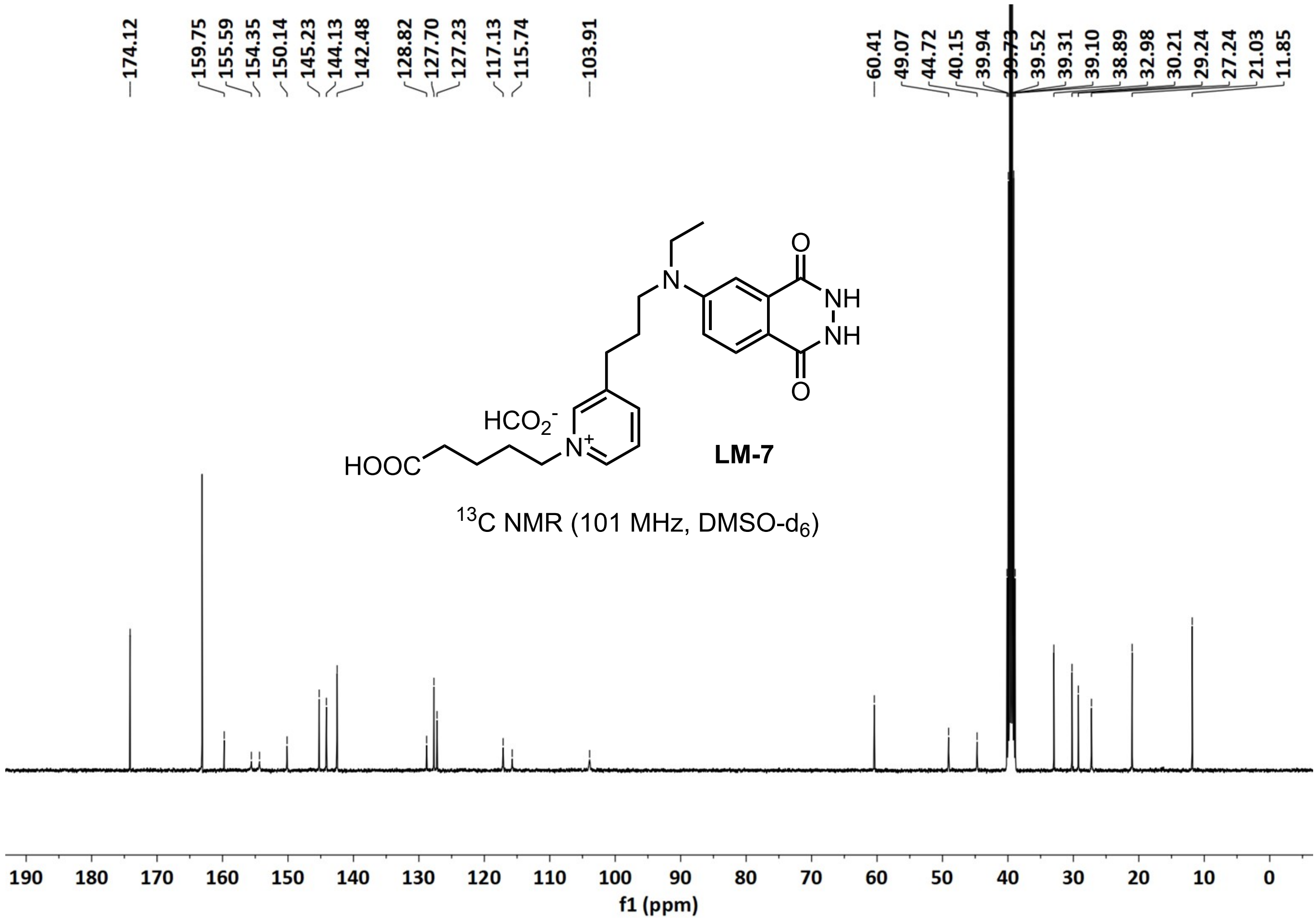
174.12
159.75 155.59 154.35 150.14 145.23 144.13 142.48
128.82 127.70 127.23 117.13 115.74
103.91
60.41 49.07 44.72 40.15 39.94 39.73 39.52 39.31 39.10 38.89 32.98 30.21 29.24 27.24 21.03 11.85
O
NH
NH
O
N
HCO2-
N+
HOOC
LM-7
13C NMR (101 MHz, DMSO-d6)
190 180 170 160 150 140 130 120 110 100 90 80 70 60 50 40 30 20 10 0
f1 (ppm)

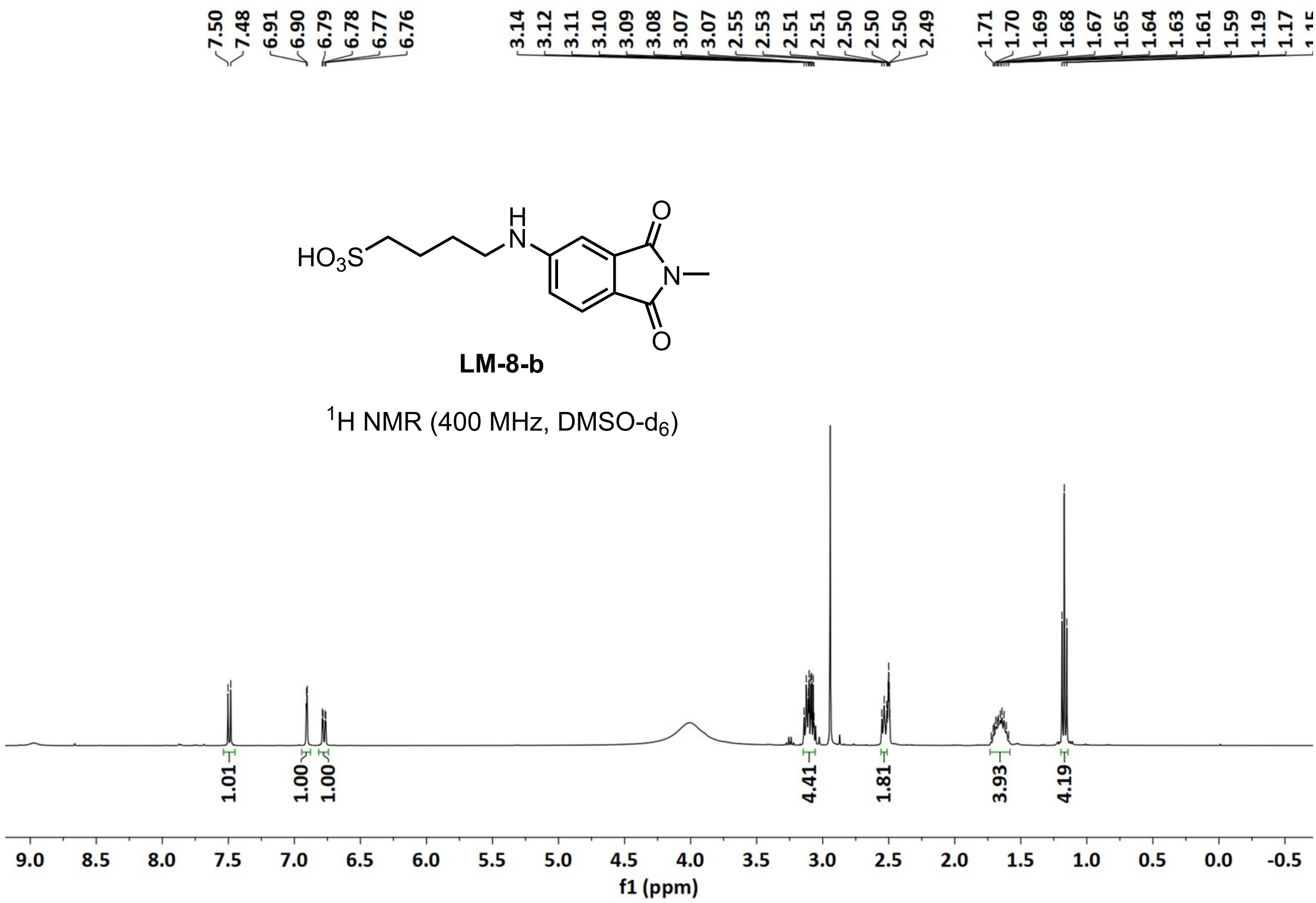
7.50
7.48
6.91
6.90
6.79
6.78
6.77
6.76
3.14
3.12
3.11
3.10
3.09
3.08
3.07
3.07
2.55
2.53
2.51
2.51
2.50
2.50
2.50
2.49
1.71
1.70
1.69
1.68
1.67
1.65
1.64
1.63
1.61
1.59
1.19
1.17
HO3S
H
N
O
N
O
LM-8-b
1H NMR (400 MHz, DMSO-d6)
1.01
1.00
1.00
4.41
1.81
3.93
4.19
f1 (ppm)

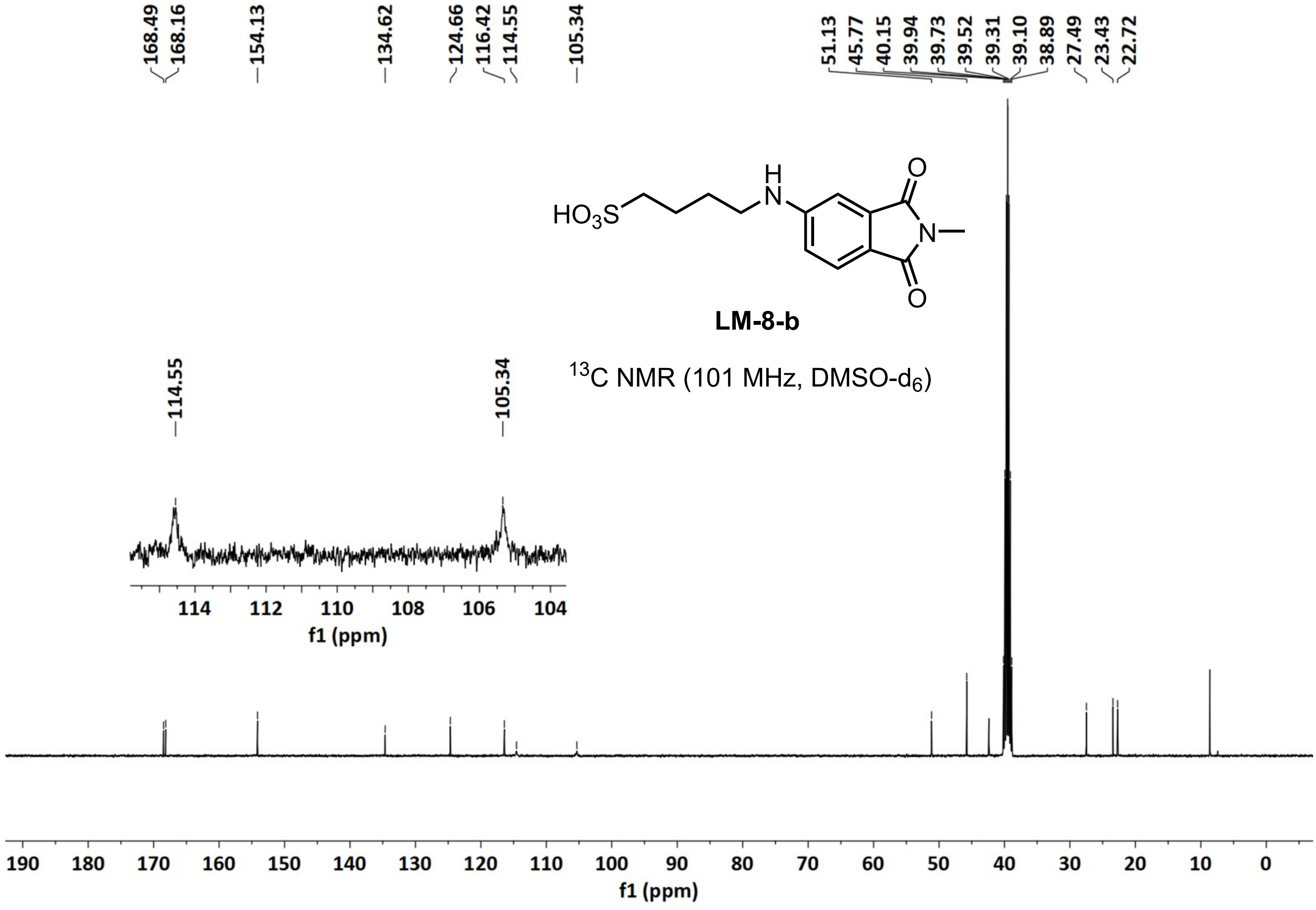
168.49
168.16
154.13
134.62
124.66
116.42
114.55
105.34
51.13
45.77
40.15
39.94
39.73
39.52
39.31
39.10
38.89
27.49
23.43
22.72
HO3S
H
N
O
N
O
LM-8-b
13C NMR (101 MHz, DMSO-d6)
114.55
105.34
f1 (ppm)
f1 (ppm)

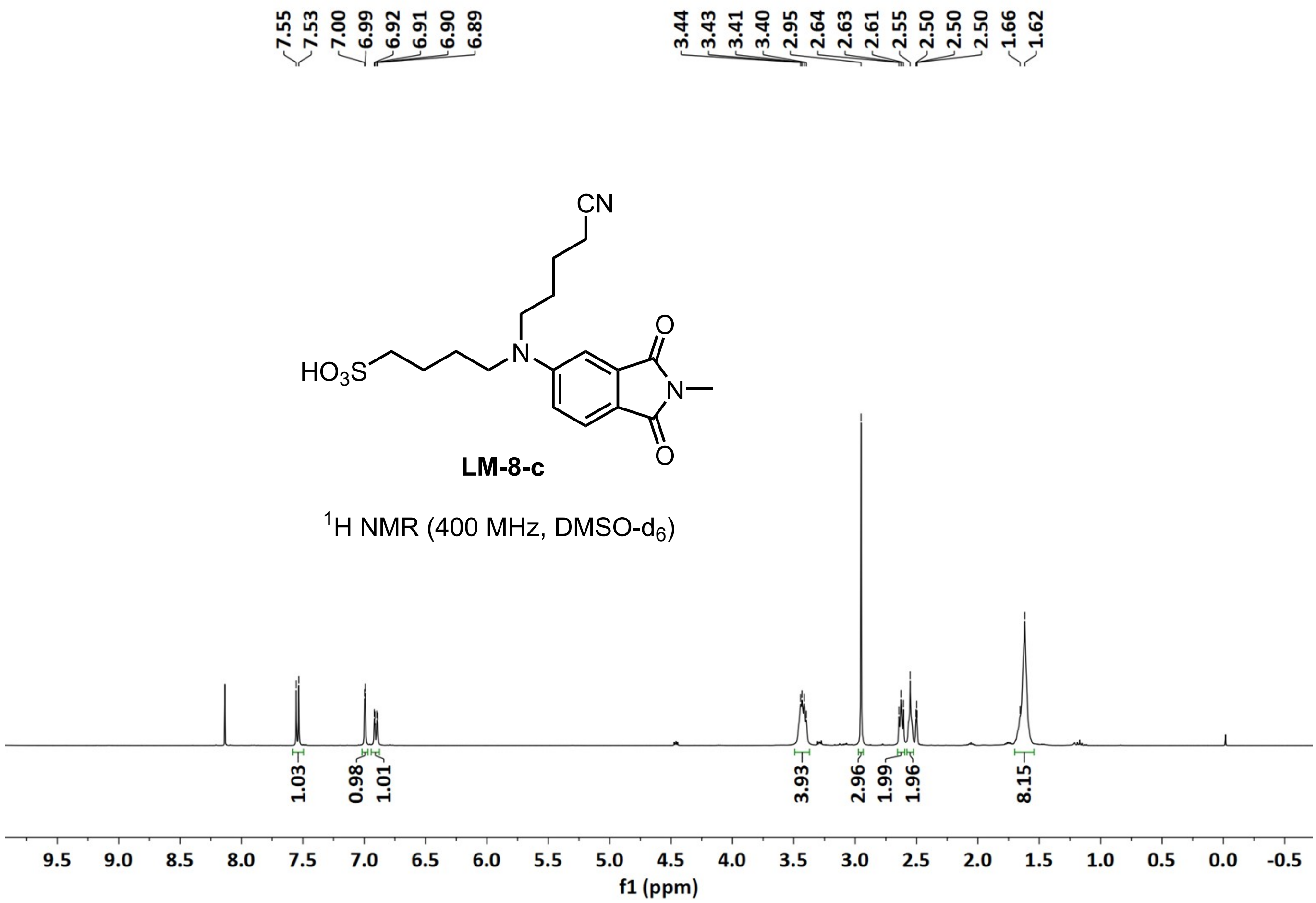
7.55
7.53
7.00
6.99
6.92
6.91
6.90
6.89
3.44
3.43
3.41
3.40
2.95
2.64
2.63
2.61
2.55
2.50
2.50
2.50
1.66
1.62
CN
O
HO_3S
N
N
O
LM-8-c
^1H NMR (400 MHz, DMSO-d_6)
1.03
0.98
1.01
3.93
2.96
1.99
1.96
8.15
9.5 9.0 8.5 8.0 7.5 7.0 6.5 6.0 5.5 5.0 4.5 4.0 3.5 3.0 2.5 2.0 1.5 1.0 0.5 0.0 -0.5
f1 (ppm)

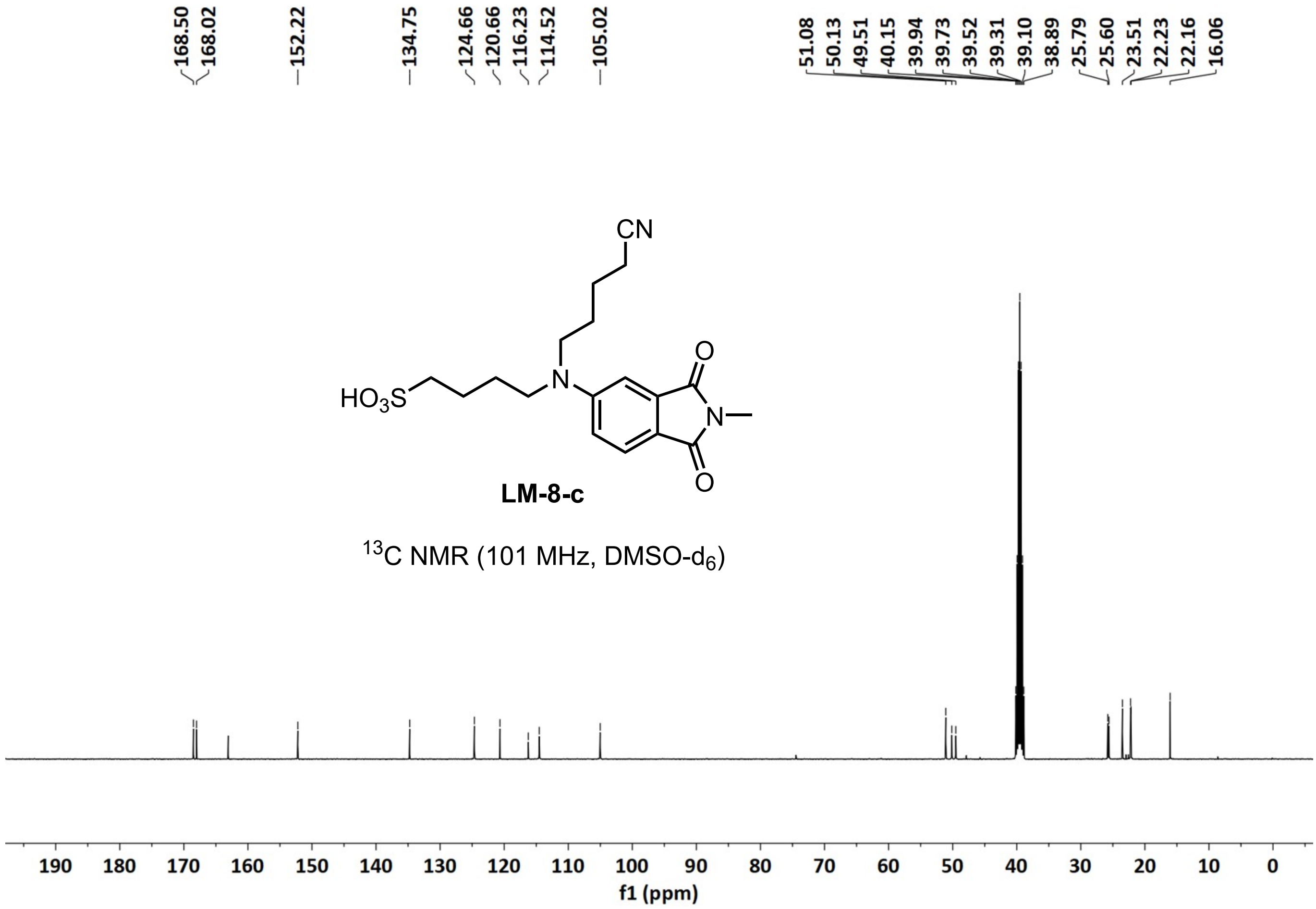
168.50
168.02
152.22
134.75
124.66
120.66
116.23
114.52
105.02
51.08
50.13
49.51
40.15
39.94
39.73
39.52
39.31
39.10
38.89
25.79
25.60
23.51
22.23
22.16
16.06
CN
O
HO_3S
N
N
O
LM-8-c
^{13}C NMR (101 MHz, DMSO-d_6)
190 180 170 160 150 140 130 120 110 100 90 80 70 60 50 40 30 20 10 0
f1 (ppm)

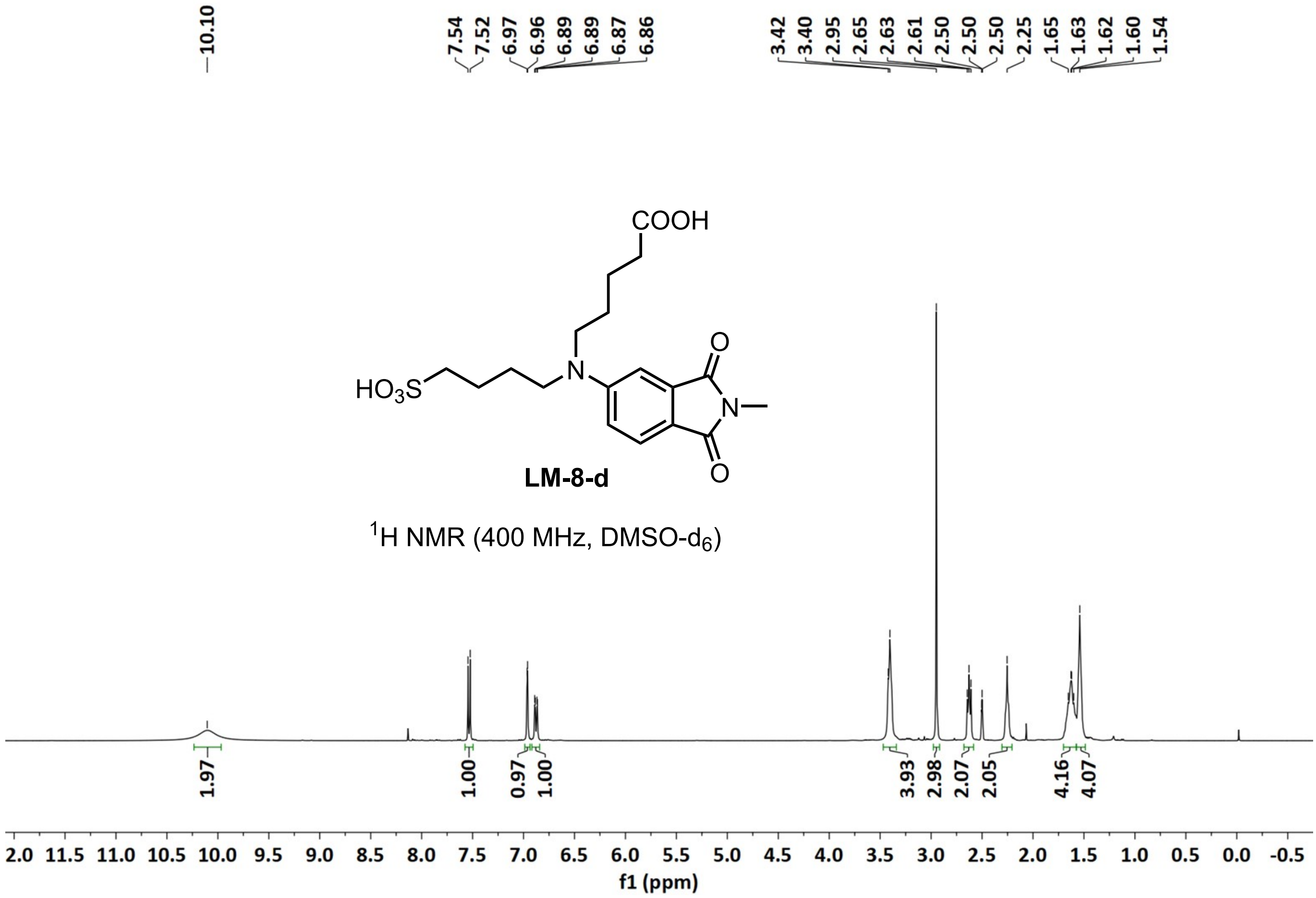
10.10
7.54
7.52
6.97
6.96
6.89
6.89
6.87
6.86
3.42
3.40
2.95
2.65
2.63
2.61
2.50
2.50
2.50
2.25
1.65
1.63
1.62
1.60
1.54
COOH
HO3S
N
O
N
O
LM-8-d
1H NMR (400 MHz, DMSO-d6)
1.97
1.00
0.97
1.00
3.93
2.98
2.07
2.05
4.16
4.07
f1 (ppm)

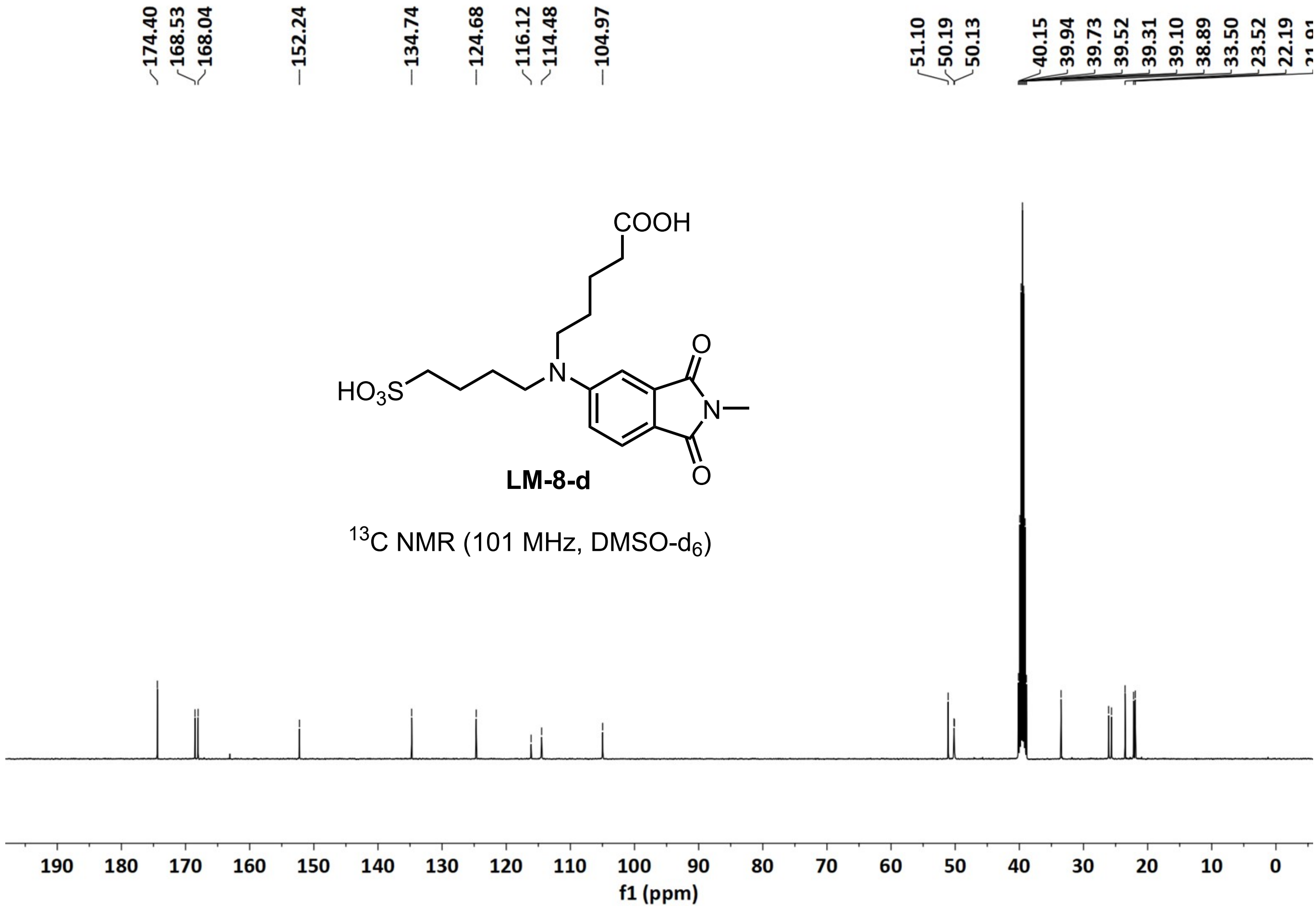
174.40
168.53
168.04
152.24
134.74
124.68
116.12
114.48
104.97
51.10
50.19
50.13
40.15
39.94
39.73
39.52
39.31
39.10
38.89
33.50
23.52
22.19
COOH
HO3S
N
O
N
O
LM-8-d
13C NMR (101 MHz, DMSO-d6)
f1 (ppm)

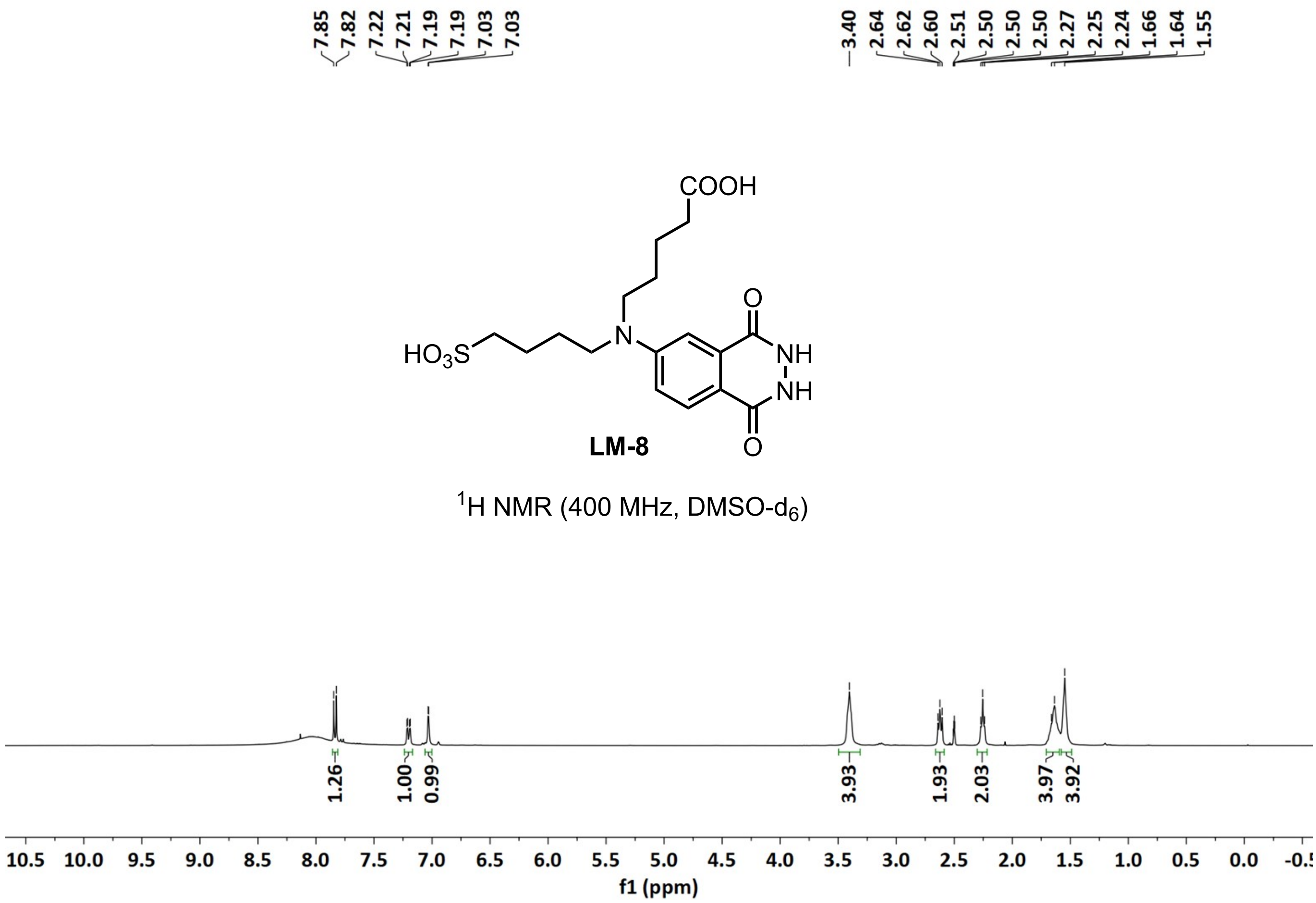
7.85
7.82
7.22
7.21
7.19
7.19
7.03
7.03
3.40
2.64
2.62
2.60
2.51
2.50
2.50
2.50
2.27
2.25
2.24
1.66
1.64
1.55
COOH
O
HO3S
N
NH
NH
O
LM-8
1H NMR (400 MHz, DMSO-d6)
1.26
1.00
0.99
3.93
1.93
2.03
3.97
3.92
10.5 10.0 9.5 9.0 8.5 8.0 7.5 7.0 6.5 6.0 5.5 5.0 4.5 4.0 3.5 3.0 2.5 2.0 1.5 1.0 0.5 0.0
f1 (ppm)

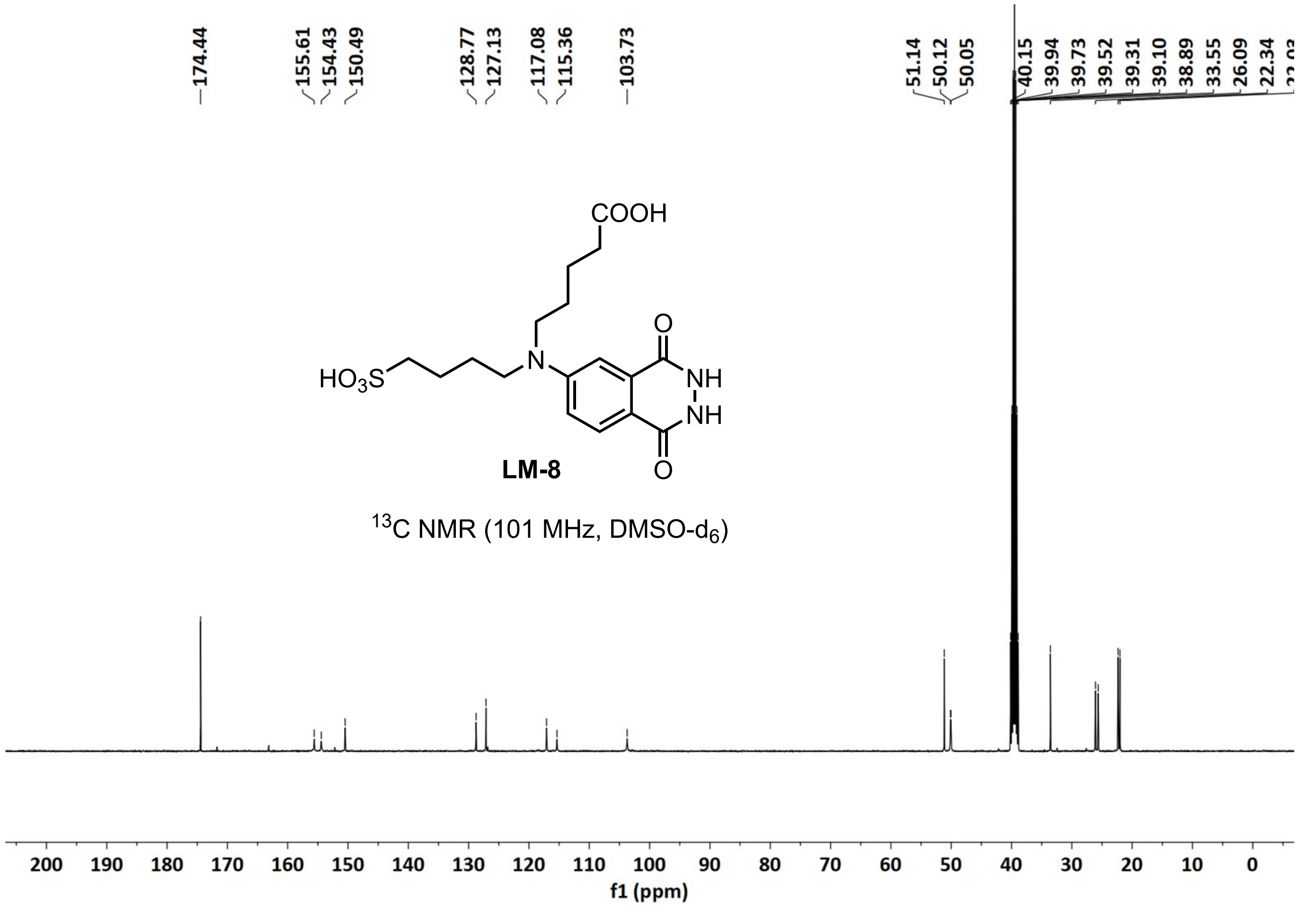
174.44
155.61
154.43
150.49
128.77
127.13
117.08
115.36
103.73
51.14
50.12
50.05
40.15
39.94
39.73
39.52
39.31
39.10
38.89
33.55
26.09
22.34
COOH
O
HO3S
N
NH
NH
O
LM-8
13C NMR (101 MHz, DMSO-d6)
200 190 180 170 160 150 140 130 120 110 100 90 80 70 60 50 40 30 20 10 0
f1 (ppm)

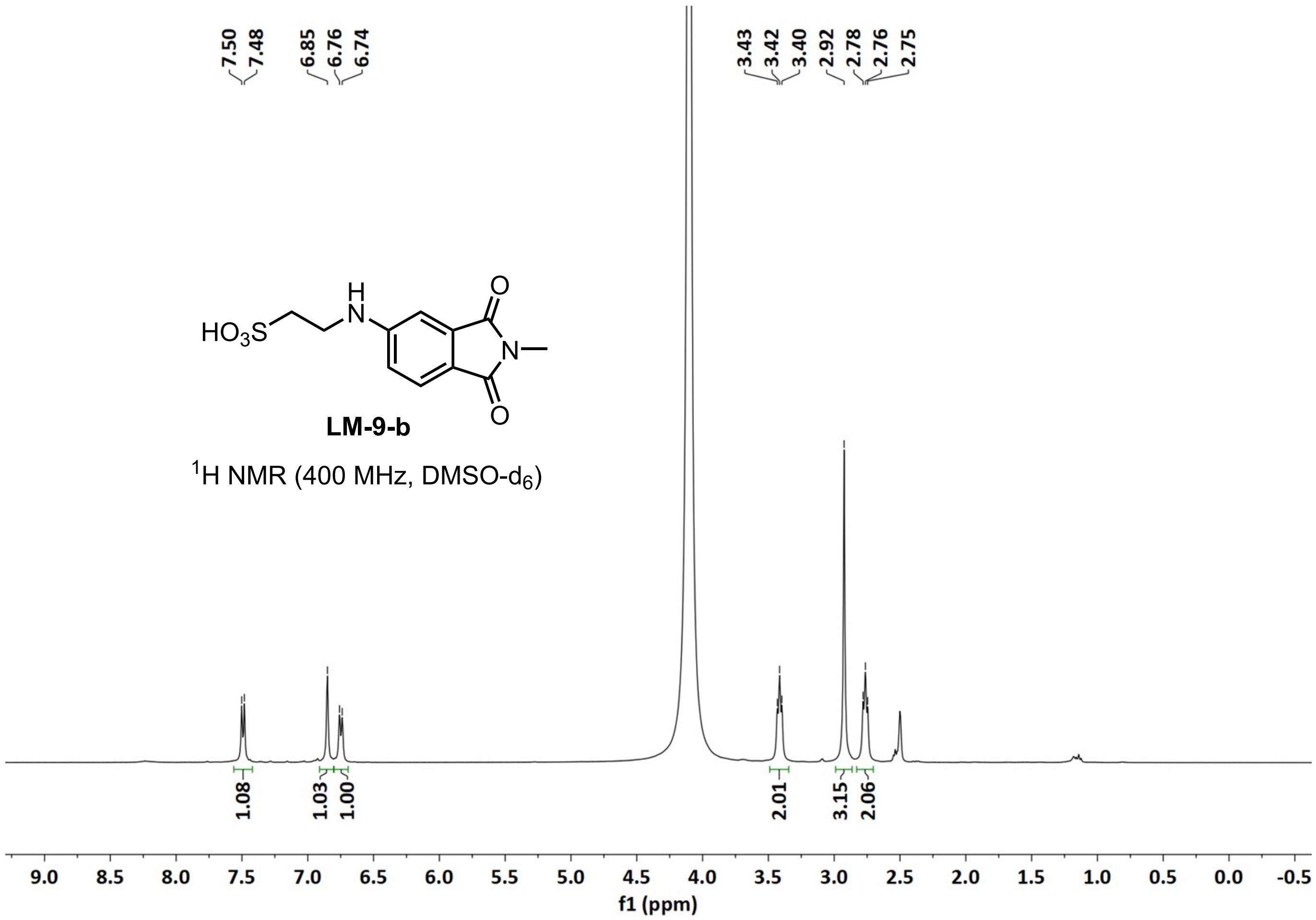
7.50
7.48
6.85
6.76
6.74
3.43
3.42
3.40
2.92
2.78
2.76
2.75
HO3S
H
N
O
N
O
LM-9-b
1H NMR (400 MHz, DMSO-d6)
1.08
1.03
1.00
2.01
3.15
2.06
f1 (ppm)

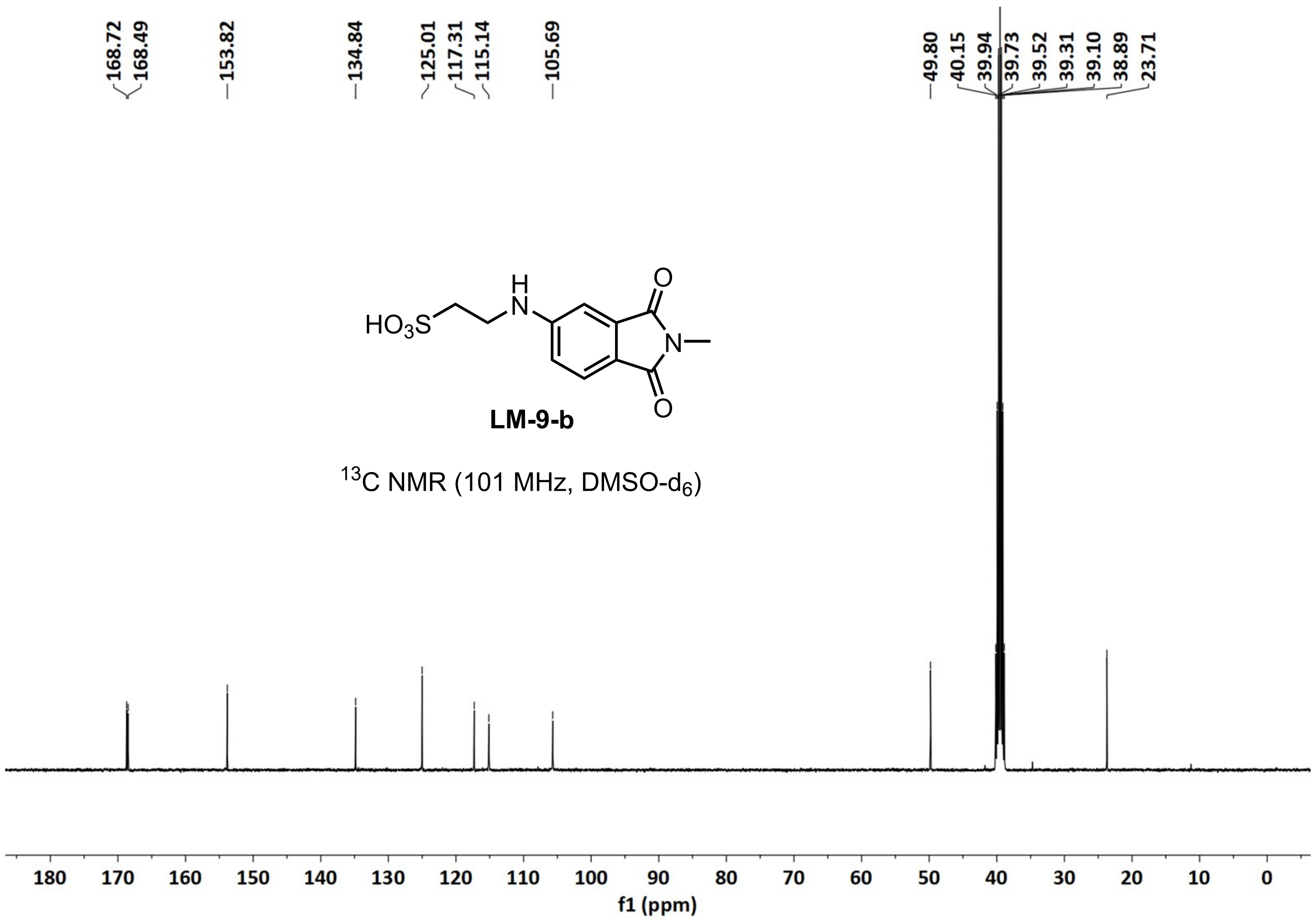
168.72
168.49
153.82
134.84
125.01
117.31
115.14
105.69
49.80
40.15
39.94
39.73
39.52
39.31
39.10
38.89
23.71
HO3S
H
N
O
N
O
LM-9-b
13C NMR (101 MHz, DMSO-d6)
f1 (ppm)

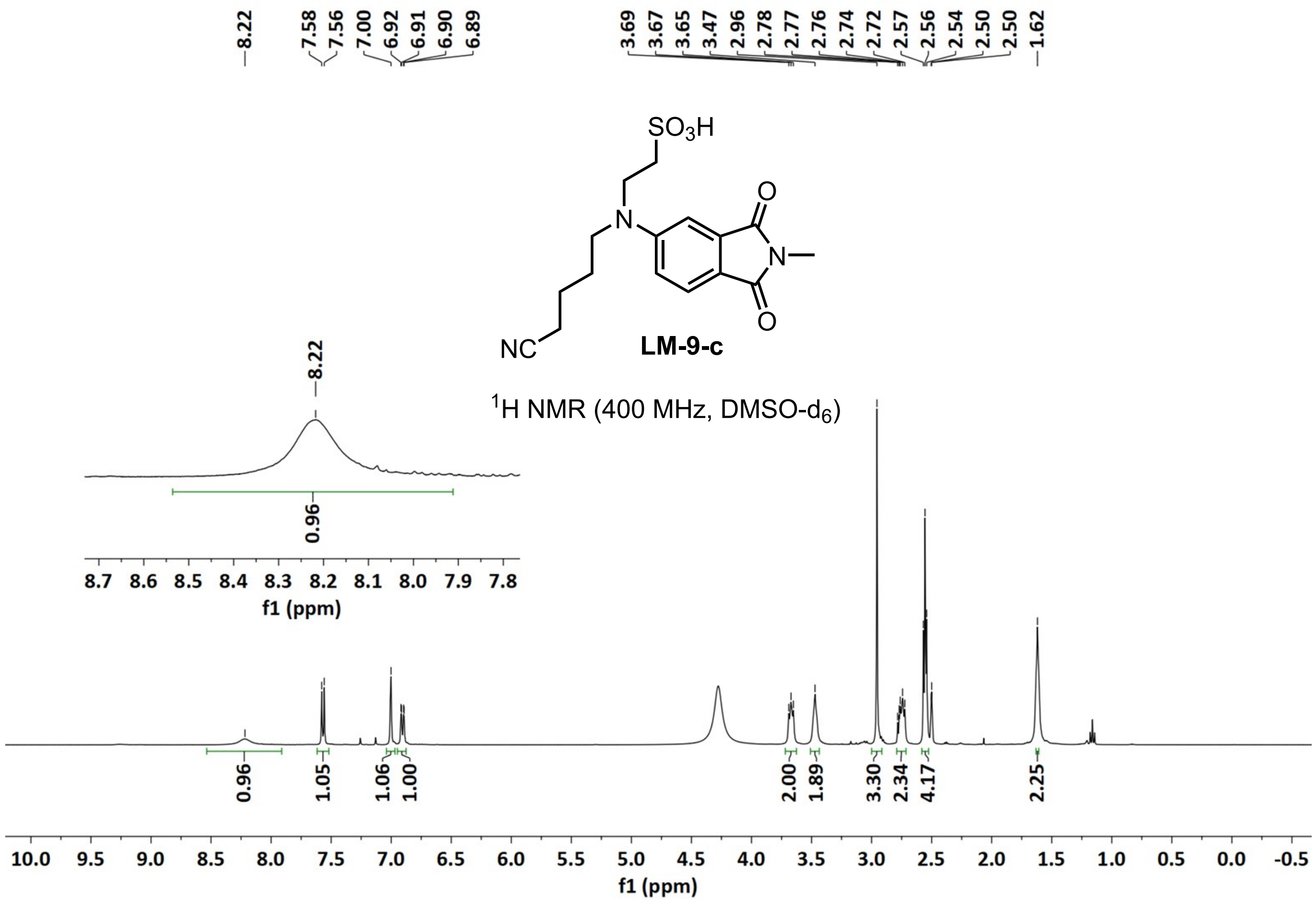
8.22
7.58
7.56
7.00
6.92
6.91
6.90
6.89
3.69
3.67
3.65
3.47
2.96
2.78
2.77
2.76
2.74
2.72
2.57
2.56
2.54
2.50
2.50
1.62
SO3H
N
O
N
O
NC
LM-9-c
1H NMR (400 MHz, DMSO-d6)
0.96
8.7 8.6 8.5 8.4 8.3 8.2 8.1 8.0 7.9 7.8
f1 (ppm)
0.96
1.05
1.06
1.00
2.00
1.89
3.30
2.34
4.17
2.25
10.0 9.5 9.0 8.5 8.0 7.5 7.0 6.5 6.0 5.5 5.0 4.5 4.0 3.5 3.0 2.5 2.0 1.5 1.0 0.5 0.0 -0.5
f1 (ppm)

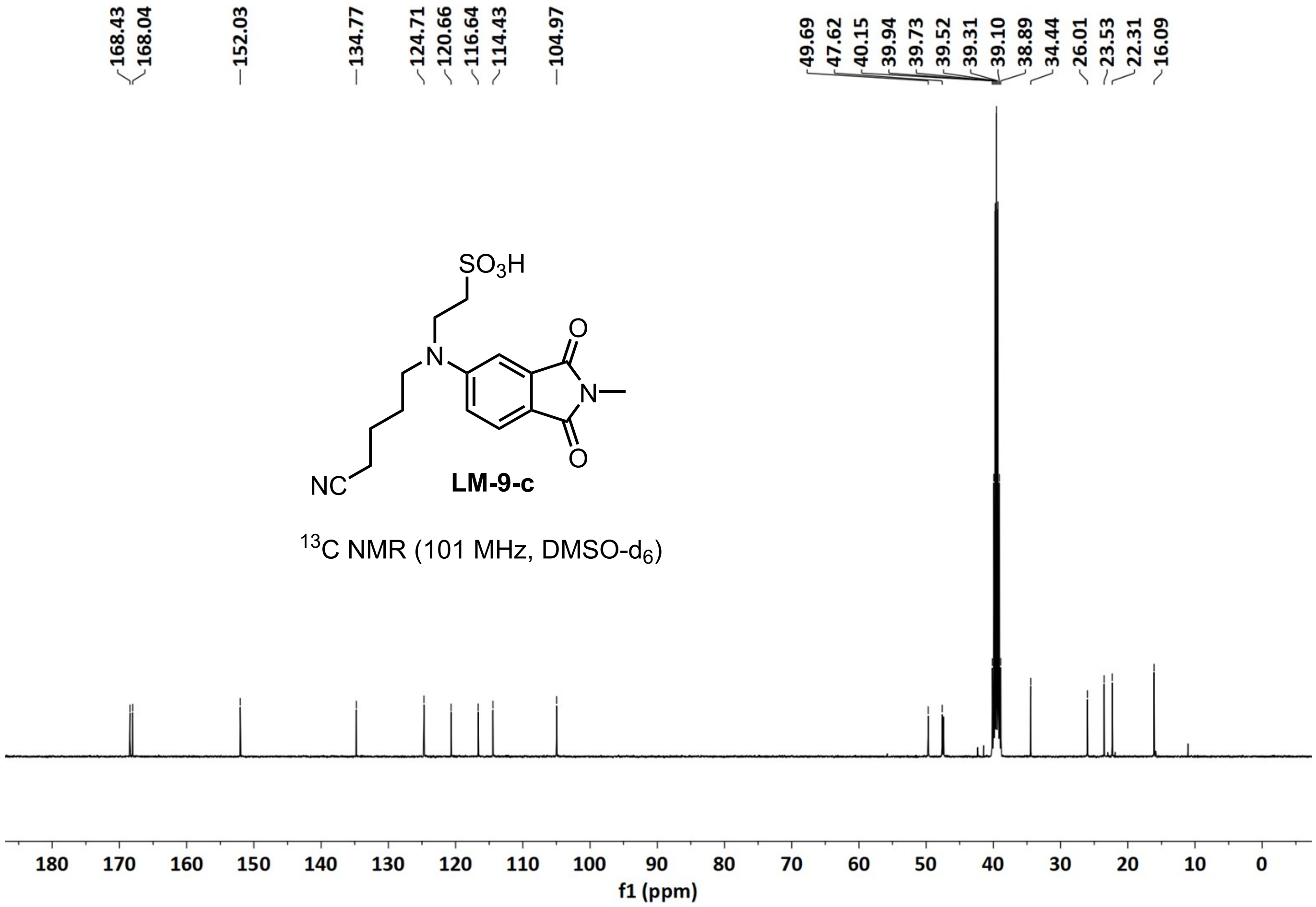
168.43
168.04
152.03
134.77
124.71
120.66
116.64
114.43
104.97
49.69
47.62
40.15
39.94
39.73
39.52
39.31
39.10
38.89
34.44
26.01
23.53
22.31
16.09
SO3H
N
O
N
O
NC
LM-9-c
13C NMR (101 MHz, DMSO-d6)
180 170 160 150 140 130 120 110 100 90 80 70 60 50 40 30 20 10 0
f1 (ppm)

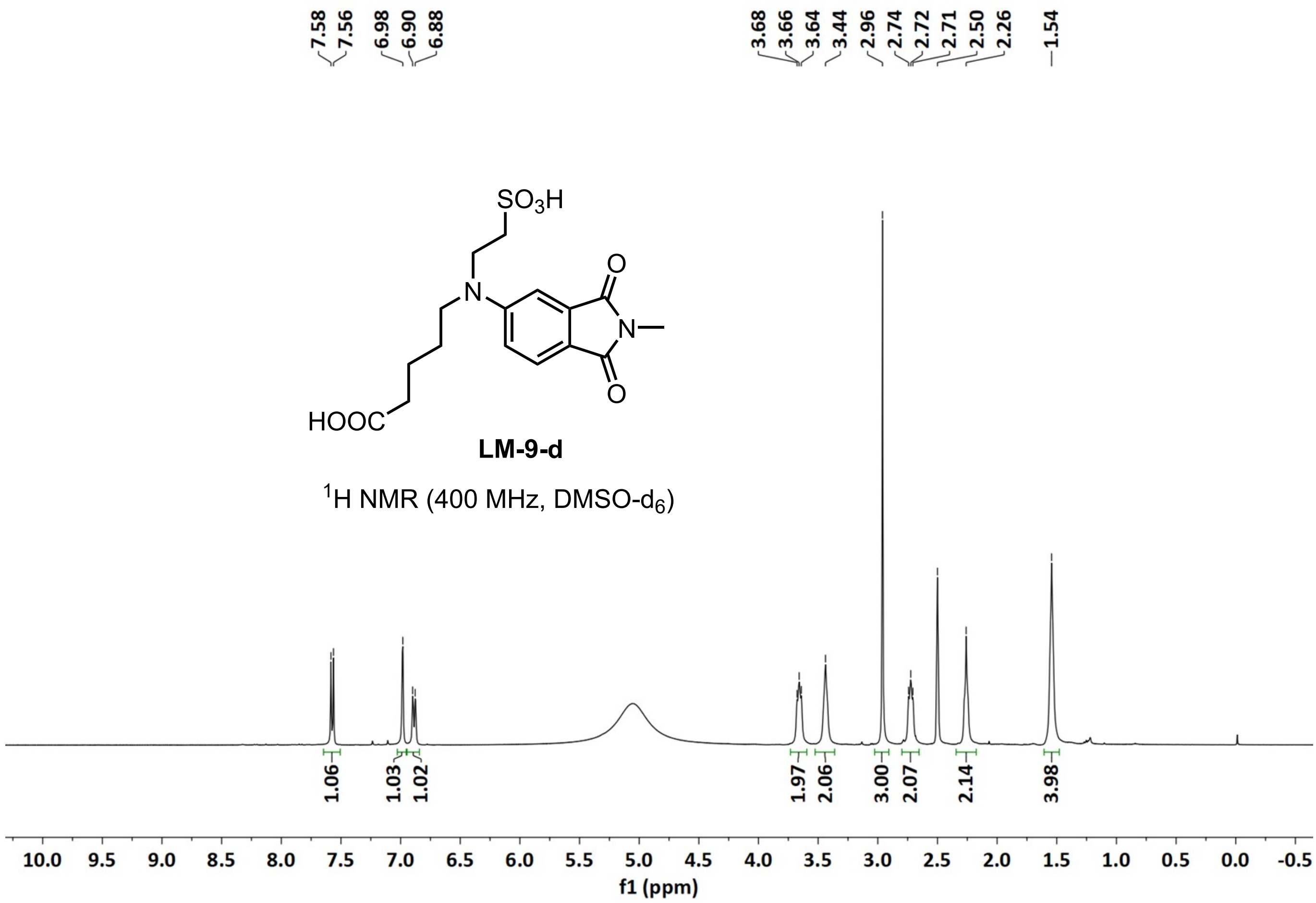
7.58
7.56
6.98
6.90
6.88
3.68
3.66
3.64
3.44
2.96
2.74
2.72
2.71
2.50
2.26
1.54
SO3H
N
O
N
O
HOOC
LM-9-d
1H NMR (400 MHz, DMSO-d6)
1.06
1.03
1.02
1.97
2.06
3.00
2.07
2.14
3.98
10.0 9.5 9.0 8.5 8.0 7.5 7.0 6.5 6.0 5.5 5.0 4.5 4.0 3.5 3.0 2.5 2.0 1.5 1.0 0.5 0.0 -0.5
f1 (ppm)

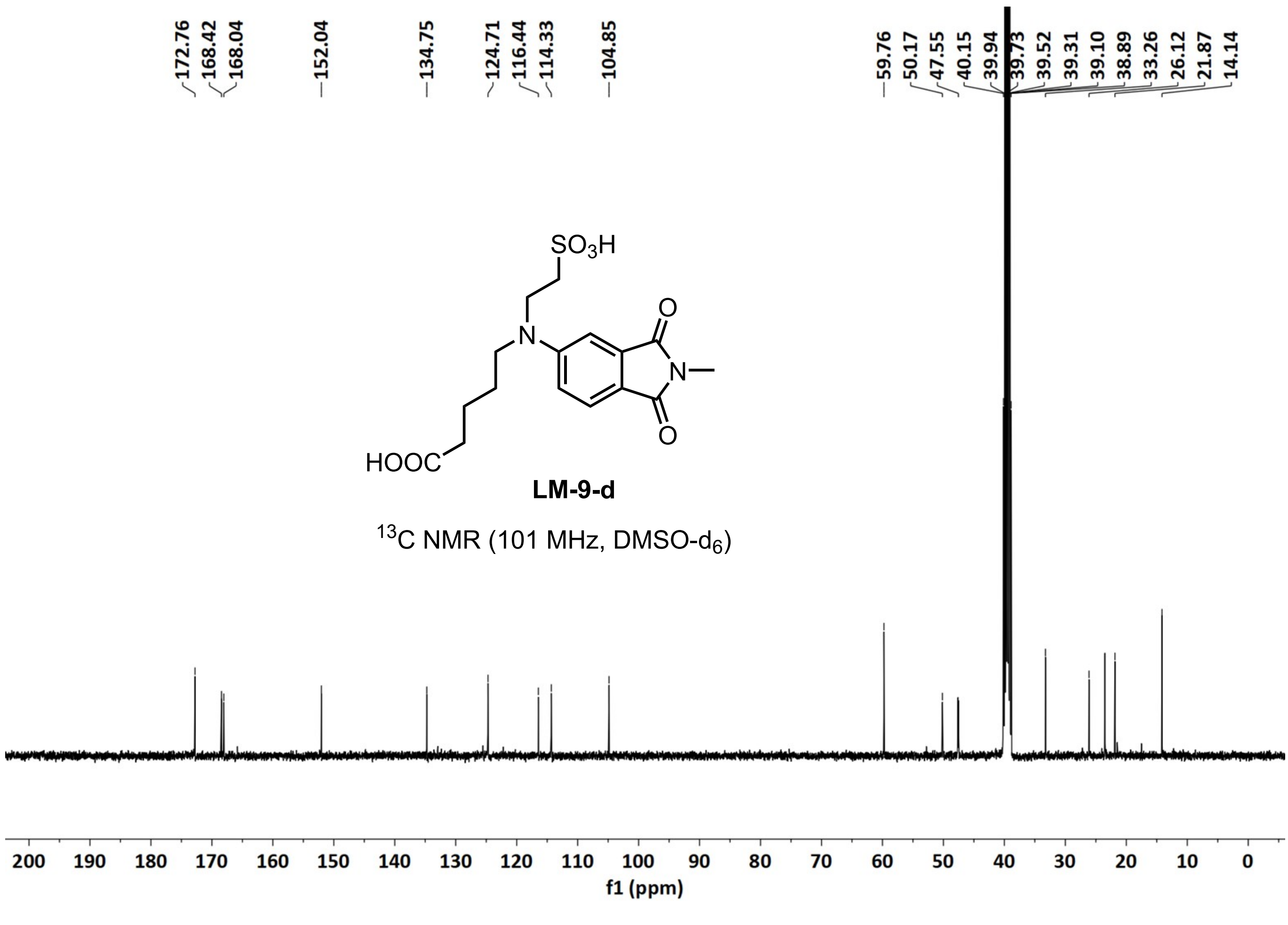
172.76
168.42
168.04
152.04
134.75
124.71
116.44
114.33
104.85
59.76
50.17
47.55
40.15
39.94
39.73
39.52
39.31
39.10
38.89
33.26
26.12
21.87
14.14
SO3H
N
O
N
O
HOOC
LM-9-d
13C NMR (101 MHz, DMSO-d6)
200 190 180 170 160 150 140 130 120 110 100 90 80 70 60 50 40 30 20 10 0
f1 (ppm)

LM-9

^1H NMR (400 MHz, $DMSO-d_6$)

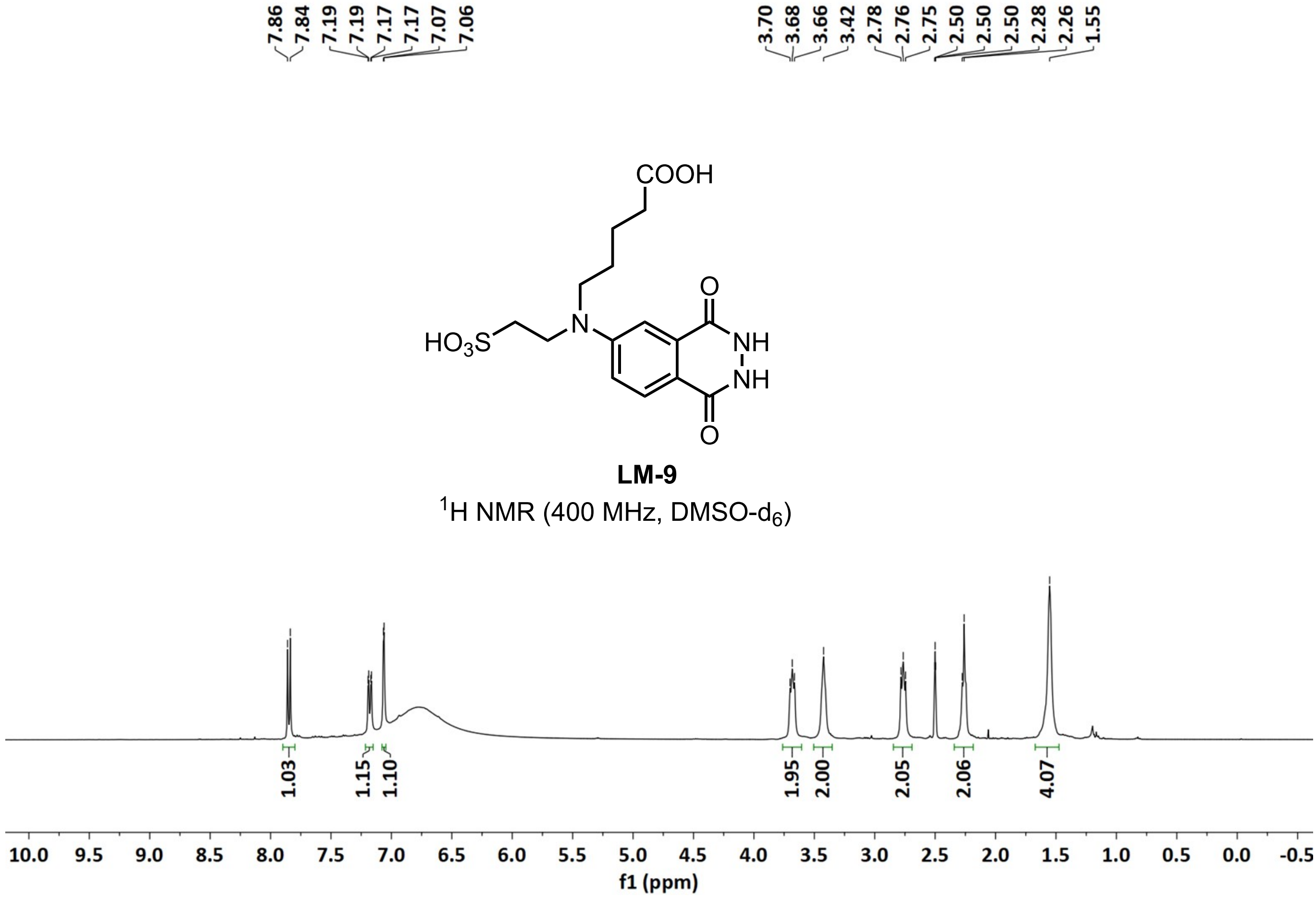

LM-9

^{13}C NMR (101 MHz, $DMSO-d_6$)

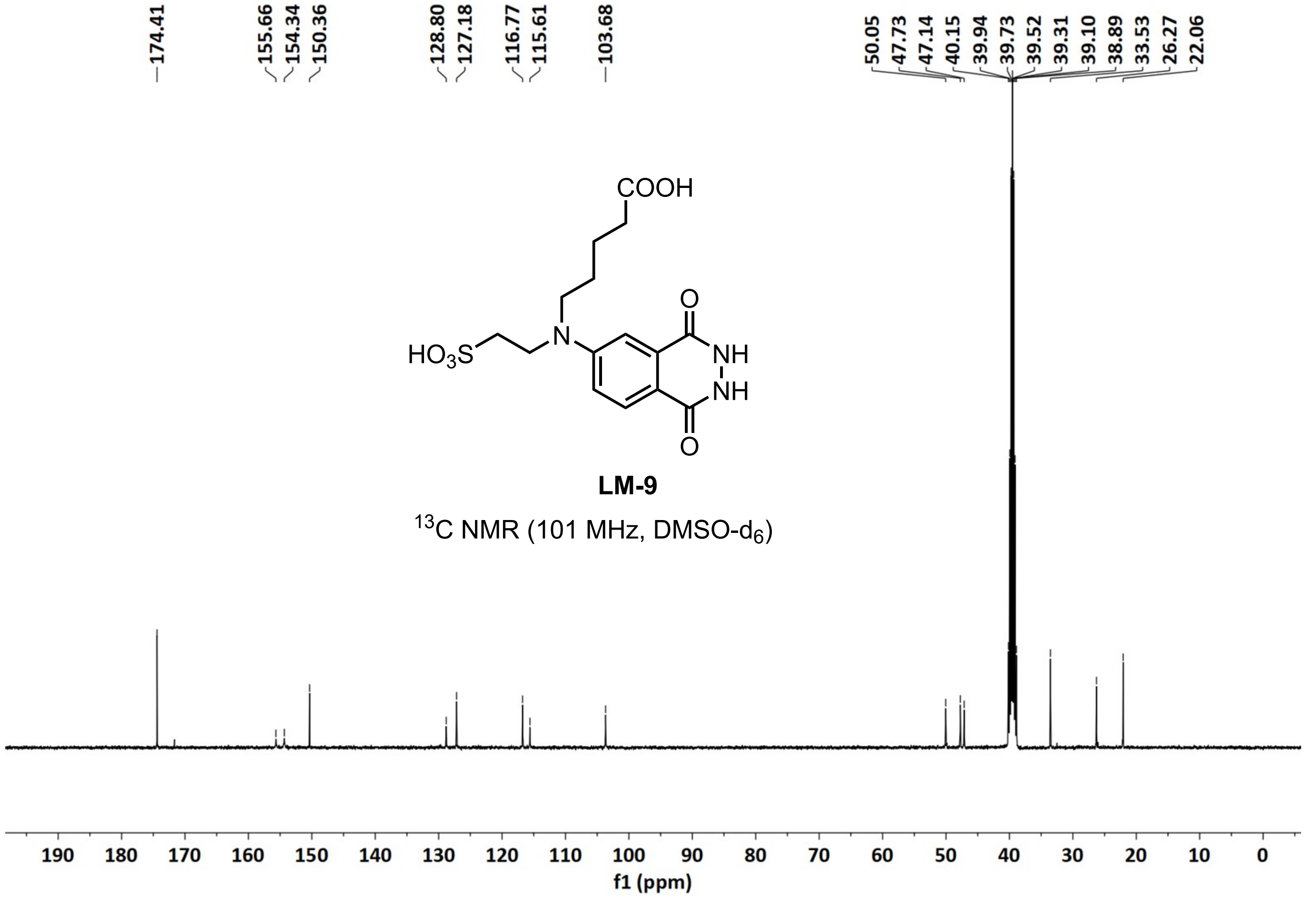

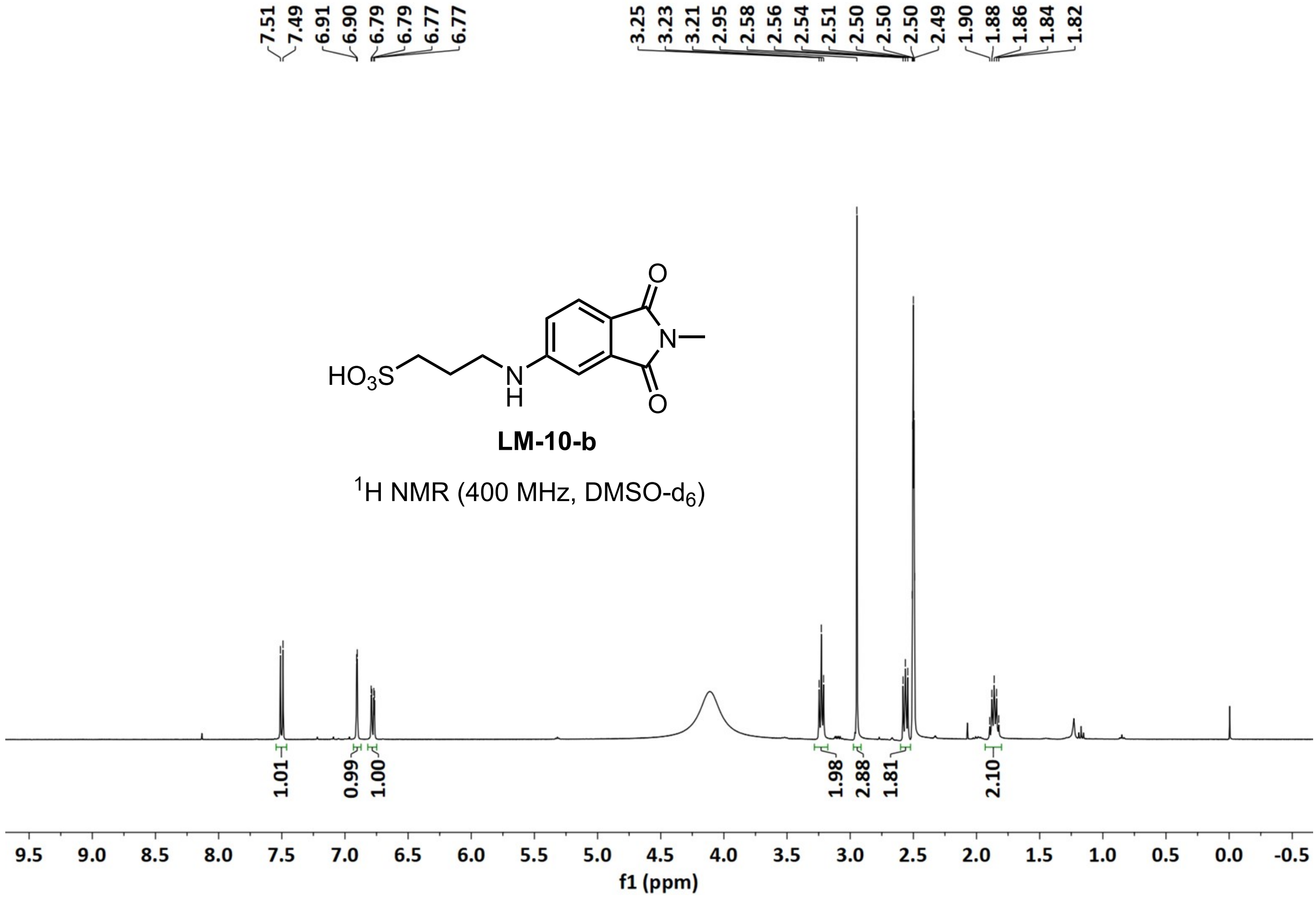

LM-10-b

^{1}H NMR (400 MHz, DMSO-d$_6$)

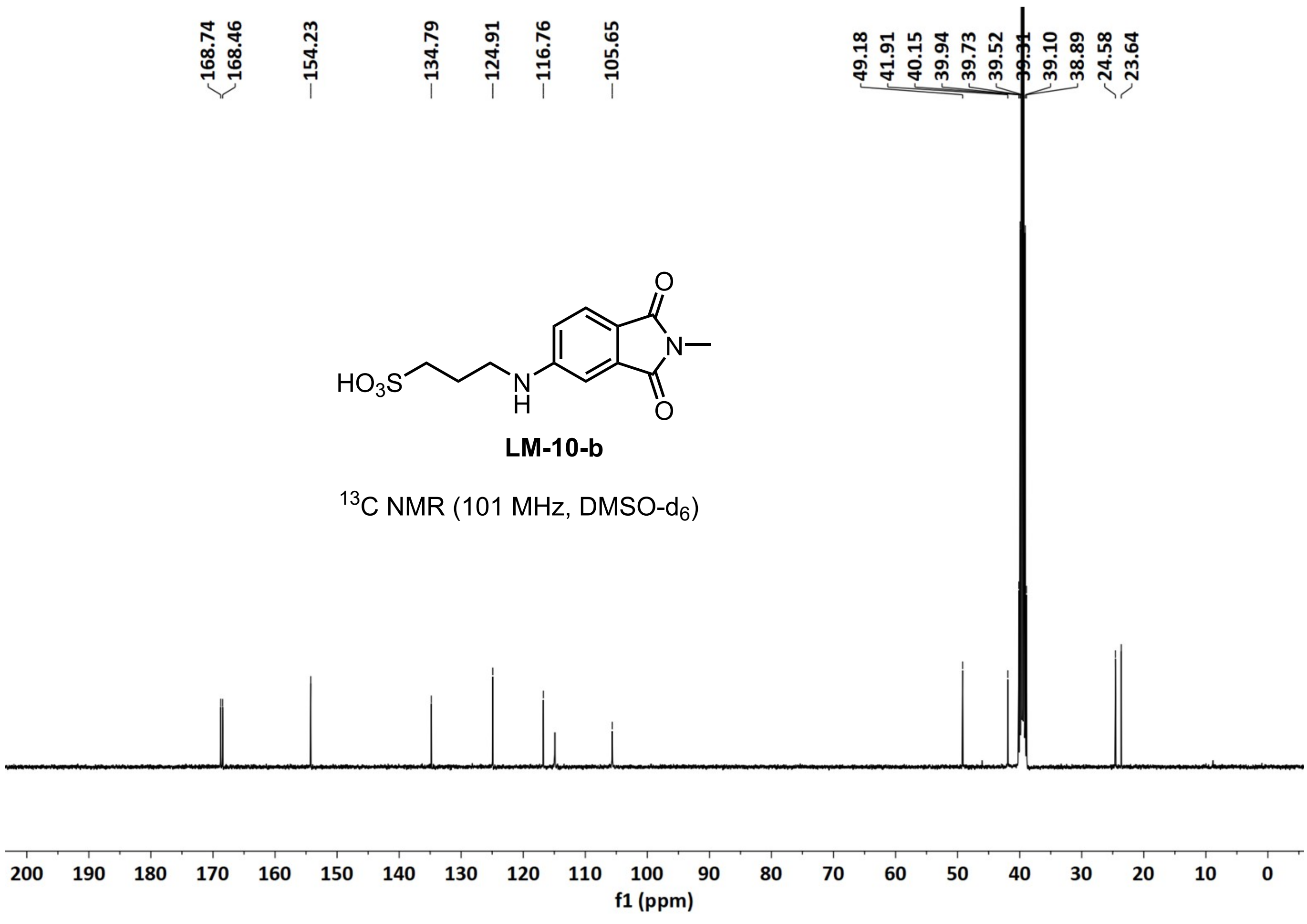

LM-10-b

^{13}C NMR (101 MHz, DMSO-d$_6$)

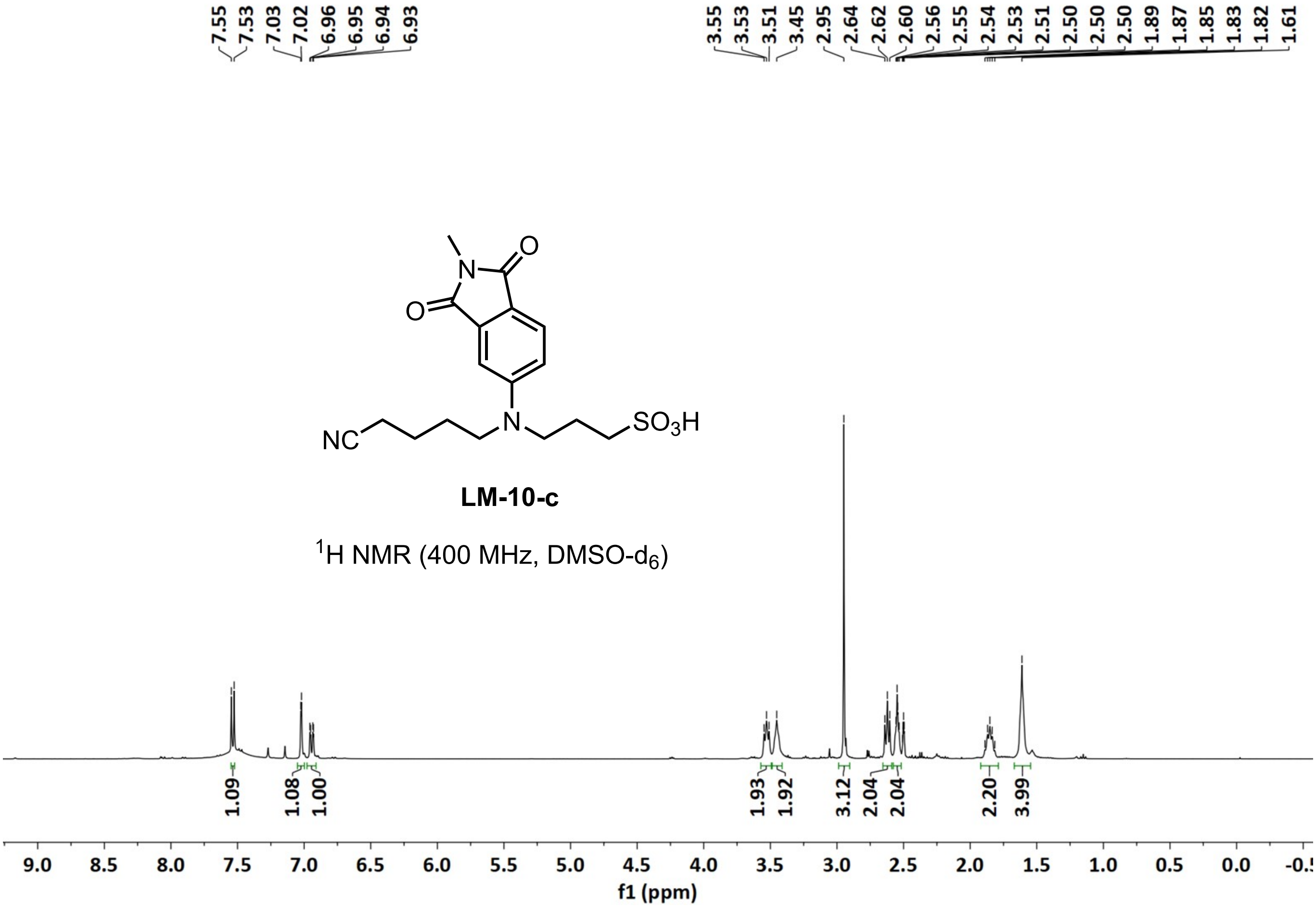
7.55
7.53
7.03
7.02
6.96
6.95
6.94
6.93
3.55
3.53
3.51
3.45
2.95
2.64
2.62
2.60
2.56
2.55
2.54
2.53
2.51
2.50
2.50
2.50
1.89
1.87
1.85
1.83
1.82
1.61
NC
N
SO$_3$H
O
O
N
LM-10-c
^{1}H NMR (400 MHz, DMSO-d$_6$)
1.09
1.08
1.00
1.93
1.92
3.12
2.04
2.04
2.20
3.99
f1 (ppm)

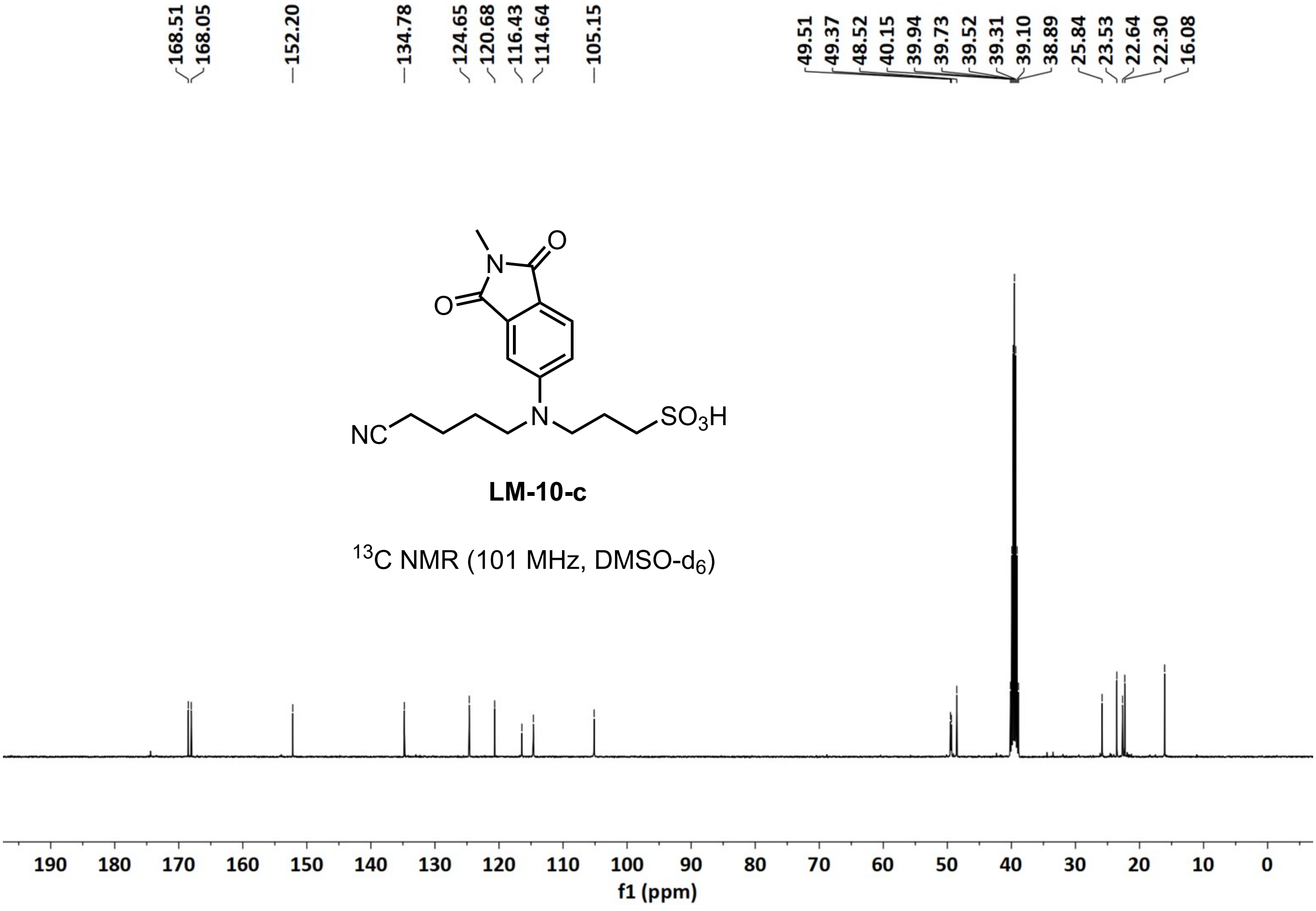
168.51
168.05
152.20
134.78
124.65
120.68
116.43
114.64
105.15
49.51
49.37
48.52
40.15
39.94
39.73
39.52
39.31
39.10
38.89
25.84
23.53
22.64
22.30
16.08
NC
N
SO$_3$H
O
O
N
LM-10-c
^{13}C NMR (101 MHz, DMSO-d$_6$)
f1 (ppm)

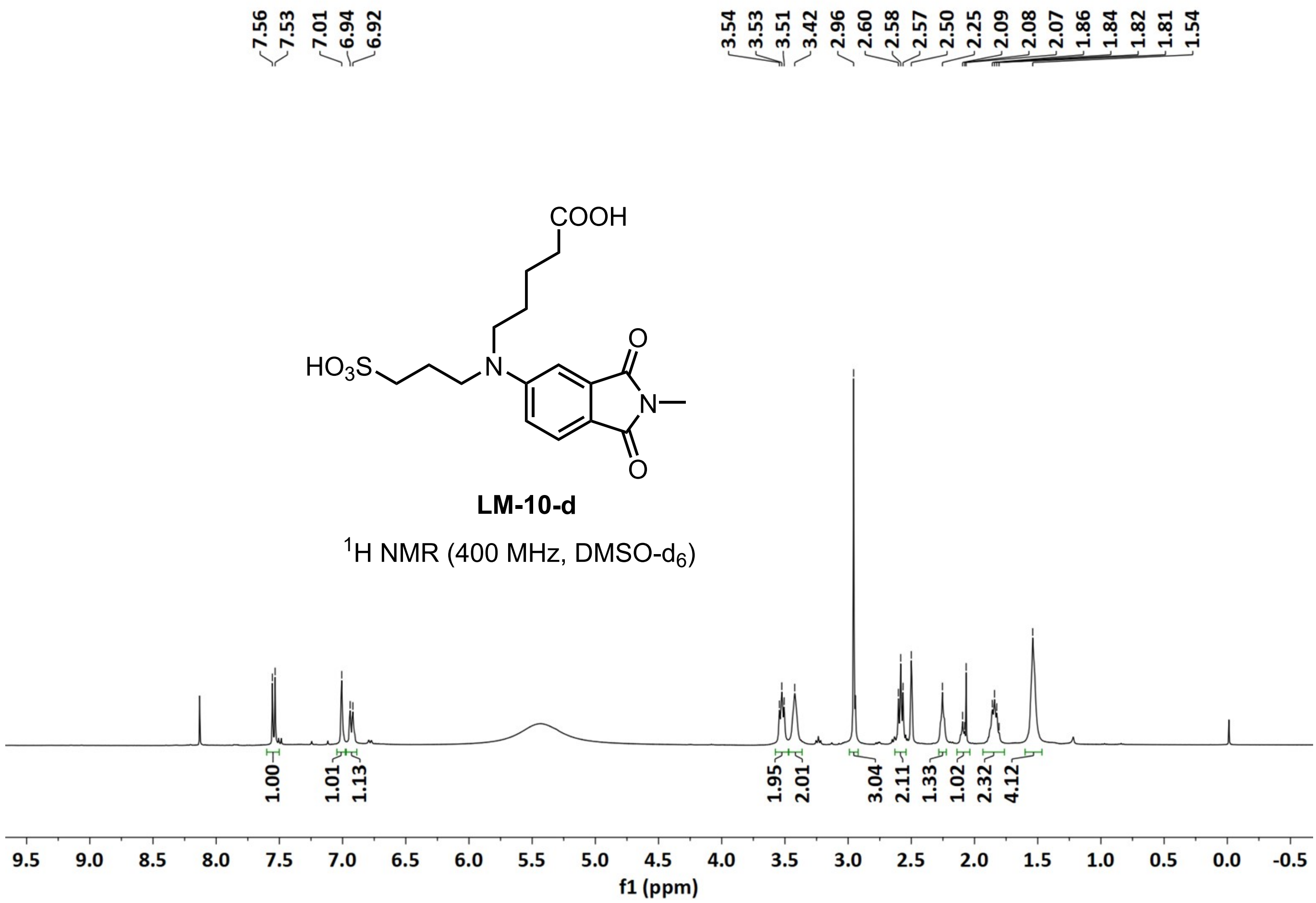

7.56
7.53
7.01
6.94
6.92
3.54
3.53
3.51
3.42
2.96
2.60
2.58
2.57
2.50
2.25
2.09
2.08
2.07
1.86
1.84
1.82
1.81
1.54
COOH
HO_3S
N
O
O
N
LM-10-d
^1H NMR (400 MHz, DMSO-d_6)
1.00
1.01
1.13
1.95
2.01
3.04
2.11
1.33
1.02
2.32
4.12
f1 (ppm)

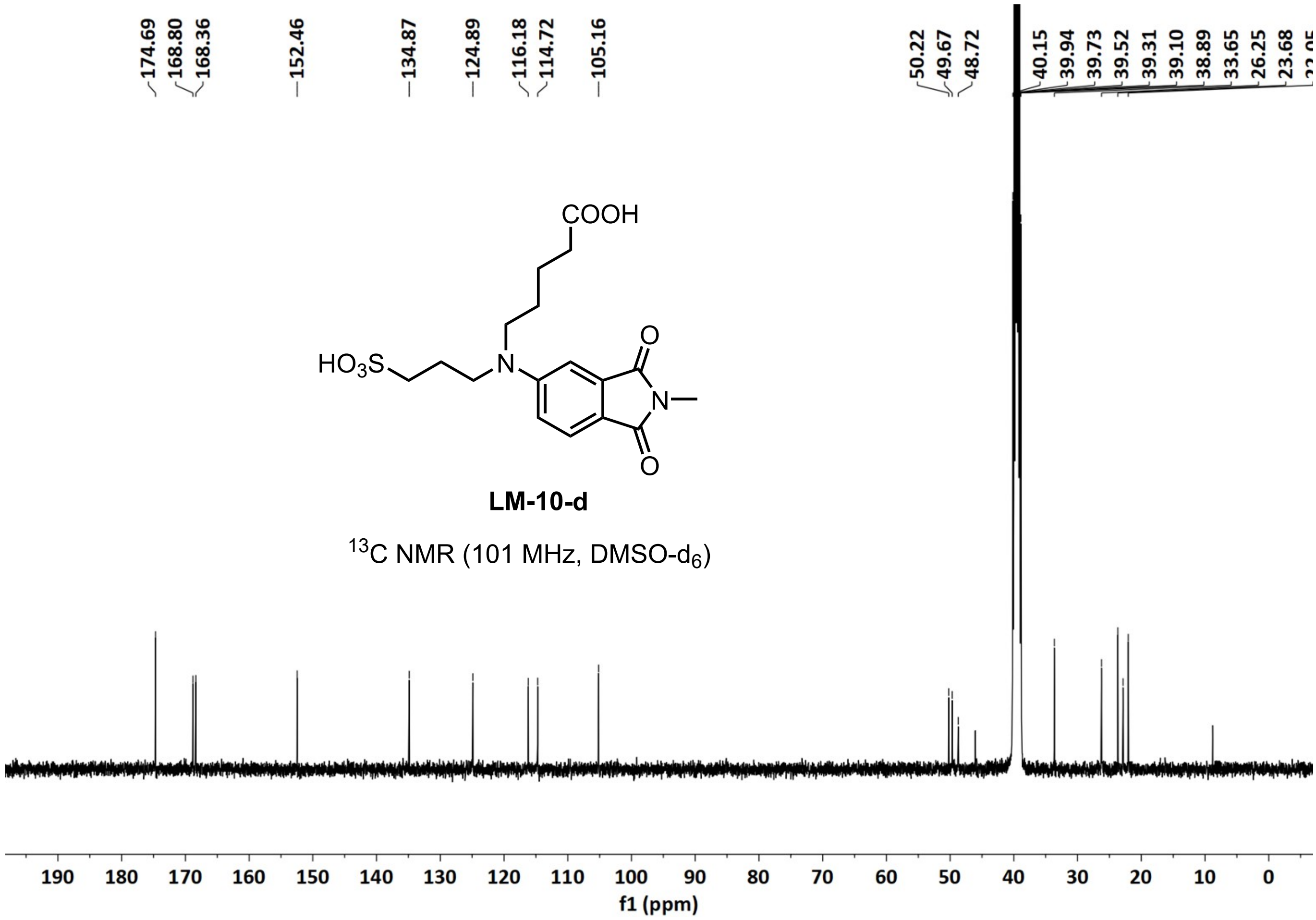

174.69
168.80
168.36
152.46
134.87
124.89
116.18
114.72
105.16
50.22
49.67
48.72
40.15
39.94
39.73
39.52
39.31
39.10
38.89
33.65
26.25
23.68
COOH
HO_3S
N
O
O
N
LM-10-d
^{13}C NMR (101 MHz, DMSO-d_6)
f1 (ppm)

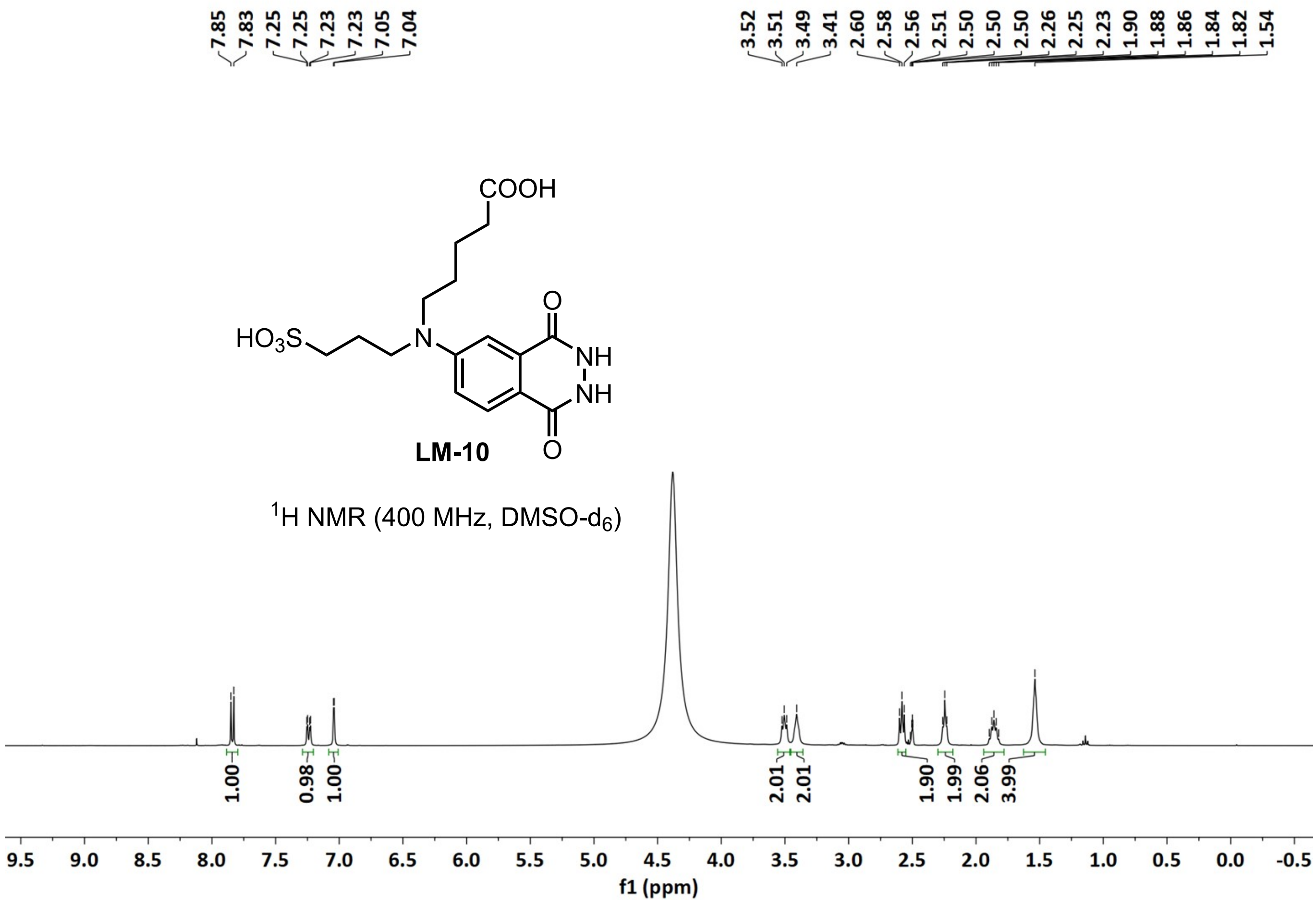
7.85
7.83
7.25
7.25
7.23
7.23
7.05
7.04
3.52
3.51
3.49
3.41
2.60
2.58
2.56
2.51
2.50
2.50
2.50
2.26
2.25
2.23
1.90
1.88
1.86
1.84
1.82
1.54
COOH
HO3S
N
O
NH
NH
O
LM-10
1H NMR (400 MHz, DMSO-d6)
1.00
0.98
1.00
2.01
2.01
1.90
1.99
2.06
3.99
f1 (ppm)

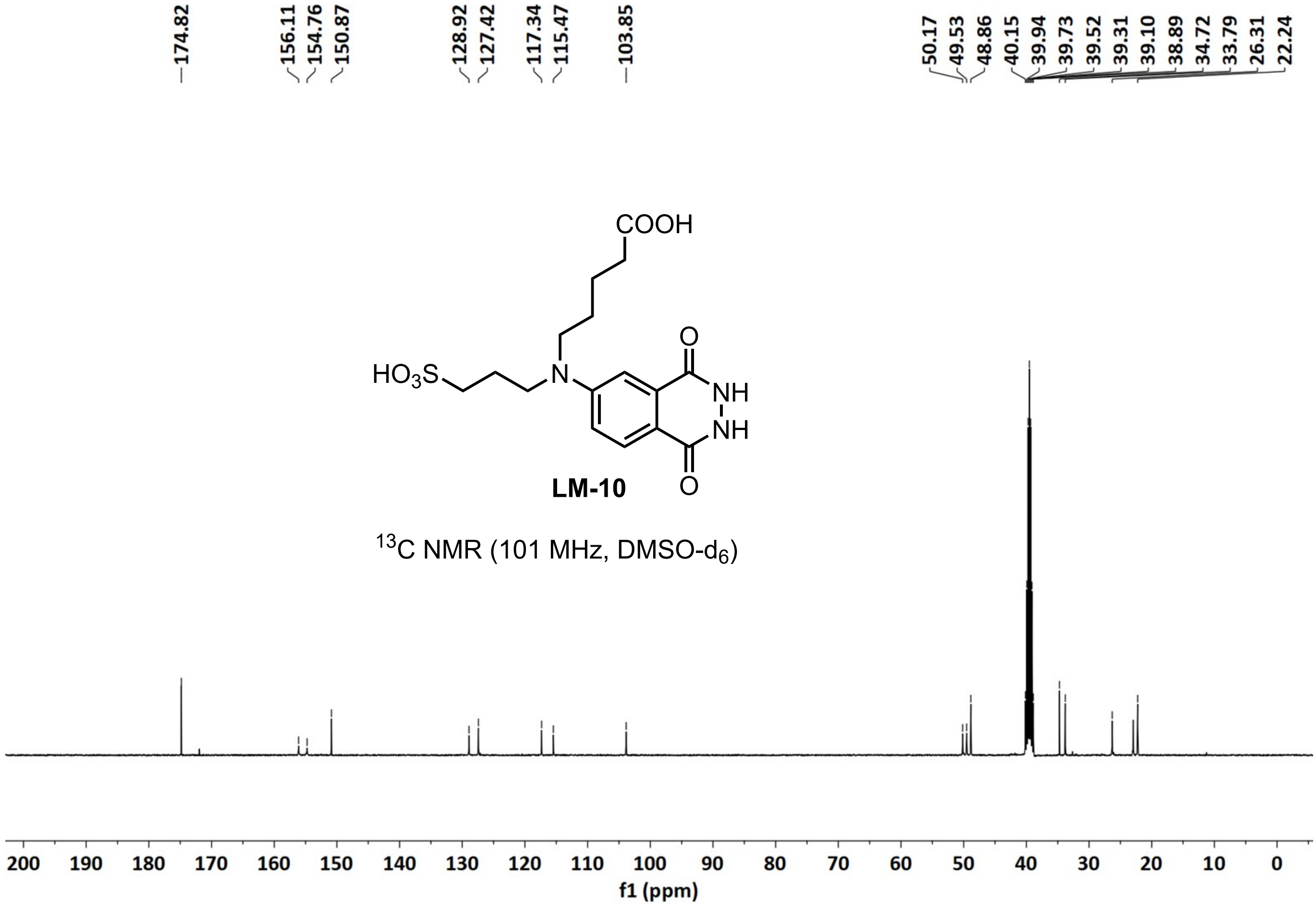
174.82
156.11
154.76
150.87
128.92
127.42
117.34
115.47
103.85
50.17
49.53
48.86
40.15
39.94
39.73
39.52
39.31
39.10
38.89
34.72
33.79
26.31
22.24
COOH
HO3S
N
O
NH
NH
O
LM-10
13C NMR (101 MHz, DMSO-d6)
f1 (ppm)

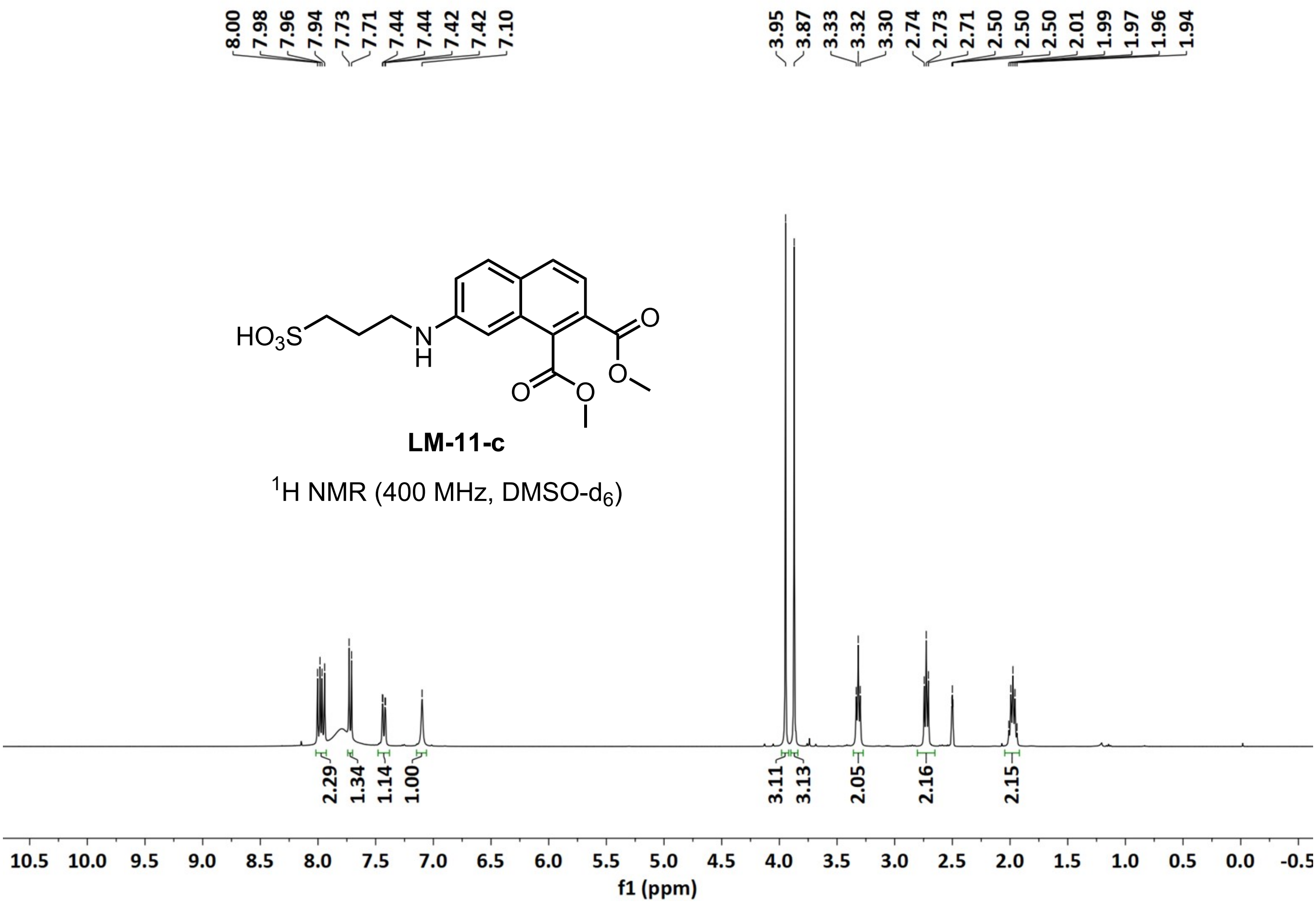
8.00 7.98 7.96 7.94 7.73 7.71 7.44 7.44 7.42 7.42 7.10
3.95 3.87 3.33 3.32 3.30 2.74 2.73 2.71 2.50 2.50 2.50 2.01 1.99 1.97 1.96 1.94
HO3S
N
H
O
O
O
O
LM-11-c
1H NMR (400 MHz, DMSO-d6)
2.29 1.34 1.14 1.00
3.11 3.13 2.05 2.16 2.15
10.5 10.0 9.5 9.0 8.5 8.0 7.5 7.0 6.5 6.0 5.5 5.0 4.5 4.0 3.5 3.0 2.5 2.0 1.5 1.0 0.5 0.0 -0.5
f1 (ppm)

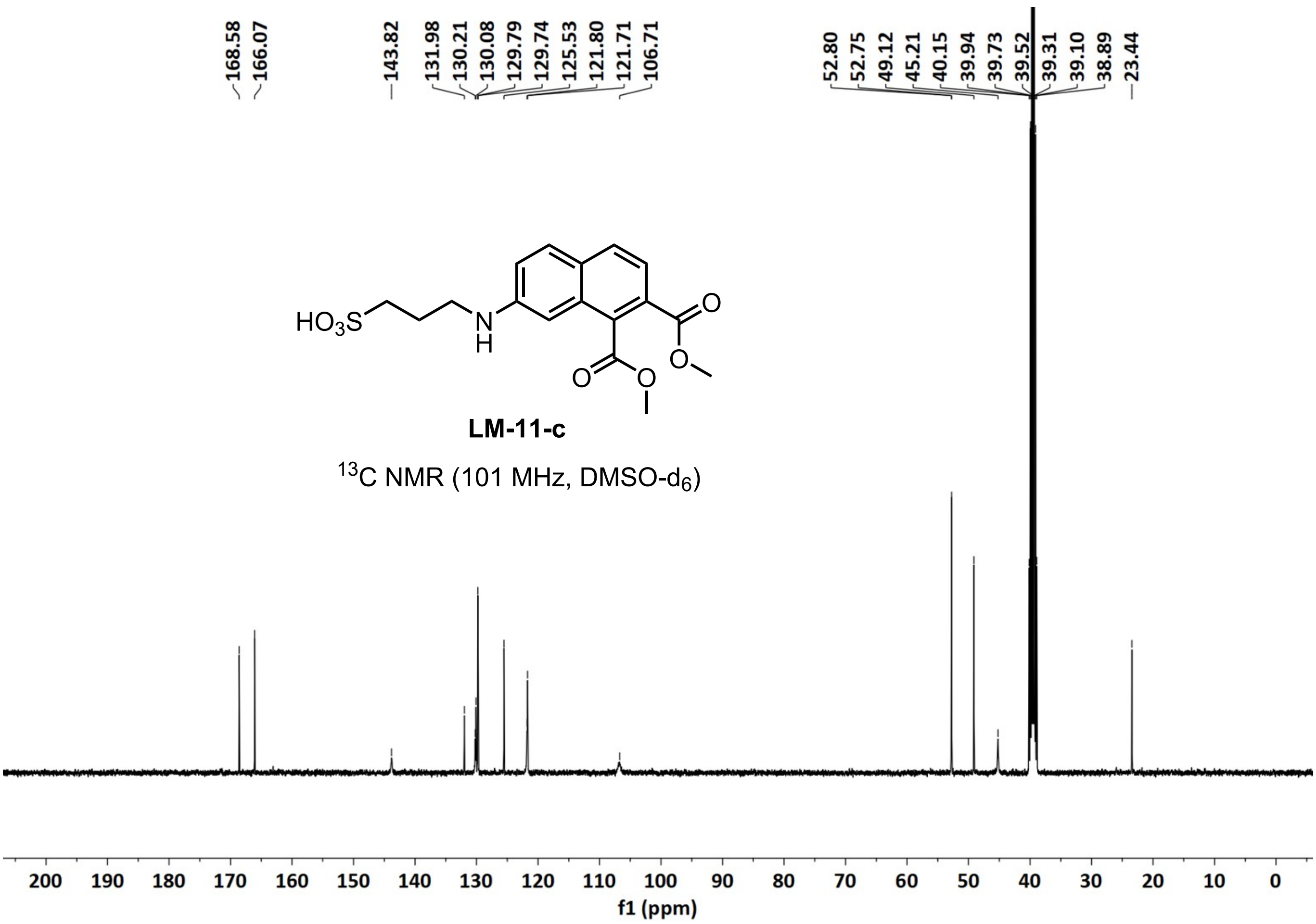
168.58 166.07
143.82 131.98 130.21 130.08 129.79 129.74 125.53 121.80 121.71 106.71
52.80 52.75 49.12 45.21 40.15 39.94 39.73 39.52 39.31 39.10 38.89 23.44
HO3S
N
H
O
O
O
O
LM-11-c
13C NMR (101 MHz, DMSO-d6)
200 190 180 170 160 150 140 130 120 110 100 90 80 70 60 50 40 30 20 10 0
f1 (ppm)

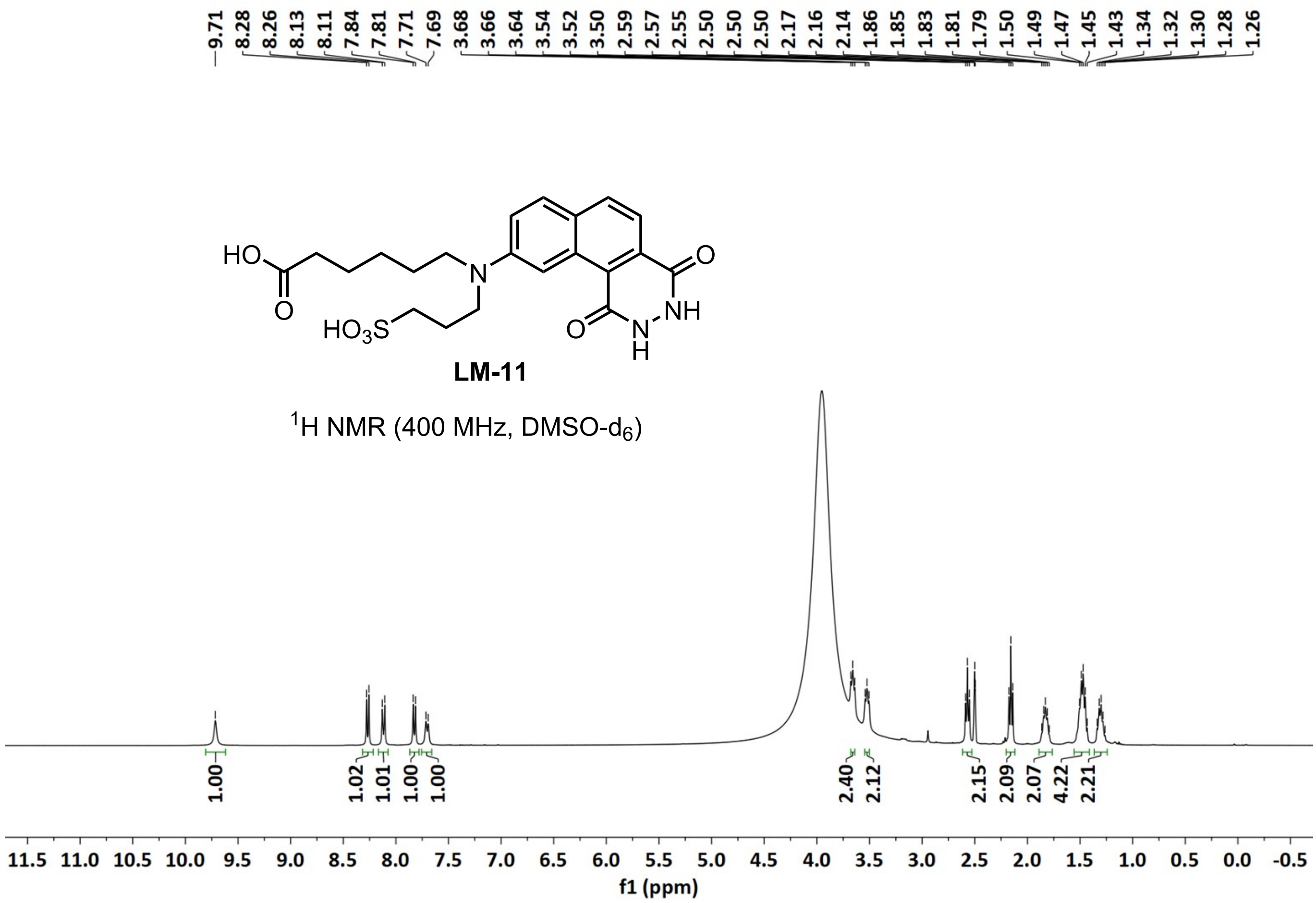
9.71
8.28
8.26
8.13
8.11
7.84
7.81
7.71
7.69
3.68
3.66
3.64
3.54
3.52
3.50
2.59
2.57
2.55
2.50
2.50
2.50
2.17
2.16
2.14
1.86
1.85
1.83
1.81
1.79
1.50
1.49
1.47
1.45
1.43
1.34
1.32
1.30
1.28
1.26
HO
O
N
HO3S
O
NH
O
N
H
LM-11
1H NMR (400 MHz, DMSO-d6)
1.00
1.02
1.01
1.00
1.00
2.40
2.12
2.15
2.09
2.07
4.22
2.21
11.5 11.0 10.5 10.0 9.5 9.0 8.5 8.0 7.5 7.0 6.5 6.0 5.5 5.0 4.5 4.0 3.5 3.0 2.5 2.0 1.5 1.0 0.5 0.0 -0.5
f1 (ppm)

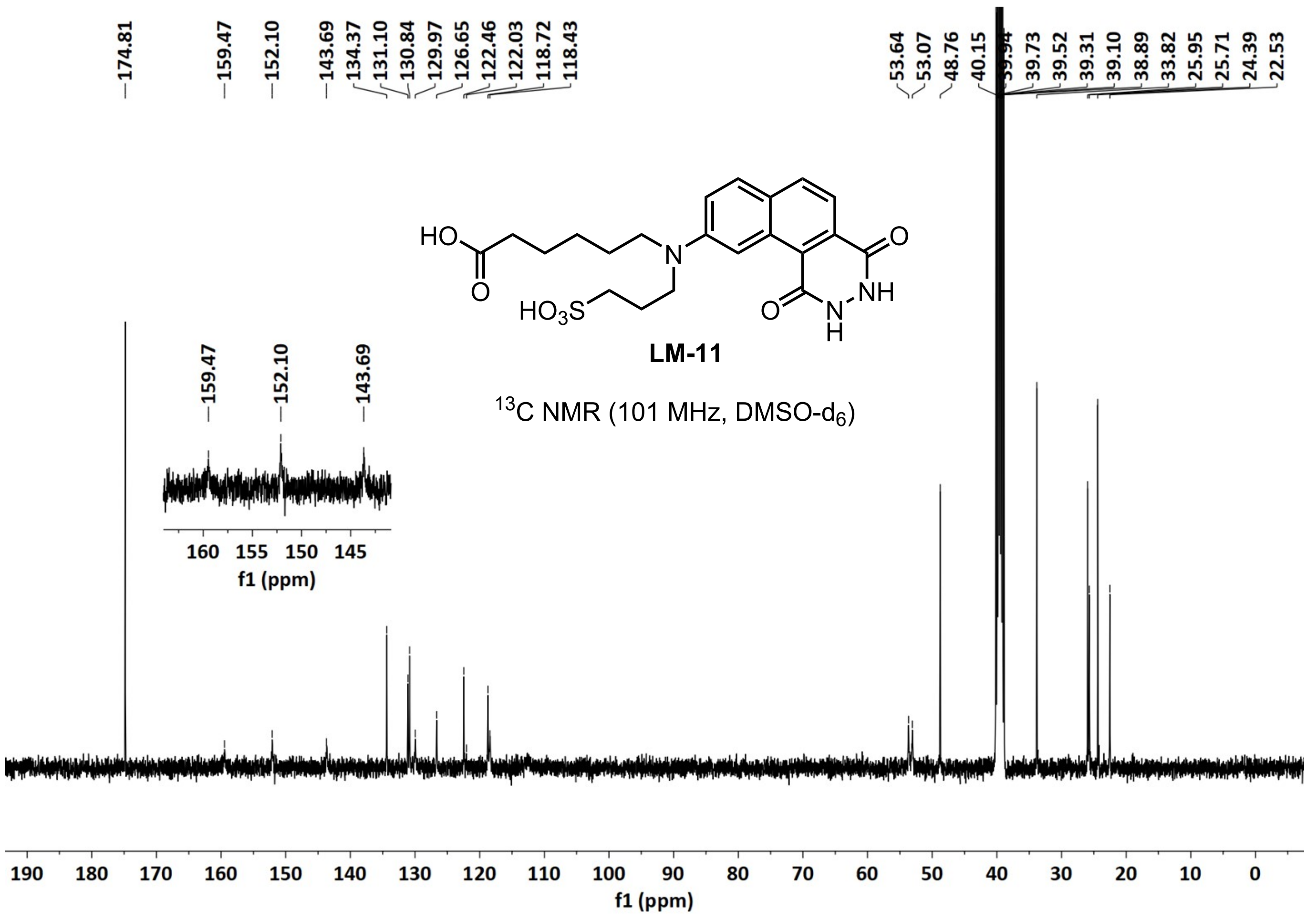
174.81
159.47
152.10
143.69
134.37
131.10
130.84
129.97
126.65
122.46
122.03
118.72
118.43
53.64
53.07
48.76
40.15
39.73
39.52
39.31
39.10
38.89
33.82
25.95
25.71
24.39
22.53
HO
O
N
HO3S
O
NH
O
N
H
LM-11
13C NMR (101 MHz, DMSO-d6)
159.47
152.10
143.69
160 155 150 145
f1 (ppm)
190 180 170 160 150 140 130 120 110 100 90 80 70 60 50 40 30 20 10 0
f1 (ppm)

Further Explanation of Broad Peaks in ^{1}H-NMR.

1) Exchangeable protons of different types (such as the hydrogens in carboxyl groups, amino groups, sulfonic acid groups, etc.) may undergo rapid proton exchange with each other (or with deuterium). In this case, the nuclear magnetic resonance instrument detects the **AVERAGE** signals of these exchangeable protons, resulting in the appearance of one broad peak. Similar phenomena have been observed in the existing literature. For detailed information, please refer to https://doi.org/10.1002/anie.202204025.

2) When the deuterated solvent is changed from DMSO-d6 to deuterated water, the broad peaks in the spectrum disappear, proving that the previous broad peaks originated from labile hydrogens，see example of **LM-7**.

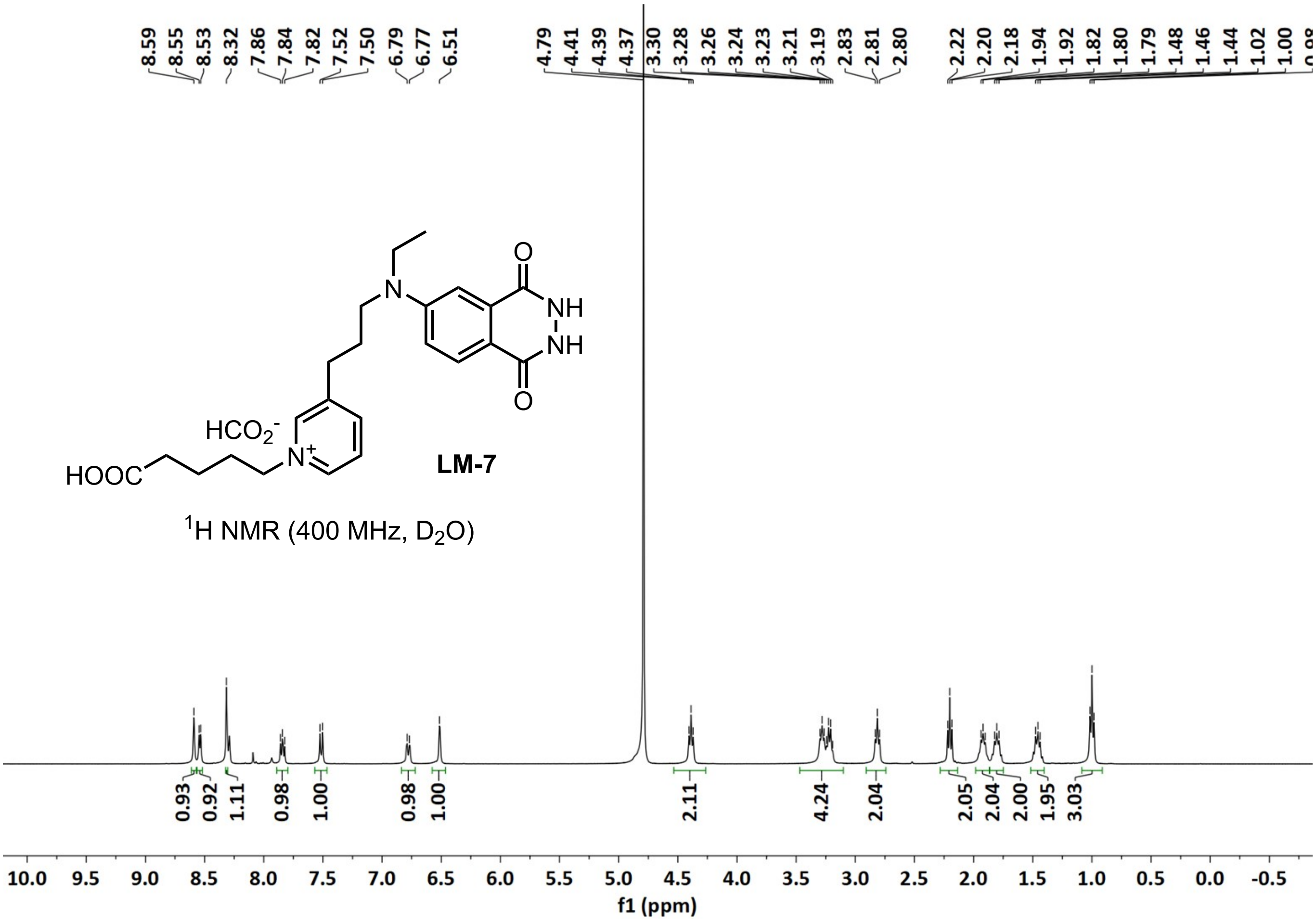

3) For the known compound **luminol**, ^{1}H-NMR experiments were conducted using DMSO-d6 and deuterated methanol as solvents, respectively. Comparative analysis of the resulting spectra revealed the absence of broad peaks in the spectrum obtained with deuterated methanol. This observation strongly suggests that the broad peaks previously observed were attributable to labile hydrogens.

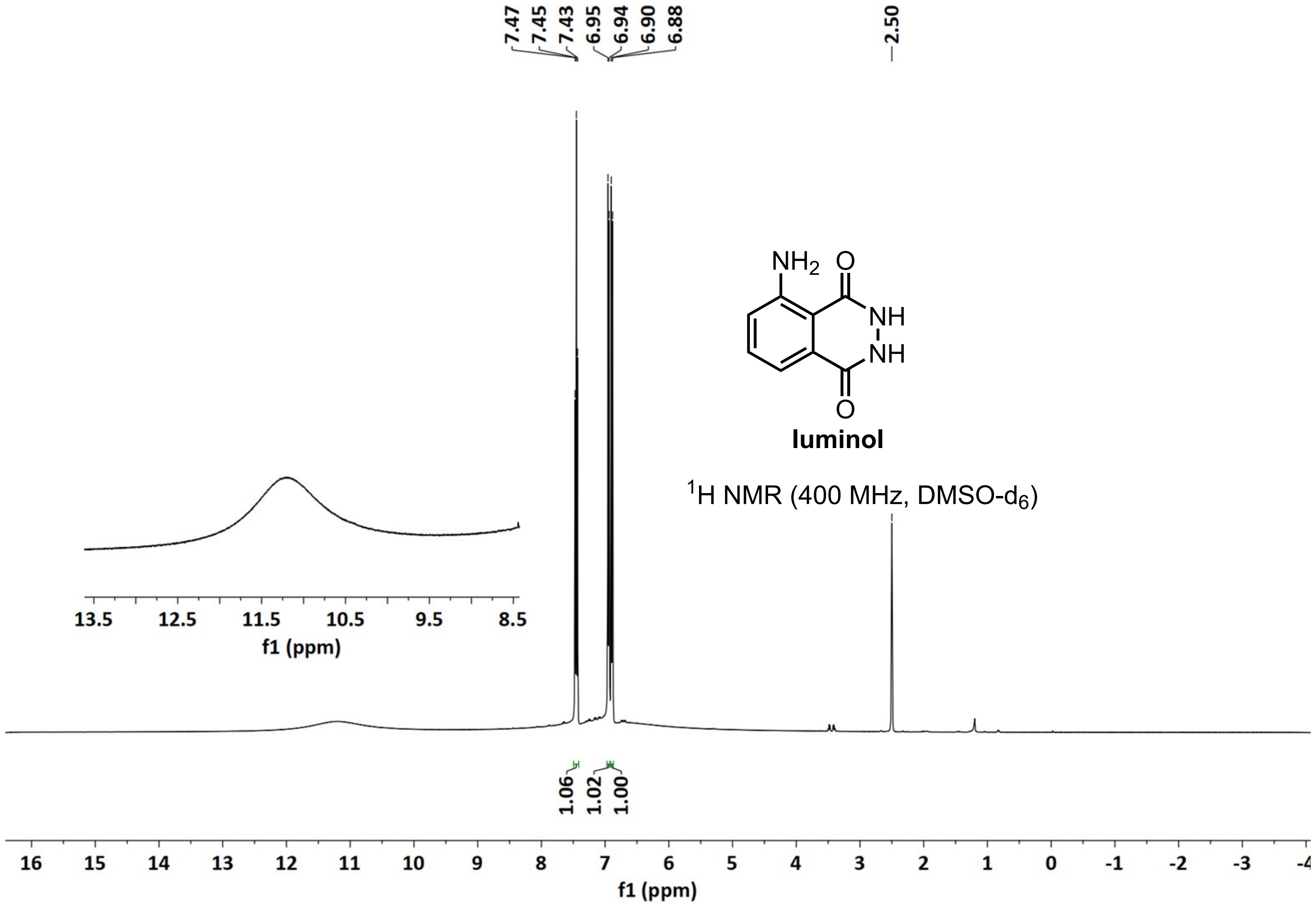
7.47
7.45
7.43
6.95
6.94
6.90
6.88
2.50
NH2
O
NH
NH
O
luminol
1H NMR (400 MHz, DMSO-d6)
13.5 12.5 11.5 10.5 9.5 8.5
f1 (ppm)
1.06
1.02
1.00
16 15 14 13 12 11 10 9 8 7 6 5 4 3 2 1 0 -1 -2 -3 -4
f1 (ppm)

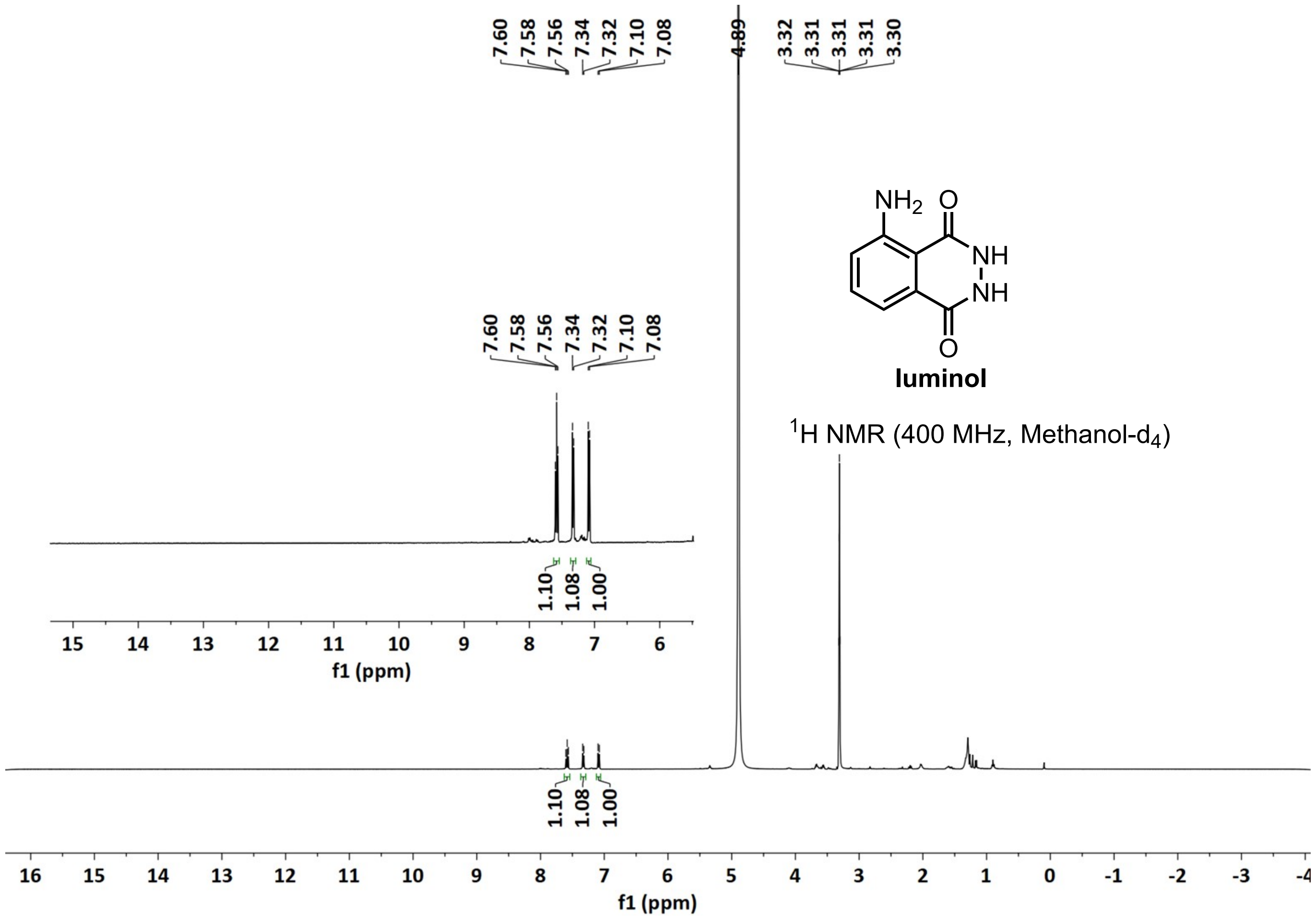
7.60
7.58
7.56
7.34
7.32
7.10
7.08
4.89
3.32
3.31
3.31
3.31
3.30
NH2
O
NH
NH
O
luminol
1H NMR (400 MHz, Methanol-d4)
1.10
1.08
1.00
15 14 13 12 11 10 9 8 7 6
f1 (ppm)
16 15 14 13 12 11 10 9 8 7 6 5 4 3 2 1 0 -1 -2 -3 -4
f1 (ppm)
